# Safety, pharmacokinetics and efficacy of HA121-28 in patients with advanced solid tumors and RET fusion-positive non-small-cell lung cancer: a multicenter, open-label, single-arm phase 1/2 trial

**DOI:** 10.1038/s41392-025-02155-5

**Published:** 2025-02-28

**Authors:** Dan-Yun Ruan, Wen-Wen Huang, Yongsheng Li, Yanqiu Zhao, Yehui Shi, Yuming Jia, Shundong Cang, Wei Zhang, Jianhua Shi, Jun Chen, Jie Lin, Yunpeng Liu, Jianming Xu, Weiwei Ouyang, Jian Fang, Wu Zhuang, Caigang Liu, Qing Bu, Manxiang Li, Xiangjiao Meng, Meili Sun, Nong Yang, Xiaorong Dong, Yueyin Pan, Xingya Li, Xiujuan Qu, Tongmei Zhang, Xianglin Yuan, Sheng Hu, Wei Guo, Yalun Li, Shengqing Li, Dongying Liu, Feixue Song, Liping Tan, Yan Yu, Xinmin Yu, Aimin Zang, Chang Sun, Qian Zhang, Kai Zou, Mo Dan, Rui-Hua Xu, Hongyun Zhao

**Affiliations:** 1 Department of Clinical Research, Sun Yat-sen University Cancer Center, State Key Laboratory of Oncology in South China, Guangdong Provincial Clinical Research Center for Cancer, Sun Yat-sen University, Guangzhou, People’s Republic of China; 2 Department of Phase 1 Ward, Chongqing University Cancer Hospital, Chongqing Cancer Hospital, Chongqing, People’s Republic of China; 3 Department of Respiratory Medicine, Henan Cancer Hospital, Affiliated Cancer Hospital of Zhengzhou University, Zhengzhou, Henan People’s Republic of China; 4 Department of Breast Oncology, Tianjin Medical University Cancer Institute & Hospital, Tianjin, People’s Republic of China; 5 Department of Oncology, The second people’s hospital of Yibin, Yibin, Sichuan People’s Republic of China; 6 Department of Medical Oncology, Phase 1 Clinical Research Unit, Department of Medical Oncology, Henan Provincial People’s Hospital, Zhengzhou, Hannan, People’s Republic of China; 7 Department of the Second General Medicine, Linyi Cancer Hospital, Linyi, Shandong People’s Republic of China; 8 Department of Pulmonary Oncology, Tianjin Medical University General Hospital, Tianjin, People’s Republic of China; 9 Department of Oncology, The Second Affiliated hospital of Kunming Medical University, Kunming, Yunnan People’s Republic of China; 10 Department of Medical Oncology, The First Hospital of China Medical University, Shenyang, Liaoning People’s Republic of China; 11 Department of Medical Oncology, The Fifth Medical Center, Chinese PLA General Hospital, Beijing, People’s Republic of China; 12 The Phase1 Clinical Center, The Affiliated Cancer Hospital of Guizhou Medical University, Guiyang, Guizhou People’s Republic of China; 13 Department of the Second Thoracic Oncology, Beijing Cancer Hospital, Beijing, People’s Republic of China; 14 Department of Respiratory Oncology, Fujian Cancer Hospital, Fuzhou, Fujian, People’s Republic of China; 15 Department of Oncology, Shengjing Hospital of China Medical University, Shenyang, Liaoning People’s Republic of China; 16 Department of Medical Oncology, The First Affiliated Hospital of Guangxi Medical University, Nanning, Guangxi People’s Republic of China; 17 Department of Respiratory and Critical Care Medicine, The First Affiliated Hospital of Xi’an Jiaotong University, Xi’an, Shanxi People’s Republic of China; 18 Department of the Fourth Thoracic Radiotherapy Ward, Shandong Cancer Hospital & Institute, Jinan, Shandong People’s Republic of China; 19 Department of Oncology, General Hospital Affiliated Shandong First Medical University, Jinan, Shandong People’s Republic of China; 20 Department of Pulmonary and Gastrointestinal Medicine, Hunan Cancer Hospital, Changsha, Hunan People’s Republic of China; 21 Department of Cancer Center, Wuhan Union Hospital of China, Wuhan, Hubei People’s Republic of China; 22 Department of Oncology Chemotherapy, The First Affiliated Hospital of USTC, Hefei, Anhui People’s Republic of China; 23 Department of the Second Oncology Ward, The First Affiliated Hospital of Zhengzhou University, Zhengzhou, Henan People’s Republic of China; 24 General Department, Beijing Chest Hospital, Beijing, People’s Republic of China; 25 Department of Oncology, Tongji Hospital, Tongji Medical College of HUST, Wuhan, Hubei People’s Republic of China; 26 Department of Oncology, Hubei Cancer Hospital, Wuhan, Hubei People’s Republic of China; 27 Respiratory Department, Shanxi Cancer Hospital, Taiyuan, Shanxi People’s Republic of China; 28 Department of Respiratory and Critical Care Medicine, West China Hospital of Sichuan University, Chengdu, Sichuan People’s Republic of China; 29 Respiratory Department, Huashan Hospital Fudan University, Shanghai, People’s Republic of China; 30 Department of Medical Oncology, The Second Hospital & Clinical Medical School, Lanzhou University, Lanzhou, Gansu People’s Republic of China; 31 Department of Respiratory Oncology, Guangxi Medical University Cancer Hospital & Guangxi Cancer Institute, Nanning, Guangxi People’s Republic of China; 32 Department of the Third Respiratory Medicine, Harbin Medical University Cancer Hospital, Harbin, Heilongjiang People’s Republic of China; 33 Department of Thoracic Oncology, Zhejiang Cancer Hospital, Hangzhou, Zhejiang People’s Republic of China; 34 Department of Medical Oncology, Affiliated Hospital of Hebei University, Baoding, Hebei, People’s Republic of China; 35 CSPC ZhongQi Pharmaceutical Technology (Shijiazhuang) Co., Ltd., Shijiazhuang, Hebei, People’s Republic of China; 36 Department of Medical Oncology, Sun Yat-sen University Cancer Center, State Key Laboratory of Oncology in South China, Guangdong Provincial Clinical Research Center for Cancer, Sun Yat-sen University, Guangzhou, People’s Republic of China; 37 Research Unit of Precision Diagnosis and Treatment for Gastrointestinal Cancer, Chinese Academy of Medical Sciences, Guangzhou, People’s Republic of China

**Keywords:** Lung cancer, Drug development

## Abstract

HA121-28, a promising multikinase inhibitor, mainly targets rearranged during transfection (RET) fusions and selectively targets vascular endothelial growth factor receptor-2, endothelial growth factor receptor, and fibroblast growth factor receptor 1-3. The safety, pharmacokinetics, and efficacy of HA121-28 were assessed in advanced solid tumors (phase 1, ClinicalTrials.gov NCT03994484) and advanced RET fusion-positive non-small-cell lung cancer (RET-TKI naive NSCLC, phase 2, ClinicalTrials.gov NCT05117658). HA121-28 was administered orally in doses range from 25 to 800 mg under the 21-day on/7-day off scheme for a 28-day cycle in phase 1 trial. The recommended dose identified in phase 1 (450 mg) was administered for patients during phase 2. The primary endpoints were the maximum tolerated dose (MTD) in phase 1 and the objective response rate (ORR) in phase 2. 162 patients were enrolled in phase 1 and 48 in phase 2. A total of 600 mg once daily was set as MTD. Across 100–800 mg, the exposure of HA121-28 increased in a dose-dependent manner. Consistent between both trials, diarrhea, rash, and prolonged QTc interval, were the most reported treatment-emergent adverse events. 40.0% (phase 1) and 62.5% (phase 2) patients experienced grade ≥3 treatment-related adverse events, respectively. The overall ORR was 26.8% and the median progression-free survival (PFS) was 5.5 months among 97 NSCLC patients with advanced RET fusion receiving a dose at ≥450 mg once daily. HA121-28 showed encouraging efficacy in advanced RET fusion NSCLC and its toxicity was tolerable in most patients. Nevertheless, cardiotoxicity is a notable concern that warrants careful attention.

## Introduction

Worldwide, non–small-cell lung cancer (NSCLC) is one of the primary causes of cancer mortality. It accounts for approximately 85% of lung cancer cases. Due to the prevalence of late-stage diagnosis, the 5-year survival rate of NSCLC is very low, at only 7%.^1,2^ Great progress has been achieved in elucidating the molecular features of NSCLC in the past few decades. The recognition of oncogenic drivers has revolutionized subsequent therapeutic strategies for the disease. A disruptive therapeutic avenue emerged with the occurrence of tyrosine kinase inhibitors (TKIs), which target the critical function of aberrant receptor tyrosine kinase activation in regulating tumor cell growth and metastasis.^3^ New desire and prolonged survival for patients with advanced or metastatic NSCLC who carried definite genetic abnormalities have been offered by TKIs, and gradually become the standard treatment for these patients. Extraordinary potential in the epidermal growth factor receptor (EGFR)-mutated NSCLC patients were initially identified following the application of TKIs in NSCLC therapy. This advancement is of pivotal significance, providing personalized treatment plans for these patients and significantly improving therapeutic outcomes. Concurrently, drug development in NSCLC has also focused more on the innovation of novel TKIs.^4^

With the expanding of genetic screening, an increasing number of oncogenes and therapeutic targets have been identified, and the expression of rearranged during transfection (RET) gene has been detected across various types of solid tumors.^5^ Physiologically, a crucial capacity in the development of enteric nervous system was elaborated by the RET gene encoding a receptor tyrosine kinase which had proto-oncogenic properties.^6–8^ Core RET kinase domain is similar to typical tyrosine kinase receptors.^9^ Pathologically, the constitutive activation of oncogenic signaling pathways (for example, PI3K/AKT, MAPK, and RAS/RAF) can occur through the mediation of ligand-independent dimerization of RET fusions that contain the RET kinase domain. This activation enhances cellular proliferation, survival, migration, and differentiation.^5,10,11^ Furthermore, the kinesin family member 5B (KIF5B)-RET and coiled-coil domain-containing protein 6 (CCDC6)-RET are the most commonly RET fusions which have been identified in 1% to 2% NSCLC patients.^12^ Per previous clinical experiences, the most RET-fusion NSCLC patients didn’t smoke and were diagnosed at a late stage. These patients typically exhibited low expression of programmed cell death protein 1 (PD-1) and had a low burden of tumor mutation.^13,14^ Therefore, upon progression after first-line chemotherapy, the options for second- and subsequent-line treatments are narrow, representing unmet medical needs in this setting.

In recent years, targeted therapy of RET fusions as an important new treatment has been disclosed in NSCLC. Promising outcomes for multi-kinase inhibitors demonstrating anti-RET activity in NSCLC have been shown in numerous clinical trials, such as Vandetanib, Cabozantinib and Lenvatinib. The relatively modest clinical efficacy and a high proportion of treatment-related toxicity was observed in those early agents. So far, Pralsetinib and Selpercatinib, two newly approved selective RET inhibitors for treatment of RET fusion-positive NSCLC, have shown their robust results. However, eventually all patients who received treatments will develop resistance to those medications.^15,16^ The novel solvent-front mutations KIF5B-RET G810C/S/R may represent an on-target mechanism of resistance to Pralsetinib and Selpercatinib.^15,16^ But off-target resistance was more common mechanism resistance to RET inhibitors. *MET* amplification, activating *PIK3CA* mutation or *PTEN* loss, *EGFR* amplification, *ERBB2* amplification, *KRAS* gain or mutation and *BRAF V600E* alteration were potential off-target resistance gene alternations to RET inhibitions.^17^ As many clinical experiences already demonstrated that significant survival benefits for NSCLC patients in treating the endothelial growth factor receptor (EGFR)-TKIs, especially in Asian populations.^18,19^ Previous in vitro study has shown that the EGFR signals was associated with RET inhibitors resistance in lung cancer cells, indicating a multi-targeted TKI with anti-RET and anti-EGFR effects may lead to improved efficacy in treating lung cancer.^20^ Moreover, tumor growth, recurrence, and microenvironment were related with angiogenesis medicated by a widely recognized therapeutic target, vascular endothelial growth factor receptor (VEGFR).^21,22^ In addition, a study using KIF5B-RET models demonstrated that KIF5B-RET fusions recruited multiple kinases, including EGFR, and upregulated the total level of VEGFR. This highlights the need for simultaneous inhibition of RET, EGFR, and VEGFR in certain tumor types.^23^ Since there are no better therapeutic options after selective RET inhibitors resistance and considering that Vandetanib and Lenvatinib inhibit EGFR- and VEGFR- dependent signaling, simultaneously RET receptor tyrosine kinase, which are all important growth drivers in NSCLC. Therefore, it is worthwhile to develop more targeted drugs against RET fusion and new RET TKIs are being developed.^24–29^

As a potent, multi-targeted TKI, selected against RET, and inhibits EGFR and VEGFR-2, HA121-28 has its half-maximal inhibitory concentrations in a nanomolar range. Preclinical studies showed that HA121-28 has robust antitumor activities in several cancer cell lines and human tumor xenograft models, with good safety profiles (Supplementary Table 11-17, Supplementary Fig. 5, preclinical data). In the phase 1 study, HA121-28 exhibited a linear pharmacokinetics (PK) profile and a tolerable safety in patients with solid tumors. The most reported treatment-related adverse events (TRAEs) were low-grade rash and diarrhea. The preliminary antitumor activity was observed among RET-fusion positive NSCLC patients (ESMO 2021, data cutoff on April 28, 2021).^30^ Here we aimed to evaluate the safety and PK profile of HA121-28 in patients with solid tumors; furthermore, the preliminary efficacy in RET fusion NSCLC patients.

## Results

### Patients

**Fig. 1:**
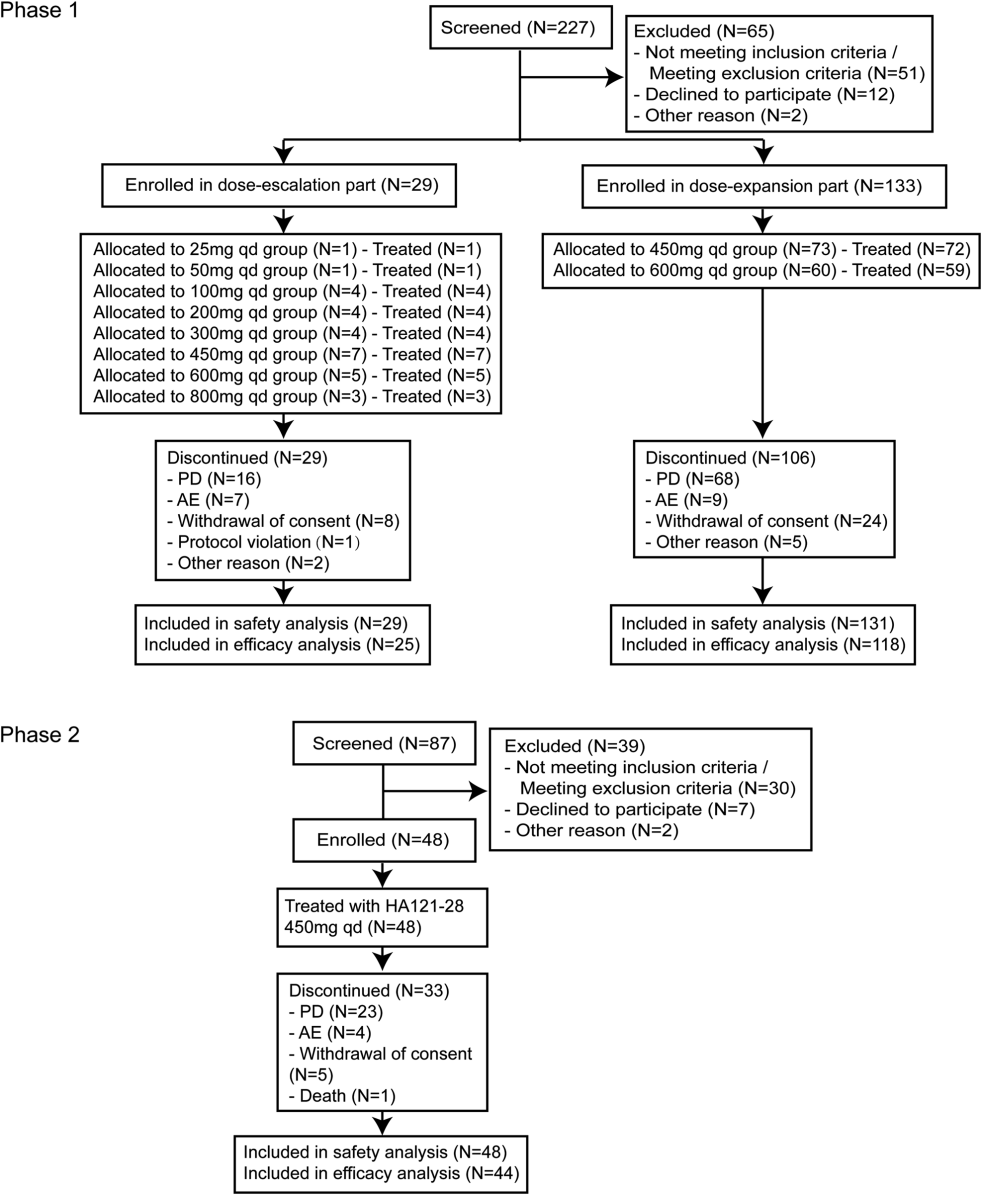
CONSORT Flow Diagram. AE adverse event, ERS efficacy response analysis set, FAS full analysis set, PD progressive disease, SS safety set, PKCS pharmacokinetic concentration set, PKPS Pharmacokinetic parameter set

In the phase 1 trial, conducted from October 2018 to April 2023, 162 patients who had diverse advanced solid tumors were enrolled from 16 study sites across China. Twenty-nine patients were included in the dose-escalation and 133 patients in the dose-expansion parts (Fig. 1). The most frequent cancer types were lung cancer (108, 67.5%), including 62 NSCLC with RET fusion-positive, colorectal cancer (14, 8.8%), and breast cancer (11, 6.9%). Most patients were heavily pretreated; 54.4% (87) underwent three prior lines of treatments (Table 1). A total of 134 (83.8%) patients discontinued from the study treatment with the main reason of disease progression (84, 52.5%) (Fig. 1). The median study follow-up time was 3.4 months (range: 0.6, 36.1) (Table 1).

**Table 1:** Demographic and disease characteristics of patients (FAS*)

Characteristic	No. (%)	
	Phase 1 (*N* = 160)	Phase 2 (*N* = 48)
Age, years, Median (IQR)	54.0 (48.0, 61.5)	53.0 (46.0, 60.0)
<60	112 (70.0)	34 (70.8)
≥60	48 (30.0)	14 (29.2)
Sex		
Male	75 (46.9)	16 (33.3)
Female	85 (53.1)	32 (66.7)
Body mass index, kg/m^2^, Median (IQR)	23.5 (21.3, 26.0)	23.2 (20.4, 25.6)
ECOG performance status		
0	43 (26.9)	13 (27.1)
1	117 (73.1)	35 (72.9)
Primary site of cancer		
Lung cancer	108 (67.5)	48 (100)
Colorectal cancer	14 (8.8)	0
Breast cancer	11 (6.9)	0
Thyroid cancer	5 (3.1)	0
Nasopharyngeal cancer	5 (3.1)	0
Others	17 (10.6)	
No. of patients with brain metastasis	22 (13.8)	22 (45.8)
No. of patients with liver metastasis	31 (19.4)	10 (20.8)
No. of prior systemic treatments		
0 line	10 (6.3)	0
1 line	26 (16.3)	26 (54.2)
2 lines	37 (23.1)	10 (20.8)
≥3 lines	87 (54.4)	11 (22.9)
Others	0	1^a^
Type of prior systemic treatments		
Prior antitumor medication	150 (93.8)	48 (100)
Prior radiotherapy	43 (26.9)	14 (29.2)
Prior cancer surgery	69 (43.1)	12 (25.0)
Prior other cancer treatment	0	2 (4.2)
Prior TKI treatment	10 (6.3)	11 (22.9)
Prior RET-TKI treatment	2 (1.3)^b^	0
Follow-up time (month)		
Median (minimum, maximum)	3.4	7.7

Data are expressed as count (percentage) unless otherwise specified. Percentages may not add up to 100% because of rounding

*FAS* full analysis set, *IQR* Interquartile range, *N(n)* count

* FAS included all patients who received at least one dose of study drug

^a^ Chinese herbal medicine with antitumor indication

^b^ One patient had PR, the other had SD as response

From March 2022 to April 2023 (data cutoff date), 48 RET-TKI naive NSCLC patients who had RET fusion-positive from 23 sites in China were enrolled and received the study drug in the phase 2 trial. Platinum-based chemotherapy was previously received for all patients. At the data cutoff date, treatment was discontinued for 33 (68.8%) patients with the primary reason of disease progression (23, 47.9%) (Fig. 1). The median study follow-up time was 7.7 months (range: 0.4, 12.5) (Table 1).

### Safety in the phase 1 and 2

In the phase 1 trial, almost all patients (159, 99.4%) experienced at least one treatment emergent adverse event (TEAE), and 48.8% (78) of patients reported grade ≥ 3 TEAEs (Supplementary Table 1). Overall, the most common TEAEs were diarrhea (126, 78.8%), rash (92, 57.5%), and prolonged QT interval (80, 50.0%). The most frequent grade ≥ 3 TEAEs was prolonged QT interval (24, 15.0%), followed by diarrhea (13, 8.1%) and rash (9, 5.6%) (Supplementary Table 2). One hundred fifty-seven (98.1%) patients reported at least one TRAE, and 64 (40.0%) patients experienced grade ≥3 TRAEs. Diarrhea, rash, and prolonged QT interval were also the most frequent TRAEs (Table 2, Supplementary Table 3). The predefined adverse event of special interest (AESI) ( ≥ grade-3 prolonged QT interval) occurred in 24 (15.0%) patients, leading to dose interruption in 17 (10.6%) patients, and permanent discontinuation in one (0.6%) patient. Among the 24 patients who reported ≥ grade-3 prolonged QT interval, 9 also reported cardiac adverse events, mostly were low in grade, and only one case of grade-4 ventricular arrhythmia (Supplementary Table 7). The median duration of AESI was two days (Interquartile range 1–7). Serious adverse events (SAEs) occurred in 34 (21.2%) patients; treatment-related SAEs were reported by 23 (14.4%) patients. Among these, only rash (4, 2.5%) was reported in more than three patients (Supplementary Table 4). Eleven patients had AEs leading to death within the safety observation period (within 28 days of the last dose of the study drug); Four of them in the 600 mg group were suspected to be treatment-related (one case of being discontinued treatment due to abnormal liver and kidney function, and eventually died due to tumor progression; one case of hemoptysis, one sudden death of unknown cause and one of sudden cardiac arrest). The patient died following a sudden cardiac arrest, never reported QT interval prolongation and no obvious cause can be found. Eleven deaths occurred during the survival follow-up period, which were mainly attributed to disease progression and unknown causes.

**Table 2:** Treatment-related Adverse Events (SS*)

Preferred Term	No. (%)			
	Phase 1 (*N* = 160)		Phase 2 (*N* = 48)	
	All	≥ Grade 3	All	≥ Grade 3
Any	157 (98.1)	64 (40.0)	46 (95.8)	30 (62.5)
Diarrhea	122 (76.3)	11 (6.9)	35 (72.9)	5 (10.4)
Rash	90 (56.3)	9 (5.6)	29 (60.4)	1 (2.1)
Prolonged QT interval	79 (49.4)	24 (15.0)	20 (41.7)	14 (29.2)
Proteinuria	48 (30.0)	0	16 (33.3)	0
ALT increased	54 (33.8)	2 (1.3)	20 (41.7)	2 (4.2)
AST increased	52 (32.5)	3 (1.9)	19 (39.6)	1 (2.1)
Hypokalemia	27 (16.9)	4 (2.5)	8 (16.7)	3 (6.3)
Blood creatinine increased	36 (22.5)	2 (1.3)	8 (16.7)	0
Nausea	40 (25.0)	2 (1.3)	3 (6.3)	0
Vomiting	32 (20.0)	2 (1.3)	5 (10.4)	0
Hypoalbuminemia	21 (13.1)	1 (0.6)	4 (8.3)	0
Decreased appetite	28 (17.5)	3 (1.9)	4 (8.3)	1 (2.1)
Asthenia	22 (13.8)	0	6 (12.5)	0
Anemia	14 (8.8)	1 (0.6)	5 (10.4)	0
Fecal occult blood positive	10 (6.3)	0	0	0
Hypertriglyceridemia	13 (8.1)	0	5 (10.4)	0
Hypertension	17 (10.6)	6 (3.8)	10 (20.8)	8 (16.7)
White blood cell count decreased	13 (8.1)	2 (1.3)	8 (16.7)	1 (2.1)
Blood ALP increased	14 (8.8)	0	5 (10.4)	0
Dizziness	10 (6.3)	0	3 (1.9)	0
Neutrophil count decreased	13 (8.1)	2 (1.3)	7 (14.6)	2 (4.2)
Platelet count decreased	13 (8.1)	1 (0.6)	6 (12.5)	0
Hyponatremia	10 (6.3)	1 (0.6)	6 (12.5)	0
GGT increased	13 (8.1)	3 (1.9)	6 (12.5)	0
Sinus tachycardia	8 (5.0)	0	5 (10.4)	0
Cough	2 (1.3)	0	0	0
Weight loss	7 (4.4)	0	5 (10.4)	0
Electrocardiogram T wave abnormal	9 (5.6)	0	0	0
COVID-19	0	0	0	0
Urinary tract infection	2 (1.3)	0	0	0

Data are expressed as count (percentage). Percentages may not add up to 100% because of rounding

*SS included all patients who received at least one dose of the study drug and had safety data recorded in the phase 1 and phase 2 studies. Treatment-related adverse events are summarized by Preferred Term according to MedDRA for events occurring in ≥ 2 patients in SS

*ALP* alkaline phosphatase, *ALT* alanine aminotransferase, *AST* Aspartate aminotransferase, *GGT* γ-glutamyl transpeptidase, *TRAE* treatment-related adverse events, *SS* safety set

In the phase 2 trial, all patients reported at least one TEAE, and 46 (95.8%) reported at least one TRAE (Table 2, Supplementary Table 1). The most common grade ≥ 3 TEAEs were prolonged QT interval (15, 31.3%), diarrhea (6, 12.5%), and hypertension (9, 18.8%), which were also the most common grade ≥ 3 TRAEs (Supplementary Table 5). Among the 15 patients who reported ≥ grade-3 prolonged QT interval, 7 patients (14.6%) experienced dose interruption, 3 patients (6.3%) required dose reduction, and there were no cases of permanent discontinuation; 9 also reported cardiac adverse events, and all were low in grade (Supplementary Table 7). Nineteen (39.6%) patients experienced at least one SAE and 9 (18.8%) were treatment-related (Supplementary Table 4). Five deaths occurred during the safety observation period, which were associated with pneumonia, embolism, disease progression, and one unknown cause of death, respectively, but none were deemed treatment related. Another seven deaths were found during the survival follow-up period, including one case of septic shock, one case of respiratory failure, four cases of disease progression, and one unknown cause of death.

In phase 1 and phase 2 trials, the incidence of ≥ grade-3 TRAE was 58.1% in 43 patients with previous immunotherapy compared to 41.2% in those without (165 patients). The incidence of ≥ grade-3 TRAEs with a difference of ≥ 5% between the two subgroups (with and without previous immunotherapy) included: prolonged QT interval (27.9% vs 15.8%), hypertension (0 vs 8.5%), and decreased neutrophil counts (7.0% vs 0.6%). The incidence of other ≥ grade-3 TRAEs was similar between patients with and without previous immunotherapy (Supplementary Table 6).

### Efficacy in the phase 1 and 2

Across the phase 1 and phase 2 trials, 97 efficacy evaluable NSCLC patients with RET fusion-positive received HA121-28 at 450 mg (q.d.) or above and were evaluated for efficacy. Eight of these patients were treatment-naive, and 89 (91.8%) had previously received first-line chemotherapy. Seventy-one patients (73.2%) harbored a KIF5B-RET fusion and 15 (15.5%) with a CCDC6-RET fusion. Twenty patients (20.6%) had prior multikinase inhibitors treatment and 52 (53.6%) had prior PD-(L)1 inhibitor (Supplementary Table 8).

**Fig. 2:**
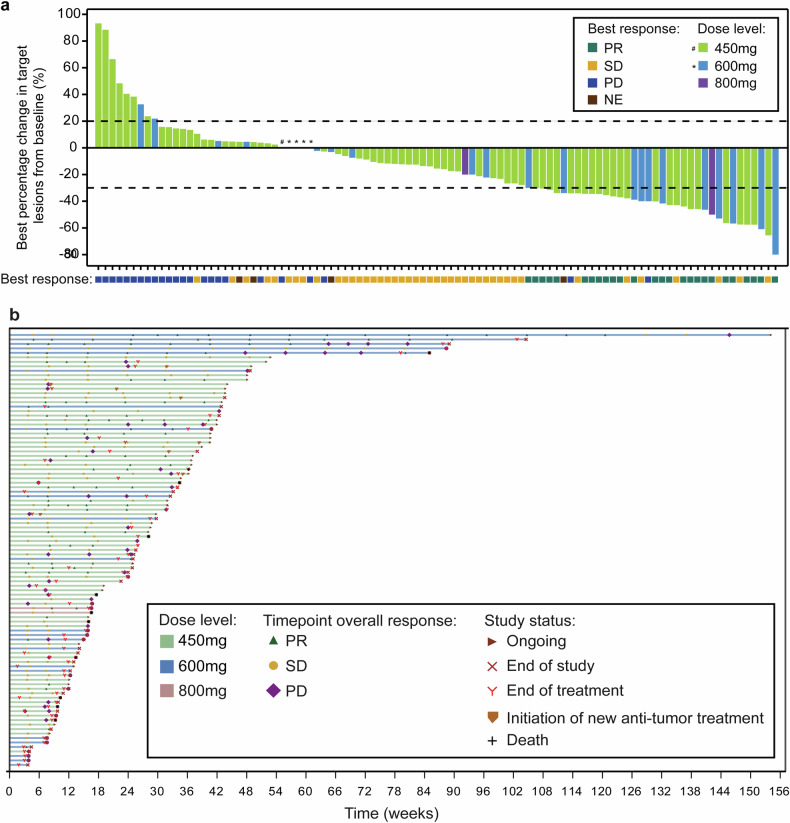
Tumor Response. **a** Waterfall plot of best percent change from baseline in the sum of longest diameters of target lesions in patients with RET-fusion positive NSCLC. **b** Swimmer plot according to dose level in patients with RET-fusion positive NSCLC. Best response is evaluated as per RECIST 1.1. NE not evaluable, NSCLC non-small-cell lung cancer, PD progressive disease, PR partial response, RET rearranged during transfection, SD stable disease

**Fig. 3:**
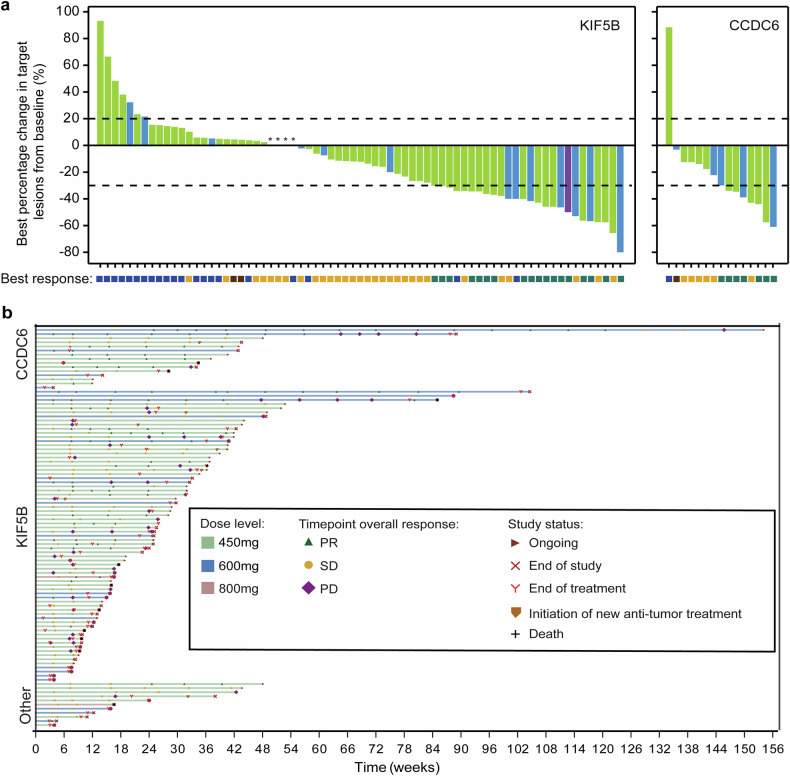
Tumor Response of KIF5B or CCDC6-RET Fusion. **a** Waterfall plots of best percent change from baseline in the sum of longest diameters of target lesions in patients with a KIF5B or CCDC6-RET fusion. **b** Swimmer plot according to dose level and RET-fusion type. Best response is evaluated as per RECIST 1.1. CCDC6 coiled-coil domain-containing protein 6, KIF5B kinesin family member 5B, NE not evaluable, NSCLC non-small-cell lung cancer, PD progressive disease, PR partial response, RET rearranged during transfection, SD stable disease

Overall, the investigator-assessed confirmed ORR in NSCLC patients with RET fusion-positive was 26.8% (95%CI 18.3–36.8%), the DCR was 72.2% (95%CI 62.1–80.8%), and 26 (26.8%) patients with a partial response (PR) (Supplementary Table 9). In patients who carried a CCDC6-RET fusion, the confirmed ORR by investigator assessment was 46.7% (95%CI 21.3–73.4%) and the median duration of response (DOR) was 14.0 months (95% CI 5.8-NE). In patients who had KIF5B-RET fusion, the confirmed ORR by investigator assessment was 25.4% (95%CI 15.8–37.1%) with the median DOR of 7.7 months (95% CI 3.7-NE) (Supplementary Table 9). As shown in Figs. 2 and 3, a shrinkage in tumor size was experienced in 66 (68.0%) patients, and in 14 (out of 15) patients with a CCDC6-RET fusion. 55 patients had disease progression or death with a median PFS of 5.5 months (95% CI 3.8–7.6) (Supplementary Fig. 1a). The median PFS was 14.9 months (95% CI 6.5-NE) in CCDC6-RET fusion patients compared to 5.5 months (95% CI 3.6–7.0) in KIF5B-RET fusion patients (Supplementary Fig. 1b, c). In subgroup analysis, though the ORR was similar across various subgroups, we could observe numerically higher ORR in some populations, such as age < 60 (30.9%), ECOG PS of 0 (35.5%), and with live metastasis (37.5%) (Supplementary Fig. 2). Similar ORR was observed regardless of brain metastasis or prior TKI therapy. In phase 1, only two patients with previous RET-TKI treatment were enrolled. One patient achieved a confirmed response of PR, while the other had stable disease (Table 1). According to the exclusion criteria, all patients enrolled in phase 2 were RET-TKI naive. In dose expansion of phase 1 trial, no response was found for 73 patients with non-RET fusion tumors.

### Genomic alternations in the phase 1 and 2

**Fig. 4:**
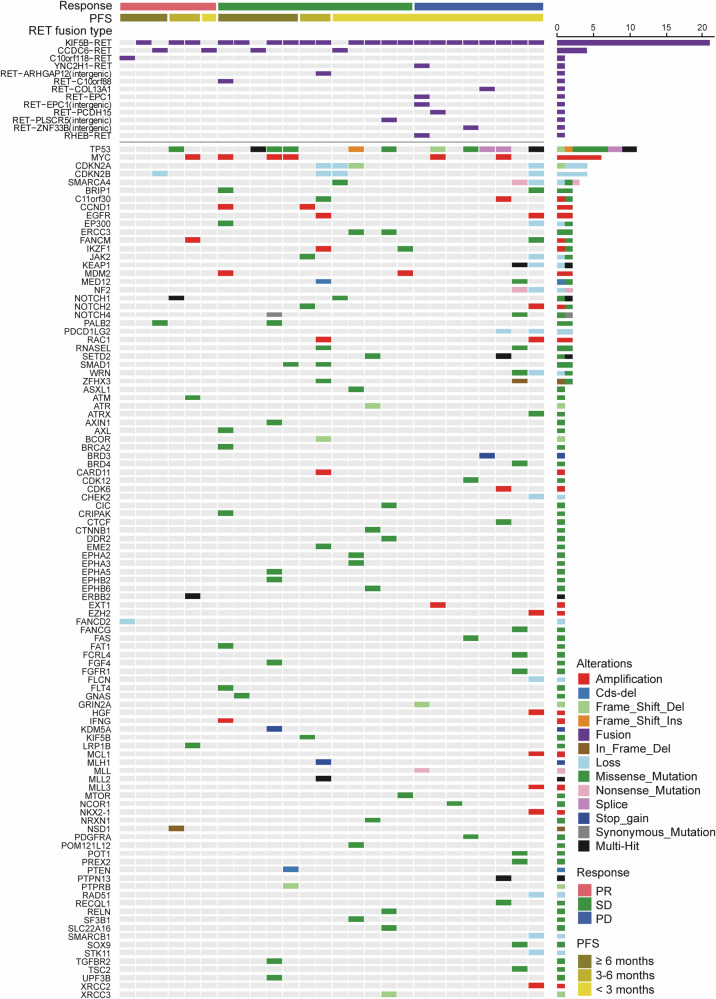
Baseline mutation landscape in RET-fusion positive NSCLC patients. NSCLC non-small-cell lung cancer, PD progressive disease, PFS progression-free survival, PR partial response, RET rearranged during transfection, SD stable disease

A total of 26 tumor tissues collected from RET-fusion positive NSCLC patients were performed targeted exome sequencing by the same panel covering 1021 genes. The overall pattern of existing genomic alterations reflected the foundation of tumor lineage (Fig. 4). Besides the RET fusion, 5 out of 15 (33.3%) patients in the *TP53* wild-type group achieved partial response (PR). In contrast, only 1 out of 11 (9.1%) patients with concurrent *TP53* mutation had PR (Supplementary Fig. 3a). A patient with *SMARCA4*, *SMARCB1*, and *EZH2* alterations (epigenetic alterations) and a patient with *SMARCA4* mutation had progressive disease (PD) and short PFS. Another *SMARCA4* mutation occurred in a stable disease (SD) patient with short PFS (PFS of 1.8 months) (Supplementary Fig. 3b).

### Pharmacokinetics in the phase 1

In the phase 1 dose-escalation part, a T_max_ of approximately 4–24 h was observed, indicating a slow absorption of HA121-28 following doses range from 25 mg to 800 mg (Supplementary Table 10). The plasma concentration versus time curves showed a slow decline post-peak with mean t_1/2z_ ranging from 74.1 to 111.6 hours (Supplementary Fig. 4). Across 25 mg to 800 mg of HA121-28, the PK exposure (C_max_, AUC_0-t_, AUC_0-24_, AUC_0-24, ss_) increased approximately in a dose-proportional manner after receiving a single- and multiple-dose. Significant accumulation was found upon multiple dosing (Supplementary Table 10). The steady state was achieved approximately after two weeks.

### MTD/ The recommended phase 2 dose

In the dose-escalation part of phase 1, a total of eight dose levels were evaluated. At the 800 mg (q.d.) dose level, two patients experienced dose-limiting toxicities, which was a grade-3 decreased appetite and a grade-3 prolonged QT interval. At 600 mg/day dose level, the incidence of ≥ grade-3 TEAEs was higher compared to 450 mg/day (68.8% vs. 36.7%, as shown in Supplementary Table 1). A similar trend was observed for grades 4 and 5 TEAEs. The MTD was set to 600 mg (q.d.) considering the PK profiles, safety, and preliminary efficacy results of HA121-28. The recommended dose levels for the dose-expansion part in phase 1 were 450 mg and 600 mg (q.d.), while 450 mg (q.d.) in phase 2 (the recommended phase 2 dose, RP2D).

## Discussion

The safety, PK, and preliminary efficacy of HA121-28 were evaluated in the studies, and our results demonstrated that HA121-28 was tolerated across the dose range of 25–600 mg in patients who had advanced solid tumors. An ORR of 26.8% was observed, demonstrating evident antitumor activity in NSCLC patients with RET fusion-positive. A median PFS of 5.5 months was also seen indicating a modest signal of survival benefit. Interestingly, more favorable data were observed in CCDC6-RET fusions compared to KIF5B-RET fusions (ORR 46.7% vs 25.4%; DOR 14.0 vs 7.7 months).

Similar safety profile was observed in HA121-28 to that of other multi-targeted TKIs with inhibiting activity against RET, such as Vandetanib, mainly characterized by QTc interval prolongation (Supplementary Fig. 5), rash and diarrhea.^25,26,31^ Grade-3 and above TRAEs in phase 1 trial were commonly associated with VEGFR inhibition; the predefined AESI, QTc interval prolongation, was observed in our studies, but all were well-managed by dose suspension or reduction, and led to no serious complications. And more than half of the events lasting less than 10 days with outcomes of recovery. Only one patient in phase 1 trial who experienced several QTc interval prolongations required permanent drug discontinuation while the outcome was recovered. Except for one grade-4 ventricular arrhythmia, all other cardiac events in patients who reported a grade-3 or higher prolonged QT interval were grade 1 or 2. In light of the incidence of QTc interval prolongations observed in this study, it would be valuable to further investigate potential risk factors for high-grade QTc prolongation and propose measures to mitigate this risk. Additionally, it is important to consider the cardiac risk for patients in real-life settings.^32^

With the recognition of driver genes, as well as the research and development of molecular target medications, biomarker-based regimens have become the standard treatments for advanced NSCLC harboring corresponding genetic mutations. Several EGFR- and ALK-TKIs are marketed worldwide for EGFR-mutated or ALK-positive NSCLC therapy. However, the treatment options for RET fusion-positive NSCLC are still very limited. Studies on some multi-targeted TKIs exhibiting activity on RET were conducted in NSCLC patients with RET fusion-positive, including Cabozantinib,^24^ Vandetanib,^25,33^ Lenvatinib,^26^ Ponatinib,^34^ Sunitinib,^35^ and Sorafenib.^36^ Among those TKIs, low to moderate antitumor activity was observed, but none received FDA approval for this indication. To date, the FDA only approved two novel drugs, Selpercatinib and Pralsetinib, as the highly selective RET inhibitors, for treatment NSCLC with RET fusion-positive, both showing an outstanding ORR of 61% and 61% in platinum-pretreated patients.^37,38^ In the treatment of NSCLC and other tumors, targeting RET fusion has been identified as an important aspect. However, there remails an unmet clinical need within this group of patients. As tumor heterogeneity and the possibility of sequential resistance, combined therapy has been proposed as a promising strategy. HA121-28 is developed as a multi-targeted TKI, which can selectively against RET and inhibits EGFR and VEGFR-2. In our study, one out of two patients with previous RET-TKI treatment in phase 1 had a confirmed response of PR (PFS 5.36 months, dosing 167 days), another had SD (PFS 5.52 months, dosing 176 days) (Table 1). Although the sample size of patients with previous RET-TKI treatment was small, simultaneous inhibition of RET, EGFR, and VEGFR by HA121-28 might be a viable treatment option for such patients. Therefore, it is worthwhile to further explore the benefit population of HA121-28, and develop more targeted drugs against RET fusion.

In the treatment of NSCLC with RET-fusion-positive, HA121-28 may be helpful for patients who have experienced disease progression following the first-line treatment with Selpercatinib or Pralsetinib. The antitumor activity of HA121-28 was suboptimal compared to Selpercatinib and Pralsetinib in this area. This because RET-independent pathways may drive most of resistance to these drugs, the selective RET inhibitions.^17^ In preclinical study, the epidermal growth factor (EGF) could trigger resistance to RET inhibition, which was reversed by EGFR-TKI.^20^ RET rearrangement has been believed to be mutually exclusive with the EGFR and other oncodrivers, while coexistent alterations were found in a few patients recently.^39^ Interestingly, simultaneous inhibition of RET, EGFR, and VEGFR, and HER2 by HA121-28 may be beneficial for such patients (Supplementary Table 11), but further studies on resistance should be considered. Nevertheless, HA121-28 could be a viable treatment option for patients when highly selective RET inhibitors are not accessible or in progression of disease.^29^

Objectively, modest antitumor activity of HA121-28 was observed in our studies. However, a more notable ORR (46.7%) and median PFS (14.9 months) were seen for NSCLC patients with CCDC6-RET fusion. Such favorable responses in patients harboring a CCDC6-RET fusion partner were also observed in the previous studies of Vandetanib and RXDX-105 compared to KIF5B-RET patients.^25,29^ Even though the possible mechanism behind the higher response and longer PFS in CCDC6-RET fusion was still unclear, more powerful signaling activity were observed in some studies for KIF5B fusions, compared to CCDC6 and NCOA4 fusions. It is not the unique instance where poorer antitumor responses to Selpercatinib and RXDX-105 have been associated with the higher expression levels of KIF5B.^29^

In addition, some possible mechanisms of poorer responses were found in KIF5B-RET NSCLC patients.^16^ First, mutations at KIF5B-RET L730V/I were relevant mechanisms of Pralsetinib resistance while remained sensitive to Selpercatinib.^23^ Second, frequently acquired MET amplification in KIF5B-RET NSCLC, described as acquired off-target resistance to selective RET inhibitors, was observed after treatment with Selpercatinib.^40^ For KIF5B-RET, through combination of kinesin and kinase domains, a ‘signaling hub’ RET-SRC-EGFR-FGFR was established, whereas RET inhibition alone was ineffective in KIF5B-RET models.^23^ Therefore, it is worth exploring for the favorable responses of HA121-28 in patients harboring a CCDC6-RET fusion partner in more clinical trials. Besides, the potential to improve activities of multikinase inhibitors, especially for KIF5B–RET tumors, warrants further investigation.^29^ Given the small sample size in the CCDC6-RET fusion group and cases with progression in this study, these results should be interpreted cautiously.

Concurrent *TP53* mutations and RET-fusion-positive are not yet well defined in tumor genomic features and their roles. In this study, the concurrent *TP53* mutated patients had poorer clinical responses compared to the wild type. Despite the small sample size, the values of ORR and DCR showed numerically different between *TP53* mutation and *TP53* wildtype. Thus, further research is needed. Similar results were observed in the Selpercatinib study, a shorter median PFS was observed in concurrent *TP53* mutated patients.^40^ The negative action of *TP53* concurrent mutation to TKI monotherapy in NSCLC has been reported, and TKI plus chemotherapy may offer greater benefits to these patients.^41,42^ Therefore, the strategy of combination therapy may be an alternative option for *TP53* co-mutation patients. Epigenetic alterations, like *EZH2*, may adjust resistance to targeted therapies in tumors, particularly in those with unclear genetic resistance mechanisms.^43,44^ Indeed, similar findings in EGFR mutated lung adenocarcinoma were discovered in *SMARCA4/A2* subunits, which played pivotal roles in adjusting the resistance to Osimertinib.^45^ Besides, *SMARCA4* has been identified as a unique factor for tumor maintenance and oncogenicity.^46^ In our study, compared to the wild type, patients with concurrent *SMARCA4/SMARCB1/EZH2* alterations trended toward worse clinical response to HA121-28. Concurrent *SMARCA4/SMARCB1/EZH2* mutations in NSCLC with RET fusions, in conjunction with selective RET inhibition, have not been reported yet, worth to be validated by further analysis.

We acknowledge several limitations of our studies. The major limitation of this study is that HA 121-28 was tested in patients almost without prior RET-TKI exposure, since the current standard treatment for NSCLC patients with RET fusion is Selpercatinib and Pralsetinib, the antitumor activity of HA121-28 after Selpercatinib or Pralsetinib treatment remains unknown. Due to the rarity of NSCLC with RET-fusion-positive, the single-arm design was adopted in phase 2. The inherent bias in results interpretation may exist. In addition, since our phase 2 trial is ongoing, the number of patients was still insufficient to draw a definitive conclusion on HA121-28 in NSCLC with RET-fusion.

In conclusion, HA121-28 demonstrated a promising antitumor activity in NSCLC with RET fusion; especially, CCDC6-RET patients showed a numerically higher response, and the cardiac risk associated with HA121-28 should be noted. Further studies are warranted to evaluate HA121-28 and its combination with other active drugs in cancer patients with RET-fusion.

## Materials and methods

### Study design and participants

#### Phase 1

The phase 1 trial was a two-part, multicenter, open-label, nonrandomized study to evaluate the safety and preliminary efficacy of HA121-28.

According to the U.S. Food and Drug Administration (FDA) guidelines,^47^ the starting dose of HA121-28 was selected based on the no observed adverse effect level in rats (the most sensitive species) with a safety factor of 5. In rats, the no observed adverse effect level was clarified as 15 mg/kg. Supposing 60 kg as the average human body weight, the starting dose of human was approximately 28.8 mg. The starting dose was set to 25 mg orally once a day (q.d.).

Safety, tolerability, and PK of HA121-28 was assessed in dose-escalation part. The single-patient accelerated titration was carried out at 25 mg and 50 mg; then, the dose escalation proceeded according to a “3 + 3” design in dose range of 100 to 800 mg. Escalation to a higher dose level was considered by the satisfactory review of safety results from the previous lower dose during the first cycle.

The dose-expansion part aims to further access the safety and efficacy of HA121-28 at the selected doses based on data from the dose-escalation part. The RP2D was determined by investigators in view of a thorough review of safety, PK, and efficacy results.

Patients aged 18–75 years (inclusive) patients who had locally advanced or metastatic solid tumors through histopathological or cytological examinations were enrolled. Additional key eligibility criteria included patients must be relapsed/refractory or intolerant to at least one previous line of standard treatment (except for RET fusion-positive tumor), must have had at least one measurable lesion at baseline assessed by Response Evaluation Criteria in Solid Tumors version 1.1 (RECIST 1.1); 0 or 1 for an Eastern Cooperative Oncology Group performance status; at least three months for a life expectancy; adequate function in hematological, hepatic, cardiac and renal. The RET fusion was tested locally.

#### Phase 2

The phase 2 trial was still ongoing. It was a single-arm, multicenter, open-label, nonrandomized study to assess the efficacy and safety of HA121-28 in RET-fusion-positive NSCLC.

Patients aged 18–75 years (inclusive) who had unresectable locally advanced or metastatic NSCLC through histologically or cytologically examination were enrolled. Patients were also required to have documented RET fusions centrally examined by next-generation sequencing in certified laboratories but without other oncogenic alterations. Had progression on at least one previous line of standard treatment but have no previous use of other anti-RET inhibitors. The complete lists of inclusion and exclusion criteria of phase 1 and phase 2 are provided in both protocols (in Supplement).

Both trials were approved by the Independent Ethics Committee and conducted following the Declaration of Helsinki and Good Clinical Practices. Before enrolling patients, written informed consent was obtained. The phase 1 and phase 2 trials are registered with ClinicalTrials.gov: NCT03994484, NCT05117658, respectively.

#### Sequencing for tumor tissue

The sequencing system “Gene+Seq-2000” with a panel including 1021 genes (Geneplus-Beijing, Beijing, China) was used to sequence the genomic DNA from pre-treatment tumor tissue of 26 patients in the study. To identify the single nucleotide and copy number variations, short insertion and deletion, as well as gene fusions in the target genes. Briefly, a commercially available kit was used to extract DNA through formalin-fixed paraffin-embedded tissues, and the DNA concentration was measured. Using a commercial panel (Integrated DNA Technologies, Inc., Coralville, USA), 1021 genes of cancer-related were included to enrich tumor genomic and match germline DNA libraries through hybridization. This was followed by sequencing through a 100-bp paired-end configuration on a DNBSEQ-T7RS sequencer (MGI Tech, Shenzhen, China).^48^

### Procedures

In phase 1 trial, during the dose-escalation part, patients first administered HA121-28 in a single ascending dose under the fasted state, then followed up for seven days. Then, patients received ascending doses of HA121-28 (q.d.) on a 21-day on and 7-day off treatment scheme for a 28-day cycle. For patients deemed beneficial from the treatment by investigators, treatment could remain until disease progression, or they discontinued from the study. In the dose-expansion part of phase 1, as well as phase 2, patients received HA121-28 (q.d.) at the selected doses on a 21-day on and 7-day off treatment scheme until disease progression, or they discontinued from the study.

In the study, dose adjustment including dose reductions, interruptions, and permanent discontinuation were permitted based on the occurrence and severity of TRAEs. The maximum dose reduction was to 300 mg. Specific details on dose interruptions, reductions, and permanent discontinuation are provided in the protocol (in Supplement).

In the phase 1 trial, the patient-provided gene report was acceptable and needed to be reviewed and verified by investigators. PK samples were collected during the single dose stage, then the first 28-day dosing cycle of phase 1; the scheduled blood sampling time points are provided in the protocol (in Supplement).

Treatment-emergent adverse events (TEAEs) were documented during the entire study period. The predefined adverse event of special interest (AESI) was ≥ grade 3 prolonged QT interval. Tumor imaging was performed using contrast-enhanced computed tomography or magnetic resonance imaging at screening, Day 28 of the first cycle, then approximately every eight weeks till the end of the study for phase 1; in phase 2, imaging was scheduled at screening, and every eight weeks during the first 40 weeks, followed by every 12 weeks for the rest of the study period. RECIST 1.1 was used by the independent review committee or investigators for assessment of tumor response. After the original assessment, a repeated assessment was required around four weeks later to confirm a complete or partial response.

### Outcomes

The primary endpoints of phase 1 were to investigate the MTD, dose-limiting toxicities, and the incidence and severity of TEAEs. Each TEAE was graded according to the Common Terminology Criteria for Adverse Events (CTCAE, version 5.0) and coded by Medical Dictionary for Regulatory Activities (MedDRA, version 21.0). The secondary endpoints included calculating PK parameters, assessing the ORR and DCR at the recommended dose by investigators as per RECIST 1.1. The exploratory endpoint was PFS.

The primary endpoint of phase 2 was the independent review committee evaluated ORR using RECIST 1.1. The secondary endpoints included investigators assessed ORR; DCR, PFS, and DOR by the independent review committee and investigators; overall survival and TEAEs. Each TEAE was graded according to CTCAE version 5.0 and coded by MedDRA (version 24.0). The phase 2 trial is ongoing; not all endpoints are reported in this paper.

### Statistical analysis

The sample size of phase 1 was not calculated from power analysis; instead, it depended on the evaluated number of dose levels and cohorts’ number of dose-expansion. The maximum sample size for dose-expansion part was set to 150. For the phase 2 trial, the sample size of 83 pretreated NSCLC patients was estimated based on the assumption of 80% power, a significance test level of 0.025 (1-sided) to rule out a ≤ 30% ORR when the true ORR is 45%.^49^

The full analysis set consisted of all patients who had administered at least one dose of HA121-28 and was used to calculate the baseline characteristics, OS, and PFS results. The safety set included all patients who had administered at least one dose of the HA121-28 and had at least one safety-related observation. This population was used for analysis of TEAEs. The efficacy evaluable set consisted of patients who had administered at least one dose of HA121-28 and completed at least one efficacy assessment following study treatment. This population was used to calculate the ORR, DOR, and DCR. In addition, the ORRs for subgroup analyses, including age ( < 60, ≥60), ECOG PS (0 or 1), gender (male or female), prior treatment with TKIs (yes or no), brain or liver metastasis (yes or no), were also calculated. The plasma drug concentration-time profile was characterized using PK concentration set that included patients who had administered at least one dose of HA121-28 and had at least one measurable plasma concentration of HA121-28. The PK parameter set was used to calculate PK parameters, which consisted of patients who had administered at least one dose of HA121-28 with at least one valid PK parameter. ORR and DCR were described using the Clopper-Pearson method with two-sided 95% confidence intervals (CIs). PFS and DOR were assessed using the Kaplan-Meier method, including an estimate of the median, along with corresponding 95% CIs. Statistical analyses of clinical outcomes were conducted by SAS (version 9.4 or later) and R software 4.1.0 for genomic DNA from pre-treatment tumor tissue (http://cran.r-project.org).

## Supplementary information

HA121-28 Phase 1 study protocol

Supplementary materials

HA121-28 Phase 2 study protocol

## Data Availability

The datasets (including de-identified individual data) generated during the current study are available from the corresponding author upon reasonable request. Requestors will need to submit a proposal to the corresponding author and sign a data access agreement to gain the data access.
