# Supplementary material for: Safety, pharmacokinetics and efficacy of HA121-28 in patients with advanced solid tumors and RET fusion-positive non-small-cell lung cancer: a multicenter, open-label, single-arm phase 1/2 trial
Source: Signal Transduct Target Ther. 2025 Feb 28;10:62. doi: 10.1038/s41392-025-02155-5 (PMC11868595; doi:10.1038/s41392-025-02155-5)
Supplement: Supplementary file 1 — HA121-28 Phase 1 study protocol [file 41392_2025_2155_MOESM1_ESM.pdf]

Phase I Clinical Study of HA121-28 Tablets

Clinical Study Protocol

Approval Number:	2017L04800; 2017L04791; 2017L04790
Protocol Title:	A Phase I Clinical Study to Evaluate the Maximum Tolerated Dose and Pharmacokinetics of Single and Multiple-dose of HA121-28 Tablets in Patients with Advanced Solid Tumors
Protocol Number:	HA121-28/2017/01
Principal Testing Facility:	Sun Yat-sen University Cancer Center
	No. 651 Dongfeng East Road, Guangzhou
Principal Investigator:	Professor Xu Ruihua
Statistical Analysis Institution:	Beijing Improve-Quality Technology Co., Ltd.
Statistical Analysis Director:	Liu Yisong
Analytical Testing Institution:	HQ Bioscience Co., Ltd.
Protocol Design:	Zhao Hongyun, Chen Shan
Sponsor:	CSPC ZhongQi Pharmaceutical Technology (Shijiazhuang) Co., Ltd.
	No. 226 Huanghe Avenue, Shijiazhuang, Hebei Province
Sponsor Contact:	Xu Wen
Protocol Version:	V4.0
Version Date:	December 6, 2021

Confidentiality Statement

All information in this study protocol is confidential and owned by CSPC ZhongQi Pharmaceutical Technology (Shijiazhuang) Co., Ltd.

Protocol Signature Page

Phase I Clinical Study of HA121-28 Tablets
Statement and Signature

1. Sponsor Statement

I will be responsible for initiating, applying for, organizing, funding, and monitoring this clinical study in accordance with Good Clinical Practice (GCP) in China and drug registration regulations. I will provide treatment costs and financial compensation to patients who suffer from study-related harm or death during the clinical study and provide legal and financial guarantees to investigators. CSPC ZhongQi agrees to conduct the clinical study according to the protocol (Protocol number: HA121-28/2017/01, Version: V4.0, Version date: December 06, 2021).

Sponsor: CSPC ZhongQi Pharmaceutical Technology (Shijiazhuang) Co., Ltd.

Signature of Project Director:

Date: MM-DD-YYYY

Protocol Signature Page

Phase I Clinical Study of HA121-28 Tablets
Statement and Signature

2. Statement of Testing Facility

I will strictly conduct this clinical study in accordance with the ethical, moral, and scientific principles outlined in the Helsinki Declaration and GCP regulations and in strict accordance with the design and regulations of this protocol (Protocol number: HA121-28/2017/01, Version: V4.0, Version date: December 06, 2021). I will also maintain the confidentiality of this protocol and related information.

Testing Facility:

Signature of Principal Investigator:

Date: MM-DD-YYYY

Protocol Signature Page

Phase I Clinical Study of HA121-28 Tablets
Statement and Signature

3. Statement of Statistical Institution

I have reviewed and agreed to abide by the protocol (Protocol ID: HA121-28/2017/01, Version: V4.0, Version Date: December 06, 2021) to conduct the study.

Statistical Institution: Beijing Improve-Quality Technology Co., Ltd.

Signature of Project Director:

Date: MM-DD-YYYY

Table of Contents

Protocol Synopsis 7
Abbreviations 16
1. Study Background 17
1.1. Drug Name 17
1.2. Chemical Structural Formula, Molecular Formula, Molecular Weight, Basic Physicochemical Properties 17
1.3. Pharmacological Action and Mechanism of Action 17
1.3.1. Pharmacodynamic Studies 17
1.3.2. Pharmacokinetic Studies 19
1.3.3. Toxicology Studies 21
2. Study Objectives 22
2.1. Dose Escalation Phase 22
2.2. Dose expansion phase 22
3. Overall Study Design 22
3.1. Dose Escalation Phase 23
3.2. Dose Expansion Phase 23
4. Investigational Product 23
5. Study Population 24
5.1. Inclusion Criteria 24
5.2. Exclusion Criteria 25
5.3. Discontinuation criteria: 26
5.4. Withdrawal Criteria: 26
5.5. Early Discontinuation/Withdrawal Post-treatment 26
6. Design of Dose Escalation Phase 26
6.1. Estimation of Maximum Safe Starting Dose 26
6.2. Dose Escalation Scheme 27
6.3. Determination of Maximum Escalation Dose 28
6.4. Design of Dose Escalation 28
6.4.1. Patient Replacement 29
6.4.2. Treatment of Missed Dose 29
6.5. Determination of DLT and MTD 29
6.6. Single and multiple dose pharmacokinetics 29
7. Design of Dose Expansion Phase 30
7.1. Dose Selection 30
7.2. Rationale for Dose Selection 30
8. Pharmacokinetic Study Design 30
8.1. Method of Administration 30
8.2. Blood Sampling (Dose Escalation Phase) 31
8.2.1. Single-dose Pharmacokinetic Study (Cycle 0) 31
8.2.2. Multiple-dose Tolerability and Pharmacokinetic Study (Cycle 1) 31
8.3. Blood Sampling (Dose Expansion Phase) 32
8.4. Biomarkers 33
9. Study Procedures 33
9.1. Screening: 33
9.2. Single-dose Period (Cycle 0): applicable only for the dose escalation phase 34
9.3. Multiple-dose Period (Cycle 1): 34
9.4. Subsequent Treatment Period: 35
9.5. End of Treatment: 35
9.6. Follow-up 36

10. Concomitant Medications 36
10.1. Drugs Prohibited or Used with Caution 36
10.2. Medications that May Be Used as Appropriate in the Study 36
10.2.1. During Cycle 1 of Continuous Dosing 36
10.2.2. During and after Continuous Dosing 37
11. Safety and tolerability evaluation 38
11.1. Potential toxicity risk 38
11.2. Safety Monitoring Measures 38
11.3. Safety Evaluation Rules 40
11.4. Efficacy Evaluation 47
12. Data Review and Database Management 48
12.1. Construction of Electronic Case Report Form 48
12.2. Permission Assignment 48
12.3. Data Entry 48
12.4. Data Cleaning 49
12.5. On-site Verification of Source Data 49
12.6. Data Locking and Export 49
13. Statistical Analysis 49
13.1. Analysis Sets 49
13.2. Data Analysis Content 49
13.2.1. Case Enrollment Analysis 49
13.2.2. Demographic Data and Baseline Analysis 49
13.2.3. Safety Analysis 50
13.2.4. Pharmacokinetic Analysis 50
13.2.5. Efficacy Analysis 50
13.3. Interim Analysis 51
14. Management Procedures 51
14.1. Protocol Compliance 51
14.2. Protocol Amendment 51
14.3. Administrative Revisions 52
14.4. Management of Investigational Product 52
14.5. Sample Collection Management 52
15. Quality Control and Assurance of the Study 52
15.1. Ward and Laboratory Quality Control 52
15.2. Qualifications of Testing Facility and Study Personnel 53
15.3. Investigator Training 53
15.4. Monitoring of Clinical Studies 53
16. Ethical Requirements 53
17. Study Summary Report 54
18. Data Storage 54
19. Publication of Study Results 55
20. Reference Regulation 55
Appendix I Schedule of Assessment 56
Appendix II ECOG performance score 60
Appendix III Response Evaluation Criteria in Solid Tumors 61

Protocol Synopsis

**Protocol Title**	A Phase I Clinical Study to Evaluate the Maximum Tolerated Dose and Pharmacokinetics of Single and Multiple-dose of HA121-28 Tablets in Patients with Advanced Solid Tumors
**Protocol Number**	HA121-28/2017/01
**Protocol Version Number and Version Date**	V4.0/06-DEC-2021
**Sponsor**	CSPC ZhongQi Pharmaceutical Technology (Shijiazhuang) Co., Ltd.
**Leading Unit**	Sun Yat-sen University Cancer Center
**Principal Investigator**	Professor Xu Ruihua
**Investigational Product**	Dosage Form: Tablet<br>Strength: 25 mg, 100 mg, 200 mg
**Study Design**	Phase I clinical study with single-arm, open-label, single and multiple doses, dose escalation, and dose expansion.
**Study Objectives**	Dose Escalation Phase<br>Primary Objectives:<br>• To observe the safety of HA121-28 tablets in humans, determine the dose-limiting toxicity (DLT) and maximum tolerated dose (MTD) of HA121-28 tablets in humans, and recommend a safe dose for the dose expansion phase.<br>• To preliminarily investigate the pharmacokinetic (PK) characteristics of HA121-28 tablets after single and multiple doses.<br>• To investigate the safety and tolerability of HA121-28 tablets.<br>Secondary Objective:<br>• To preliminarily observe the efficacy of HA121-28 tablets.<br>Dose Expansion Phase<br>Primary Objectives:<br>• To investigate the PK characteristics of multiple doses of HA121-28 tablets and whether there is accumulation in the human body after multiple doses.<br>• To investigate the safety and tolerability of HA121-28 tablets.<br>• To determine the recommended dose for subsequent clinical studies.<br>Secondary Objective:

- To investigate the efficacy of HA121-28 tablets in patients with advanced solid tumors.

Study Design

Dose Escalation Phase:

The study will start with a dose of 25 mg and use a 3+3 study design. PK assessments and DLT event observations will be conducted during the initial single and multiple dosing in cycle 0 and cycle 1. After the completion of cycle 1, patients who can continue treatment with the study treatment will receive it every 28 days until disease progression or intolerable toxicity occurs, as determined by the investigator. Efficacy assessments will be conducted every two cycles.

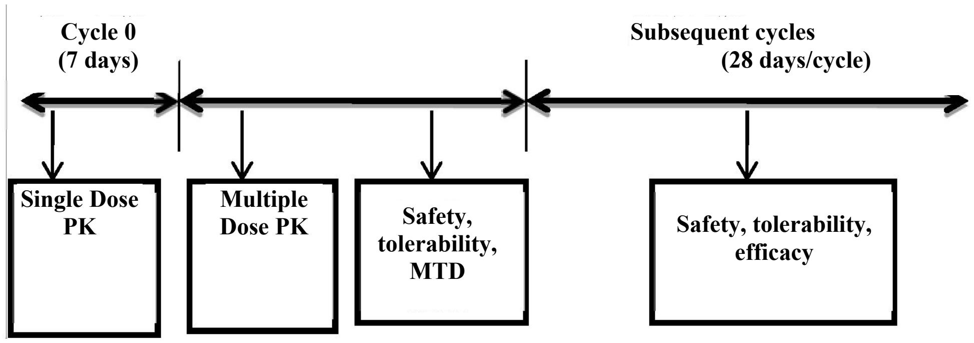

Dose expansion phase:

The maximum tolerated dose (or 800 mg) group obtained during the dose escalation phase and the maximum tolerated dose below groups determined by the investigator in consultation with the sponsor will be conducted for a multicenter, open-label extension study. This study plans to enroll a maximum of 150 patients with advanced solid tumors to evaluate the efficacy, safety, and PK characteristics of HA121-28 tablets in patients with advanced solid tumors. Eligible patients will be directly enrolled in the first dosing cycle, receiving continuous treatment for 21 days, followed by a 7-day rest period, with a 28-day dosing cycle until disease progression, intolerable toxicity, withdrawal of informed consent, pregnancy, or the initiation of new anti-tumor therapy, whichever occurs first.

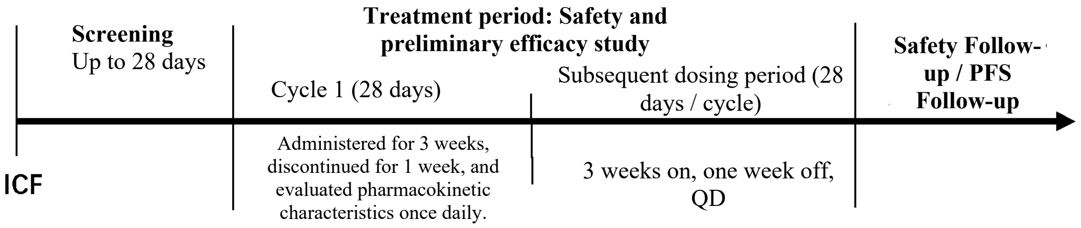

Method of administration

Oral administration with warm water.

Patients are administered under a fasting condition in the morning. If a patient experiences significant gastrointestinal adverse reactions due to fasting, administration after a meal is allowed, and the timing of the meal and drug administration should be recorded.

Single dose: Cycle 0 Day 1.

Multiple doses: Cycle 1 dosing starts from 7 days after administration of study treatment on Day 1 of the cycle once daily for 21 consecutive days, followed by a 7-day rest period. Each cycle lasts 28 days, and the administration time should be kept as consistent as possible. Subsequent administrations: After the blood collection for multiple doses is completed, administration begins once daily for 21 consecutive days, followed by a 7-day rest period. Each cycle lasts 28 days.

Dose Escalation Phase

1. Estimation of Maximum Safe Starting Dose

For non-cytotoxic anti-tumor drugs, the calculation of the initial dose for a single administration is based on the No Observed Adverse Effect Level (NOAEL) recommended by the FDA for animal studies to estimate the Human Equivalent Dose (HED). According to the “Estimating the Maximum Safe Starting Dose in Initial Clinical Trials for Therapeutics in Adult Healthy Volunteers,” select toxicology-related varieties or doses of the most sensitive animals with no adverse reactions, and estimate the maximum recommended initial amount for the first human trial of the new drug. The specific calculation process is as follows:

a. To determine the NOAEL for long-term toxicity testing of experimental animals, this study will use the dose that causes mild lesions in SD rats after 28 days of medication and is recoverable after discontinuation.

b. Calculate the human equivalent dose based on the coefficients of the human equivalent dose scale.

c. The safety factor is set to 5, according to the Guidelines for Clinical Study of Antitumor Drugs". For some non-cytotoxic antitumor drugs, due to their relatively low toxicity, the starting dose calculation for Phase I clinical study can use 1/5 of the non-rodent animal NOAEL in non-clinical studies.

d. Calculate the maximum recommended starting dose based on body weight. Please refer to the table below:

Table 1 Conversion of Maximum Starting Dose

	28-day Sprague-Dawley rats	Animal Species	28 d Beagle dog	
NOAELs	15 mg/kg*		12 mg/kg*	
HED	$15\times(0.2/60)^{0.33}$ =2.28 mg/kg	15×0.16 =2.4 mg/kg	$12\times(10/60)^{0.33}$ =6.64 mg/kg	12×0.54 =6.48 mg/kg
Safety factor, 5	0.456 mg/kg	0.48 mg/kg	1.328 mg/kg	1.296 mg/kg
Maximum recommended starting dose (BW: 60 kg)	27.36 mg	28.8 mg	79.68 mg	77.76 mg
Select the most appropriate/sensitive species		√		

NOAEL: No-observed-adverse-effect level

HED: Human equivalent dose

BW: Body weight

* The dose at which mild reversible lesions occur in rats and dogs and which can be recovered after discontinuation of the drug.

2. Determination of Maximum Escalating Dose

Based on the Phase I clinical study results of the positive reference vandetanib, the MTD is 500 mg/d. Taking into account the animal PK results, the C_{max} and AUC of HA121-28 at the same dose are about half of those of vandetanib. Considering 500 mg/d*2=1000 mg/d, the maximum dose for this study is tentatively set as 800 mg/d.

3. Dose Escalation Design

1) According to the above calculation, the dose escalation will start from 25 mg, and considering the marketed dose of 300 mg for a similar tyrosine kinase inhibitor vandetanib, the number of patients at low doses will be minimized. One patient will be enrolled in each of the 25 mg and 50 mg dose groups. Subsequently, three patients will be enrolled in each dose group. If no DLT occurs in the three patients, the study will proceed to the next dose group. If one patient experiences DLT, three additional patients will be enrolled in that dose group. If no DLT occurs in the additional three patients, the study will proceed to the next dose group. If one or more DLTs occur in the additional three patients, the dose escalation will stop. If two patients experience DLT in a dose group, the dose escalation will stop, and the previous dose will be considered as the MTD.

2) The observation period for DLT includes cycle 0 of single-dose administration and the first treatment cycle of multiple-dose administration. Patients who continue treatment will undergo efficacy evaluation every two cycles. Although adverse reactions that occur in the second and subsequent cycles are not used as the basis for judging DLT, if they are severe, they will be discussed again.

3) Escalation to the next dose group after the last patient in each group has completed the observation for multiple-dose administration of cycle 1.

4) During the dose escalation phase, with the joint decision of the investigator and the sponsor, expansion can be carried out in the dose group that has completed the tolerability evaluation, but only for solid tumor patients with RET and FGFR gene alterations and no more than 6 cases.

4. Dose Escalation Scheme

The dosage of this study starts at 25mg/day/person and increases according to the modified Fibonacci method with a ratio of 100%, 100%, 100%, 50%, 50%, 33%, 33%... The starting dose is 25mg/day, with the aim of exposing the minimum number of patients to ineffective doses. Only one patient is enrolled in each of the 25mg/day and 50mg/day dose groups, while three patients are enrolled in the 100mg dose group to observe the safety and tolerability of the patients. Each patient receives only one corresponding dose, and the study starts from the lowest dose. The next dose group can only begin after the tolerability observation of all three patients in the previous dose group is completed. See the table below for grouping.

Table 2 Dose Escalation Scheme

Grouping	Dose Escalation Proportion	Number	Dose (mg/day/person)	Dosing regimen	Study Contents

1		1	25	25 mg*1	PK, tolerability
2	100%	1	50	25 mg*2	PK, tolerability
3	100%	3	100	100 mg*1	PK, tolerability
4	100%	3	200	200 mg*1	PK, tolerability
5	50%	3	300	200 mg*1+100 mg*1	PK, tolerability
6	50%	3	450	200 mg*2+25 mg*2	PK, tolerability
7	33%	3	600	200 mg*3	PK, tolerability
8	33%	3	800	200 mg*4	PK, tolerability

If grade 2 or higher toxicity related to the study drug occurs in Groups 1 and 2 (as determined by the investigator), dose escalation will proceed using a 3+3 design starting from that dose level. If no DLT is observed at the 800 mg/d dose level after dose escalation, a meeting of investigators will be convened to decide whether to proceed with further dose escalation based on the available PK data, safety, and tolerability information.

5. Definition of DLT and MTD

DLT Definition:

The following events related to the study drug (including those that are definitely related, highly likely to be related, and possibly related):

(1) Grade 4 hematologic toxicity or grade 3 neutropenia accompanied by a fever of 38.5℃ or higher, or grade 3 thrombocytopenia accompanied by severe bleeding.

(2) Patients who discontinued the medication for more than 7 days due to unresolved toxicity reactions or who discontinued the medication more than twice due to toxicity reactions.

(3) In the context of supportive treatment, diarrhea of grade 2 lasting more than 7 days, or grade 3 diarrhea lasting more than 3 days.

(4) QTc interval prolongation: a single QTc interval ≥550 ms or an increase from baseline of ≥100 ms; or two consecutive QTc intervals ≥500 ms within 48 hours or an increase from baseline of ≥60 ms but <100ms and the QTc interval value is ≥480ms.

(5) Grade 3 or above skin toxicity;

(6) Other grade 3 or higher non-hematologic toxicities (according to CTCAE V4.03 criteria).

MTD definition: The maximum tolerated dose (MTD) is defined as the dose level below the one at which dose-limiting toxicity (DLT) occurs.

6. Pharmacokinetics after Single and Multiple Doses

Two patients from the 25 mg/d and 50 mg/d dose groups will be selected for pilot single and multiple-dose PK studies. PK parameters obtained from the pilot study results will be used to adjust the PK study and blood sampling time point design for subsequent studies.

For detailed information on blood sample collection, processing, storage, transportation, and testing methods, please refer to the relevant SOP.

1. Dose Selection in Dose Expansion Phase

After reaching the maximum tolerated dose during the dose escalation phase, it is recommended to use a dose below the maximum tolerated dose, which is determined through discussion between the investigator and the sponsor, as the maximum recommended starting dose for the expansion phase. The drug will be administered continuously for 21 days, followed by a 7-day rest period, constituting a 28-day dosing cycle. Dose adjustments will be made during the study based on the patient's tolerance, as detailed in section 10.2 on the drugs

that may be used at the investigator's discretion during the study.

If the maximum tolerated dose is not reached during the dose escalation phase, a dose of 800mg or a dose below 800mg determined by the investigator and sponsor together will be recommended as the maximum starting dose for patients in the expansion phase.

If a patient misses a dose during the study, the same principles for dose escalation should be followed.

2. Rationale for Dose Selection

According to the results of preclinical studies, the anti-tumor efficacy of the drug increases with increasing doses, and the maximum inhibition of the target can be achieved at the maximum tolerated dose in clinical studies, resulting in the expected clinical anti-tumor efficacy. In addition, during the dose escalation phase of Phase I of this study, the selected dose has been preliminarily proven to be tolerable in patients with solid tumors. In order to further fully evaluate the safety and PK characteristics of HA121-28, and to preliminarily evaluate the drug's anti-tumor activity, the investigator and sponsor jointly decided to conduct an appropriate expansion in the dose group below the maximum tolerated dose (or 800mg).

Inclusion Criteria

1) Be willing to participate in the clinical trial and sign the informed consent;
2) Men and women aged 18 to 75 years;
3) Histologically/cytologically confirmed advanced/metastatic solid tumor, and have failed prior standard of care or for which no standard of care is durable (patients with RET fusion/mutation can be included regardless of whether they have received standard of care or not);
4) At least one measurable lesion according to RECIST 1.1;
5) Patients have not received any anti-cancer therapies, including chemotherapy, radiotherapy, targeted treatment, and surgery, within 4 weeks prior to participation. The washout period for traditional Chinese medicine or Chinese patent medicine with anti-tumor indications is 2 weeks. The washout period for local palliative radiotherapy for relieving bone metastasis pain is 2 weeks;
6) Eastern Cooperative Oncology Group (ECOG) Performance Status of 0~1;
7) Expected overall survival (Life expectancy) ≥ 3 months;
8) Laboratory test results must meet the following standards:

Absolute neutrophil count (ANC) ≥1.5× 10^9/L; Platelet count (PLT) ≥75×10^9/L; Hemoglobin (Hb) ≥90 g/L (no blood transfusion within 14 days); Alanine aminotransferase (ALT) and aspartate aminotransferase (AST) ≤2.5× upper limit of normal (ULN) (in patients with liver metastasis ≤5.0× ULN); Total bilirubin ≤ 1.5× ULN; Serum creatinine≤ 1.5× ULN/

9) Male and female patients of childbearing potential should agree to use suitable methods of contraception during the treatment and 6 months after the last dose of study medication; female patients should have negative results of serum/urine pregnancy test within 7 days prior to enrollment and cannot be breastfeeding.

Exclusion Criteria

(1) Has participated in other clinical trials and received the treatment within 4 weeks prior to enrollment;

(2) Patients who cannot swallow or have chronic diarrhea and intestinal obstruction, which may affect the administration and absorption of the drug;

(3) Patients who meet one of the following criteria:

- Corrected QT (QTc) ≥470ms in women, ≥450ms in men; or congenital long QT syndrome (LQTS), taking QT-prolonging medications, and has a family history of long QT syndrome;
- Resting ECG result shows clinically significant abnormalities of rhythm, conduction or morphology, requiring therapeutic intervention;

(4) Urinalysis result shows protein in urine ≥ ++ and 24-hour urine protein > 1.0 g;

(5) Based on the investigator's assessment, patients with known severe comorbidities which may influence the safety of the patients and the study completion [such as uncontrolled hypertension (systolic pressure ≥150 mmHg or diastolic pressure ≥100 mmHg, despite being treated with optimal medicine), diabetes, etc.];

(6) Patients who have symptoms of metastatic brain/meningeal tumors within 4 weeks of participation, unless the investigator determines that the clinical symptoms are stable and the patient is suitable for enrollment;

(7) Ongoing adverse events (AEs) >grade 1 at the time of participation (except hair loss and pigmentation);

(8) Patients who have undergone major surgery or have not recovered from Invasive operation within 4 weeks prior to initiation of study treatment;

(9) Coagulation disorders (INR >1.5, prothrombin time (PT) > ULN+4s or APTT >1.5 ULN): with bleeding diathesis (such as an active peptic ulcer) or receiving thrombolytic or anti-coagulant treatment;

(10) Known pulmonary infection/ pneumonitis/interstitial pneumonia who are not suitable for the study;

(11) Known active Hepatitis B or Hepatitis C virus infection;

- if the HBsAg result is positive, additional HBV DNA testing is required (the result is higher than the ULN of the study site);
- if the HCV antibody result is positive, additional HCV RNA testing is required (the result is higher than the ULN of the research center);

(12) Known history of human immunodeficiency virus (HIV) or other acquired/congenital immune deficiency diseases or organ transplantation;<br>(13) Other anti-tumor therapies are required (including radiotherapy, chemotherapy, immunotherapy, targeted treatment, traditional Chinese medicine, etc.);<br>(14) Patients with a known history of neurological or psychiatric disorders, including epilepsy or dementia;<br>(15) Not suitable for the treatment assessed by the investigators;<br>(16) Cardiac ejection fraction less than 50%;<br>(17) Patients who have suffered from or are complicated with any other malignant tumor within 5 years (except radically resected skin basal cell carcinoma, skin squamous cell carcinoma, superficial bladder cancer, local prostate cancer, in situ cervical cancer or other carcinoma in situ)
**Pharmacokinetics**<br>Single- and multiple- dose PK<br>Calculate the PK parameters for each patient, including C_{max}, AUC_{0-24}, AUC_{0-t}, AUC_{0-inf}, T_{max}, V_z/F, $t_{1/2z}$, CL_z/F, and $\%AUC_{ext}$ after the first dose (single dose) of the drug, as well as C_{max}, $AUC_{0-\tau}$, AUC_{0-t}, AUC_{0-inf}, T_{max}, V/F, $t_{1/2z}$, CL_z/F, and $\%AUC_{ext}$ after multiple-doses at steady state. Calculate the accumulation index $R1_{ac}=C_{max}$, (D21)/C_{max} (after the first dose), and $R2_{ac}=AUC_{0-\tau}/AUC_{0-24}$ (after the first dose). Additionally, calculate the mean and standard deviation for each parameter.
**Evaluation Measures**<br>Safety evaluation measures<br>Safety evaluation includes monitoring and recording all AEs and serious adverse events (SAEs), regular monitoring of blood routine, blood biochemistry, urine routine, electrocardiogram, vital signs, and physical examination, etc.<br>Efficacy evaluation measures<br>Evaluate the efficacy of the patients according to RECIST 1.1 criteria.<br>Primary efficacy measure: Objective response rate (ORR)<br>Secondary efficacy measures: progression-free survival (PFS) and disease control rate (DCR).
**Biomarkers**<br>This study investigates the use of RET, FGFR, EGFR, and VEGFR as biomarkers. Prior to treatment initiation, pathological reports of all enrolled patients are collected to record the gene expression and mutation status of RET, FGFR, EGFR/VEGFR (information is collected based on the actual situation of the patients, and further testing is not conducted for patients without corresponding tests).

The collected information will only be used for subsequent preliminary exploration of the correlation between biomarkers and drug efficacy, and will not affect patient enrollment.
**End of Study**<br>The end of the dose escalation phase study is defined as the completion of DLT observation for the last enrolled patient. However, patients who are eligible for subsequent long-term treatment can be treated for up to one year at the discretion of the investigator, with the possibility of an extension for more than one year with the agreement of the sponsor. The end of the dose expansion phase study is defined as the observation of survival data for the last patient used for statistical analysis. Termination of the study by the sponsor for any reason during the study period is also considered the end of the study.
**Planned Study Period**<br>April 2018-December 2022

Abbreviations

Abbreviation	Definition	Abbreviation	Definition
PTA%	Plasma prothrombin activity	HNSTD	Highest No-Serious Adverse Effect Level
AE	Adverse Events	IC50	Half inhibitory concentration
ALT	Alanine aminotransferase	IRB	Ethics Committee
ANC	Neutrophil count	MTD	Maximum tolerated dose
APTT	Activated partial prothrombin time	NCI-CTCAE	National Cancer Institute Common Terminology Criteria for Adverse Events
AST	Aspartate aminotransferase	NOAEL	No-observed-adverse-effect level
AUC	Area under the plasma concentration-time curve	ORR	Objective response rate
CLz/F	Clearance	OS	Overall survival
Cmax	Peak plasma concentration	PD	Progressive disease
CR	Complete response	PFS	Progression-free survival
CRF	Case Report Form	PK	Pharmacokinetics
CT	Computed tomography	PLT	Platelet count
DCR	Disease control rate	PR	Partial response
DLT	Dose-limiting toxicity	PT	Plasma prothrombin time
ECG	Electrocardiogram	QTc	Correction of QT interval by Fridericia's method
ECOG	Eastern Cooperative Oncology Group	Rauc	Accumulation index
eCRF	Electronic Case Report Form	RECIST1.1	Response Evaluation Criteria in Solid Tumors Version 1.1
EDC	Data Management System	RP2D	Recommended Phase 2 Dose
EGFR	Epidermal growth factor receptor	SAE	Serious Adverse Events
EGJ	Esophageal or gastroesophageal junction	SCC	Squamous cell carcinoma
FAS	Full Analysis Set	SD	Stable disease
Fbg	Fibrinogen	SOP	Standard Operating Procedure
FT3	Free triiodothyronine	SS	Safety Dataset
FT4	Free thyroxine	Tmax	Time to peak
GCP	Good Clinical Practice	TSH	Thyroid-stimulating hormone
Hb	Hemoglobin	TT	Thrombin time
HBV	Hepatitis B virus	TT3	Total triiodothyronine
HCV	Hepatitis C virus	TT4	Total thyroxine
HED	Human equivalent dose	VEGFR	Vascular endothelial growth factor receptor
HIV	Human immunodeficiency virus	V_z/F	Apparent volume of distribution

1. Study Background

1.1. Drug Name

Generic Name: Not yet available

English Name: Not yet available

Chinese Pinyin Name: Not yet available

Laboratory Code: HA121-28

[redacted]

1.2. Chemical Structural Formula, Molecular Formula, Molecular Weight, Basic Physicochemical Properties

[redacted]

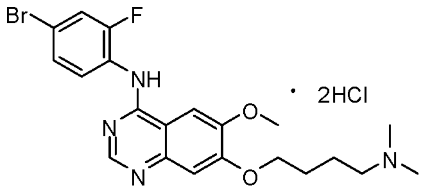

[redacted]

1.3. Pharmacological Action and Mechanism of Action

1.3.1. Pharmacodynamic Studies

HA121-28 is a multi-target tyrosine kinase inhibitor. At the molecular level, it has strong inhibitory effects on the tyrosine kinase activity of proteins such as RET, KDR, EGFR, FGFR1-3, FLT-1, HER-2, LCK, EphA1, and SRC, [redacted].

The IC_{50} values of HA121-28 for inhibiting CFPAC-1, A498, A375, 8305C, and A431 cell lines *in vitro* are between 0.1-1 μM, with values of 207.2±38.33, 858.5±17.88, 715.59±153.65, 623.05±80.26, and 774.87±76.74 nmol/L, respectively. The inhibitory effect on CFPAC-1 is the most significant. The IC_{50} values of HA121-28 for inhibiting ECA-109, HCT116, TT, HT29, SGC-7901, and KYSE-150 cell lines *in vitro* are between 1-10 μM, with values of 6662.8±54.45, 1419.75±235.82, 3825.9±91.5, 5397.3±813.03, 4364.65±338.07, and 1083.7±220.05 nmol/L, respectively. The IC_{50} values of HA121-28 for inhibiting BEL-7402 and MDA-MB-231 cell lines *in vitro* are between 10-100 μM. The metabolite M4 has a significant inhibitory effect on the proliferation of KYSE-150 cells *in vitro*, with an IC_{50} of 1428.0 nM, slightly lower than that of HA121-28, which is 1083.7 nM. The inhibitory effect of M5, M8, and M9 on KYSE-150 cells is not significant, with IC_{50} values greater than 10μM.

In an *in vitro* model of human skin squamous cell carcinoma A431 cells stimulated with 10 ng/mL EGF, both HA121-28 and vandetanib significantly inhibited the activation of EGFR and its downstream signaling protein ERK induced by EGF stimulation, with a concentration-dependent inhibitory effect, and the activity of HA121-28 was higher than that of vandetanib. At all time points, the expression of EGFR and ERK was not significantly affected. In an in vitro model of human HUVEC cells stimulated with 50 ng/mL VEGF165, both HA121-28 and vandetanib significantly inhibited the activation of VEGFR and its downstream signaling protein ERK, with a concentration-dependent inhibitory effect, and the activity of HA121-28 was higher than that of vandetanib. At all time points, the expression of VEGFR and ERK was not significantly affected. In an in vitro model of human thyroid cancer TT cells, both HA121-28 and vandetanib significantly inhibited the activation of RET and its downstream signaling protein ERK, with a concentration-dependent inhibitory effect, and the activity of HA121-28 was higher than that of vandetanib. At all time points, the expression of RET and ERK was not significantly affected. In an *in vitro* model of human HUVEC cells stimulated with 50 ng/mL VEGF165, both HA121-28 and vandetanib significantly inhibited the proliferation of HUVEC cells stimulated by VEGF165, with a concentration-dependent inhibitory effect, and the IC_{50} values were 65.44 and 117.21 nM, respectively. HA121-28 was able to inhibit rat arterial ring angiogenesis and mouse matrigel angiogenesis induced by VEGF and FGF, and its inhibitory activity was equivalent to or better than that of vandetanib, indicating that HA121-28 had anti-angiogenic effects.

HA121-28 has *in vivo* anti-tumor effects on human esophageal cancer ECA109 and KYSE-150, human thyroid cancer TT, human melanoma A375, and human gastric cancer SGC7901 cell lines. In the KYSE-150 and TT models of human esophageal and thyroid cancer, respectively, tumor shrinkage was observed at a dose of 50 mg/kg. HA121-28 had a broad-spectrum anti-tumor effect and could cause partial tumor shrinkage in all the above-mentioned tumor models in nude mice. Vandetanib also had good efficacy in the above tumor models, but its efficacy was weaker than that of HA121-28. After oral administration to mice, HA121-28 showed a linear PK profile in both plasma and tumor tissues within the dose range of 12.5-50 mg/kg. The pharmacodynamic experiment results showed a clear dose-effect relationship between the pharmacodynamic index of tumor-bearing mice and the dose of HA121-28 within the dose range of 12.5-50 mg/kg. The drug concentration of HA121-28 in tumor tissues was higher than that in plasma, with a tumor tissue AUC_{0-72} of 4-6 times that of plasma AUC_{0-72}. The elimination of HA121-28 in tumor tissues was significantly slower than that in plasma, with an MRT of 17-19h, which was conducive to the drug's action at the tumor site. HA121-28 at doses of 25 and 50 mg/kg had inhibitory effects on the activity of EGFR and the downstream signaling protein ERK. The inhibitory effect of HA121-28 on the EGFR signaling pathway began to appear 2 hours after a single dose and lasted for 72 h (EGFR) and 24 h (ERK), respectively. Therefore, the level of kinase inhibition in tumor tissues was positively correlated with the tissue drug concentration.

In summary, HA121-28 is a multi-target protein kinase inhibitor. At the molecular level, it has strong inhibitory effects on the tyrosine kinase activity of RET, KDR, EGFR, FGFR1-3, FLT-1, HER-2, LCK, EphA1, SRC. It has stronger inhibitory activity on multiple kinases than vandetanib. HA121-28 has good inhibitory effects on various tumor cell lines. It can inhibit the signaling pathways of EGFR, VEGFR, and RET at the cellular level. HA121-28 can inhibit angiogenesis. It has significant therapeutic effects on various human tumor xenografts in nude mice. In conclusion, HA121-28 has demonstrated strong in vivo and in vitro anti-tumor effects, with activity superior to vandetanib. Therefore, preclinical data supports further clinical investigation of HA121-28.

1.3.2. Pharmacokinetic Studies

1.3.2.1. Absorption

Rat plasma kinetics

Parameter	Unit	Low dose (5 mg/kg)	Medium dose (10 mg/kg)	High dose (20 mg/kg)	Intravenous administration (5 mg/kg)	7 consecutive days (10 mg/kg)
C_{max}/C_{5min}	ng/mL	58.7±12.7	147±26.6	288±38.5	246±43.1	284±56.8
T_{max}	h	5.00±1.10	4.50±1.22	5.00±1.55	/	6.00±0.00
$AUC_{0\text{-}96h}$	h·ng/mL	1350±419	3200±629	6460±849	1440±214	8420±1720
$AUC_{0\text{-}\infty}$	h·ng/mL	1390±438	3300±696	6760±1040	1470±232	9690±2310
$MRT_{0\text{-}96h}$	h	19.2±2.41	21.8±2.80	22.8±2.85	17.2±1.68	/
$MRT_{0\text{-}\infty}$	h	21.5±3.23	24.7±4.71	27.3±5.24	19.0±2.82	40.0±7.30
V_d	L/kg	93.5±15.9	82.8±9.31	96.8±8.19	90.9±12.3	110±24.9
CL	L/h/kg	3.91±1.19	3.15±0.695	3.02±0.483	3.47±0.528	2.36±0.392
$t_{1/2}$	h	17.4±3.52	18.8±3.60	22.8±5.00	18.5±3.86	32.9±7.77
F	%	93.8				

Dog plasma kinetics

Parameter	Unit	Low dose (2.5 mg/kg)	Medium dose (5 mg/kg)	High dose (10 mg/kg)	Intravenous administration (2.5 mg/kg)	7 consecutive days (5 mg/kg)
C_{max}/C_{5min}	ng/mL	13.4±2.71	24.8±3.90	58.9±26.7	124±17.6	30.6±3.25
T_{max}	h	6.50±3.51	2.17±0.753	2.17±0.753	/	6.17±2.48
$AUC_{0\text{-}72h}$	h·ng/mL	274±68.2	393±74.0	837±207	489±73.0	639±69.0

$AUC_{0-\infty}$	h·ng/mL	279±70.5	398±75.3	848±212	493±73.7	655±72.2
MRT_{0-72h}	h	16.3±2.03	14.3±1.36	14.8±1.96	10.9±1.30	/
$MRT_{0-\infty}$	h	17.6±2.45	15.2±1.57	15.7±2.45	11.5±1.51	18.5±1.89
V_d	L/kg	160±41.3	217±53.6	197±57.5	85.0±27.0	214±25.1
CL	L/h/kg	9.41±2.24	13.0±2.57	12.7±4.51	5.18±0.889	10.5±1.29
$t_{1/2}$	h	11.8±1.00	11.5±1.16	11.0±1.51	11.2±1.70	14.2±1.19
F	%	56.0				

Cynomolgus monkey plasma kinetics

Parameter	Unit	Administration by gavage (5 mg/kg)	Intravenous (5 mg/kg)
C_{max}/C_{6min}	ng/mL	75.9±27.8	314±61.4
T_{max}	h	6.75±1.50	
AUC_{0-72h}	h·ng/mL	1680±766	2610±1340
$AUC_{0-\infty}$	h·ng/mL	1920±992	3000±1880
MRT_{0-72h}	h	20.9±2.67	18.8±3.77
$MRT_{0-\infty}$	h	30.5±8.00	27.5±11.4
V_d	L/kg	100±24.1	58.7±15.7
CL	L/h/kg	3.05±1.22	2.05±0.816
$t_{1/2}$	h	24.4±5.68	22.3±8.39
F	%	64.4	

Summary

HA121-28 was orally administered to rats, dogs, and monkeys and showed a moderate absorption rate and good systemic exposure. The C_{max} and $AUC_{0-96\,h}$ were positively correlated and proportional to the dose. The parameters such as V_d, CL, and $t_{1/2}$ did not change significantly with increasing doses, showing linear PK characteristics. The oral bioavailability in rats, dogs, and monkeys was 93.8%, 56.0%, and 64.4%, respectively.

HA121-28 has a high binding rate with plasma proteins in rats, mice, dogs, monkeys, and humans. The order of plasma protein binding rate from high to low is: mice (97.2±0.266%) > rats (92.4±1.28%) ≈ dogs (91.9±0.857%) ≈ monkeys (92.5±0.331%) ≈ humans (90.1±1.05%), and there is no concentration dependence.

1.3.2.2. Distribution

Tissue distribution studies in rats showed that HA121-28 was widely distributed in tissues, and the parent drug could be detected in all tissues studied. The AUC_{0-72h} exposure levels in various tissues were higher than those in plasma, with the spleen, lungs, and adrenal glands having the highest levels (≈200 times plasma) followed by the liver, kidneys, and ovaries (≈100 times plasma), and then the uterus, testes, thymus, intestines, thyroid, heart, and stomach (≈20~60 times plasma). The brain, adipose tissue, and muscles had lower levels (≈3~9 times plasma), while the bone marrow had the lowest levels (≈1.3 times plasma).

The tumor tissue AUC_{0-72} of the tumor-bearing mice is 4-6 times that of the plasma AUC_{0-72}.

After intragastric administration in rats, the distribution peak of HA121-28 was reached at 0.5 h post-dose in the liver, stomach, and intestines, and at 24 h post-dose in the testes. In most tissues, the content of HA121-28 reached the distribution peak at 4 h post-dose and gradually decreased thereafter. At 72 h post-dose, the content of HA121-28 in most tissues had decreased to 1/10 to 1/70 of the distribution peak, except for the adrenal glands, uterus, and testes, where the content of HA121-28 decreased to 1/8, 1/5, and 1/1.3 of the distribution peak, respectively.

1.3.2.3 Metabolism

In addition to the parent compound, 7, 7, and 4 metabolites were found in the plasma of rats, dogs, and monkeys, respectively. The main metabolic pathways include N-dealkylation, N-oxidation, O-dealkylation, dehalogenation, monooxygenation, glucuronidation, methylation, and various combinations of metabolic pathways.

[redacted]

[redacted]

[redacted]

1.3.2.4 Excretion

After administering HA121-28 via gastric lavage, the proportion of excretion through various routes in its original form was found to be feces (27.5%) > bile (3.91%) > urine (2.18%). The proportion of HA121-28 excreted through the intestines with feces was much greater than that excreted through the kidneys.

The total excretion rate of HA121-28 through urine and feces was 29.7%, and the absolute bioavailability of oral administration in rats was 93.8%, indicating that the clearance of HA121-28 absorbed into the body was mainly through metabolic pathways.

1.3.3. Toxicology Studies

1.3.3.1. Safety Pharmacology

Safety pharmacology results showed that a single oral dose of HA121-28 at doses of 30, 100, and 300 mg/kg did not affect respiratory function and neurological behavior in rats. In conscious, unrestrained Beagle dogs, a single oral dose of HA121-28 at doses of 5, 10, and 15 mg/kg, as well as 10 and 15 mg/kg of the marketed drug fentanyl, caused a mild prolongation of the Q-T interval and corrected Q-T interval. The degree of corrected Q-T interval prolongation caused by HA121-28 at the same dose was comparable to that of fentanyl.

1.3.3.2. Single-Dose Toxicity

Results of a single-dose toxicity study showed that administering HA121-28 orally via gavage to SD rats at doses of 500, 1000, and 2000 mg/kg resulted in deaths at doses of 1000 and 2000 mg/kg. The maximum tolerated dose (MTD) was 500 mg/kg. Gastrointestinal, hepatic, and immune system toxicity may have contributed to the rat deaths. Administering HA121-28 orally to Beagle dogs at doses of 500 and 1000 mg/kg resulted in an MTD of 1000 mg/kg.

1.3.3.3. Repeat-dose Toxicity

The results of a 28-day repeated dose toxicity study showed that the main target organs for toxicity of HA121-28 in SD rats given orally by gavage for four consecutive weeks were the liver, kidneys, immune system, intestines, female reproductive system, bones, teeth, and skin. The highest non-severely toxic dose (HNSTD) was 15 mg/kg. In Beagle dogs given HA121-28 orally for four consecutive weeks, the main target organs for toxicity were the immune system, gastrointestinal tract, and adrenal glands. The highest HNSTD was 12 mg/kg. Section 1.3.3.4 discusses genetic toxicity.

Genetic toxicity results showed that the in vitro Ames test, CHL test, and in vivo mouse micronucleus test were all negative, indicating that HA121-28 does not exhibit significant genetic toxicity.

2. Study Objectives

2.1. Dose Escalation Phase

Primary Objective:

- To observe the safety of HA121-28 tablets in humans, determine the dose-limiting toxicity (DLT) and maximum tolerated dose (MTD) of HA121-28 tablets in humans, and recommend a safe dose for the sample expansion stage.
- To preliminarily investigate the PK characteristics of HA121-28 tablets after single and multiple doses.
- To investigate the safety and tolerability of HA121-28 tablets.

Secondary objectives:

- To preliminarily observe the efficacy of HA121-28 tablets.

2.2. Dose expansion phase

Primary Objective:

- To investigate the PK characteristics of multiple doses of HA121-28 tablets and whether there is accumulation in the human body after multiple doses.
- To investigate the safety and tolerability of HA121-28 tablets.
- To determine the recommended dose for subsequent clinical studies.

Secondary objectives:

- To investigate the efficacy of HA121-28 tablets in patients with advanced solid tumors.

3. Overall Study Design

This study is a single-arm, open-label, dose-escalation, and dose-expansion phase 1 clinical study with single and multiple dosing.

This study is divided into two phases: a dose escalation phase and a dose expansion phase.

Dose escalation phase: exploration of dose-limiting toxicity (DLT) and maximum tolerated dose (MTD); preliminary study of PK of single and multiple doses; recommended dose selection for the sample expansion phase.

Dose expansion phase: multiple-dose PK study; safety and tolerability assessment; efficacy assessment; recommended phase 2 dose (RP2D) determination.

3.1. Dose Escalation Phase

The study will begin with a dose escalation starting from 25 mg, using a 3+3 study design. PK assessments and DLT event observations will be conducted during the initial single and multiple dosing in cycle 0 and cycle 1. After the completion of cycle 1, patients who are judged by the investigator to be able to continue treatment with the study treatment will receive treatment every 28 days until disease progression or intolerable toxicity occurs. Efficacy assessments will be conducted every two cycles.

3.2. Dose Expansion Phase

The maximum tolerated dose group (or 800 mg) obtained during the dose escalation phase and the dose group below the maximum tolerated dose determined by joint discussion between the investigator and the sponsor will undergo a multicenter, open-label extension study. This study plans to enroll a maximum of 150 patients with advanced solid tumors to evaluate the efficacy, safety, and PK characteristics of HA121-28 tablets in patients with advanced solid tumors. Eligible patients will receive treatment directly in the first cycle, with continuous administration for 21 days, followed by a 7-day rest period, and a 28-day treatment cycle until disease progression, intolerable toxicity reactions, withdrawal of informed consent, pregnancy, or the initiation of new anti-tumor treatment, whichever occurs first.

Process Design:

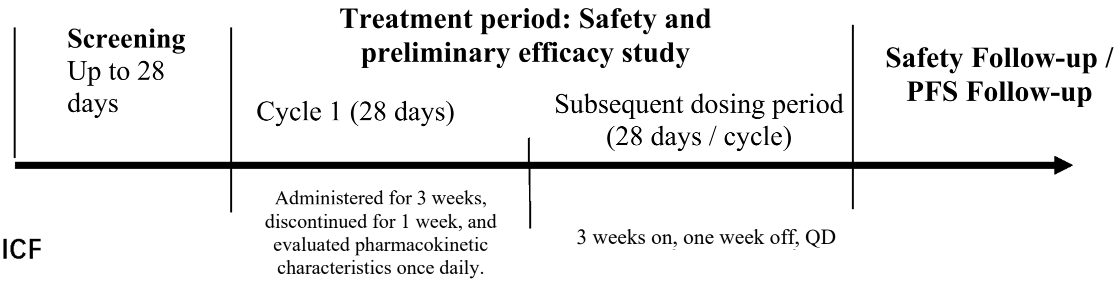

4. Investigational Product

Investigational product: HA121-28 tablets

Dosage Form: Tablet

Specification: 25 mg, 100 mg, 200 mg (as HA121-28 hydrochloride salt)

Expiry date: Tentatively 2 years

Storage condition: Stored below 30 °C

Providing organization: CSPC ZhongQi Pharmaceutical Technology (Shijiazhuang) Co., Ltd.

Method of use:

1) Oral administration with warm water.
2) Patients should be administered under a fasting condition in the morning. If a patient experiences significant gastrointestinal adverse reactions due to fasting, they may take the medication after a meal, and the time of the meal and medication should be recorded.

5. Study Population

5.1. Inclusion Criteria

Patients must meet all of the following inclusion criteria to be enrolled in this study:

1) Be willing to participate in the clinical trial and sign the informed consent;
2) Men and women aged 18 to 75 years;
3) Histologically/cytologically confirmed advanced/metastatic solid tumor, and have failed prior standard of care or for which no standard of care is durable (patients with RET fusion/mutation can be included regardless of whether they have received standard of care or not);
4) At least one measurable lesion according to RECIST 1.1;
5) Patients have not received any anti-cancer therapies, including chemotherapy, radiotherapy, targeted treatment, and surgery, within 4 weeks prior to participation. The washout period for traditional Chinese medicine or Chinese patent medicine with anti-tumor indications is 2 weeks. The washout period for local palliative radiotherapy for relieving bone metastasis pain is 2 weeks;
6) Eastern Cooperative Oncology Group (ECOG) Performance Status of 0~1;
7) Expected overall survival (Life expectancy) ≥ 3 months;
8) Laboratory test results must meet the following standards:

   Absolute neutrophil count (ANC) ≥1.5× 10^9/L; Platelet count (PLT) ≥75×10^9/L; Hemoglobin (Hb) ≥90 g/L (no blood transfusion within 14 days); Alanine aminotransferase (ALT) and aspartate aminotransferase (AST) ≤2.5× upper limit of normal (ULN) (in patients with liver metastasis ≤5.0× ULN); Total bilirubin ≤ 1.5× ULN; Serum creatinine≤ 1.5× ULN;
9) Male and female patients of childbearing potential should agree to use suitable methods of contraception during the treatment and 6 months after the last dose of study medication; female patients should have negative results of serum/urine pregnancy test within 7 days prior to enrollment and cannot be breastfeeding.

5.2. Exclusion Criteria

Patients who meet any of the following criteria are not eligible to participate in this study:

1) Has participated in other clinical trials and received the treatment within 4 weeks prior to enrollment;

2) Patients who cannot swallow or have chronic diarrhea and intestinal obstruction, which may affect the administration and absorption of the drug;

3) Participant who meets one of the following criteria:

   - Corrected QT (QTc) ≥470ms in women, ≥450ms in men; or congenital long QT syndrome (LQTS), taking QT-prolonging medications, and has a family history of long QT syndrome;

   - Resting ECG result shows clinically significant abnormalities of rhythm, conduction or morphology, requiring therapeutic intervention;

4) Urinalysis result shows protein in urine ≥ ++ and 24-hour urine protein > 1.0 g;

5) Based on the investigator's assessment, patients with known severe comorbidities which may influence the safety of the patients and the study completion [such as uncontrolled hypertension (systolic pressure ≥150 mmHg or diastolic pressure ≥100 mmHg, despite being treated with the optimal medicine), diabetes, etc.];

6) Patients who have symptoms of metastatic brain/meningeal tumors within 4 weeks of participation, unless the investigator determines that the clinical symptoms are stable and the patient is suitable for enrollment;

7) Ongoing adverse events (AEs) >grade 1 at the time of participation (except hair loss and pigmentation);

8) Patients who have undergone major surgery or have not recovered from Invasive operation within 4 weeks prior to initiation of study treatment;

9) Coagulation disorders (INR >1.5, prothrombin time (PT) > ULN+4s or APTT >1.5 ULN): with bleeding diathesis (such as active peptic ulcer) or receiving thrombolytic or anti-coagulant treatment;

10) Known pulmonary infection/ pneumonitis/interstitial pneumonia who are not suitable for the study;

11) Known active Hepatitis B or Hepatitis C virus infection;

- if the HBsAg result is positive, additional HBV DNA testing is required (the result is higher than the ULN of the study site);
- if the HCV antibody result is positive, additional HCV RNA testing is required (the result is higher than the ULN of the research center);

12) Known history of human immunodeficiency virus (HIV), or other acquired/congenital immune deficiency diseases or organ transplantation;

13) Other anti-tumor therapies are required (including radiotherapy, chemotherapy, immunotherapy, targeted treatment, traditional Chinese medicine, etc.);

14) Patients with a known history of neurological or psychiatric disorders, including epilepsy or dementia;

15) Not suitable for the treatment assessed by the investigators;

16 Cardiac ejection fraction less than 50%;

17) Patients who have suffered from or are complicated with any other malignant tumor within 5 years (except radically resected skin basal cell carcinoma, skin squamous cell carcinoma, superficial bladder cancer, local prostate cancer, in situ cervical cancer or other carcinoma in situ).

5.3. Discontinuation criteria:

The investigator must discontinue the administration of the study treatment to a patient if any of the following occur during the course of the study:

1) intolerable toxicity;
2) disease progression assessed by imaging;
3) The investigators believe that continuing to receive the study treatment treatment carries more risks than benefits;
4) Start a new anti-tumor therapy;
5) Patients are unwilling to continue to participate in the study;
6) Pregnancy;
7) Meet any of the withdrawal criteria.

Discontinuation of treatment by a patient does not mean that the patient has withdrawn from the study. The follow-up procedures specified in the study protocol should be completed unless the reason for discontinuation is death.

5.4. Withdrawal Criteria:

During the study, patients are allowed to withdraw from the study at any stage without compromising their interests. The patients must withdraw from the study if any of the following situations occur:

1) The sponsor decides to terminate the clinical study;
2) The relevant administrative and supervisory authorities require termination of the clinical study.
3) Ethics committees can terminate or suspend approved clinical studies.

5.5. Early Discontinuation/Withdrawal Post-treatment

When a patient terminates treatment or withdraws from a clinical study, the investigator should provide appropriate treatment immediately. The patient should complete the end-of-treatment (EOT) visit within 7 days after the last dose and notify the sponsor. The investigator should record the date and reason for termination/withdrawal in the source documents and eCRF records.

6. Design of Dose Escalation Phase

6.1. Estimation of Maximum Safe Starting Dose

For non-cytotoxic anti-tumor drugs, the initial dose for a single-dose administration is calculated using the No Observed Adverse Effect Level (NOAEL) in animals recommended by the FDA to estimate the Human Equivalent Dose (HED). According to the Estimation of the Maximum Safe Starting Dose in First-in-human Studies, the maximum recommended starting dose for the first-in-human study of a new drug is estimated based on the NOAEL in toxicology-related species or the most sensitive animal species. The specific calculation process is as follows:

a. To determine NOAEL of long-term toxicity testing in animals, this study used the dose that caused mild lesions in SD rats after 28 days of drug administration, which were recovered after cessation of treatment.

b. Calculate the human equivalent dose based on the coefficients of the human equivalent dose scale.

c. The safety factor is set to 5 (according to the Guidelines for Clinical Study of Antitumor: for some non-cytotoxic antitumor drugs, due to their relatively low toxicity, the starting dose calculation for Phase I clinical study can use 1/5 of the NOAEL in non-rodent animals in non-clinical studies).

d. Maximum recommended starting dose is calculated based on body weight.

See the following table for details:

Table 1 Conversion of Maximum Starting Dose

	Animal Species			
	28-day Sprague-Dawley rat		28 d Beagle dog	
NOAELs	15 mg/kg*		12 mg/kg*	
HED	15×(0.2/60)0.33 =2.28 mg/kg	15×0.16 =2.4 mg/kg	12 × (10/60)0.33 =6.64 mg/kg	12×0.54 =6.48 mg/kg
Safety factor, 5	0.456 mg/kg	0.48 mg/kg	1.328 mg/kg	1.296 mg/kg
Maximum recommended starting dose (BW: 60 kg)	27.36 mg	28.8 mg	79.68 mg	77.76 mg
Select the most appropriate/sensitive species		√		

NOAEL: No-observed-adverse-effect level
HED: Human equivalent dose
BW: Body weight
* The dose at which mild reversible lesions occur in rats and dogs, and which can be recovered after discontinuation of the drug.

6.2. Dose Escalation Scheme

The dosage of this study starts at 25mg/day/person and increases according to the modified Fibonacci method with a ratio of 100%, 100%, 100%, 50%, 50%, 30%... The starting dose is 25mg/day, with the aim of exposing the minimum number of patients to ineffective doses. Only one patient is enrolled in each of the 25mg/day and 50mg/day dose groups, while three patients are enrolled in the 100mg dose group to observe the safety and tolerability of the patients. Each patient receives only one corresponding dose. The study starts from the lowest dose, and the next dose group can only begin after the tolerability observation of all three patients in the previous dose group is completed. The grouping is shown in the table below:

Table 2 Dose Escalation Scheme

Grouping	Dose Escalation Proportion	Number	Dose (mg/day/person)	Dosing Regimen	Study Contents
1		1	25	25 mg*1	PK, tolerability
2	100%	1	50	25 mg*2	PK, tolerability
3	100%	3	100	100 mg*1	PK, tolerability
4	100%	3	200	200 mg*1	PK, tolerability
5	50%	3	300	200 mg*1+100 mg*1	PK, tolerability
6	50%	3	450	200 mg*2+25 mg*2	PK, tolerability
7	33%	3	600	200 mg*3	PK, tolerability
8	33%	3	800	200 mg*4	PK, tolerability

If a Grade 2 or higher drug-related toxicity occurs in Groups 1 and 2 (as determined by the investigator to be related to the study drug), then a dose-escalation study will be conducted using the 3+3 principle starting from that dose group.

6.3. Determination of Maximum Escalation Dose

Based on the phase 1 clinical study results of the positive reference vandetanib, the MTD was 500 mg/d. Taking into account the animal PK results, the C_{max} and AUC of HA121-28 at the same dose were about half of those of vandetanib, 500 mg/d * 2 = 1000 mg/d. The maximum dose of this study is currently set as 800 mg/d. If no DLT is observed in the 800 mg/d dose group during dose escalation, a meeting of investigators will be convened to decide whether to proceed to higher doses based on the obtained PK data.

6.4. Design of Dose Escalation

1) According to the above calculation, the dose escalation will start from 25 mg, and considering the marketed dose of 300 mg for a similar tyrosine kinase inhibitor vandetanib, the number of patients at low doses will be minimized. One patient will be enrolled in each of the 25 mg and 50mg dose groups. Subsequently, three patients will be enrolled in each dose group. If no DLT occurs in the three patients, the next dose group will be entered. If one patient experiences DLT, three additional patients will be enrolled in that dose group. If no DLT occurs in the additional three patients, the next dose group will be entered. If one or more DLTs occur in the additional three patients, the dose escalation will be terminated. If two patients experience DLT in a dose group, the dose escalation will be terminated. The dose before this dose will be considered as the MTD.

2) The observation period for DLT includes cycle 0 of single-dose administration and the first treatment cycle of multiple doses. Patients receiving continued treatment will undergo efficacy evaluation every two cycles. Although adverse reactions occurring in the second and subsequent cycles are not used as the basis for judging DLT, they will be discussed if they are severe.

3) Each group of patients can proceed to the next dose group only after the last patient in the group has completed the observation for the multiple-dose administration of cycle 1.

4) During the dose escalation phase, with the joint decision of the investigator and the sponsor, expansion can be carried out in the dose group that has completed the tolerability evaluation, but only for solid tumor patients with RET and FGFR gene alterations and no more than 6 cases.

6.4.1. Patient Replacement

Before initiating the study at a higher dose level, it is necessary to ensure that at least three patients receiving the current drug dose have been observed for a period of time, not less than one continuous treatment cycle or until a DLT is observed. If one patient does not experience a DLT and withdraws early, an additional patient is required for that dose group.

If a patient discontinues the medication for more than 7 days or discontinues the medication more than twice due to toxicity reactions, it can be considered as a DLT and no new cases will be added.

6.4.2. Treatment of Missed Dose

In the process of continuous medication study, if a patient misses a dose, the medication should be taken as soon as possible. The time for taking the medication should be at least 16 hours before the next scheduled dose. If there are less than 16 hours until the next dose, the medication should not be taken and the next scheduled dose should be taken as planned.

If vomiting occurs and medication is expelled, do not take supplements. Instead, proceed with the next scheduled dose and continue the study as planned. If vomiting persists, the investigator should take prompt action. Prophylactic antiemetics are not used in the study. If vomiting occurs, antiemetics may be used.

Drugs missed due to toxicity should not be taken as supplements.

6.5. Determination of DLT and MTD

DLT Definition:

The following events related to the study drug (including those that are related, probably related, and possibly related):

1) Grade 4 hematologic toxicity or grade 3 neutropenia accompanied by a fever of 38.5°C or higher, or grade 3 thrombocytopenia accompanied by severe bleeding.
2) The patient discontinues the medication for more than 7 days due to toxicity reactions/toxicity that does not resolve, or discontinues the medication more than twice due to toxicity reactions.
3) In the context of supportive treatment, diarrhea of grade 2 lasting more than 7 days, or grade 3 diarrhea lasting more than 3 days.
4) QTc interval prolongation: a single QTc interval ≥550 ms or an increase from baseline of ≥100 ms; or two consecutive QTc intervals ≥500 ms within 48 hours or an increase from baseline of ≥60 ms but <100 ms and the QTc interval value is ≥480 ms.
5) Grade 3 or higher skin toxicity;
6) Other grade 3 or higher non-hematologic toxicities (according to CTCAE V4.03 criteria).

MTD definition: The dose level immediately below the dose at which DLT occurs is defined as the maximum tolerated dose (MTD).

6.6. Single and multiple-dose pharmacokinetics

Two patients from the 25 mg/d and 50 mg/d dose groups will be selected for single- and multiple-dose PK pilot studies. PK parameters obtained from the pilot study results will be used to adjust the PK study and blood sampling time points for subsequent studies. Refer to the relevant SOP for detailed information on blood sample collection, processing, storage, transportation, and detection methods.

7. Design of Dose Expansion Phase

7.1. Dose Selection

After reaching the maximum tolerated dose during the dose escalation phase, it is recommended to use a dose below the maximum tolerated dose, which is determined through joint discussion between the investigator and the sponsor, as the maximum recommended starting dose for the expansion phase. The drug will be administered continuously for 21 days, followed by a 7-day rest period, constituting a 28-day dosing cycle. During the study, dose adjustments will be made based on the patient's tolerance, as detailed in section 10.2 on drugs that may be used at the investigator's discretion.

If the maximum tolerated dose is not reached during the dose escalation phase, a dose of 800mg or a dose below 800mg determined by the investigator and sponsor will be recommended as the maximum starting dose for patients in the expansion phase. This recommendation will be made in consultation with the sponsor.

If a patient misses a dose during the study, the same principles for dose escalation should be followed.

7.2. Rationale for Dose Selection

According to the results of preclinical studies, the anti-tumor efficacy of the drug increases with increasing doses, and the maximum target inhibition can be achieved at the maximum tolerated dose in clinical studies, resulting in the expected clinical anti-tumor efficacy. In addition, during the dose escalation phase of this phase 1 study, the selected dose has been preliminarily proven to be tolerable in patients with solid tumors. In order to further fully evaluate the safety and PK characteristics of HA121-28, and to preliminarily evaluate its anti-tumor activity, the investigator and the sponsor have jointly decided to expand the dose group below the maximum tolerated dose (or 800mg) appropriately.

8. Pharmacokinetic Study Design

8.1. Method of Administration

Single dose: HA121-28 tablets will be orally administered under a fasting condition in the morning. The drug will be administered on Day 1 of Cycle 0 (7 days), followed by a 6-day drug-free period. Tolerance, safety, and PK of the single dose will be observed and blood samples will be collected.

Multiple doses: Take HA121-28 tablets under a fasting condition in the morning, once a day for 21 consecutive days. Try to keep the administration time consistent for each administration. Discontinue for 7 days. Each cycle is 28 days. Observe tolerance, safety, and PK of multiple doses by collecting blood samples.

Follow-up treatment: Take HA121-28 tablets orally under a fasting condition in the morning, once a day for 21 consecutive days. Try to take the medication at the same time every day. Stop taking the medication for 7 days. Each cycle is 28 days. The investigator will determine whether the treatment can continue.

Patients in the single-dose phase will start fasting after 22:00 on the day before the study. Water

will be restricted for 1 hour before and after dosing (except for water used to take the drug), and fasting will be required for 4 hours after dosing. On the day of the study, patients will be administered HA121-28 tablets with 200 mL of warm water under a fasting condition.

Multiple-dose administration should be received with 200 mL of warm water under a fasting condition. Water intake is prohibited 1 hour before and after administration (except for water used to take the medication), and fasting is required within 2 hours after administration.

If there are obvious gastrointestinal adverse reactions due to fasting administration intolerance, the drug can be administered after a meal, and the time of eating and administration should be recorded. Smoking and alcohol are prohibited during the study, and beverages containing coffee, carbonated drinks, and citrus fruits are prohibited. Citrus fruits should not be eaten, and vigorous exercise should be avoided.

Administration Record: The time and dose of administration are recorded in the administration record table.

8.2. Blood Sampling (Dose Escalation Phase)

See SOP for blood sample collection and handling for details.

8.2.1. Single-dose Pharmacokinetic Study (Cycle 0)

Single PK blood sampling time points: pre-dose (0 h, blank value), 1 h, 2 h, 4 h, 8 h, 12 h, 24 h, 48 h, 72 h, 96 h, 120 h,144 h post-dose.

8.2.2. Multiple-dose Tolerability and Pharmacokinetic Study (Cycle 1)

Multiple PK blood sampling time points: pre-dose on Days 1, 8, 15, and 21 of Cycle 1, and post-dose at 1 h, 2 h, 4 h, 8 h, 12 h, 24 h, 48 h, 72 h, 96 h, 120 h, 144h, and 168 h on Day 21.

Note: Blood sampling can only continue for subsequent treatment after completing 168 hours of medication on Day 21 of Cycle 1. For the specific timing and time window of blood sampling, please refer to the table below.

Table 3 Blood Sampling Time Points and Blood Sampling Time Window

Visit Cycle		Sampling Time	
Cycle	Day	Scheduled Blood Sampling Time	Window
0	1	Pre-dose (0 h)	Within -30 min
		1 h	±1min
		2 h	±1min
		4 h	±3min
		8 h	±3min
		12 h	±6min
	2	24 h	±15min
	3	48 h	±15min

	4	72 h	±15min
	5	96 h	±15min
	6	120 h	±15min
	7	144 h	±15min
1	1	Pre-dose	Within -30 min
	8	Pre-dose	Within -30 min
	15	Pre-dose	Within -30 min
	21	Pre-dose	Within -30 min
		1 h	±1min
		2 h	±1min
		4 h	±3min
		8 h	±3min
		12 h	±6min
	22	24 h	±15min
	23	48 h	±15min
	24	72 h	±15min
	25	96 h	±15min
	26	120 h	±15min
	27	144 h	±15min
	28	168 h	±15min

Note: First, select the initial two dose groups (25 mg/d and 50 mg/d) for single and multiple-dose PK pilot studies using the up-and-down method to obtain PK parameters such as the half-life of HA121-28. After the pilot study, conduct formal PK studies for single and multiple doses in dose groups above 100 mg/d, as well as PK studies in the extended dose groups. Adjust the PK sampling time points based on the results of the pilot study.

8.3. Blood Sampling (Dose Expansion Phase)

During the dose escalation phase, after obtaining PK data from 12 patients in the expected dose group, the remaining patients will undergo sparse PK sampling. Accurate records of patient dosing and blood sampling times will be kept.

8.3.1 Close PK Sample Collection Period

Blood collection points for Cycle 1: before taking medication on Day 1 (-1h), and 1h (±5min), 2h (±5min), 4h (±10min), 8h (±10min), 12h (±15min), and 24h (±30min) after taking medication (blood collection on Day 2 before taking medication), before taking medication on Days 8, 15, and 21 (within -1h), and 1h (±5min), 2h (±5min), 4h (±10min), 8h (±10min), 12h (±15min), 24h (±30min), 48h (±1h), 72h (±1h), 120h (±2h), and 168h (±2h) after taking medication on Day 21.

Blood sampling for PK analysis will be conducted at any time on Day 28 of each cycle during the subsequent treatment period (Cycle 2 to Cycle 6).

8.3.2 Sparse PK Sample Collection

Blood collection points for Cycle 1: within 1 hour before dosing on Day 1, 4 hours after dosing on Day 1 (±10 minutes), within 1 hour before dosing on Days 8, 15, and 21, 4 hours after dosing on Day 21 (±10 minutes), and at any time on the Day 28 visit.

Blood sampling for PK analysis will be conducted at any time on Day 28 of each cycle during the subsequent treatment period (Cycle 2 to Cycle 6).

8.4. Biomarkers

This study investigates the use of RET, FGFR, EGFR, and VEGFR as biomarkers. Prior to treatment initiation, pathological reports of all enrolled patients are collected to record the gene expression and mutation status of RET, FGFR, EGFR/VEGFR (information is collected based on the actual situation of the patients, and further testing is not conducted for patients without corresponding tests).

The collected information will only be used for subsequent preliminary exploration of the correlation between biomarkers and drug efficacy, and will not affect the enrollment of patients.

9. Study Procedures

9.1. Screening:

Within 28 days before starting the study drug:

1) Signed informed consent;
2) Demographic and general information: gender, date of birth, height, weight, ethnicity.
3) Tumor history and treatment history: Pathological diagnosis of advanced solid tumor; History of tumor surgery, chemotherapy, and radiotherapy.
4) Medical history: treatment history of other major diseases;
5) Baseline tumor assessment
6) Echocardiography
7) Collection of biomarker information
8) Serum virological test

Within 7 days prior to starting the study drug:

1) Vital signs
2) Physical examination
3) ECOG performance score
4) Laboratory tests: Complete blood count, urinalysis, stool analysis, blood biochemistry, coagulation function, thyroid function, pregnancy test (only for female patients of childbearing age), myocardial enzyme spectrum test, electrocardiogram.

9.2. Single-dose Period (Cycle 0): applicable only for the dose escalation phase

In the expansion phase, patients will skip the single-dose stage of cycle 0 and proceed directly to the multiple-dose stage of cycle 1.

1) Administration: In the morning under fasting conditions
2) Vital signs (before administration and at 4 h, 12 h, 24 h post-dose and on Day 7)

Electrocardiogram (before administration and at 4 h, 12 h, and 24 h post-dose, and on Day 7).

Physical Examination (Day 2, Day 7 post-dose)

Hematology (pre-dose and Day 7)

Urinalysis (pre-dose and Day 7)

Blood chemistry (pre-dose and Day 7)

If the screening complete blood count, urinalysis, and blood biochemistry tests are performed within 3 days prior to administration, there is no need to repeat the tests before administration.

3) PK blood sample collection: pre-dose (0 h, blank value), 1 h, 2 h, 4 h, 8 h, 12 h, 24 h, 48 h, 72 h, 96 h, 120 h, 144 h post-dose.

9.3. Multiple-dose Period (Cycle 1):

1) Dosing: fasting in the morning, QD, for 21 days
2) Vital signs (Day 1, Day 8, Day 15, Day 21, Day 28)
3) Physical examination (on Day 1, Day 8, Day 15, Day 21, and Day 28)
4) Complete blood count (on Day 8, Day 15, Day 21, and Day 28).
5) Urinalysis (Day 15, Day 28)
6) Stool routine (Day 28)
7) Blood chemistry (Day 15, Day 28)
8) Electrocardiogram (Day 1, Day 8, Day 15, Day 21, Day 28)
9) Coagulation function (Day 28)
10) Thyroid function test (Day 28)

11) Echocardiography (Day 28)
12) ECOG performance score (Day 28)
13) Tumor assessment (Day 28)
14) PK blood sampling

9.4. Subsequent Treatment Period:

1) Dosing: In the morning, under fasting conditions, QD
2) Vital signs (Day 28)
3) Physical examination (Day 28)
4) Hematology (Day 28)
5) Urinalysis (Day 28)
6) Stool routine (Day 28)
7) Blood biochemistry (Day 28)
8) Electrocardiogram (Day 28)
9) Coagulation function test (Day 28)
10) Thyroid function test (Day 28)
11) Echocardiography (Day 28 of even cycles)
12) ECOG performance scoreECOG performance score (Day 28)
13) Tumor assessment (Day 28 of even cycles)
14) PK blood sampling (only applicable for dose expansion stage Cycle 2~Cycle 6)

9.5. End of Treatment:

1) Vital signs,
2) Physical examination,
3) Hematology,
4) Urinalysis,
5) Stool routine,
6) Blood biochemistry,
7) Electrocardiogram,
8) Coagulation function test,
9) Thyroid function test,
10) Pregnancy;

11) Echocardiography,
12) Tumor assessment,
13) ECOG performance score.

9.6. Follow-up

Progression-free Survival Follow-up

Patients who receive medication and discontinue treatment without progression will undergo follow-up for progression-free survival every 2 months (±7 days) with imaging examinations until disease progression, death, withdrawal of informed consent, or initiation of new anti-tumor treatment, whichever occurs first.

10. Concomitant Medications

10.1. Drugs Prohibited or Used with Caution

Other anti-tumor drugs:

During the treatment period, patients are not allowed to use any other anti-tumor drugs, including radiotherapy, chemotherapy, endocrine therapy, targeted and anti-angiogenic drugs, interferon, or any other drugs that may affect the efficacy evaluation. This includes Chinese herbal medicines that are indicated for anti-tumor effects in the CFDA-approved product labeling.

Drugs causing QT interval prolongation in the heart

During clinical use, it has been found that tyrosine kinase inhibitors have the effect of prolonging the QT interval. Therefore, it is required to use drugs that prolong the QT interval with caution during the study. These drugs mainly include, but are not limited to, the following categories:

Antibiotics (clarithromycin, azithromycin, erythromycin, roxithromycin, metronidazole, moxifloxacin);

Antiarrhythmic drugs (quinidine, sotalol, amiodarone, procainamide, procainamide).

Antifungals (fluconazole, ketoconazole)

Antimalarials (mefloquine, chloroquine)

Antidepressants (amitriptyline, imipramine, clomipramine, lofepramine, doxepin)

10.2. Medications that May Be Used as Appropriate in the Study

If a patient experiences adverse reactions, close observation should be conducted and symptomatic treatment should be given if necessary. The drugs used should be recorded and explained on the eCRF form.

Patients with bone metastases may be treated symptomatically with bisphosphonates and denosumab as appropriate. If pain from bone metastases cannot be effectively controlled with systemic or local analgesics, palliative small-area radiation therapy is allowed.

10.2.1. During Cycle 1 of Continuous Dosing

During the dose escalation phase, if the DLT toxicity specified in the protocol occurs, the drug should be temporarily discontinued and actively managed. The drugs used should be recorded in the eCRF. If the toxicity of the patient who experienced DLT recovers to ≤ Grade 1 after treatment, the drug can be administered at the original dose level (when the original dose is the lowest dose) or at a reduced dose level.

After determining the DLT and MTD, the management of AEs during the first cycle of continuous administration should follow the same principles as those for the second cycle of continuous administration.

10.2.2. During and after Continuous Dosing

After the occurrence of toxic reactions, the investigator can provide corresponding treatment and adjust the dosage of the study treatment according to the patient's condition. The principles of dosage adjustment for toxic reactions are recommended principles. The investigator can also judge whether to adjust the dosage and treatment based on the patient's risk-benefit and current condition, taking into account their own clinical experience and updated study data during the study. The time of adjusting the dosage of the study treatment, the level of dosage after adjustment, and the reasons for adjustment should be recorded in detail. The dosage level and principles of adjustment are detailed in the table below.

The study treatment allows for a maximum of two dose reduction levels. If more dose reductions are needed, a comprehensive evaluation by the investigator and sponsor is required and permission from the sponsor must be obtained. The maximum duration of drug interruption is 28 days. If the interruption exceeds 28 days, the patient must discontinue treatment. Unless the investigator determines that there is a clinical benefit to the patient continuing treatment and obtains permission from the sponsor, the patient must discontinue treatment.

Recommended Dose Modifications for Toxicity

**NCI-CTCAE V 5.0**	**Dose Modification**
**Liver**	
AST/ALT (Grade 2)	Continued treatment is recommended with weekly monitoring of liver function until it recovers to grade ≤1 or baseline level.
ALT/AST (Grade 3)	Discontinue medication temporarily and recommend monitoring liver function weekly until it recovers to grade ≤1 or baseline level. Then, reduce the dosage by one level and recommend monitoring liver function weekly for at least 4 weeks. If it occurs again, discontinue treatment.
ALT/AST (Grade 4)	Treatment discontinuation
ALT/AST>3×ULN (≥Grade 2) and total bilirubin>2×ULN.	Treatment discontinuation
**Skin adverse reactions (rash, hand-foot syndrome, etc.)**	
Grade 2	First occurrence: Supportive treatment until recovery to ≤ grade 1 or baseline level. The investigator may consider reducing the dose level or temporarily discontinuing the drug based on their judgment.<br>Reappear: Discontinue treatment until recovery to ≤ Grade 1 or baseline level, then resume treatment at a reduced dose level.
Grade 3	First occurrence: Discontinue treatment until recovery to ≤ Grade 1 or baseline, then resume at a reduced dose level.<br>Second occurrence: Discontinue treatment until recovery to ≤ Grade 1 or baseline, then resume at a reduced dose level.<br>3rd occurrence: Treatment discontinuation;
Grade 4	Treatment discontinuation
**QTc prolongation**	

QTc>500 msec or an increase in QTc≥60 msec.	After the initial electrocardiogram (ECG) collection, if the average QTc is >500 msec or increases by ≥60 msec compared to baseline during the same day's retest, the patient must suspend the medication until the QTc recovers to ≤1 grade or baseline and the dosage is reduced by one level. If it occurs again, the patient should discontinue the treatment.
**Diarrhoea**	
Grade 2	First occurrence: Support treatment until recovery to ≤ Grade 1 or baseline level. The investigator may consider reducing the dose level or temporarily discontinuing the drug based on their judgment.<br>Reappear: Discontinue treatment until recovery to ≤ Grade 1 or baseline level, then resume at a reduced dose level.
Grade 3	First occurrence: Discontinue treatment until recovery to ≤ Grade 1 or baseline, then resume at a reduced dose level.<br>Second occurrence: Discontinue treatment until recovery to ≤ Grade 1 or baseline, then resume at a reduced dose level.<br>3rd appearance: Treatment discontinued
Grade 4	Treatment discontinuation
**Hematological toxicity and other drug-related non-hematological AEs in the study.**	
Grade 3	Discontinue medication until recovery to ≤ grade 1 or baseline level, then reduce the dosage by one level.<br>If the AE occurs again, the investigator will determine whether to reduce the dosage or discontinue the treatment based on the patient's tolerance.
Grade 4	Treatment discontinuation
Note: The results of liver function monitoring in another hospital are accepted;	

The drugs used in the above treatment should be recorded in the eCRF form.

11. Safety and tolerability evaluation

11.1. Potential toxicity risk

Based on the results of acute and long-term toxicity studies in animals and references to similar mechanism products in the literature, HA121-28 may pose the following toxicological risks:

- General conditions: Headache, fatigue, somnolence;
- Skin manifestations: pruritus, rash (according to clinical studies of vandetanib, rash may occur within 2 weeks after discontinuation of the drug, and photoprotection should be considered when managing this symptom), eczema.
- Digestive system: Nausea, vomiting, diarrhea;
- Cardiovascular system: QTc prolongation, hypertension;
- Liver and kidney function: Alanine aminotransferase increased, serum creatinine increased

11.2. Safety Monitoring Measures

Vital Signs and Physical Measurements

During the designated time period of the study, the investigator should obtain the patient's height, weight, and axillary temperature.

When measuring systolic and diastolic blood pressure and pulse, the patients must first sit and rest for at least 3 minutes.

ECG Evaluation

Standard 12-lead electrocardiogram. The electrocardiogram report should include an overall assessment and confirm whether there are clinically significant abnormal findings, which should be described in detail if present. The signed original electrocardiogram will be archived at the study site.

Echocardiography

The investigators will perform echocardiography on the patients during the screening period, on day 28 of the first cycle, during even cycles of the follow-up treatment stage, and at the end of the study to monitor cardiac function.

Clinical Laboratory Evaluations

If laboratory measurements during the screening period exceed the range specified in the protocol for inclusion/exclusion criteria, the test should be repeated as soon as possible before enrollment to exclude laboratory errors. If the repeated measurement still exceeds the range specified in the protocol, the patient should be excluded from the study.

If a laboratory test range is not specified in the protocol and the measurement is outside the normal range of the study site during the screening period, the investigator should determine whether it has clinical significance based on the nature and degree of the observed abnormality. This measurement should be repeated as soon as possible and prior to enrollment to rule out laboratory error.

In all cases, investigators must document in source files the clinical decision regarding whether or not to allow patients to continue in the study based on whether the results have clinical significance and/or medical relevance.

Clinically significant and/or drug-related values should be recorded on the annotation page of the CRF, indicating the date, study day, and specific time.

Blood routine

Hemoglobin, red blood cell count, WBC count and classification (monocytes, eosinophils, basophils, neutrophils, lymphocytes) and platelet count.

Blood biochemistry

Total protein, albumin, globulin, blood glucose, total cholesterol, low-density lipoprotein, high-density lipoprotein, triglycerides, urea, creatinine, alkaline phosphatase, total bilirubin, direct bilirubin or indirect bilirubin, AST, ALT, calcium, phosphorus, magnesium, potassium, sodium, chloride, uric acid.

Urinalysis

Urinary specific gravity, pH, glucose, protein, bilirubin, ketones, white blood cells, and red blood cells.

Stool routine

Description, color, white blood cells, red blood cells, fecal occult blood.

Coagulation

Prothrombin time (PT), activated partial thromboplastin time (APTT), and international normalized ratio (INR) of plasma coagulation.

Thyroid function

Thyroid stimulating hormone (TSH), free triiodothyronine (FT3), and free thyroxine (FT4).

Pregnancy test

Female patients of childbearing age will undergo pregnancy testing during the screening period and at the end of treatment.

Myocardial zymogram

Creatine kinase (CK), lactate dehydrogenase (LDH).

Serum virology

HIV antibodies, HBV antibodies and viral load, HCV antibodies and viral load.

Adverse Events

This refers to the evaluation of the definition, severity, and relationship to study drug of AEs, as well as the collection, recording, and reporting of AEs.

11.3. Safety Evaluation Rules

Safety evaluation includes monitoring and recording all AEs and serious adverse events (SAEs), as well as laboratory tests mentioned above.

Adverse Event (AE)

All adverse medical events that occur in patients after receiving study drug may manifest as symptoms, signs, diseases, or laboratory abnormalities, but may not necessarily be causally related to study drug.

Adverse Drug Reaction (ADR)

Any harmful or unexpected reaction related to study drug that may occur during a clinical study. There is at least a reasonable possibility of a causal relationship between study drug and the AE, meaning that correlation cannot be ruled out.

Treatment Emergent Adverse Events (TEAEs) refer to adverse events that occur after treatment.

Events that occur during the course of treatment that are not present before treatment or have worsened compared to before treatment.

Serious Adverse Event (SAE)

An SAE refers to one of the following conditions that occur in a patient after receiving study drug in a clinical study:

a. Leading to death

b. Life-threatening

The term "life-threatening" in the definition refers to the risk of death for the patient at the time of the event, not the possibility of death if the event worsens.

c. Requires hospitalization or prolongation of hospitalization

However, the following admissions are not considered SAEs:

- Less than 24 hours in hospital
- Preplanned hospitalization

(For example, elective or scheduled surgeries arranged before the start of the study; hospitalization as part of the study procedure.)

- Hospitalization is not associated with AEs

(e.g., social hospitalization for short-term care).

d. Permanent or severe disability or loss of function

Loss of function refers to a severe impairment of the normal life ability of an individual.

e. Congenital anomaly or birth defect

f. Other medically significant events

Important medical events may not immediately endanger life, or lead to death, or hospitalization, but are generally considered serious if medical measures are needed to prevent any of the above situations. For example, important treatments in the emergency room, allergic bronchospasm at home, non-hospitalized cachexia or convulsions, drug dependence or addiction.

Suspected Unexpected Serious Adverse Reaction (SUSAR)

The nature and severity of the clinical presentation exceeded the suspected and unexpected serious adverse reactions that are documented in the investigator's brochure, package insert of marketed drugs, or product characteristics summary.

Adverse Events of Special Interest (AESI)

AESI (serious or non-serious) is a type of event that is of scientific and medical concern for the sponsor's drug or study project. These events usually require further investigation in order to describe their characteristics and gain understanding. Given the nature of the events, continuous monitoring is required, and a rapid communication mechanism should be established between the investigator and the sponsor.

Criteria for determination of severity of adverse events:

AEs are classified into grades 1-5 according to NCI-CTCAE 5.0, as shown in the table below.

Table 4NCI-CT CA E5.0 Grading Criteria

Grading	Judgment Criteria
Grade 1	Mild; asymptomatic or mild; only observed clinically or diagnostically; no treatment required.
Grade 2	Moderate; requires minor, localized, or non-invasive treatment; associated with age-appropriate limitations in instrumental activities of daily living*.

Grade 3	Severe or clinically significant but not immediately life-threatening; resulting in hospitalization or prolonged hospital stay; causing disability; limiting self-care activities of daily living**.
Grade 4	Life-threatening; urgent treatment indicated.
Grade 5	Deaths related to AEs.

Note: Activities of Daily Living (ADL)
*Instrumental Activities of Daily Living (IADL) refer to activities such as cooking, shopping for clothes, using the telephone, managing finances, etc.
**Activities of daily living (ADL) refer to the ability to perform self-care tasks such as bathing, dressing, eating, grooming, and taking medication, without being bedridden.
Not all AEs include all grades. Therefore, some AEs may have less than five available grades. Grade 5 (death) does not apply to certain AEs, and therefore is not applicable.
Note the difference between the severity and intensity of AEs. Severity is used to describe intensity, but not necessarily SAE. For example, headache may be rated as grade 3 (severe) in terms of intensity, but cannot be classified as a SAE unless it meets the SAE criteria.

Causality between adverse event and study treatment:

For all AEs, investigators should assess the causal relationship between each event and each study drug separately. The assessment of causality should be performed by an authorized clinical physician, who should provide a rationale for their judgment in addition to determining whether there is a causal relationship with the study drug.

Important factors to consider when assessing the causal relationship between AEs and the study drug include:

a. Temporal relationship to dosing

The event should occur after the drug administration. The time interval between drug exposure and the occurrence of the event should be evaluated in the clinical context of the event.

b. Response after discontinuation (dechallenge) and re-administration (rechallenge) of the drug.

The response of the patients after a dechallenge or a rechallenge should be evaluated based on the common clinical course of relevant events.

c. Underlying diseases, concomitant diseases, intercurrent diseases

Each event should be evaluated in the context of the current treatment of the disease and the patient's natural history and course of any other conditions.

d. Concomitant Medications or Treatments

The investigator should examine the other drugs or treatments that the patient is receiving when AEs occur, to determine if they may have contributed to the event.

e. Known types of reactions (clinical/preclinical) for this type of medication.

f. Exposure to physical/psychological stress

Exposure to stress may induce adverse changes in the patients and provide a more reasonable explanation for the event.

g. Pharmacology and Pharmacokinetics of Study Treatment

The PK properties of the therapeutic agent (absorption, distribution, metabolism, and excretion) as well as the pharmacodynamics of individual patients should be taken into account when considering the study treatment.

Causality between adverse events and study procedures and procedures:

Based on the question of whether AEs are causally related to the study procedures and operations, the evaluation results should be recorded as "related" or "unrelated" in the CRF. This generally refers to one of the following situations: AEs caused by any clinical operation (such as tissue biopsy); AEs caused by drug discontinuation(such as washout), dose reduction, or adjustment of treatment regimen required by the study procedures; AEs caused by other prophylactic drugs administered before the study drug.

Criteria for determination of relationship to study drug:

The relationship between the drug and AEs in the study is classified as: not related, possibly unrelated, possibly related, probably related, and related.

Related: There is evidence of using study drug, the occurrence of AEs is temporally related to the use of study drug, and the explanation of AEs by study drug is more reasonable than by other reasons. Positive dechallenge, positive rechallenge (if feasible).

Probably related: There is evidence of using the study drug, and the occurrence of AEs has a reasonable temporal sequence with the use of the study drug. AEs are more reasonably explained by the use of investigational drugs than by other reasons. Positive withdrawal reaction.

Possibly related: There is evidence of the use of study drug, and the occurrence of AEs is reasonably correlated with the use of study drug in time. AEs may be explained by other reasons. The discontinuation reaction is positive.

Possibly unrelated: There is evidence of using study drug, and the AE that occurred may be better explained by other reasons. Discontinuation reaction is negative or unclear.

Not related: The study drug has not been used, or there is no correlation between the use of study drug and the occurrence of AEs, or there is another clear cause for the AE.

Based on the answers to the following 5 questions, determine the relationship between the drug and the AE:

1) Is there a reasonable temporal relationship between the start of medication and the occurrence of AEs?

2) Does the suspected adverse reaction match the known types of adverse reactions for the drug?

3) Can the suspected adverse reaction be explained by the effects of the medication used, the patient's clinical condition, or the influence of other therapies?

4) Does the reaction decrease or disappear after discontinuation or dose reduction?

5) Will the same reaction occur if the suspicious drug is taken again?

Table 5 Determination of Relationship between Adverse Events and Study Treatments

	Related	Probably related	Possibly related	Possibly unrelated	Not related
a. Is there a reasonable temporal relationship between the event and study drug?	+	+	+	+	-
b. Does the event match the known adverse reactions of the drug?	+	+	±	-	-
c. Does the event resolve or disappear after discontinuation or dose reduction?	+	+	±?	±?	-
d. Does the event reappear after re-dosing?	+	?	?	?	-
e. Can the effect of combined medication, progression of patients' condition, and the impact of other treatments be used to explain the event?	-	-	±	±	+

Explanation: + means positive; - means negative; ± means uncertain; ? means unclear.

Action Taken with Study Treatment

Record all measures taken to address the AEs related to study drug according to the following categories. The specific measures should be detailed and recorded in the CRF.

- Permanent discontinuation
- Dose reduction
- Dose increase
- Dose unchanged
- Drug interruption
- Unknown
- N/A

Other Specific Treatment for Adverse Events

- No treatment was received
- Drug therapy
- Other treatments

Outcome

The outcome of the AE will be recorded as follows:

- Recovered/resolved
- Recovering/resolving
- Not recovered/not resolved/ongoing
- Recovered/resolved with sequelae
- Fatal
- Unknown

Physical examination and laboratory evaluation

The investigator should list the normal range of values for all examination items, the standard deviation between each evaluated parameter and the control value, and evaluate each laboratory test value that exceeds the reference range. Laboratory test values that are considered significant and/or considered related to the study drug should be noted in the case report form, along with the date and time of the study. The investigator may repeat any abnormal results to eliminate examination errors. The evaluating physician should date and sign each form. Any clinically significant deviations in laboratory test results that occur during or at the end of the study must be reported to the sponsor and discussed with clinical experts, and must be re-evaluated until the results are normal or the changes are no longer relevant clinically.

Collection, Recording and Evaluation of Serious Adverse Events

Once an AE/SAE is reported, the investigator must make every effort to follow up with each patient. All AEs/SAEs must be followed up until recovery (to baseline or complete recovery) or until clinical stability is achieved. Once the issue is resolved, the case report form for the AE/SAE will be promptly updated. The investigator is responsible for adding any necessary additional examinations during follow-up, which may help clarify the nature and/or cause of the AE or SAE. This may include additional laboratory tests or studies, histopathological examinations, or consultations with other medical professionals.

The sponsor may require the investigator to perform or arrange for additional tests and/or assessments.

The investigator is responsible for collecting all adverse medical events that occur from the signing of the informed consent form to the end of the safety follow-up period (four weeks after the last dose of the drug). After the safety follow-up period ends, AE will no longer be actively collected. If an SAE occurs and the investigator considers that there is a reasonable causal relationship between the SAE and study drug, it should be reported to the sponsor according to the SAE reporting process. For AEs that are not fully recovered or stabilized at the end of the safety follow-up period (regardless of causality), follow-up must be conducted until recovery (baseline level or complete recovery) or clinical stability is achieved.

Clinical AEs occurring between the signing of the informed consent form and the first drug administration may be recorded in the case report form (CRF) as medical history/concomitant disease, unless they meet one of the following criteria, in which case they should be recorded as AEs: any harm/damage caused by clinical laboratory examination procedures; AEs caused by discontinuation related to the study protocol; AEs caused by drugs other than study drug taken as part of the treatment regimen.

All AEs that occur from the time of drug administration until the end of the safety follow-up period must be recorded. The recorded information includes the name of the AE, the start and end dates (and times) of the AE, severity, seriousness, the investigator's assessment of the relationship between the AE and study drug, measures taken for study drug in response to the AE, and outcome.

- If a patient dies, the cause or symptom leading to the death should be reported as an AE name. "Death" should be considered as an outcome of AE. If the cause of death is unknown, "death of unknown cause" should be reported as the AE name.
- Events that clearly correspond to the progression of the tumor disease should not be recorded as AEs, such as jaundice caused by tumor progression compressing the bile duct, pain caused by bone metastasis, or increased intracranial pressure caused by brain metastasis, unless the investigator considers the progression to be atypical or accelerated, or caused by study drug. However, any death events caused by any reason during the safety follow-up period should be reported to the sponsor according to the SAE process.

The investigator is responsible for determining the causal relationship between AEs and study drugs, as well as the study procedures and operations.

For SAEs, the sponsor must conduct a separate assessment of their expectedness, severity, and causal relationship with study drug. The reference document for expectedness assessment is the most current version of the Investigator's Brochure (IB) in effect.

Reporting of Serious Adverse Events

Investigator Responsibilities

Reporting Sponsor

For all SAEs occurring within four weeks from the first dose to the last dose of the drug, the investigator must report to the sponsor within 24 hours of becoming aware. In the case of reports involving death, the investigator should provide the sponsor with other necessary information, such as autopsy reports and final medical reports.

Reporting Ethics Committee

The investigator should promptly acknowledge receipt and review of relevant safety information provided by the sponsor of a clinical study, consider the treatment of the patients, make appropriate adjustments if necessary, communicate with the patients as early as possible, and report any suspected unexpected serious adverse reactions provided by the sponsor to the ethics committee. In the case of a report involving a death event, the investigator should provide the ethics committee with other necessary information, such as autopsy reports and final medical reports.

Responsibilities of the Sponsor

The sponsor should report suspected and unexpected serious adverse reactions to the drug regulatory authority and the health department in a timely manner. The reporting time limits are as follows:

- For fatal or life-threatening SUSARs, they should be reported within 7 days of first knowledge (day 0 being the day the sponsor first becomes aware) and followed up with additional information within the following 8 days.
- Report non-fatal or non-life-threatening SUSARs within 15 days.
- For follow-up reports, report within 15 days of obtaining new information.

The sponsor should promptly report suspected and unexpected serious adverse reactions to all investigators and clinical study sites participating in the clinical study, as well as to the ethics committee.

Adverse Events of Special Interest

For AESI of severity grade ≥3 according to CTCAE or meeting the criteria for SAE, the investigator should report to the sponsor according to the SAE reporting process within 24 hours of becoming aware of the event. Other AESI should be reported to the sponsor within 7 days.

AESIs in this study are:

(1) QT interval prolongation

Pregnancy

The investigator must report all pregnancy events that occur in female patients during the course of this study to the sponsor. Pregnancy outcomes should be carefully followed up and any abnormal outcomes in the mother or infant should be reported.

If a male patient's partner becomes pregnant, information regarding the pregnancy process and outcome should be obtained with the partner's consent as much as possible.

Once a pregnancy event occurs during the study, the investigator should communicate with the patient rigorously based on drug information science, informing her/him of the potential impact and risks of the study treatment on pregnant women and fetuses. If a female patient experiences a pregnancy event, the investigator should immediately suspend the clinical study for that patient and discontinue the study treatment.

Within 24 hours of confirming a pregnancy event in a patient (or in a patient's sexual partner), the investigator should complete a pregnancy report form and report to the sponsor (institutional review board, if required).

Upon knowledge of the pregnancy outcome, the investigator will complete the pregnancy report form and follow-up report within 24 hours of notification, and report to the sponsor (institutional review board, if required).

11.4. Efficacy Evaluation

Evaluate the efficacy of the patients according to RECIST 1.1 criteria.

Primary efficacy measure: Objective response rate (ORR).

ORR is defined as the percentage of all enrolled patients who achieve a complete response (CR) or partial response (PR) from the first day of treatment. For patients who meet the criteria for CR or PR, their response must be confirmed by repeat measurements using the same assessment method, with the repeat assessment performed at least 4 weeks after the initial assessment of CR or PR.

Secondary efficacy measures: progression-free survival (PFS) and disease control rate (DCR).

PFS refers to the duration from the first day of drug administration to the date of tumor progression (assessed by imaging diagnosis as PD) or all-cause death (whichever occurs first).

DCR is defined as the percentage of enrolled patients who achieve a complete response (CR), partial response (PR), or stable disease (SD) from the first day of treatment.

12. Data Review and Database Management

The electronic data capture (EDC) system is used for data collection in this study, and the clinical records serve as the source documents for this study.

12.1. Construction of Electronic Case Report Form

Construct an electronic case report form (eCRF) based on the clinical study protocol and data management plan (DMP), and release it after internal and external testing. The eCRF release and modification should be version-controlled.

12.2. Permission Assignment

The data manager creates accounts for different roles, such as investigators, sponsors, clinical research associates, and auditors, and grants different levels of access to the EDC system. For example, investigators at each site can only view the content of their own site and have the ability to modify data. Sponsors are only allowed to browse all case information. Monitors and auditors can read case information from each site but do not have the ability to modify data. However, they can insert comments or questions.

Role	Permission				
	Data Entry/Modification	Query Sending/Approval	Question Response	Data Review	Data Lock
Investigator/Study Associate	■	□	■	□	□
Data Manager	□	■	□	□	■
Clinical Research Associate	□	■	□	■	□
Project Manager	□	□	□	□	□
Observer	□	□	□	□	□

■Representative users with reading and writing disabilities have operational rights to the corresponding functional modules.

□ On behalf of the read-only user, accessible only, inoperable

12.3. Data Entry

The investigator or a data entry personnel designated by the investigator (clinical coordinator) should enter the data from the study records into the eCRF in a timely and accurate manner, following the EDC system manual. The eCRF is not considered as the source document, and its content is derived from the clinical records.

12.4. Data Cleaning

Data cleaning work includes data verification (systematic and manual logic checks), raising queries, answering investigator questions, data updates, and continuing the process until queries are resolved.

Data managers and clinical research associates perform real-time data cleaning work through the EDC system. If any issues are found, they can raise questions online at any time, and investigators can provide answers and modify incorrect data online. At the same time, data managers verify the data in response to questions and can repeat questions if necessary.

12.5. On-site Verification of Source Data

The clinical research associate conducts 100% source data verification (SDV) of the eCRF data on site, and raises questions in real time online if there are inconsistencies with the study medical records data, which the investigator answers.

12.6. Data Locking and Export

After each patient completes the study and the data is reviewed and cleaned by the clinical research associate, the data is locked by the data manager until the data of the last patient is locked. After all data is locked, the data manager imports it into the designated database and submits it to the statistician for review.

13. Statistical Analysis

This study includes two stages: dose escalation and dose expansion, which will be analyzed separately.

13.1. Analysis Sets

Safety Set (SS): All patients who have received at least one dose of the study treatment and have safety assessments recorded. Safety data must not be carried forward. The incidence of AEs is calculated using the number of patients in the safety set as the denominator.

PK Concentration Set: All patients who take at least one dose of the study treatment and at least one measurement of the study treatment concentration in blood during the study.

PK parameter analysis set: All patients who take at least one dose of the study treatment, and at least one valid PK parameter of the study treatment can be calculated.

Full Analysis Set (FAS): All patients who are enrolled and have received at least one dose of the study treatment. This set is used for baseline data and progression-free survival analysis.

Evaluable for response analysis set (ERS): All patients who have received at least one dose of study treatment and have had one tumor response evaluation. This set is used for efficacy measure analysis based on RECIST 1.1 criteria, including objective response rate and disease control rate.

13.2. Data Analysis Content

13.2.1. Case Enrollment Analysis

- Analysis using FAS
- The number of enrolled and completed patients will be listed to determine the analysis set. Dropouts and excluded patients and their reasons will be listed.

13.2.2. Demographic Data and Baseline Analysis

- Analysis using FAS

- Descriptive statistics of demographic data and data of other baseline characteristics:
- Calculate the frequency, mean, standard deviation, median, minimum and maximum values of the continuous variable.
- Use counting and ranking data to calculate frequency and constituent ratios.

13.2.3. Safety Analysis

- Analysis using SS set
- Calculate the incidence of AEs and adverse reactions
- List AEs and adverse reactions by subsystem, calculate frequency and percentage of occurrence.
- Detailed Listing of Cases of Adverse Events
- Detailed Listing of Cases of Various Adverse Reactions
- Number of patients with laboratory tests, electrocardiograms, and physical examinations changing from normal to abnormal or with worsening abnormalities after the study, as well as conversion-to-abnormality rates.
- List patients with abnormal laboratory tests, electrocardiograms, and physical examinations, and provide clinical interpretation.

13.2.4. Pharmacokinetic Analysis

- Using the PK concentration set, plot the concentration-time (c-t) curve based on the blood drug concentration (c) and time (t) data obtained from each patient in the experiment. Also, list the mean and standard deviation of drug concentration at each time point and plot the average blood concentration curve with its standard deviation.
- Analyze PK parameters using a non-compartmental model to calculate PK parameters for each patient, including: C_{max}, AUC_{0-24}, AUC_{0-t}, AUC_{0-inf}, T_{max}, V_z/F, $t_{1/2z}$, CL_z/F, and $\%AUC_{ext}$ after the first dose (single dose) of the drug. Also calculate C_{max}, $AUC_{0-\tau}$, AUC_{0-t}, AUC_{0-inf}, T_{max}, V/F, $t_{1/2z}$, CL_z/F, and $\%AUC_{ext}$ after multiple doses of the drug. Calculate the accumulation index $R1_{ac}=C_{max}$ (D21)/C_{max} (first dose) and $R2_{ac}=AUC_{0-\tau}/AUC_{0-24}$(first dose). Additionally, calculate the mean and standard deviation for each parameter.
- Due to the limited number of patients in each dose group, an exploratory power function model will be used to analyze the linear relationship of single-dose multiple doses. The linearity will be determined by the inclusion of the 90% confidence interval of the power function model exponent.

13.2.5. Efficacy Analysis

- FAS and ERS will be used for analysis.
- Efficacy measures: Objective response rate (ORR), progression-free survival (PFS), disease control rate (DCR); ORR is defined as the percentage of all enrolled patients who achieve a complete response (CR) or partial response (PR) from the first day of treatment.

PFS refers to the duration from the first day of drug administration to the date of tumor progression (assessed by imaging diagnosis as PD) or all-cause death (whichever occurs first).

DCR is defined as the percentage of enrolled patients who achieve a complete response (CR), partial response (PR), or stable disease (SD) from the first day of treatment.

Objective response rate (ORR): Calculate the objective response rate for each dose group and calculate the 95% confidence interval using the Clopper-Pearson method. In addition, the best overall response will be summarized by dose group in a countable manner. The objective response rate and best overall response will be analyzed based on the evaluable data set. A list will be provided to display the best overall response for each patient.

Disease control rate (DCR): Calculate the disease control rate for each dose group and calculate the 95% confidence interval using the Clopper-Pearson method.

Progression-free survival (PFS): The median time and 95% confidence interval will be estimated using the Kaplan-Meier method, and the corresponding survival curve will be plotted.

13.3. Interim Analysis

The analysis of safety and efficacy data will be conducted after the dose escalation phase of this study.

14. Management Procedures

14.1. Protocol Compliance

Investigators should be aware that they should proceed with caution to avoid deviating from the protocol. Under no circumstances should investigators request the sponsor or its representative to approve a deviation from the protocol. The only situation in which a deviation from the protocol is permissible is when it is necessary to protect the safety of the patients and to eliminate immediate hazards.

If the investigator believes that a certain protocol violates the principles of improving the study, the protocol should be revised. Such revision can only be implemented with the agreement of the sponsor and the ethics committee.

All significant protocol deviations should be documented and reported in the clinical study report. This includes the following protocol deviations:

1) Impact on the safety of patients;

2) Impact on the eligibility of patients who are enrolled in the study or continuing the study.

3) Impact on data analysis.

14.2. Protocol Amendment

Investigators should exercise caution to avoid deviating from the protocol. Under no circumstances should investigators deviate from the protocol. The only situation in which deviation from the protocol is permissible is when it is necessary to protect the safety of the patients and to eliminate immediate hazards.

Any changes or additions to the study protocol must be provided in writing.

Any modifications that significantly affect patient safety, the scope of the study, or the scientific quality of the study must be approved by the study site's ethics committee. Examples of such modifications include:

1) Increase or decrease the dosage or duration of medication for the patients.

2) Significant changes in study design (such as adding or removing study groups);

3) Test procedures are added or removed for safety monitoring.

14.3. Administrative Revisions

Modifications only involved in the study management do not require approval from the ethics committee, but the committee must be informed of these modifications. Examples of management changes that can be considered administrative modifications and do not require written protocol revisions or ethics committee approval include:

1) Changes in study monitors (such as changes in the sponsor).

2) minor changes in drug delivery;

3) Change of CRF shipping address.

14.4. Management of Investigational Product

The sponsor provided labels and packaging for all study medications.

The study site must have a designated person responsible for receiving, handling, and properly storing investigational products. The study drugs must be kept in a secure location and only accessed by investigators and authorized personnel. Once received, the investigational product must be stored according to the conditions specified on the drug label.

Adequate supervision of storage conditions is necessary, and relevant temperature/humidity logs must be kept as source documents. Investigators must accurately record drug transportation and distribution on drug accountability records. The study monitor should conduct a drug inventory during site visits or at the end of the study.

To prepare for drug dispensing in the study, the drug will be sent to the pharmacist at the study site. The study site should keep accurate records and properly store the process of administering the drug to each patient.

All provided drugs must be used for this study and cannot be used for other purposes. Unless there are special requirements from the sponsor, investigators cannot destroy any drug labels or any partially used or unused drugs.

Only after receiving written authorization from the sponsor, the investigator/designated personnel send all unused and partially used drugs as well as empty packaging to the designated destruction address, or have the unused or partially used drugs and empty packaging destroyed by the site pharmacist, and keep the drug destruction certificate.

14.5. Sample Collection Management

Sample collection time strictly follows the experimental protocol. In case of conflict between blood collection, medical evaluation, and meal times, blood collection takes priority.

15. Quality Control and Assurance of the Study

15.1. Ward and Laboratory Quality Control

Clinical study wards must meet standard requirements. Before conducting the study, investigators should simulate the feasibility of on-site rescue operations, check that all medical equipment is functioning properly without any malfunctions, and conduct instrument study runs. For the study ward, standard operating procedures and quality control programs should be established for experimental observation measures. All study processes should be carried out in accordance with the relevant standard operating procedures (SOP) of this site.

15.2. Qualifications of Testing Facility and Study Personnel

The testing facility must be a clinical drug study institution with clinical study qualifications determined by the National Medical Products Administration. Investigators must be physicians and related medical personnel who have undergone GCP and study protocol training and work under the guidance of senior professionals. Before the clinical study begins, investigators, doctors, and nurses must receive study protocol and GCP training related to the study.

15.3. Investigator Training

Before the start of a clinical study, the project director should provide training on the study protocol to the investigators.

15.4. Monitoring of Clinical Studies

The monitor appointed by the sponsor conducts regular on-site visits to the study hospital to ensure strict adherence to the study protocol and to inspect the source data to ensure consistency with the content on the CRF.

On-site monitoring

Before the start of the study (such as when the study site initiates visits or during investigator meetings), the sponsor representative will review the study protocol and CRF with the investigators and patients. The CRF is not the source document, and all data entered in the CRF must be traceable to the original electronic or paper records, including electronic data or patient files.

During the study, the investigator will visit the study site to monitor the execution of the following steps, including:

1) Completeness, consistency, and accuracy of CRF completion.

2) Confirmation of source data;

3) Quality control of CRF entry;

4) the progress of study enrollment;

5) Compliance with the study protocol and GCP;

6) Confirm that the study drug is being stored, dispensed, and accounted for correctly according to the protocol instructions (if it is a blinded study, dispensing and accounting may need to be done after database lock).

Audit

In addition to routine monitoring procedures, the sponsor will conduct quality control of the clinical study in accordance with relevant SOPs to evaluate compliance with GCP principles. The CFDA may also conduct inspections during or after the study.

16. Ethical Requirements

Before the start of the study, the study protocol and informed consent form must be reviewed and approved by the Institutional Review Board (IRB/IEC/REB). The sponsor must obtain a signed and dated approval letter before initiating the study, which confirms that the study protocol and informed consent form have been approved by the ethics committee. Prior to the start of the study, the investigator and sponsor should sign the protocol signature page, confirming their agreement to conduct the study in accordance with the above documents and all guidelines and procedures outlined in the protocol, and allowing the sponsor's monitor, designated sponsor representatives, ethics committee, and relevant regulatory authorities to

access relevant data and records. If the regulatory authorities require an inspection of the clinical study site, the investigator must immediately notify the sponsor.

If there are any issues during the actual implementation of this clinical study protocol, revisions to the study protocol must be submitted to the ethics committee for approval before implementation. If new important information regarding the study treatment is discovered, the informed consent form must be revised in writing and submitted to the ethics committee for approval before obtaining consent from the patients again.

The investigator must explain to each patient (or their legal representative) the nature, purpose, specific schedule, potential risks and benefits, and any discomfort that may arise from the study. Each patient must be informed that participation in the study is voluntary, and they may withdraw from the study at any time without affecting their future treatment or relationship with their physician.

If the patient's representative signs the informed consent, the patient should be informed of the study to the extent possible for the patient to understand. If the patient is capable, he/she should personally sign the written informed consent or a separate consent form with the date indicated. The informed consent form must be obtained from the patient before implementing any specific study procedures (i.e., all steps described in the protocol). The process of obtaining informed consent should be documented in writing in the patient's source documents.

Informed consent must be provided in standard written form, avoiding the use of professional terminology and using language that can be understood by the patients. The patients should read and understand the content before signing and dating and keep a signed copy of the document. Patients cannot participate in the study without obtaining informed consent.

17. Study Summary Report

Write a summary report of the study based on the relevant regulations issued by CFDA.

18. Data Storage

Important documents (including the following essential documents) must be retained by the investigator until the time required by relevant regulations (usually 5 years after the end of the clinical study or final marketing approval). The sponsor will notify the investigator/institution when it is no longer necessary to retain study-related documents. The investigator must sign the signature page of the protocol to indicate agreement to comply with the document retention requirements. Essential documents include, but are not limited to:

1) Study report and all attachments stamped with the official seals of the study sites, testing facility and sponsor.

2) Clinical trial approval from the National Medical Products Administration.

3) Certificate of Analysis of the Study Drug

4) Approval of the study protocol and all modifications by the ethics committee.

5) All original documents and laboratory records

6) CRF

7) Informed Consent Form

8) Chromatographic analysis methods and graphical representation of blood drug concentration analysis in study patients.

9) Calculation Results of Pharmacokinetic Parameters

10) Pharmacokinetic Study Original Notebook

11) Other relevant test documents

19. Publication of Study Results

The ownership of the study results belongs to the sponsor. The sponsor does not restrict the investigator from publishing any information collected or generated during the study, regardless of whether the results are favorable to the study treatment. However, to prevent inadvertent disclosure of confidential information or unprotected inventions, the investigator should provide the sponsor with an opportunity to review any proposed publications or other forms of disclosure before submission or public release. The investigator should provide the sponsor with a copy of any planned publication (poster, invited speech, or guest lecture) manuscript, abstract, or full text at least 30 days before submission for publication or other form of public release. If patent protection is necessary to protect intellectual property, the investigator should agree to delay publication. Prior to public disclosure, the investigator may be required to delete any previously unpublished confidential information (excluding study results). If the study is part of a multicenter study, the investigator must agree that the first publication will be the combined results of all study sites. However, if a manuscript for publication of the combined analysis has not been submitted within 12 months of completion or termination of the study at all study sites, the investigator may independently publish the results in accordance with other requirements of this section.

20. Reference Regulation

[1] National Medical Products Administration. Guidelines for Clinical Pharmacokinetic Studies of Chemical Drugs, 2005.

[2] National Medical Products Administration. Guidelines for Clinical Studies of Antitumor Drugs, 2012.

[3] National Medical Products Administration. Measures for the Administration of Drug Registration, 2007.

[4] National Medical Products Administration. Good Clinical Practice (GCP), 2003.

[5] Declaration of Helsinki, 2013

[6] Appendix 9012 Guidelines for the Validation of Quantitative Analysis Methods for Biological Samples, Volume IV, Chinese Pharmacopoeia (2015).

Appendix I Schedule of Assessment

Item	Screening		Single dose (Cycle 0) (Only applicable for dose escalation phase)				Multiple doses (Cycle 1)					Subsequent treatment	End of study	Progression-free Survival Follow-up
	（-28~0）	（-7~0）	D1	D2	D3-D6	D7	D1	D8 （±1）	D15 （±1）	D21 （±1）	D28 （±3）	D28 （±3）	End of Treatment/ Withdrawal	Every 2 months (± 7 days)
Signed informed consent	×													
Demographic and General Information [1]	×													
Tumor History/Other Medical History [2]	×													
ECOG performance score		×									×	×	×	
Vital signs [3]		×	×	×		×	×	×	×	×	×	×	×	
Physical examination [4]		×		×		×	×	×	×	×	×	×	×	
PK blood sampling [5] [6]			×	×	×	×	×	×	×	×	×	×		
Biomarkers [7]	×													
Hematology [8]		×	×			×		×	×	×	×	×	×	
Urinalysis [9]		×	×			×			×		×	×	×	
Stool routine [10]		×									×	×	×	
Blood chemistry [11]		×	×			×			×		×	×	×	
Coagulation [12]		×									×	×	×	
Thyroid function [13]		×									×	×	×	
Serum virological testing [14]	×													
Pregnancy test [15]		×											×	

<table>
<tr><th rowspan="2">Item</th><th colspan="2">Screening</th><th colspan="4">Single dose (Cycle 0)
(Only applicable for dose escalation phase)</th><th colspan="5">Multiple doses (Cycle 1)</th><th>Subsequent treatment</th><th>End of study</th><th>Progression-free Survival Follow-up</th></tr>
<tr><th>（-28~0）</th><th>（-7~0）</th><th>D1</th><th>D2</th><th>D3-D6</th><th>D7</th><th>D1</th><th>D8（±1）</th><th>D15（±1）</th><th>D21（±1）</th><th>D28（±3）</th><th>D28（±3）</th><th>End of Treatment/ Withdrawal</th><th>Every 2 months (± 7 days)</th></tr>
<tr><td>Myocardial zymogram [16]</td><td></td><td>×</td><td></td><td></td><td></td><td></td><td></td><td></td><td></td><td></td><td></td><td></td><td></td><td></td></tr>
<tr><td>Electrocardiogram [17]</td><td></td><td>×</td><td>×</td><td>×</td><td></td><td>×</td><td>×</td><td>×</td><td>×</td><td>×</td><td>×</td><td>×</td><td>×</td><td></td></tr>
<tr><td>Echocardiography [18]</td><td>×</td><td></td><td></td><td></td><td></td><td></td><td></td><td></td><td></td><td></td><td>×</td><td>Even cycle</td><td>×</td><td></td></tr>
<tr><td>Tumor imaging [19]</td><td>×</td><td></td><td></td><td></td><td></td><td></td><td></td><td></td><td></td><td></td><td>×</td><td>Even cycle</td><td>×</td><td>×</td></tr>
<tr><td>Record AEs [20]</td><td colspan="13">×</td><td></td></tr>
<tr><td>Concomitant Medications/Treatments [21]</td><td colspan="13">×</td><td></td></tr>
<tr><td>Study drug administration [22]</td><td></td><td></td><td colspan="10">×</td><td></td><td></td></tr>
<tr><td>Death [23]</td><td></td><td></td><td colspan="11">×</td><td></td></tr>
</table>

Note: Various examinations and experimental procedures should be carried out according to the schedule of the study process, regardless of the duration of drug discontinuation. However, occasional changes within the window period of each examination may be allowed due to holidays, weekends, or other management reasons. The end-of-treatment/exit examination should be conducted within 7 days after the patient stops receiving the study drug treatment due to reasons other than death or loss to follow-up. If the patient withdraws from the study on the same day after completing the examination on D28, there is no need to repeat the end-of-treatment/exit examination.

[1] Demographic and general information: gender, date of birth, height, weight, and ethnicity.

[2] Tumor history/other medical history: Collect information on tumor diagnosis, date and results of initial pathological diagnosis, TNM staging, tumor recurrence/metastasis, biomarkers (if any), surgical history, radiotherapy history, and drug treatment history for anti-tumor therapy. Collect information on other medical history or accompanying diseases that the patient had within 6 months prior to enrollment.

[3] Vital sign assessments including temperature, respiration rate, pulse rate, and blood pressure will be conducted at baseline, 4 hours (±15 minutes), 12 hours (±15 minutes), and 24 hours (±15 minutes) after the first dose (cycle 0), and on day 7 (at an appropriate time) within 7 days prior to enrollment.

Multiple doses (Cycle 1): Day 1, 8 (±1), 15 (±1), and 21 (±1); Day 28 (±3) and every 28 (±3) days thereafter until the end of the study, with examinations performed at an appropriate time on the same day.

Before measuring blood pressure, smoking and drinking coffee should be avoided for at least 30 minutes. The patient should rest quietly for at least 3 minutes before measurement. Blood pressure should be measured while sitting, with the elbow at the same level as the heart. The same side of body should be used for each measurement.

[4] Physical examination: including skin and mucous membranes, lymph nodes, head and neck, chest, abdomen, spine, musculoskeletal system, nervous system, and other

areas. Within 7 days before enrollment, a single dose (cycle 0): on day 2 and day 7 after administration (both at an appropriate time on the same day).
Multiple doses (Cycle 1): Day 1, 8 (±1), 15 (±1), 21 (±1), and 28 (±3) with subsequent administrations on Day 28 (±3) of each cycle and at the end of the study. Examinations will be conducted on the same day at an appropriate time.

[5] Dose escalation stage PK blood sampling: Single-dose PK sampling time points: before administration (0 hour, blank value), 1 hour, 2 hours, 4 hours, 8 hours, 12 hours, 24 hours, 48 hours, 72 hours, 96 hours, 120 hours, 144 hours after administration. Multiple-dose PK sampling points: before administration on day 1, 8, 15, and 21 of the first cycle, and 1 hour, 2 hours, 4 hours, 8 hours, 12 hours, 24 hours, 48 hours, 72 hours, 96 hours, 120 hours, 144 hours, 168 hours after administration on day 21.
Refer to the above for PK blood sampling window.

[6] The dose escalation phase Cycle 1 PK intensive blood sampling: On Day 1 of the first cycle, blood samples should be collected before dosing (-1h), and at 1h (±5min), 2h (±5min), 4h (±10min), 8h (±10min), 12h (±15min), and 24h (±30min, to be collected before dosing on Day 2), before dosing (-1h) on Days 8, 15, and 21, and at 1h (±5min), 2h (±5min), 4h (±10min), 8h (±10min), 12h (±15min), 24h (±30min), 48h (±1h), 72h (±1h), 120h (±2h), and 168h (±2h) after dosing on Day 21. Cycle 1 PK sparse blood sampling: On Day 1 of the first cycle, blood samples should be collected before dosing (-1h) and at 4h (±10min) after dosing, before dosing (-1h) on Days 8, 15, and 21, at 4h (±10min) after dosing on Day 21, and at any time point on Day 28 of the visit.
PK intensive and sparse blood sampling points during the follow-up dosing period (Cycle 2~Cycle 6) in the extension phase: PK blood samples will be collected at any time point on Day 28 of each cycle during the visit.

[7] Biomarkers: Pathological examination reports will be collected before administration in this study to record the expression and mutation status of RET, FGFR, EGFR/VEGFR genes. The collection will be based on the actual situation of the patients and the results will not affect their enrollment.

[8] Complete Blood Count (CBC): Peripheral blood is collected to measure hemoglobin, red blood cell count, white blood cell count and differentiation (monocyte count, eosinophil count, basophil count, neutrophil count, lymphocyte count) and platelet count.
Within 7 days before enrollment, on Day 1 of the single-dose administration (Cycle 0), and on Day 7, blood routine examination should be performed prior to dosing (if the screening blood routine examination is completed within 3 days before dosing, it does not need to be repeated before dosing).
Multiple doses (cycle 1): on day 8 (±1), day 15 (±1), and day 21 (±1) (all administered prior to the aforementioned days); on day 28 (±3) of cycle 1, and subsequently on day 28 (±3) of each cycle and at the end of the study.

[9] Urinalysis: Test urine specific gravity, pH, glucose, protein, bilirubin, ketones, white blood cells, and red blood cells. If the semi-quantitative method shows protein >2+ (e.g. urine test paper), then a 24-hour urine protein quantitative test should be performed. Urinalysis should be conducted on the 7th day before enrollment, on the first day of single-dose administration (cycle 0), and before the first dose (if the urine routine test during the screening period is completed within 3 days before administration, there is no need to repeat the test before administration), on the 7th day, on the 15th (±1) and 28th (±3) days of multiple-dose administration (cycle 1), on the 28th (±3) day of each subsequent cycle, and at the end of the study, all at an appropriate time on the same day.

[10] Stool routine: examine the characteristics, color, white blood cells, red blood cells, and fecal occult blood. The examination will be conducted within 7 days before enrollment, on the 28th day (±3) of each cycle, and at the end of the study, all at an appropriate time on the same day.

[11] Blood biochemistry: Total protein, albumin, globulin, blood glucose, total cholesterol, low-density lipoprotein, high-density lipoprotein, triglycerides, urea, creatinine, alkaline phosphatase, total bilirubin, direct or indirect bilirubin, AST, ALT, calcium, phosphorus, magnesium, potassium, sodium, chloride, uric acid. Within 7 days before enrollment, blood biochemistry tests will be conducted on the first day of single-dose administration (cycle 0, if the blood biochemistry test during the screening period is completed within 3 days before administration, no need to repeat the test before administration), on day 7 of cycle 0, on day 15 (±1) and day 28 (±3) of multiple-dose administration (cycle 1, all tests will be conducted before administration on the same day), and on day 28 (±3) of each subsequent cycle and at the end of the study.

[12] Coagulation function: PT, APTT, INR will be tested before enrollment, on day 28 (±3) of each cycle, and at the end of the study.

[13] Thyroid function: including TSH, FT3, FT4; to be measured within 7 days before enrollment, on day 28 (±3) of each cycle, and at the end of the study.

[14] Serological testing: including HBV, HCV, HIV, to be conducted within 28 days prior to enrollment.

[15] Pregnancy check: Only eligible for women of childbearing age, to be conducted within 7 days before enrollment and at the end of the study.

[16] Cardiac enzyme profile test: Measures creatine kinase and lactate dehydrogenase levels; to be conducted once within 7 days prior to enrollment.

[17] 12-lead electrocardiogram: to detect heart rate, QT, QTc, QRS duration, and P-R interval; screening period, before single-dose administration (cycle 0) and 4 hours (±15 minutes), 12 hours (±15 minutes), 24 hours (±30 minutes), and 7 days (at an appropriate time on that day) after administration; before the first dose during multiple-dose administration, 4 hours (±1 hour) after administration on day 1, and 4 hours (±1 hour) after administration on days 8 (±1), 15 (±1), 21 (±1), and 28 (±3); check once at the end of each cycle on day 28 (±3) and at the end of treatment, and the check should be performed at an appropriate time on that day. If the patient experiences symptoms such as chest pain or palpitations, additional checks may be performed.

[18] Echocardiography: performed within 28 days before enrollment, on day 28 (±3) of multiple-dose administration (cycle 1), and on day 28 (±3) of every even treatment cycle thereafter. If symptoms such as chest pain or palpitations occur during the study period, additional tests may be performed as appropriate.

[19] Imaging examinations: including CT or MRI of the chest, abdomen, pelvis, and other areas deemed necessary by the investigator. The screening period for tumor baseline assessment can be extended to within 4 weeks before treatment. CT/MRI scan results obtained before signing the informed consent form can be used for screening tumor evaluation as long as they meet the requirements. Brain CT/MRI examination is required when central nervous system metastasis is clinically suspected, and bone scan examination is required when bone metastasis is clinically suspected. The allowed time window for tumor imaging examination is ±7 days. Evaluation time: the first evaluation is performed on day 28 of the first cycle of treatment, and then once every two cycles of treatment until the end of treatment or the patient withdraws. If the first evaluation is CR or PR, confirmation is required after 4 weeks. The confirmed tumor evaluation cannot change the fixed two-cycle examination time points. When treatment ends or the patient withdraws (if no tumor evaluation has been performed within the previous 4 weeks), follow-up for progression-free survival is conducted every 2 months (±7 days) until disease progression, death, withdrawal of informed consent, or the initiation of new anti-tumor treatment, whichever occurs first.

[20] AEs: AEs will be recorded from the time of signing the informed consent form until 4 weeks after the last dose of the study drug, and followed up until the AEs have recovered (baseline level or complete recovery) or reached clinical stability.

[21] Recording concomitant medications: Record concomitant medications and treatments taken during the 28 days prior to administration and during the study period. If a patient discontinues the experimental treatment, only concomitant medications and treatments used for new or unresolved AEs related to the experimental treatment should be recorded.

[22] Drug administration during the study: Record the administration time and dose during the study, collect specific information related to adjusted administration (including adjusted administration time, adjusted dose level, reasons, etc.), and record it in the eCRF.

[23] Death: Collect all death events of patients from the first administration of the drug to the end of the safety follow-up period (28 days after the last administration of the drug), including the time of death, cause of death, and the relationship between death and the drug. After the end of the safety follow-up period, death events will no longer be actively collected, but if a death event occurs and the investigator considers that there is a reasonable causal relationship with study drug, it should also be collected.

Appendix II ECOG performance score

Activity score	Description status
0	Fully active, able to carry on all pre-disease performance without restriction. Asymptomatic, fully ambulatory, and able to engage in unrestricted activities.
1	Restricted in physically strenuous activity but ambulatory and able to carry out work of a light or sedentary nature, e.g., light housework, office work
2	Ambulatory and capable of all selfcare but unable to carry out any work activities; up and about more than 50% of waking hours
3	Capable of only limited selfcare; confined to bed or chair more than 50% of waking hours
4	Completely disabled; cannot carry on any selfcare; totally confined to bed or chair
5	Dead

Appendix III Response Evaluation Criteria in Solid Tumors

Response Evaluation Criteria in Solid Tumors Version 1.1 (Excerpts)

（New Response Evaluation Criteria in Solid Tumors： Revised RECIST Version 1.1）

Note: This attachment is for internal translation reference only. Please refer to the English version for actual use.

1. Measurability of tumour at baseline

1.1. Definitions

At baseline, the tumor lesions/lymph nodes will be classified as measurable or non-measurable as follows:

1.1.1. Measurable

Tumour lesions: Must be accurately measured in at least one dimension (longest diameter in the plane of measurement is to be recorded) with a minimum size of:

- 10 mm by CT scan (CT scan slice thickness no greater than 5 mm).
- 10 mm caliper measurement by clinical exam (lesions that cannot be accurately measured with calipers should be recorded as non-measurable).
- 20 mm by chest X-ray.

Malignant lymph nodes: To be considered pathologically enlarged and measurable, a lymph node must be ≥15 mm in the short axis when assessed by CT scan (CT scan slice thickness recommended to be no greater than 5 mm). At baseline and in follow-up, only the short axis will be measured and followed.

1.1.2. Non-measurable

All other lesions, including small lesions (longest diameter <10 mm or pathological lymph nodes with ≥10 to <15 mm short axis), as well as truly non-measurable lesions. Lesions considered truly non-measurable include: leptomeningeal disease, ascites, pleural or pericardial effusion, inflammatory breast disease, lymphangitic involvement of skin or lung, abdominal masses/abdominal organomegaly identified by a physical exam that is not measurable by reproducible imaging techniques.

1.1.3. Special considerations regarding lesion measurability

Bone lesions, cystic lesions, and lesions previously treated with local therapy require particular comments:

Bone lesions:

- Bone scans, PET scans or plain films are not considered adequate imaging techniques to measure bone lesions. However, these techniques can be used to confirm the presence or disappearance of bone lesions.
- Lytic bone lesions or mixed lytic-blastic lesions with identifiable soft tissue components that can be evaluated by cross-sectional imaging techniques such as CT or MRI can be considered as measurable lesions if the soft tissue component meets the definition of measurability described above.
- Blastic bone lesions are non-measurable.

Cystic lesions:

- Lesions that meet the criteria for radiographically defined simple cysts should not be considered as malignant lesions (neither measurable nor non-measurable) since they are, by definition, simple cysts.
- 'Cystic lesions' thought to represent cystic metastases can be considered as measurable lesions, if they meet the definition of measurability described above. However, if noncystic lesions are present in the same patient, these are preferred for selection as target lesions.

Lesions with prior local treatment:

- Tumour lesions situated in a previously irradiated area, or in an area patiented to other loco-regional therapy, are usually not considered measurable unless there has been demonstrated progression in the lesion. Study protocols should detail the conditions under which such lesions would be considered measurable.

1.2. Specifications by methods of measurements

1.2.1. Measurement of Lesions

All measurements should be recorded in metric notation, using calipers if clinically assessed. All baseline evaluations should be performed as close as possible to the treatment start and never more than 4 weeks before the beginning of the treatment.

1.2.2. Method of assessment

The same method of assessment and the same technique should be used to characterize each identified and reported lesion at baseline and during follow-up. Imaging-based evaluation should always be done rather than clinical examination unless the lesion(s) being followed cannot be imaged but are assessable by clinical exam.

Clinical lesions: Clinical lesions will only be considered measurable when they are superficial and ≥10 mm in diameter as assessed using calipers (e.g. skin nodules). For the case of skin lesions, documentation by colour photography, including a ruler to estimate the size of the lesion, is suggested. As noted above, when lesions can be evaluated by both clinical exam and imaging, imaging evaluation should be undertaken since it is more objective and may also be reviewed at the end of the study.

Chest X-ray: Chest CT is preferred over chest X-ray, particularly when progression is an important endpoint, since CT is more sensitive than X-ray, particularly in identifying new lesions. However, lesions on chest X-ray may be considered measurable if they are clearly defined and surrounded by aerated lungs.

CT, MRI: CT is the best currently available and reproducible method to measure lesions selected for response assessment. This guideline has defined the measurability of lesions on CT scans based on the assumption that CT slice thickness is 5 mm or less. When CT scans have a slice thickness greater than 5 mm, the minimum size for a measurable lesion should be twice the slice thickness. MRI is also acceptable in certain situations (e.g. for body scans).

Ultrasound: Ultrasound is not useful in the assessment of lesion size and should not be used as a method of measurement. Ultrasound examinations cannot be reproduced in their entirety for independent review at a later date and because they are operator-dependent, it cannot be guaranteed that the same technique and measurements will be taken from one assessment to the next. If new lesions are identified by ultrasound in the course of the study, confirmation by CT or MRI is advised. If there is concern about radiation exposure at CT, MRI may be used instead of CT in selected instances.

Endoscopy, laparoscopy: The utilisation of these techniques for objective tumour evaluation is not advised. However, they can be useful to confirm complete pathological response when biopsies are obtained or to determine relapse in trials where recurrence following complete response or surgical resection is an endpoint.

Tumour markers: Tumour markers alone cannot be used to assess objective tumour response. If markers are initially above the upper normal limit, however, they must normalise for a patient to be considered in complete response. Because tumour markers are disease-specific, instructions for their measurement should be incorporated into protocols on a disease-specific basis. Specific guidelines for both CA-125 response (in recurrent ovarian cancer) and PSA response (in recurrent prostate cancer), have been published. In addition, the Gynecologic Cancer Intergroup has developed CA125 progression criteria which are to be integrated with objective tumour assessment for use in first-line trials in ovarian cancer.

Cytology, histology: These techniques can be used to differentiate between PR and CR in rare cases if required by protocol (for example, residual lesions in tumour types such as germ cell tumours, where known residual benign tumours can remain). When effusions are known to be a potential adverse effect of treatment (e.g. with certain taxane compounds or angiogenesis inhibitors), the cytological confirmation of the neoplastic origin of any effusion that appears or worsens during treatment can be considered if the measurable tumour has met criteria for response or stable disease in order to differentiate between response (or stable disease) and progressive disease.

2. Tumor Response Evaluation

2.1. Assessment of overall tumour burden and measurable disease

To assess objective response or future progression, it is necessary to estimate the overall tumour burden at baseline and use this as a comparator for subsequent measurements. Only patients with measurable disease at baseline should be included in protocols where objective tumour response is the primary endpoint. Measurable disease is defined by the presence of at least one measurable lesion. In studies where the primary endpoint is tumour progression (either time to progression or proportion with progression at a fixed date), the protocol must specify if entry is restricted to those with measurable disease or whether patients having non-measurable disease only are also eligible.

2.2. Baseline documentation of 'target' and 'non-target' lesions

When more than one measurable lesion is present at baseline, all lesions up to a maximum of five lesions total (and a maximum of two lesions per organ) representative of all involved organs should be identified as target lesions and will be recorded and measured at baseline (this means in instances where patients have only one or two organ sites involved a maximum of two and four lesions respectively will be recorded). For evidence to support the selection of only five target lesions, see analyses on a large prospective database in the article by Bogaerts et al. Target lesions should be selected on the basis of their size (lesions with the longest diameter), be representative of all involved organs, but in addition, should be those that lend themselves to reproducible repeated measurements. It may be the case that, on occasion, the largest lesion does not lend itself to reproducible measurement, in which circumstance, the next largest lesion which can be measured reproducibly should be selected.

Lymph nodes merit special mention since they are normal anatomical structures that may be visible by imaging even if not involved by tumour. As noted in Section 3, pathological nodes that are defined as measurable and may be identified as target lesions must meet the criterion of a short axis of ≥15 mm by CT scan. Only the short axis of these nodes will contribute to the baseline sum. The short axis of the node is the diameter normally used by radiologists to judge

if a node is involved in a solid tumour. Nodal size is normally reported as two dimensions in the plane in which the image is obtained (for CT scan, this is almost always the axial plane; for MRI the plane of acquisition may be axial, sagittal or coronal). The smaller of these measures is the short axis. For example, an abdominal node that is reported as being 20 mm×30 mm has a short axis of 20 mm and qualifies as a malignant, measurable node. In this example, 20 mm should be recorded as the node measurement. All other pathological nodes (those with a short axis ≥10 mm but <15 mm) should be considered non-target lesions. Nodes that have a short axis <10 mm are considered non-pathological and should not be recorded or followed.

A sum of the diameters (longest for non-nodal lesions, short axis for nodal lesions) for all target lesions will be calculated and reported as the baseline sum diameters. If lymph nodes are to be included in the sum, then as noted above, only the short axis is added to the sum. The baseline sum diameters will be used as a reference to further characterise any objective tumour regression in the measurable dimension of the disease.

All other lesions (or sites of disease), including pathological lymph nodes, should be identified as non-target lesions and should also be recorded at baseline. Measurements are not required and these lesions should be followed as 'present', 'absent', or in rare cases 'unequivocal progression' (more details to follow). In addition, it is possible to record multiple nontarget lesions involving the same organ as a single item on the case record form (e.g. 'multiple enlarged pelvic lymph nodes' or 'multiple liver metastases').

2.3. Response criteria

This section provides the definitions of the criteria used to determine objective tumour response for target lesions.

2.3.1. Evaluation of target lesions

Complete Response (CR): Disappearance of all target lesions. Any pathological lymph nodes (whether target or non-target) must have a reduction in short axis to <10 mm.

Partial Response (PR): At least a 30% decrease in the sum of diameters of target lesions, taking as reference the baseline sum diameters.

Progressive Disease (PD): At least a 20% increase in the sum of diameters of target lesions, taking as reference the smallest sum in the study (this includes the baseline sum if that is the smallest in the study). In addition to the relative increase of 20%, the sum must also demonstrate an absolute increase of at least 5 mm. (Note: the appearance of one or more new lesions is also considered progression).

Stable Disease (SD): Neither sufficient shrinkage to qualify for PR nor sufficient increase to qualify for PD, taking as reference the smallest sum diameters while on the study.

2.3.2. Special notes on the assessment of target lesions

Lymph nodes. Lymph nodes identified as target lesions should always have the actual short axis measurement recorded (measured in the same anatomical plane as the baseline examination), even if the nodes regress to below 10 mm on study. This means that when lymph nodes are included as target lesions, the 'sum' of lesions may not be zero even if complete response criteria are met, since a normal lymph node is defined as having a short axis of <10 mm. Case report forms or other data collection methods may therefore be designed to have target nodal lesions recorded in a separate section where, in order to qualify for CR, each node must achieve a short axis <10 mm. For PR, SD and PD, the actual short-axis measurement of the nodes is to be included in the sum of target lesions.

Target lesions that become 'too small to measure'. While on study, all lesions (nodal and non-

nodal) recorded at baseline should have their actual measurements recorded at each subsequent evaluation, even when very small (e.g. 2 mm). However, sometimes lesions or lymph nodes which are recorded as target lesions at baseline become so faint on CT scan that the radiologist may not feel comfortable assigning an exact measure and may report them as being 'too small to measure'. When this occurs, it is important that a value be recorded on the case report form. If it is the opinion of the radiologist that the lesion has likely disappeared, the measurement should be recorded as 0 mm. If the lesion is believed to be present and is faintly seen but too small to measure, a default value of 5 mm should be assigned. (Note: It is less likely that this rule will be used for lymph nodes since they usually have a definable size when normal and are frequently surrounded by fat such as in the retroperitoneum; however, if a lymph node is believed to be present and is faintly seen but too small to measure, a default value of 5 mm should be assigned in this circumstance as well). This default value is derived from the 5 mm CT slice thickness (but should not be changed with varying CT slice thickness). The measurement of these lesions is potentially non-reproducible; therefore, providing this default value will prevent false responses or progressions based upon measurement error. To reiterate, however, if the radiologist is able to provide an actual measure, that should be recorded, even if it is below 5 mm.

Lesions that split or coalesce on treatment. When non-nodal lesions 'fragment', the longest diameters of the fragmented portions should be added together to calculate the target lesion sum. Similarly, as lesions coalesce, a plane between them may be maintained that would aid in obtaining maximal diameter measurements of each individual lesion. If the lesions have truly coalesced such that they are no longer separable, the vector of the longest diameter in this instance should be the maximal longest diameter for the 'coalesced lesion'.

2.3.3. Evaluation of non-target lesions

This section provides the definitions of the criteria used to determine the tumour response for the group of non-target lesions. While some non-target lesions may actually be measurable, they need not be measured and instead should be assessed only qualitatively at the time points specified in the protocol.

Complete Response (CR): Disappearance of all non-target lesions and normalisation of tumour marker level. All lymph nodes must be non-pathological in size (<10 mm short axis).

Non-CR/Non-PD: Persistence of one or more non-target lesion(s) and/or maintenance of tumour marker level above the normal limits.

Progressive Disease (PD): Unequivocal progression of existing non-target lesions. (Note: the appearance of one or more new lesions is also considered progression).

2.3.4. Special notes on assessment of progression of nontarget disease

The concept of progression of non-target disease requires additional explanation as follows: When the patient also has measurable disease. In this setting, to achieve 'unequivocal progression' on the basis of the non-target disease, there must be an overall level of substantial worsening in non-target disease such that, even in presence of SD or PR in target disease, the overall tumour burden has increased sufficiently to merit discontinuation of therapy. A modest 'increase' in the size of one or more non-target lesions is usually not sufficient to quality for unequivocal progression status. The designation of overall progression solely on the basis of change in non-target disease in the face of SD or PR of target disease will therefore be extremely rare.

When the patient has only non-measurable disease.This circumstance arises in some phase III trials when it is not a criterion of study entry to have measurable disease. The same general

concepts apply here as noted above, however, in this instance there is no measurable disease assessment to factor into the interpretation of an increase in non-measurable disease burden. Because worsening in non-target disease cannot be easily quantified (by definition: if all lesions are truly non-measurable) a useful test that can be applied when assessing patients for unequivocal progression is to consider if the increase in overall disease burden based on the change in non-measurable disease is comparable in magnitude to the increase that would be required to declare PD for measurable disease: i.e. an increase in tumour burden representing an additional 73% increase in 'volume' (which is equivalent to a 20% increase diameter in a measurable lesion). Examples include an increase in a pleural effusion from 'trace' to 'large', an increase in lymphangitic disease from localised to widespread, or may be described in protocols as 'sufficient to require a change in therapy'. If 'unequivocal progression' is seen, the patient should be considered to have had overall PD at that point. While it would be ideal to have objective criteria to apply to non-measurable disease, the very nature of that disease makes it impossible to do so, therefore the increase must be substantial.

2.3.5. New Lesions

The appearance of new malignant lesions denotes disease progression; therefore, some comments on the detection of new lesions are important. There are no specific criteria for the identification of new radiographic lesions; however, the finding of a new lesion should be unequivocal: i.e. not attributable to differences in scanning technique, change in imaging modality or findings thought to represent something other than tumour (for example, some 'new' bone lesions may be simply healing or flare of pre-existing lesions). This is particularly important when the patient's baseline lesions show partial or complete response. For example, necrosis of a liver lesion may be reported on a CT scan report as a 'new' cystic lesion, which it is not.

A lesion identified on a follow-up study in an anatomical location that was not scanned at baseline is considered a new lesion and will indicate disease progression. An example of this is the patient who has visceral disease at baseline and while on study has a CT or MRI brain ordered which reveals metastases. The patient's brain metastases are considered to be evidence of PD even if he/she did not have brain imaging at baseline.

If a new lesion is equivocal, for example, because of its small size, continued therapy and follow-up evaluation will clarify if it represents a truly new disease. If repeat scans confirm there is definitely a new lesion, then progression should be declared using the date of the initial scan. While FDG-PET response assessments need additional study, it is sometimes reasonable to incorporate the use of FDG-PET scanning to complement CT scanning in assessment of progression (particularly possible 'new' disease). New lesions on the basis of FDG-PET imaging can be identified according to the following algorithm:

a. Negative FDG-PET at baseline, with a positive FDG-PET at follow-up, is a sign of PD based on a new lesion.

b. No FDG-PET at baseline and a positive FDG-PET at follow-up: If the positive FDG-PET at follow-up corresponds to a new site of disease confirmed by CT, this is PD. If the positive FDG-PET at follow-up is not confirmed as a new site of disease on CT, additional follow-up CT scans are needed to determine if there is true progression occurring at that site (if so, the date of PD will be the date of the initial abnormal FDG-PET scan). If the positive FDG-PET at follow-up corresponds to a pre-existing site of disease on CT that is not progressing on the basis of the anatomic images, this is not PD.

2.4. Evaluation of best overall response

The best overall response is the best response recorded from the start of the study treatment

until the end of treatment taking into account any requirement for confirmation. On occasion, a response may not be documented until after the end of therapy, so protocols should be clear if post-treatment assessments are to be considered in determination of best overall response. Protocols must specify how any new therapy introduced before progression will affect best response designation. The patient's best overall response assignment will depend on the findings of both target and non-target disease and will also take into consideration the appearance of new lesions. Furthermore, depending on the nature of the study and the protocol requirements, it may also require confirmatory measurement (see Section 4.6). Specifically, in non-randomised trials where response is the primary endpoint, confirmation of PR or CR is needed to deem either one the 'best overall response'. This is described further below.

2.4.1. Time point response

It is assumed that at each protocol-specified time point, a response assessment occurs. Table 1 on the next page provides a summary of the overall response status calculation at each time point for patients who have measurable disease at baseline.

When patients have non-measurable (therefore non-target) disease only, Table 2 is to be used.

2.4.2. Missing assessments and inevaluable designation

When no imaging/measurement is done at all at a particular time point, the patient is not evaluable (NE) at that time point. If only a subset of lesion measurements are made at an assessment, usually the case is also considered NE at that time point, unless a convincing argument can be made that the contribution of the individual missing lesion(s) would not change the assigned time point response. This would be most likely to happen in the case of PD. For example, if a patient had a baseline sum of 50 mm with three measured lesions and at follow-up only two lesions were assessed, but those gave a sum of 80 mm, the patient will have achieved PD status, regardless of the contribution of the missing lesion.

2.4.3. Best overall response: all time points

The best overall response is determined once all the data for the patient is known. Best response determination in trials where confirmation of complete or partial response IS NOT required: Best response in these trials is defined as the best response across all time points (for example, a patient who has SD at first assessment, PR at second assessment, and PD on last assessment has a best overall response of PR). When SD is believed to be best response, it must also meet the protocol-specified minimum time from baseline. If the minimum time is not met when SD is otherwise the best time point response, the patient's best response depends on the subsequent assessments. For example, a patient who has SD at first assessment, PD at second and does not meet the minimum duration for SD, will have a best response of PD. The same patient lost to follow-up after the first SD assessment would be considered inevaluable. Best response determination in trials where confirmation of complete or partial response IS required: Complete or partial responses may be claimed only if the criteria for each are met at a subsequent time point as specified in the protocol (generally 4 weeks later). In this circumstance, the best overall response can be interpreted as in Table 3.

2.4.4. Special notes on response assessment

When nodal disease is included in the sum of target lesions and the nodes decrease to 'normal' size (<10 mm), they may still have a measurement reported on scans. This measurement should be recorded even though the nodes are normal in order not to overstate progression should it be based on increase in size of the nodes. As noted earlier, this means that patients with CR may not have a total sum of 'zero' on the case report form (CRF).

In trials where confirmation of response is required, repeated 'NE' time point assessments may

complicate best response determination. The analysis plan for the trial must address how missing data/assessments will be addressed in determination of response and progression. For example, in most trials, it is reasonable to consider a patient with time point responses of PR-NE-PR as a confirmed response.

Patients with a global deterioration of health status requiring discontinuation of treatment without objective evidence of disease progression at that time should be reported as ‘symptomatic deterioration’. Every effort should be made to document objective progression even after discontinuation of treatment. Symptomatic deterioration is not a descriptor of an objective response: it is a reason for stopping study therapy. The objective response status of such patients is to be determined by evaluation of target and non-target disease as shown in Tables 1–3.

Conditions that define ‘early progression, early death and inevaluability’ are study specific and should be clearly described in each protocol (depending on treatment duration, treatment periodicity).

In some circumstances, it may be difficult to distinguish residual disease from normal tissue. When the evaluation of complete response depends upon this determination, it is recommended that the residual lesion be investigated (fine needle aspirate/biopsy) before assigning a status of complete response. FDG-PET may be used to upgrade a response to a CR in a manner similar to a biopsy in cases where a residual radiographic abnormality is thought to represent fibrosis or scarring. The use of FDG-PET in this circumstance should be prospectively described in the protocol and supported by disease-specific medical literature for the indication. However, it must be acknowledged that both approaches may lead to false positive CR due to limitations of FDG-PET and biopsy resolution/sensitivity.

For equivocal findings of progression (e.g. very small and uncertain new lesions; cystic changes or necrosis in existing lesions), treatment may continue until the next scheduled assessment. If at the next scheduled assessment, progression is confirmed, the date of progression should be the earlier date when progression was suspected.

Table 1: Time point response: patients with target (+/– non-target) disease.

Target Lesions	Non-Target Lesions	New Lesions	Overall response
CR	CR	No	CR
CR	Non-CR/Non-PD	No	PR
CR	Not evaluable	No	PR
PR	Non-progression or incomplete assessment	No	PR
SD	Non-progression or incomplete assessment	No	SD
Not fully evaluated	Non-progression	No	NE
PD	Any situation	Yes or No	PD
Any situation	PD	Yes or No	PD
Any situation	Any situation	Yes	PD
CR = complete	PR = partial response	SD = stable disease	PD = progressive

response			disease<br>NE = inevaluable

In SD cases, at least one follow-up measurement after enrollment must meet the SD criteria, and the follow-up interval should be at least 6-8 weeks after enrollment.

Table 2 Time point response: patients with non-target disease only.

**Non-Target Lesions**	**New Lesions**	**Overall response**
CR	No	CR
Non-CR or non-PD	No	Non-CR or Non-PD
Not all evaluated	No	NE
Unequivocal PD	Yes or No	PD
Any	Yes	PD

Note: CR = complete response, PD = progressive disease, and NE = inevaluable. a 'Non-CR/non-PD' is preferred over 'stable disease' for non-target disease since SD is increasingly used as endpoint for assessment of efficacy in some trials so to assign this category when no lesions can be measured is not advised.

Table 3 – Best overall response when confirmation of CR and PR required

**Overall response first time point**	**Overall response subsequent time points**	**Best overall response**
CR	CR	CR
CR	PR	SD, PD or PR[a]
CR	SD	SD provided minimum criteria for SD duration met, otherwise, PD.
CR	PD	SD provided minimum criteria for SD duration met, otherwise, PD.
CR	NE	SD provided minimum criteria for SD duration met, otherwise, NE.
PR	CR	PR
PR	PR	PR
PR	SD	SD
PR	PD	SD provided minimum criteria for SD duration met, otherwise, PD.
PR	NE	SD provided minimum criteria for SD duration met, otherwise, PD.
NE	NE	NE

Note: CR = complete response, PR = partial response, SD = stable disease, PD = progressive disease, and NE = inevaluable. a If a CR is truly met at first time point, then any disease seen at a subsequent time point, even disease meeting PR criteria relative to baseline, makes the

disease PD at that point (since disease must have reappeared after CR). Best response would depend on whether minimum duration for SD was met. However, sometimes 'CR' may be claimed when subsequent scans suggest small lesions were likely still present and in fact the patient had PR, not CR at the first time point. Under these circumstances, the original CR should be changed to PR and the best response is PR.

2.5. Frequency of tumour re-evaluation

Frequency of tumour re-evaluation while on treatment should be protocol specific and adapted to the type and schedule of treatment. However, in the context of phase II studies where the beneficial effect of therapy is not known, follow-up every 6–8 weeks (timed to coincide with the end of a cycle) is reasonable. Smaller or greater time intervals than these could be justified in specific regimens or circumstances. The protocol should specify which organ sites are to be evaluated at baseline (usually those most likely to be involved with metastatic disease for the tumour type under study) and how often evaluations are repeated. Normally, all target and non-target sites are evaluated at each assessment. In selected circumstances certain non-target organs may be evaluated less frequently. For example, bone scans may need to be repeated only when a complete response is identified in the target disease or when progression in bone is suspected.

After the end of the treatment, the need for repetitive tumour evaluations depends on whether the trial has as a goal the response rate or the time to an event (progression/death). If 'time to an event' (e.g. time to progression, disease-free survival, progression-free survival) is the main endpoint of the study, then a routine scheduled re-evaluation of protocol-specified sites of disease is warranted. In randomised comparative trials in particular, the scheduled assessments should be performed as identified on a calendar schedule (for example: every 6–8 weeks on treatment or every 3–4 months after treatment) and should not be affected by delays in therapy, drug holidays or any other events that might lead to an imbalance in a treatment arm in the timing of disease assessment.

2.6. Confirmatory measurement/duration of response

2.6.1. Confirmation

In non-randomised trials where response is the primary endpoint, confirmation of PR and CR is required to ensure responses identified are not the result of measurement error. This will also permit appropriate interpretation of results in the context of historical data where response has traditionally required confirmation in such trials. However, in all other circumstances, i.e. in randomised trials (phase II or III) or studies where stable disease or progression is the primary endpoints, confirmation of response is not required since it will not add value to the interpretation of trial results. However, elimination of the requirement for response confirmation may increase the importance of a central review to protect against bias, in particular in studies that are not blinded.

In the case of SD, measurements must have met the SD criteria at least once after study entry at a minimum interval (in general not less than 6–8 weeks) that is defined in the study protocol.

2.6.2. Duration of overall response

The duration of overall response is measured from the time measurement criteria are first met for CR/PR (whichever is first recorded) until the first date that recurrent or progressive disease is objectively documented (taking as reference for progressive disease the smallest measurements recorded on study).

The duration of overall complete response is measured from the time measurement criteria are first met for CR until the first date that recurrent disease is objectively documented.

2.6.3. Duration of stable disease

Stable disease is measured from the start of the treatment (in randomised trials, from date of randomisation) until the criteria for progression are met, taking as reference the smallest sum on study (if the baseline sum is the smallest, this is the reference for calculation of PD).

The clinical relevance of the duration of stable disease varies in different studies and diseases. If the proportion of patients achieving stable disease for a minimum period of time is an endpoint of importance in a particular trial, the protocol should specify the minimal time interval required between two measurements for determination of stable disease.

Note: The duration of response and stable disease as well as the progression-free survival are influenced by the frequency of follow-up after baseline evaluation. It is not in the scope of this guideline to define a standard follow-up frequency. The frequency should take into account many parameters, including disease types and stages, treatment periodicity and standard practice. However, these limitations of the precision of the measured endpoint should be taken into account if comparisons between trials are to be made.

2.7. Progression-free survival/proportion progression-free

2.7.1. Phase II trials

This guideline is focused primarily on the use of objective response endpoints for phase II trials. In some circumstances, ‘response rate’ may not be the optimal method to assess the potential anticancer activity of new agents/regimens. In such cases ‘progression-free survival’ (PFS) or the ‘proportion progression-free’ at landmark time points, might be considered appropriate alternatives to provide an initial signal of biologic effect of new agents. It is clear, however, that in an uncontrolled trial, these measures are subject to criticism since an apparently promising observation may be related to biological factors such as patient selection and not the impact of the intervention. Thus, phase II trials utilising these endpoints are best designed with a randomised control. Exceptions may exist where the behaviour patterns of certain cancers are so consistent (and usually consistently poor), that a non-randomised trial is justifiable. However, in these cases, it will be essential to document with care the basis for estimating the expected PFS or proportion progression-free in the absence of a treatment effect.
