# Supplementary material for: Safety, pharmacokinetics and efficacy of HA121-28 in patients with advanced solid tumors and RET fusion-positive non-small-cell lung cancer: a multicenter, open-label, single-arm phase 1/2 trial
Source: Signal Transduct Target Ther. 2025 Feb 28;10:62. doi: 10.1038/s41392-025-02155-5 (PMC11868595; doi:10.1038/s41392-025-02155-5)
Supplement: Supplementary file 2 — Supplementary materials [file 41392_2025_2155_MOESM2_ESM.docx]

Supplementary Materials for

**Safety, pharmacokinetics and efficacy of HA121-28 in patients with advanced solid tumors and RET fusion-positive non-small-cell lung cancer: a multicenter, open-label, single-arm phase 1/2 trial**

Dan-Yun Ruan, Wenwen Huang, Yongsheng Li, Yanqiu Zhao, Yehui Shi, Yuming Jia, Shundong Cang, Wei Zhang, Jianhua Shi, Jun Chen, Jie Lin, Yunpeng Liu, Jianming Xu, Weiwei Ouyang, Jian Fang, Wu Zhuang, Caigang Liu, Qing Bu, Manxiang Li, Xiangjiao Meng, Meili Sun, Nong Yang, Xiaorong Dong, Yueyin Pan, Xingya Li, Xiujuan Qu, Tongmei Zhang, Xianglin Yuan, Sheng Hu, Wei Guo, Yalun Li, Shengqing Li, Dongying Liu, Feixue Song, Liping Tan, Yan Yu, Xinmin Yu, Aimin Zang, Chang Sun, Qian Zhang, Kai Zou, Mo Dan, Rui-Hua Xu, Hongyun Zhao

Correspondence to: [xurh@sysucc.org.cn](mailto:xurh@sysucc.org.cn), zhaohy@sysucc.org.cn

**This PDF file includes:**

Figures. S1 to S5

Tables S1 to S17


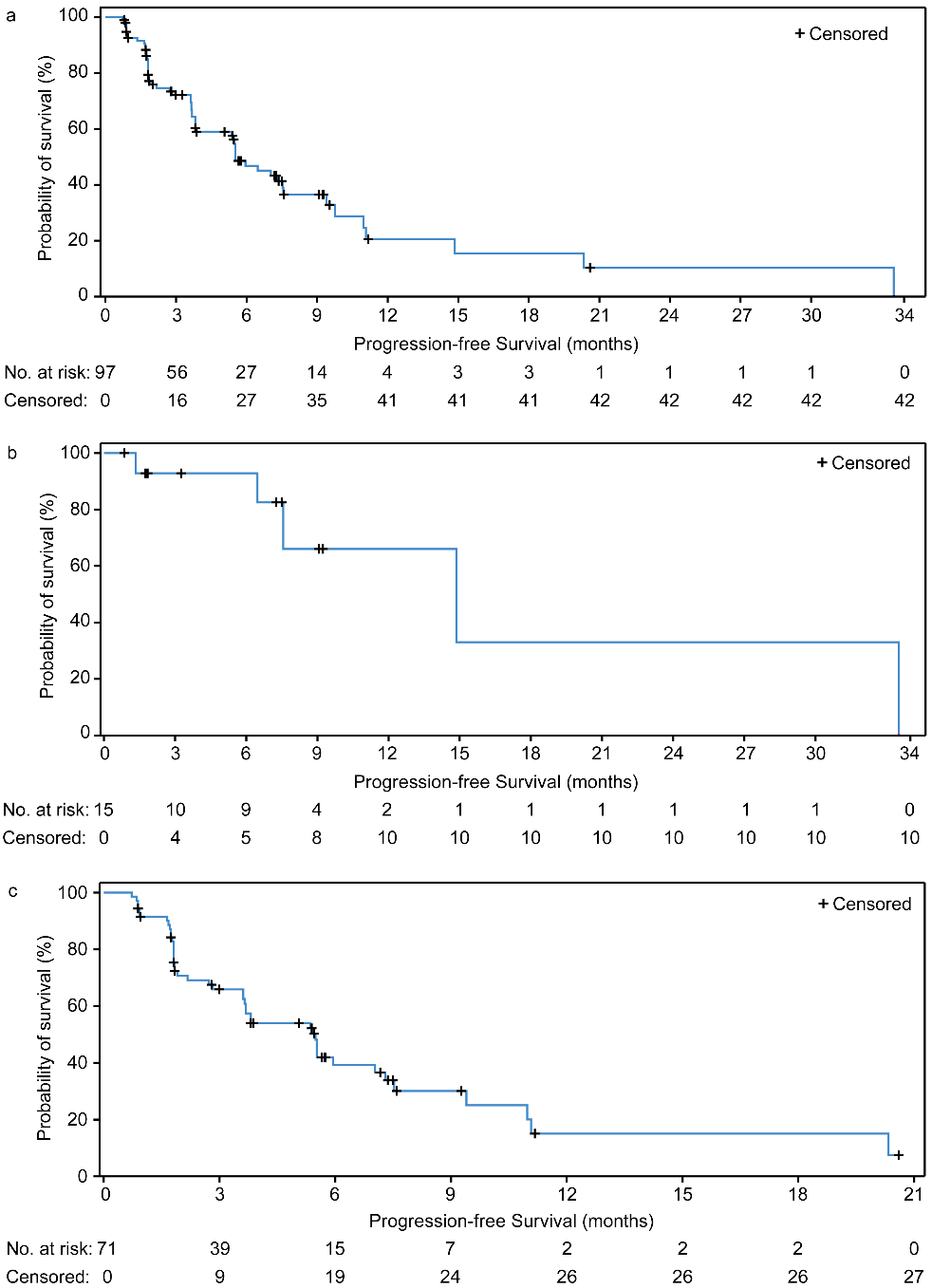


Figure. S1.

**Kaplan-Meier curve of progression-free survival.**

(a) Kaplan-Meier curve of progression-free survival in patients with RET-fusion positive NSCLC. (b) Kaplan-Meier curve of progression-free survival in NSCLC patients with a CCDC6-RET fusion. (c) Kaplan-Meier curve of progression-free survival in NSCLC patients with a KIF5B-RET fusion. Progression-free survival is defined as the time from the start of treatment to disease progression or death from any cause. CCDC6, coiled-coil domain-containing protein 6; KIF5B, kinesin family member 5B; NSCLC, non-small-cell lung cancer; RET, rearranged during transfection.


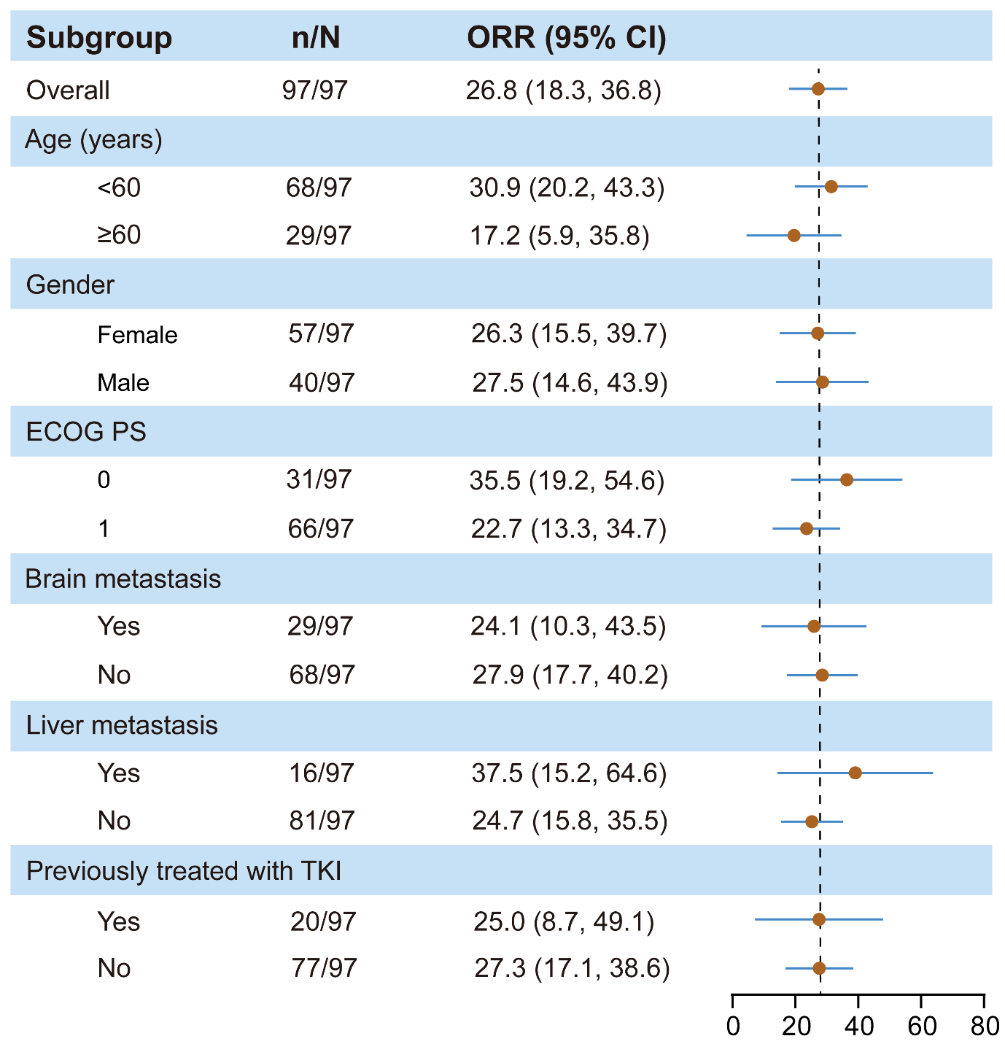


Figure. S2.

**Subgroups analysis of objective response rate (ORR) according to baseline characteristics.**

The subgroup analyses of ORR including age (≥60, <60), gender (male or female), ECOG PS (0 or 1), brain or liver metastasis (yes or no), and prior treatment with tyrosine kinases inhibitors (TKIs) (yes or no).


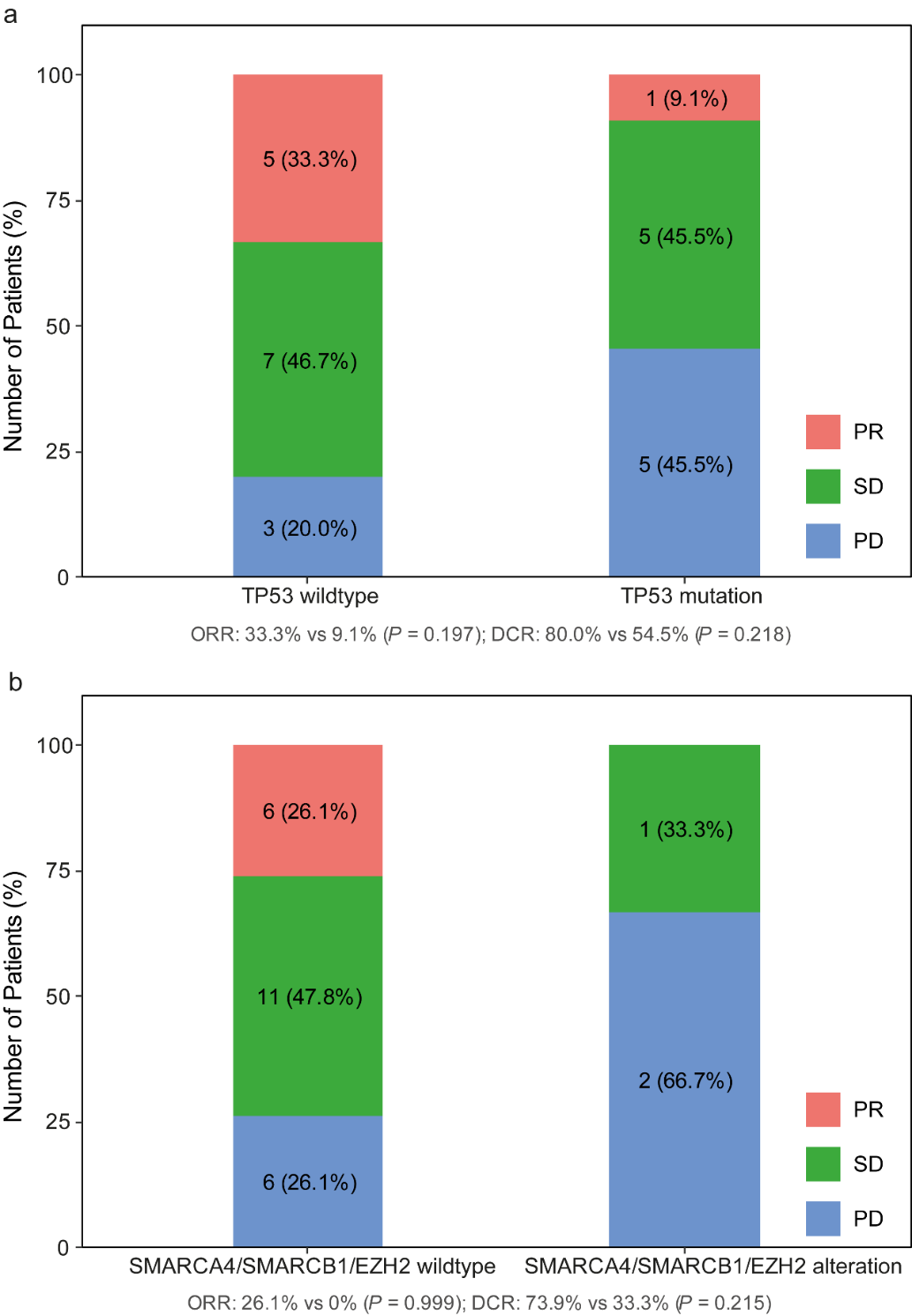


Figure. S3.

**Co-mutations and their association with tumor response.**

(a) Concurrent TP53 mutation. (b) Concurrent SMARCA4, SMARCB1and EZH2 alterations.


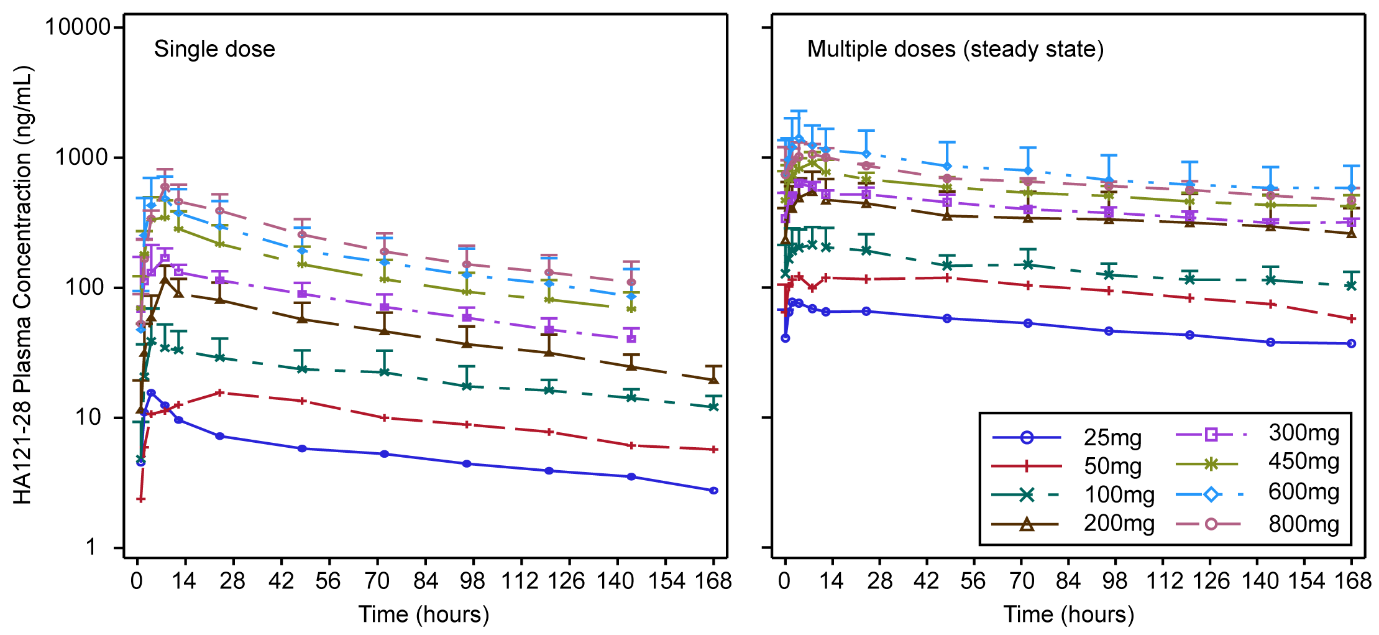


Figure. S4.

**Plasma concentration-time curves.**

Plasma concentration-time curves of HA121-28 on a semi-logarithmic scale after a single dose and multiple doses for 21 days. Point 0 represents the pre-dose values for all groups.


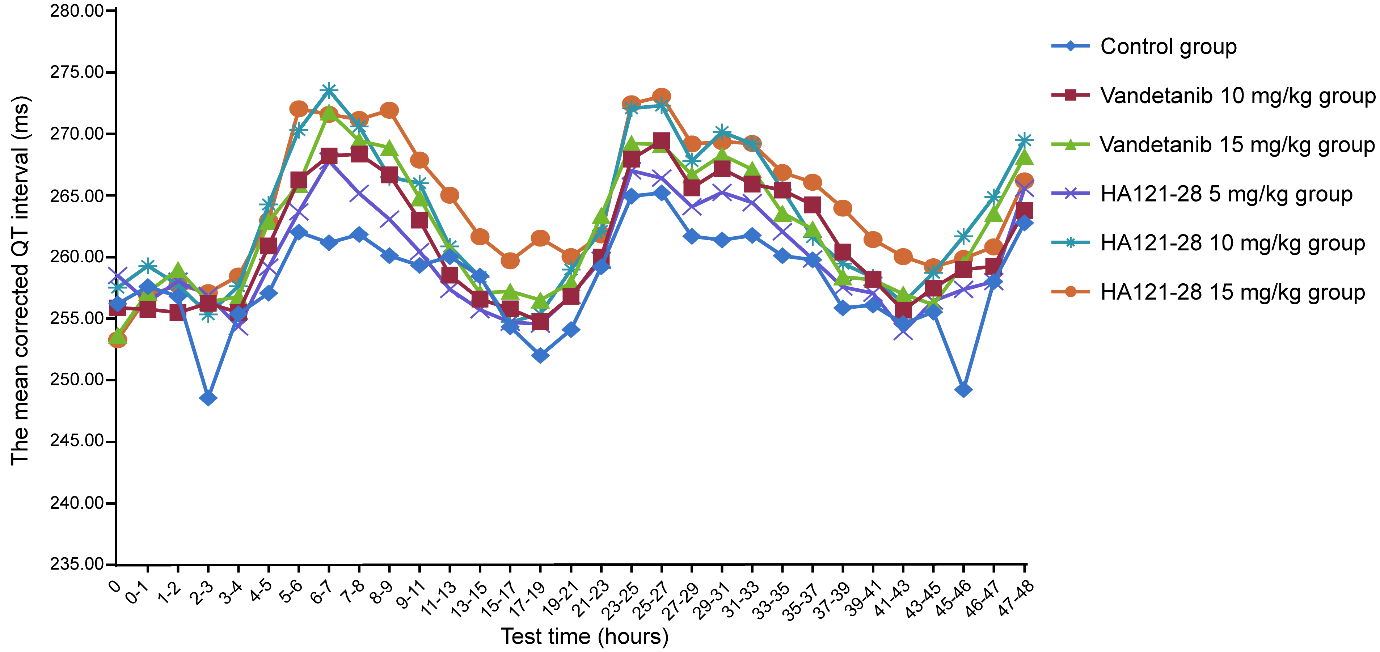


Figure. S5.

**Effect of oral administration of HA121-28 on ECG index-corrected QT interval in six awake Beagle dogs.**

Pre-clinical toxicity study on the cardiovascular system in conscious Beagle dogs (involving six groups: a control group, vandetanib 10 mg/kg and 15 mg/kg groups, and HA121-28 5 mg/kg, 10 mg/kg, and 15 mg/kg groups, with six Beagle dogs per group).

Table S1. Adverse events summary (SS*)

|  | No. (%) |  |  |  |  | |  | |  | |  | |  |  |  |  |  | |
| --- | --- | --- | --- | --- | --- | --- | --- | --- | --- | --- | --- | --- | --- | --- | --- | --- | --- | --- |
|  | Phase 1 | (N=160) |  |  |  | |  | |  | |  | |  |  |  |  | Phase 2 | |
|  | 25-50 mg  (N=2) |  | 100 mg (N=4) |  | 200 mg  (N=4) | | 300 mg  (N=4) | | 450 mg  (N=79) | | 600 mg  (N=64) | | 800 mg  (N=3) |  | Phase 1 Total  (N=160) |  | 450 mg  (N=48) |  |
|  | All Grades | ≥ Grade 3 | All Grades | ≥ Grade 3 | All Grades | ≥ Grade 3 | All Grades | ≥ Grade 3 | All Grades | ≥ Grade 3 | All Grades | ≥ Grade 3 | All Grades | ≥ Grade 3 | All Grades | ≥ Grade 3 | All Grades | ≥ Grade 3 |
| TEAEs | 2 (100) | 1 (50.0) | 4 (100) | 1 (25.0) | 4 (100) | 0 | 4 (100) | 1 (25.0) | 78 (98.7) | 29 (36.7) | 64 (100) | 44 (68.8) | 3 (100) | 2 (66.7) | 159 (99.4) | 78 (48.8) | 48 (100) | 35 (72.9) |
| TRAEs | 2 (100) | 1 (50.0) | 3 (75.0) | 0 | 4 (100) | 0 | 4 (100) | 0 | 77 (97.5) | 25 (31.6) | 64 (100) | 36 (56.3) | 3 (100) | 2 (66.7) | 157 (98.1) | 64 (40.0) | 46 (95.8) | 30 (62.5) |
| SAEs | 0 | 0 | 0 | 0 | 0 | 0 | 1 (25.0) | 1 (25.0) | 15 (19.0) | 14 (17.7) | 17 (26.6) | 15 (23.4) | 1 (33.3) | 1 (33.3) | 34 (21.3) | 31 (19.4) | 19 (39.6) | 15 (31.3) |
| Serious drug-related adverse event | 0 | 0 | 0 | 0 | 0 | 0 | 0 | 0 | 9 (11.4) | 7 (8.9) | 13 (20.3) | 11 (17.2) | 1 (33.3) | 1 (33.3) | 23 (14.4) | 19 (11.9) | 8 (16.7) | 7 (14.6) |
| TEAEs leading to dose interruption | 0 | 0 | 0 | 0 | 2 (50.0) | 0 | 0 | 0 | 26 (32.9) | 16 (20.3) | 29 (45.3) | 21 (32.8) | 1 (33.3) | 1 (33.3) | 58 (36.3) | 38 (23.8) | 18 (37.5) | 13 (27.1) |
| TEAEs leading to dose reduction | 0 | 0 | 0 | 0 | 0 | 0 | 0 | 0 | 9 (11.4) | 4 (5.1) | 13 (20.3) | 1 (1.6) | 1 (33.3) | 1 (33.3) | 23 (14.4) | 6 (3.8) | 7 (14.6) | 4 (8.3) |
| TEAEs leading to permanent discontinuation | 0 | 0 | 0 | 0 | 0 | 0 | 1 (25.0) | 1 (25.0) | 8 (10.1) | 5 (6.3) | 5 (7.8) | 3 (4.7) | 0 | 0 | 14 (8.8) | 9 (5.6) | 2 (4.2) | 2 (4.2) |
| AESI (≥ grade 3 Prolonged QT interval) | 0 | 0 | 0 | 0 | 0 | 0 | 0 | 0 | 11 (13.9) | 11 (13.9) | 12 (18.8) | 12 (18.8) | 1 (33.3) | 1 (33.3) | 24 (15.0) | 24 (15.0) | 15 (31.3) | 15 (31.3) |

Data are expressed as count (percentage). Percentages may not add up to 100% because of rounding.

*SS included all patients who received at least one dose of the study drug and had safety data recorded in the phase 1 and phase 2 studies.

AESI adverse event of special interest, SS safety set, TEAE Treatment-emergent adverse event, TRAE treatment-related adverse event

Table S2. Treatment-emergent adverse events by dose levels in the phase 1 study (SS*)

| Preferred Term | No. (%) | | | | | | | | | | | | | |  |  |
| --- | --- | --- | --- | --- | --- | --- | --- | --- | --- | --- | --- | --- | --- | --- | --- | --- |
|  | 25-50 mg  (N=2) | | 100 mg  (N=4) | | 200 mg  (N=4) | | 300 mg  (N=4) | | 450 mg  (N=79) | | 600 mg  (N=64) | | 800 mg  (N=3) | | Phase 1 Total (N=160) |  |
|  | All | ≥ Grade 3 | All | ≥ Grade 3 | All | ≥ Grade 3 | All | ≥ Grade 3 | All | ≥ Grade 3 | All | ≥ Grade 3 | All | ≥ Grade 3 | All | ≥ Grade 3 |
| Any | 2 (100) | 1 (50.0) | 4 (100) | 1 (25.0) | 4 (100) | 0 | 4 (100) | 1 (25.0) | 78 (98.7) | 29 (36.7) | 64 (100) | 44 (68.8) | 3 (100) | 2 (66.7) | 159 (99.4) | 77 (48.1) |
| Diarrhea | 1 (50.0) | 1 (50.0) | 1 (25.0) | 0 | 2 (50.0) | 0 | 2 (50.0) | 0 | 60 (75.9) | 2 (2.5) | 57 (89.1) | 10 (15.6) | 3 (100) | 0 | 126 (78.8) | 13 (8.1) |
| Rash | 1 (50.0) | 0 | 1 (25.0) | 0 | 2 (50.0) | 0 | 2 (50.0) | 0 | 43 (54.4) | 4 (5.1) | 40 (62.5) | 5 (7.8) | 3 (100) | 0 | 92 (57.5) | 9 (5.6) |
| Prolonged QT interval | 0 | 0 | 0 | 0 | 1 (25.0) | 0 | 2 (50.0) | 0 | 38 (48.1) | 11 (13.9) | 37 (57.8) | 12 (18.8) | 2 (66.7) | 1 (33.3) | 80 (50.0) | 24 (15.0) |
| Proteinuria | 1 (50.0) | 0 | 2 (50.0) | 0 | 1 (25.0) | 0 | 1 (25.0) | 0 | 36 (45.6) | 0 | 26 (40.6) | 0 | 1 (33.3) | 0 | 68 (42.5) | 0 |
| ALT increased | 1 (50.0) | 0 | 0 | 0 | 0 | 0 | 0 | 0 | 33 (41.8) | 2 (2.5) | 22 (34.4) | 2 (3.1) | 1 (33.3) | 0 | 57 (35.6) | 4 (2.5) |
| AST increased | 1 (50.0) | 0 | 0 | 0 | 1 (25.0) | 0 | 0 | 0 | 30 (38.0) | 2 (2.5) | 22 (34.4) | 3 (4.7) | 1 (33.3) | 0 | 55 (34.4) | 5 (3.1) |
| Hypokalemia | 0 | 0 | 0 | 0 | 1 (25.0) | 0 | 0 | 0 | 19 (24.1) | 2 (2.5) | 22 (34.4) | 4 (6.3) | 3 (100) | 0 | 45 (28.1) | 6 (3.8) |
| Blood creatinine increased | 0 | 0 | 1 (25.0) | 0 | 0 | 0 | 0 | 0 | 22 (27.8) | 1 (1.3) | 18 (28.1) | 1 (1.6) | 1 (33.3) | 0 | 42 (26.3) | 2 (1.3) |
| Nausea | 1 (50.0) | 0 | 0 | 0 | 1 (25.0) | 0 | 0 | 0 | 18 (22.8) | 0 | 20 (31.3) | 2 (3.1) | 2 (66.7) | 0 | 42 (26.3) | 2 (1.3) |
| Vomiting | 1 (50.0) | 0 | 1 (25.0) | 0 | 0 | 0 | 1 (25.0) | 0 | 13 (16.5) | 0 | 17 (26.6) | 2 (3.1) | 2 (66.7) | 0 | 35 (21.9) | 2 (1.3) |
| Hypoalbuminemia | 0 | 0 | 0 | 0 | 1 (25.0) | 0 | 0 | 0 | 16 (20.3) | 1 (1.3) | 15 (23.4) | 0 | 1 (33.3) | 0 | 33 (20.6) | 1 (0.6) |
| Decreased appetite | 0 | 0 | 0 | 0 | 0 | 0 | 1 (25.0) | 0 | 13 (16.5) | 0 | 17 (26.6) | 2 (3.1) | 1 (33.3) | 1 (33.3) | 32 (20.0) | 3 (1.9) |
| Asthenia | 1 (50.0) | 0 | 0 | 0 | 0 | 0 | 0 | 0 | 13 (16.5) | 0 | 12 (18.8) | 0 | 1 (33.3) | 0 | 27 (16.9) | 0 |
| Anemia | 0 | 0 | 1 (25.0) | 1 (25.0) | 1 (25.0) | 0 | 0 | 0 | 9 (11.4) | 0 | 14 (21.9) | 1 (1.6) | 0 | 0 | 25 (15.6) | 2 (1.3) |
| Hypertension | 0 | 0 | 0 | 0 | 0 | 0 | 0 | 0 | 6 (7.6) | 1 (1.3) | 12 (18.8) | 6 (9.4) | 1 (33.3) | 0 | 25 (15.6) | 0 |
| Hypertriglyceridemia | 0 | 0 | 0 | 0 | 2 (50.0) | 0 | 1 (25.0) | 0 | 12 (15.2) | 0 | 6 (9.4) | 0 | 0 | 0 | 21 (13.1) | 0 |
| White blood cell counts decreased | 1 (50.0) | 0 | 0 | 0 | 0 | 0 | 1 (25.0) | 0 | 6 (7.6) | 1 (1.3) | 9 (14.1) | 2 (3.1) | 2 (66.7) | 0 | 19 (11.9) | 7 (4.4) |
| Fecal occult blood positive | 0 | 0 | 1 (25.0) | 0 | 2 (50.0) | 0 | 0 | 0 | 7 (8.9) | 0 | 13 (20.3) | 0 | 2 (66.7) | 0 | 19 (11.9) | 3 (1.9) |
| Blood ALP increased | 0 | 0 | 0 | 0 | 0 | 0 | 1 (25.0) | 0 | 12 (15.2) | 0 | 5 (7.8) | 0 | 0 | 0 | 18 (11.3) | 0 |
| Dizziness | 2 (100) | 0 | 1 (25.0) | 0 | 0 | 0 | 0 | 0 | 9 (11.4) | 0 | 6 (9.4) | 0 | 0 | 0 | 18 (11.3) | 0 |
| Neutrophil counts decreased | 0 | 0 | 0 | 0 | 0 | 0 | 0 | 0 | 6 (7.6) | 1 (1.3) | 9 (14.1) | 2 (3.1) | 0 | 0 | 15 (9.4) | 3 (1.9) |
| Platelet counts decreased | 0 | 0 | 0 | 0 | 0 | 0 | 0 | 0 | 8 (10.1) | 1 (1.3) | 7 (10.9) | 1 (1.6) | 0 | 0 | 15 (9.4) | 2 (1.3) |
| Hyponatremia | 0 | 0 | 1 (25.0) | 0 | 0 | 0 | 0 | 0 | 7 (8.9) | 0 | 7 (10.9) | 1 (1.6) | 0 | 0 | 15 (9.4) | 1 (0.6) |
| Upper respiratory tract infection | 0 | 0 | 0 | 0 | 1 (25.0) | 0 | 0 | 0 | 8 (10.1) | 1 (1.3) | 4 (6.3) | 1 (1.6) | 1 (33.3) | 0 | 14 (8.8) | 2 (1.3) |
| GGT increased | 0 | 0 | 0 | 0 | 0 | 0 | 0 | 0 | 10 (12.7) | 2 (2.5) | 3 (4.7) | 1 (1.6) | 0 | 0 | 13 (8.1) | 3 (1.9) |
| Sinus tachycardia | 0 | 0 | 1 (25.0) | 0 | 1 (25.0) | 0 | 0 | 0 | 5 (6.3) | 0 | 6 (9.4) | 0 | 0 | 0 | 13 (8.1) | 0 |
| Cough | 2 (100.0) | 0 | 1 (25.0) | 0 | 0 | 0 | 0 | 0 | 3 (3.8) | 0 | 3 (4.7) | 0 | 0 | 0 | 9 (5.6) | 0 |
| Weight loss | 0 | 0 | 0 | 0 | 0 | 0 | 0 | 0 | 4 (5.1) | 0 | 4 (6.3) | 0 | 1 (33.3) | 0 | 9 (5.6) | 0 |
| Electrocardiogram T wave abnormal | 0 | 0 | 0 | 0 | 0 | 0 | 0 | 0 | 5 (6.3) | 0 | 4 (6.3) | 0 | 0 | 0 | 8 (5.0） |  |
| COVID-19 | 0 | 0 | 0 | 0 | 0 | 0 | 0 | 0 | 7 (8.9) | 0 | 0 | 0 | 0 | 0 | 7 (4.4) | 0 |
| Urinary tract infection | 0 | 0 | 0 | 0 | 0 | 0 | 0 | 0 | 2 (2.5) | 0 | 4 (6.3) | 0 | 0 | 0 | 6 (3.8） | 0 |

Data are expressed as count (percentage). Percentages may not add up to 100% because of rounding.

*SS included all patients who received at least one dose of the study drug and had safety data recorded in the phase 1 study. Treatment-emergent adverse events are summarized by Preferred Term according to MedDRA for events occurring in ≥5% of patients in SS.

ALP alkaline phosphatase, ALT alanine aminotransferase, AST Aspartate aminotransferase, GGT γ-glutamyl transpeptidase, SS: safety set

Table S3. Treatment-related adverse events by dose levels in the phase 1 study (SS*)

| Preferred Term | No. (%) | |  | |  | | |  | |  | |  | |  | |  | | |  | |  | |
| --- | --- | --- | --- | --- | --- | --- | --- | --- | --- | --- | --- | --- | --- | --- | --- | --- | --- | --- | --- | --- | --- | --- |
|  | 25-50 mg  (N=2) | |  | | 100 mg  (N=4) | | |  | | 200 mg  (N=4) | | 300 mg  (N=4) | | 450 mg  (N=79) | | 600 mg  (N=64) | | | 800 mg  (N=3) | |  | |
|  | All | ≥ Grade 3 | | All | | ≥ Grade 3 | All | | ≥ Grade 3 | | All | ≥ Grade 3 | All | | ≥ Grade 3 | | All | ≥ Grade 3 | | All | | ≥ Grade 3 |
| Any | 2 (100) | 1 (50.0) | | 3 (75.0) | | 0 | 4 (100) | | 0 | | 4 (100) | 0 | 77 (97.5) | | 25 (31.6) | | 64 (100) | 36 (56.3) | | 3 (100) | | 2 (66.7) |
| Diarrhea | 0 | 0 | | 1 (25.0) | | 0 | 0 | | 0 | | 1 (25.0) | 0 | 60 (75.9) | | 1 (1.3) | | 57 (89.1) | 10 (15.6) | | 3 (100) | | 0 |
| Rash | 1 (50.0) | 0 | | 1 (25.0) | | 0 | 1 (25.0) | | 0 | | 2 (50.0) | 0 | 43 (54.4) | | 4 (5.1) | | 39 (60.9) | 5 (7.8) | | 3 (100) | | 0 |
| Prolonged QT interval | 0 | 0 | | 0 | | 0 | 1 (25.0) | | 0 | | 2 (50.0) | 0 | 37 (46.8) | | 11 (13.9) | | 37 (57.8) | 12 (18.8) | | 2 (66.7) | | 1 (33.3) |
| ALT increased | 0 | 0 | | 0 | | 0 | 0 | | 0 | | 0 | 0 | 32 (40.5) | | 2 (2.5) | | 21 (32.8) | 0 | | 1 (33.3) | | 0 |
| AST increased | 0 | 0 | | 0 | | 0 | 1 (25.0) | | 0 | | 0 | 0 | 29 (36.7) | | 2 (2.5) | | 21 (32.8) | 1 (1.6) | | 1 (33.3) | | 0 |
| Proteinuria | 0 | 0 | | 1 (25.0) | | 0 | 0 | | 0 | | 1 (25.0) | 0 | 31 (39.2) | | 0 | | 15 (23.4) | 0 | | 0 | | 0 |
| Blood creatinine increased | 0 | 0 | | 0 | | 0 | 0 | | 0 | | 0 | 0 | 21 (26.6) | | 1 (1.3) | | 15 (23.4) | 1 (1.6) | | 0 | | 0 |
| Nausea | 0 | 0 | | 0 | | 0 | 1 (25.0) | | 0 | | 0 | 0 | 17 (21.5) | | 0 | | 20 (31.3) | 2 (3.1) | | 2 (66.7) | | 0 |
| Vomiting | 0 | 0 | | 0 | | 0 | 0 | | 0 | | 1 (25.0) | 0 | 12 (15.2) | | 0 | | 17 (26.6) | 2 (3.1) | | 2 (66.7) | | 0 |
| Hypokalemia | 0 | 0 | | 0 | | 0 | 0 | | 0 | | 0 | 0 | 16 (20.3) | | 2 (2.5) | | 11 (17.2) | 2 (3.1) | | 0 | | 0 |
| Decreased appetite | 0 | 0 | | 0 | | 0 | 0 | | 0 | | 0 | 0 | 12 (15.2) | | 0 | | 15 (23.4) | 2 (3.1) | | 1 (33.3) | | 1 (33.3) |
| Asthenia | 0 | 0 | | 0 | | 0 | 0 | | 0 | | 0 | 0 | 10 (12.7) | | 0 | | 11 (17.2) | 0 | | 1 (33.3) | | 0 |
| Hypertension | 0 | 0 | | 0 | | 0 | 0 | | 0 | | 0 | 0 | 5 (6.3) | | 1 (1.3) | | 11 (17.2) | 5 (7.8) | | 1 (33.3) | | 0 |
| Hypoalbuminemia | 0 | 0 | | 0 | | 0 | 1 (25.0) | | 0 | | 0 | 0 | 12 (15.2) | | 1 (1.3) | | 8 (12.5) | 0 | | 0 | | 0 |
| White blood cell counts decreased | 0 | 0 | | 0 | | 0 | 0 | | 0 | | 1 (25.0) | 0 | 4 (5.1) | | 1 (1.3) | | 7 (10.9) | 1 (1.6) | | 1 (33.3) | | 0 |
| Neutrophil counts decreased | 0 | 0 | | 0 | | 0 | 0 | | 0 | | 0 | 0 | 6 (7.6) | | 1 (1.3) | | 7 (10.9) | 1 (1.6) | | 0 | | 0 |
| GGT increased | 0 | 0 | | 0 | | 0 | 0 | | 0 | | 0 | 0 | 10 (12.7) | | 2 (2.5) | | 3 (4.7) | 1 (1.6) | | 0 | | 0 |
| Platelet counts decreased | 0 | 0 | | 0 | | 0 | 0 | | 0 | | 0 | 0 | 8 (10.1) | | 1 (1.3) | | 5 (7.8) | 0 | | 0 | | 0 |
| Blood ALP increased | 0 | 0 | | 0 | | 0 | 0 | | 0 | | 1 (25.0) | 0 | 11 (13.9) | | 0 | | 2 (3.1) | 0 | | 0 | | 0 |
| Anemia | 0 | 0 | | 0 | | 0 | 1 (25.0) | | 0 | | 0 | 0 | 6 (7.6) | | 0 | | 7 (10.9) | 1 (1.6) | | 0 | | 0 |
| Hypertriglyceridemia | 0 | 0 | | 0 | | 0 | 1 (25.0) | | 0 | | 1 (25.0) | 0 | 8 (10.1) | | 0 | | 3 (4.7) | 0 | | 0 | | 0 |

Data are expressed as count (percentage). Percentages may not add up to 100% because of rounding.

*SS included all patients who received at least one dose of the study drug and had safety data recorded in the phase 1 study. Treatment-related adverse events are summarized by Preferred Term according to MedDRA for events occurring in ≥5% of patients in the SS.

ALP alkaline phosphatase, ALT alanine aminotransferase, AST Aspartate aminotransferase, GGT γ-glutamyl transpeptidase, SS: safety set

Table S4. Serious adverse events (SS*)

| Preferred Term | No. (%) | |  | |  | |  |  |  |  |
| --- | --- | --- | --- | --- | --- | --- | --- | --- | --- | --- |
|  | Phase 1^#^ | |  | |  | |  |  | Phase 2 |  |
|  | 300 mg  (N=4) | | 450 mg  (N=79) | | 600 mg  (N=64) | | 800 mg  (N=3) |  | 450 mg  (N=48) |  |
|  | TEAE | TRAE | TEAE | TRAE | TEAE | TRAE | TEAE | TRAE | TEAE | TRAE |
| Any | 1 (25.0) | 0 | 15 (19.0) | 9 (11.4) | 17 (26.6) | 13 (20.3) | 1 (33.3) | 1 (33.3) | 19 (39.6) | 9 (18.8) |
| Rash | 0 | 0 | 1 (1.3) | 1 (1.3) | 3 (4.7) | 3 (4.7) | 0 | 0 | 2 (4.2) | 2 (4.2) |
| Malignant neoplasm progression | 0 | 0 | 1 (1.3) | 0 | 2 (3.1) | 1 (1.6) | 0 | 0 | 1 (2.1) | 0 |
| ALT increased | 0 | 0 | 0 | 0 | 2 (3.1) | 1 (1.6) | 0 | 0 | 1 (2.1) | 1 (2.1) |
| Hemoptysis | 0 | 0 | 2 (2.5) | 2 (2.5) | 1 (1.6) | 1 (1.6) | 0 | 0 | 0 | 0 |
| AST increased | 0 | 0 | 0 | 0 | 2 (3.1) | 1 (1.6) | 0 | 0 | 1 (2.1) | 1 (2.1) |
| Diarrhea | 0 | 0 | 0 | 0 | 2 (3.1) | 2 (3.1) | 0 | 0 | 1 (2.1) | 1 (2.1) |
| Weight loss | 0 | 0 | 0 | 0 | 2 (3.1) | 2 (3.1) | 1 (33.3) | 1 (33.3) | 0 | 0 |
| Hypertension | 0 | 0 | 1 (1.3) | 1 (1.3) | 0 | 0 | 0 | 0 | 2 (4.2) | 2 (4.2) |
| Upper respiratory tract infection | 0 | 0 | 1 (1.3) | 1 (1.3) | 1 (1.6) | 0 | 0 | 0 | 0 | 0 |
| Vomiting | 0 | 0 | 0 | 0 | 2 (3.1) | 2 (3.1) | 0 | 0 | 0 | 0 |
| Nausea | 0 | 0 | 0 | 0 | 2 (3.1) | 2 (3.1) | 0 | 0 | 0 | 0 |
| Death | 0 | 0 | 1 (1.3) | 0 | 0 | 0 | 0 | 0 | 1 (2.1) | 0 |
| Seizure | 0 | 0 | 2 (2.5) | 0 | 0 | 0 | 0 | 0 | 0 | 0 |
| Pneumonitis | 0 | 0 | 1 (1.3) | 1 (1.3) | 0 | 0 | 0 | 0 | 1 (2.1) | 0 |
| COVID-19 | 0 | 0 | 0 | 0 | 0 | 0 | 0 | 0 | 1 (2.1) | 0 |
| Aortic intramural hematoma | 1 (25.0) | 0 | 0 | 0 | 0 | 0 | 0 | 0 | 0 | 0 |
| Hypoalbuminemia | 0 | 0 | 0 | 0 | 1 (1.6) | 1 (1.6) | 0 | 0 | 0 | 0 |
| Coronary artery atherosclerosis | 0 | 0 | 0 | 0 | 0 | 0 | 0 | 0 | 1 (2.1) | 0 |
| Pyrexia | 0 | 0 | 0 | 0 | 0 | 0 | 0 | 0 | 1 (2.1) | 0 |
| Dyspnea | 0 | 0 | 0 | 0 | 0 | 0 | 0 | 0 | 1 (2.1) | 0 |
| Respiratory failure | 0 | 0 | 0 | 0 | 0 | 0 | 0 | 0 | 1 (2.1) | 0 |
| Multiple organ dysfunction syndrome | 0 | 0 | 1 (1.3) | 0 | 0 | 0 | 0 | 0 | 0 | 0 |
| Erythema multiforme | 0 | 0 | 0 | 0 | 0 | 0 | 0 | 0 | 1 (2.1) | 1 (2.1) |
| Headache | 0 | 0 | 0 | 0 | 0 | 0 | 0 | 0 | 1 (2.1) | 0 |
| Ventricular tachycardia | 0 | 0 | 0 | 0 | 1 (1.6) | 1 (1.6) | 0 | 0 | 0 | 0 |
| Cardiac tamponade | 0 | 0 | 1 (1.3) | 0 | 0 | 0 | 0 | 0 | 0 | 0 |
| Arrhythmia | 0 | 0 | 1 (1.3) | 1 (1.3) | 0 | 0 | 0 | 0 | 0 | 0 |
| Prolonged QT interval | 0 | 0 | 0 | 0 | 1 (1.6) | 1 (1.6) | 0 | 0 | 0 | 0 |
| Sudden cardiac arrest | 0 | 0 | 0 | 0 | 1 (1.6) | 1 (1.6) | 0 | 0 | 0 | 0 |
| Acute cholecystitis | 0 | 0 | 1 (1.3) | 0 | 0 | 0 | 0 | 0 | 0 | 0 |
| Acute pancreatitis | 0 | 0 | 0 | 0 | 1 (1.6) | 0 | 0 | 0 | 0 | 0 |
| Septic shock | 0 | 0 | 0 | 0 | 0 | 0 | 0 | 0 | 1 (2.1) | 0 |
| Pneumonia | 0 | 0 | 0 | 0 | 0 | 0 | 0 | 0 | 1 (2.1) | 0 |
| Embolism | 0 | 0 | 0 | 0 | 0 | 0 | 0 | 0 | 1 (2.1) | 0 |
| Sudden death | 0 | 0 | 0 | 0 | 1 (1.6) | 1 (1.6) | 0 | 0 | 0 | 0 |
| Rectal hemorrhage | 0 | 0 | 1 (1.3) | 1 (1.3) | 0 | 0 | 0 | 0 | 0 | 0 |
| Anal abscess | 0 | 0 | 0 | 0 | 1 (1.6) | 1 (1.6) | 0 | 0 | 0 | 0 |
| Liver injury | 0 | 0 | 0 | 0 | 1 (1.6) | 0 | 0 | 0 | 0 | 0 |
| Intestinal obstruction | 0 | 0 | 1 (1.3) | 1 (1.3) | 0 | 0 | 0 | 0 | 0 | 0 |
| Pulmonary embolism | 0 | 0 | 0 | 0 | 0 | 0 | 0 | 0 | 1 (2.1) | 1 (2.1) |
| Lung adenocarcinoma | 0 | 0 | 0 | 0 | 0 | 0 | 0 | 0 | 1 (2.1) | 0 |
| Nephrolithiasis | 0 | 0 | 0 | 0 | 0 | 0 | 0 | 0 | 1 (2.1) | 0 |
| Hydronephrosis | 0 | 0 | 0 | 0 | 0 | 0 | 0 | 0 | 1 (2.1) | 0 |
| Pleural effusion | 0 | 0 | 1 (1.3) | 0 | 0 | 0 | 0 | 0 | 0 | 0 |
| Intra-abdominal fluid collection | 0 | 0 | 0 | 0 | 0 | 0 | 0 | 0 | 1 (2.1) | 0 |
| Abdominal Pain | 0 | 0 | 0 | 0 | 0 | 0 | 0 | 0 | 1 (2.1) | 1 (2.1) |
| Abdominal distension | 0 | 0 | 0 | 0 | 0 | 0 | 0 | 0 | 1 (2.1) | 0 |
| Drug eruption | 0 | 0 | 0 | 0 | 1 (1.6) | 1 (1.6) | 0 | 0 | 0 | 0 |
| Platelet counts decreased | 0 | 0 | 0 | 0 | 1 (1.6) | 0 | 0 | 0 | 0 | 0 |
| Blood creatinine increased | 0 | 0 | 0 | 0 | 1 (1.6) | 1 (1.6) | 0 | 0 | 0 | 0 |
| Blood bilirubin increased | 0 | 0 | 0 | 0 | 1 (1.6) | 1 (1.6) | 0 | 0 | 0 | 0 |
| Intracranial tumor hemorrhage | 0 | 0 | 0 | 0 | 0 | 0 | 0 | 0 | 1 (2.1) | 0 |
| Dermatitis allergic | 0 | 0 | 0 | 0 | 0 | 0 | 0 | 0 | 0 | 1 (2.1) |

Data are expressed as count (percentage). Percentages may not add up to 100% because of rounding.

*SS included all patients who received at least one dose of the study drug and had safety data recorded in the phase 1 and phase 2 studies. All serious adverse events are summarized by Preferred Term according to MedDRA.

^#^ There were no SAEs reported in 25, 50, 100, and 200 mg groups

ALT alanine aminotransferase, AST Aspartate aminotransferase, TEAE Treatment-emergent adverse event, TRAE Treatment-related adverse event, SS safety set

##

Table S5. Treatment-emergent adverse events in the phase 2 study (SS*)

| Preferred Term | Phase 2 (N=48) | 450 mg |
| --- | --- | --- |
|  | All | ≥ Grade 3 |
| Any | 48 (100) | 35 (72.9) |
| Diarrhea | 35 (72.9) | 6 (12.5) |
| Rash | 30 (62.5) | 1 (2.1) |
| Prolonged QT interval | 21 (43.8) | 15 (31.3) |
| Proteinuria | 16 (33.3) | 0 |
| ALT increased | 20 (41.7) | 3 (6.3) |
| AST increased | 21 (43.8) | 2 (4.2) |
| Hypokalemia | 11 (22.9) | 3 (6.3) |
| Blood creatinine increased | 9 (18.8) | 0 |
| Nausea | 3 (6.3) | 0 |
| Vomiting | 6 (12.5) | 0 |
| Hypoalbuminemia | 4 (8.3) | 0 |
| Decreased appetite | 5 (10.4) | 1 (2.1) |
| Asthenia | 7 (14.6) | 0 |
| Anemia | 5 (10.4) | 0 |
| Fecal occult blood positive | 0 | 0 |
| Hypertriglyceridemia | 7 (14.6) | 0 |
| Hypertension | 11 (22.9) | 9 (18.8) |
| White blood cell counts decreased | 8 (16.7) | 1 (2.1) |
| Blood ALP increased | 5 (10.4) | 0 |
| Dizziness | 3 (6.3) | 0 |
| Neutrophil counts decreased | 7 (14.6) | 2 (4.2) |
| Platelet counts decreased | 6 (12.5) | 0 |
| Hyponatremia | 5 (10.4) | 0 |
| GGT increased | 6 (12.5) | 0 |
| Sinus tachycardia | 5 (10.4) | 0 |
| Cough | 6 (12.5) | 0 |
| Weight loss | 5 (10.4) | 0 |
| Electrocardiogram T wave abnormal | 5 (10.4) | 0 |
| COVID-19 | 7 (14.6) | 1 (2.1) |
| Urinary tract infection | 5 (10.4) | 1 (2.1) |

Data are expressed as count (percentage). Percentages may not add up to 100% because of rounding.

*SS included all patients who received at least one dose of the study drug and had safety data recorded in the phase 2 study. Treatment-emergent adverse events are summarized by Preferred Term according to MedDRA for events occurring in ≥10% of patients in SS.

ALP alkaline phosphatase, ALT alanine aminotransferase, AST Aspartate aminotransferase, GGT γ-glutamyl transpeptidase, TEAE treatment-emergent adverse events, SS safety set

Table S6. Treatment-related adverse events in patients with prior immunotherapy treatment or without in Phase1/2 study (SS*)

| Preferred Term | With prior immunotherapy treatment (N=43) | | Without prior immunotherapy treatment (N=165) | |
| --- | --- | --- | --- | --- |
|  | All | ≥ Grade 3 | All | ≥ Grade 3 |
| Any | 42 (97.7) | 25 (58.1) | 161 (97.6) | 68 (41.2) |
| Diarrhea | 32 (74.4) | 4 (9.3) | 125 (75.8) | 12 (7.3) |
| Rash | 31 (72.1) | 3 (7.0) | 88 (53.3) | 7 (4.2) |
| Prolonged QT interval | 23 (53.5) | 12 (27.9) | 76 (46.1) | 26 (15.8) |
| ALT increased | 22 (51.2) | 1 (2.3) | 52 (31.5) | 3 (1.8) |
| AST increased | 19 (44.2) | 1 (2.3) | 52 (31.5) | 3 (1.8) |
| Proteinuria | 17 (39.5) | 0 | 48 (29.1) | 0 |
| Blood creatinine increased | 11 (25.6) | 0 | 33 (20.0) | 2 (1.2) |
| Nausea | 6 (14.0) | 0 | 37 (22.4) | 2 (1.2) |
| Vomiting | 7 (16.3) | 0 | 30 (18.2) | 2 (1.2) |
| Hypokalemia | 9 (20.9) | 3 (7.0) | 26 (15.8) | 4 (2.4) |
| Decreased appetite | 7 (16.3) | 0 | 25 (15.2) | 4 (2.4) |
| Fatigue | 6 (14.0) | 0 | 22 (13.3) | 0 |
| Hypertension | 5 (11.6) | 0 | 22 (13.3) | 14 (8.5) |
| Hypoalbuminemia | 5 (11.6) | 0 | 20 (12.1) | 1 (0.6) |
| White blood cell counts decreased | 7 (16.3) | 1 (2.3) | 14 (8.5) | 2 (1.2) |
| Neutrophil counts decreased | 7 (16.3) | 3 (7.0) | 13 (7.9) | 1 (0.6) |
| Platelet counts decreased | 7 (16.3) | 0 | 12 (7.3) | 1 (0.6) |
| Anemia | 5 (11.6) | 0 | 14 (8.5) | 1 (0.6) |
| Weight loss | 6 (14.0) | 0 | 6 (3.6) | 0 |
| Decreased lymphocyte counts | 5 (11.6) | 0 | 5 (3.0) | 0 |

Data are expressed as count (percentage). Percentages may not add up to 100% because of rounding.

*SS included all patients who received at least one dose of the study drug and had safety data recorded in the phase 2 study. Treatment-emergent adverse events are summarized by Preferred Term according to MedDRA for events occurring in ≥10% of patients in SS.

ALP alkaline phosphatase, ALT alanine aminotransferase, AST Aspartate aminotransferase, GGT γ-glutamyl transpeptidase, TEAE treatment-emergent adverse events, SS safety set

Table S7. The cardiac treatment-emergent advers events of patients who had Grade 3 QTc Prolongations

|  | Cardiac treatment-emergent adverse events | Grade |
| --- | --- | --- |
| **Phase 1 Study** |  |  |
| 1 | Cardiopalmus | 2 |
| 2 | Bradycardia | 1 |
| 3 | Nodal tachycardia | 1 |
| 4 | T wave anomaly | 1 |
| 5 | Incomplete right bundle branch block | 1 |
|  | Sinus bradycardia | 1 |
|  | Complete right bundle branch block | 1 |
| 6 | T wave anomaly | 1 |
| 7 | Ventricular arrhythmia | 4 |
| 8 | ST-T wave changes | 1 |
| 9 | T wave anomaly | 1 |
|  | Abnormal Q wave of III and aVF leads | 2 |
|  | Atrial premature beats | 1 |
|  | ST-T wave changes | 1 |
| **Phase 2 study** | | |
| 1 | sinus bradycardia | 1 |
| 2 | cronary arteriosclerosis | 2 |
| 3 | sinus bradycardia | 1 |
|  | ventricular extrasystole | 2 |
|  | arhythmia | 1 |
|  | Supraventricular extrasystoles | 1 |
| 4 | nodal tachycardia | 1 |
|  | Supraventricular extrasystoles | 1 |
|  | Left ventricular dysfunction | 2 |
|  | Ventricular wall movement diffusky diminshed | 2 |
|  | Ejection fraction reduced | 2 |
| 5 | nodal tachycardia | 1 |
|  | T wave abnormal | 1 |
| 6 | myocardial damage | 2 |
| 7 | T wave abnormal | 2 |
|  | U wave abnormal | 2 |
|  | Supraventricular extrasystoles | 2 |
| 8 | T wave abnormal | 1 |
|  | nodal tachycardia | 1 |
| 9 | nodal tachycardia | 1 |

Table S8. Demographic and disease characteristics of patients with RET fusion-positive non-small-cell lung cancer (ERS*)

| Characteristics | RET fusion+ NSCLC (N=97) |
| --- | --- |
| Age, years,  Median (IQR) | 53.0 (47.0, 60.0) |
| Sex |  |
| Male | 40 (41.2) |
| Female | 57 (58.8) |
| Body mass index, kg/m^2^，Median (IQR) | 23.4 (20.6, 25.6) |
| ECOG performance status |  |
| 0 | 31 (32.0) |
| 1 | 66 (68.0) |
| NSCLC histologic subtype |  |
| Adenocarcinoma | 39 (40.2) |
| Other | 5 (5.2) |
| Missing | 53 (54.6) |
| RET fusion |  |
| KIF5B-RET | 71 (73.2) |
| CCDC6-RET | 15 (15.5) |
| Other | 11 (11.3) |
| No. of prior systemic treatments |  |
| 0 line | 8 (8.2) |
| 1 line | 45 (46.4) |
| 2 lines | 22 (22.7) |
| ≥3 lines | 21 (21.6) |
| Others ^a^ | 1 (1.0) |
| Type of prior systemic treatments |  |
| Prior chemotherapy | 89 (91.8) |
| Prior PD-(L)1 inhibitor | 52 (53.6) |
| Prior radiotherapy | 24 (24.7) |
| Prior TKI treatment^#^ | 20 (20.6) |

Data are expressed as count (percentage) unless otherwise specified. Percentages may not add up to 100% because of rounding.

* ERS included all patients with RET fusion-positive non-small-cell lung cancer who received at least one dose of the study drug that was above 450mg and had at least one tumor response assessment in the phase 1 and phase 2 studies.

^#^ Prior TKI treatments included anlotinib, icotinib, afatinib, cabozantinib, osimertinib, KC1036, and gefitinib.

^a^ Chinese herbal medicine with an anti-tumor indication

ERS efficacy response analysis set, IQR Interquartile range, N (n) count

Table S9. Tumor response in RET fusion-positive non-small-cell lung cancer (ERS*)

| Outcome | No. (%) | | | |
| --- | --- | --- | --- | --- |
|  | CCDC6 (N=15) | KIF5B (N=71) | Other (N=11) | All (N=97) |
| Best response ^a^ |  |  |  |  |
| Complete response | 0 | 0 | 0 | 0 |
| Partial response | 7 (46.7) | 18 (25.4) | 1 (9.1) | 26 (26.8) |
| Stable disease | 6 (40.0) | 30 (42.3) | 8 (72.7) | 44 (45.4) |
| Progressive disease | 1 (6.7) | 21 (29.6) | 1 (9.1) | 23 (23.7) |
| Not evaluable | 1 (6.7) | 2 (2.8) | 1 (9.1) | 4 (4.1) |
| Confirmed objective response ^b^, n (%); [95% CI] | 7 (46.7) [21.3, 73.4] | 18 (25.4) [15.8, 37.1] | 1 (9.1) [0.2, 41.3] | 26 (26.8) [18.3, 36.8] |
| Confirmed disease control ^c^, n (%), [95% CI] | 13 (86.7) [59.5, 98.3] | 48 (67.6) [55.5, 78.2] | 9 (81.8) [48.2, 97.7] | 70 (72.2) [62.1, 80.8] |
| Duration of response, months, Median (95% CI) | 14.0 (5.8, NE) | 7.7 (3.7, NE) | NE | 7.7 (4.6, NE) |

Data are expressed as count (percentage) unless otherwise specified. Percentages may not add up to 100% because of rounding.

* ERS included all patients with RET fusion-positive non-small-cell lung cancer who received at least one dose of the study drug that was above 450mg and had at least one tumor response assessment in the phase 1 and phase 2 studies.

^a^ Best response was assessed by investigators as per RECIST version 1.1.

^b^ Confirmed objective response rate was calculated as the proportion of patients who achieved a confirmed complete or partial response.

^c^ Disease control was calculated as the proportion of patients who reached complete response, partial response, or stable disease for at least six weeks.

CI confidence Interval, ERS efficacy response analysis set, N (n) count, NE not evaluable

Table S10. Pharmacokinetic Profile of HA121-28 (PKCS*)

| Parameters | Mean | (CV%) |  |  |  |  |  |  |
| --- | --- | --- | --- | --- | --- | --- | --- | --- |
|  | 25 mg  (N=1) | 50 mg  (N=1) | 100 mg  (N=4) | 200 mg  (N=4) | 300 mg  (N=4) | 450 mg  (N=7) | 600 mg  (N=5) | 800 mg  (N=3) |
| **Single dose** |  |  |  |  |  |  |  |  |
| C_max_ (ng/mL) | 15.6 (-) | 15.6 (-) | 46.4 (27.0) | 115.5 (32.7) | 193.0 (70.1) | 374.7 (140.6) | 500.0 (224.3) | 610.0 (200.1) |
| AUC_0-24_ (h*ng/mL) | 237.1 (-) | 283.0 (-) | 725.9 (306.5) | 1884.6 (514.6) | 3037.9 (854.0) | 6271.3 (2275.9) | 8403.6 (4593.9) | 9721.8 (2598.4) |
| AUC_0-t_ (h*ng/mL) | 906.8 (-) | 1645.1 (-) | 3460.0 (1126.6) | 7763.0 (2539.2) | 11276.6 (2463.8) | 20218.4 (7301.9) | 26818.4 (14650.1) | 33030.6 (10820.6) |
| T_max_ (h), median [Min-Max] | 4 [4,4] | 24 [24,24] | 6 [4-24] | 8 [8-10] | 8 [2-8] | 8 [4-8] | 8 [4-8] | 8 [4-8] |
| t_1/2z_ (h) | 111.2 (-) | 111.6 (-) | 89.1 (23.8) | 80.7 (15.3) | 83.1 (16.6) | 87.6 (11.7) | 80.8 (19.4) | 74.1 (24.6) |
| V_z_/F (L) | 2971.7 (-) | 3137.7 (-) | 2303.3 (37.5) | 2627.1 (1416.0) | 2307.1 (724.2) | 2009.5 (706.2) | 2163.4 (719.0) | 2001.6 (613.2) |
| CL_z_/F (L/h) | 18.5 (-) | 19.5 (-) | 18.8 (4.8) | 21.6 (7.0) | 19.1 (3.8) | 16.2 (6.6) | 19.7 (8.1) | 19.9 (9.2) |
| **Steady State** |  |  |  |  |  |  |  |  |
| C_max,ss_ (ng/mL) | 77.2 (-) | 122.0 (-) | 219.0 (75.2) | 550.0 (229.2) | 635.3 (30.1) | 945.8 (189.0) | 1453.0 (825.0) | 1067.0 (216.4) |
| AUC_0-24,ss_ (h*ng/mL) | 1629.1 (-) | 2728.6 (-) | 4785.0 (1808.2) | 11332.7 (4944.0) | 12969.9 (1048.1) | 18460.0 (3291.4) | 27965.4 (13872.8) | 23180.1 (3336.5) |
| T_max,ss_ (h), median [Min-Max] | 2 [2,2] | 4 [4,4] | 8 [4-8] | 8 [8-8] | 4 [4-4] | 6 [4-8] | 4 [4-8] | 6 [4-8] |
| Ra_(Cmax)_, GM (CV%) | 4.9 (-) | 7.8 (-) | 6.4 (4.1) | 4.7 (1.2) | 4.1 (0.9) | 2.7 (1.3) | 2.8 (1.0) | 2.1 (1.4) |
| Ra_(AUC)_, GM (CV%) | 6.9 (-) | 9.6 (-) | 8.1 (4.0) | 6.0 (1.0) | 5.0 (1.0) | 3.3 (1.5) | 3.2 (1.2) | 2.7 (1.4) |

Data are expressed as mean (CV%) unless otherwise specified.

*PKCS included patients who received at least one dose of the study drug and had at least one measurable plasma concentration of the study drug in the phase 1 dose-escalation part. The percentage of AUC_inf_ obtained by extrapolation >40%, AUC_inf_, V_z_/, CL_z_/F, t_1/2z_ were not calculated after multiple doses.

AUC_0-t_ area under the plasma concentration‐time curve from dosing (time 0) to the last time point with a measurable concentration, AUC_0-24_ area under the plasma concentration‐time curve over the 24-h dosing interval, AUC_0-24,ss_ AUC_0-24_ at steady state, C_max_ maximum concentration in plasma, C_max ss_ C_max_ at steady state, CL_ss_/F apparent total body clearance at steady state, CV coefficient of variation, GM geometric mean, PKCS Pharmacokinetic concentration set, Ra _(AUC)_ accumulation ratio was calculated as the ratio of AUC_0-24,ss_ to the AUC_0-24_, Ra_(Cmax)_ accumulation ratio was calculated as the ratio of C_max,ss_ to C_max_, t_1/2z_ terminal elimination half-life, T_max_ time to maximum concentration, T_max,ss_ Tmax at steady state, Vz/F apparent volume of distribution

Table S11. The inhibitory effect of HA121-28 on protein kinases

| Protein Kinases | IC_50_(nM) | |
| --- | --- | --- |
|  | HA121-28 | Vandetanib |
| EGFR | 46.57 | 115.4 |
| KDR | 35.72 | 23.06 |
| RET | 8.72 | 10.88 |
| FGFR1 | 32.33 | 58.82 |
| FGFR2 | 20.4 | 18.38 |
| FGFR3 | 23.05 | 36.98 |
| FGFR4 | 952.7 | 1797 |
| PDGFRα | 58.69 | 42.66 |
| FLT1 | 20.93 | 25.5 |
| HER2 | 42.01 | 70.77 |
| LCK | 13.98 | 10.93 |
| EphA1 | 86.88 | 47.47 |
| SRC | 65.75 | 67.46 |
| ALK1 | 2593 | 2243 |
| PDGFRβ | 165 | 286.4 |
| FMS | 2910 | 3053 |
| FLT3 | 556.4 | 496.3 |
| AXL | 588.8 | 393.1 |
| RON | >10000 | 5976 |
| BLK | 201.3 | 96.24 |
| BTK | 445.5 | 644.9 |
| TrkA | 1363 | 1141 |
| ABL1 | 152.6 | 103.5 |
| EGFR（T790M  /L828R） | 165.9 | 295.2 |
| C-KIT | >10000 | >10000 |

Table S12. Inhibitory effect of HA121-28 on the proliferation of tumor

| Cell lines | IC_50_ (nmol /L) | |
| --- | --- | --- |
|  | HA121-28 | Vandetanib |
| ECA-109 | 6662.8±54.45 | 7400.75±189.72 |
| BEL-7402 | 9914±202.23 | 15052.5±1126.42 |
| A431 | 774.87±76.74 | 1140.07±254.88 |
| HCT116 | 1419.75±235.82 | 3194.9±687.59 |
| TT | 3825.9±91.5 | 3566.75±38.25 |
| CFPAC-1 | 207.2±38.33 | 335.6±10.04 |
| A498 | 858.5±17.88 | 1034.6±42.71 |
| A375 | 715.59±153.65 | 2191.95±317.99 |
| 8305C | 623.05±80.26 | 1249.1±96.31 |
| HT29 | 5397.3±813.03 | 10431.05±630.67 |
| SGC7901 | 4364.65±338.07 | 4365.55±440.74 |
| MDA-MB-231 | 10330.2±2013.56 | 10707.45±1871.78 |
| KYSE-150 | 1083.7±220.05 | 1504.9±135.2 |

Table S13. The efficacy of HA121-28 on human esophageal cancer KYSE-150 xenografts in nude mice

| Group | Dose | Initial Tumor Volume | Terminal Tumor Volume | RTV  X±SD | T/C |
| --- | --- | --- | --- | --- | --- |
|  | (mg/kg) | (mm^3^) | (mm^3^) |  | （%） |
| HA121-28 | 50 | 170.3±81.1 | 118.2±105.7** | 0.7±0.5** | 4.5 |
| HA121-28 | 25 | 184.3±86.9 | 411.7±178.7** | 2.5±1.8** | 16.7 |
| HA121-28 | 12.5 | 189.8±81.0 | 795.0±253.1** | 4.5±1.2** | 29.4 |
| Vandetanib | 50 | 190.6±91.9 | 273.9±189.8** | 1.3±0.6** | 8.4 |
| Vandetanib | 25 | 191.1±84.3 | 489.8±273.9** | 2.6±0.7** | 16.8 |
| Vandetanib | 12.5 | 181.0±89.4 | 847.0±247.2** | 5.2±1.9** | 34.2 |
| Cisplatin | 10 | 188.5±94.3 | 694.4±237.4** | 5.0±3.9** | 32.6 |
| Blank group | - | 175.8±82.6 | 2,464.1±636.6 | 15.2±3.2 |  |

RTV: Relative Tumor Volume; T/C: Relative Tumor Growth Rate; *P<0.05, **P<0.01, compared with the blank group

Table S14. Efficacy of HA121-28 on human esophageal cancer ECA-109 tumors

| Group | Dose | Initial Tumor Volume | Terminal Tumor Volume | RTV  **$\overline{x}$**±SD | T/C |
| --- | --- | --- | --- | --- | --- |
|  | (mg/kg) | (mm^3^) | (mm^3^) |  | （%） |
| HA121-28 | 50 | 232.6±71.1 | 491.8±77.4** | 2.2±0.4** | 21.6 |
| HA121-28 | 25 | 226.3±81.0 | 693.5±538.6*^▲^ | 3.1±1.6*^▲^ | 29.8 |
| HA121-28 | 12.5 | 219.2±70.8 | 1,684.6±1,034.3 | 7.0±2.8 | 68.6 |
| Vandetanib | 50 | 227.9±49.7 | 710.3±367.1* | 3.2±1.9* | 31.2 |
| Vandetanib | 25 | 234.5±61.2 | 1,921.8±802.2 | 8.0±2.6 | 78.3 |
| Vandetanib | 12.5 | 223.0±64.8 | 1,683.6±1,162.4 | 7.2±3.4 | 70.4 |
| Cisplatin | 8 | 242.1±54.5 | 1,580.0±952.4 | 6.4±2.9 | 62.0 |
| Blank group | - | 220.6±81.2 | 2,034.8±1,133.1 | 10.3±5.3 |  |

RTV: relative tumor volume; T/C: relative tumor growth rate; *P<0.05, **P<0.01, compared with the blank group, ▲p<0.05, compared with the vandetanib group of the same dosage

Table S15. Efficacy of HA121-28 on on human thyroid cancer TT tumors

| Group | Dose | Initial Tumor Volume | Terminal Tumor Volume | RTV  **$\overline{x}$**±SD | T/C |
| --- | --- | --- | --- | --- | --- |
|  | (mg/kg) | (mm^3^) | (mm^3^) |  | （%） |
| HA121-28 | 75 | 162.6±72.8 | 86.9±40.0** | 0.5±0.2** | 8.7 |
| HA121-28 | 50 | 167.0±96.4 | 131.9±66.5** | 0.8±0.2** | 13.6 |
| HA121-28 | 25 | 166.4±72.8 | 307.4±69.5** | 2.1±0.6** | 33.8 |
| Vandetanib | 75 | 169.0±70.4 | 100.1±62.5** | 0.5±0.2** | 8.9 |
| Vandetanib | 50 | 159.6±69.2 | 155.5±82.1** | 1.0±0.4** | 17.0 |
| Vandetanib | 25 | 163.1±65.3 | 302.4±91.1** | 2.1±1.0** | 34.1 |
| Blank group | - | 160.9±79.4 | 886.7±327.2 | 6.1±2.6 |  |

RTV: Relative Tumor Volume; T/C: Relative Tumor Growth Rate; *P<0.05, **P<0.01, compared with the blank group

Table S16. Efficacy of HA121-28 on human gastric cancer SGC-7901 tumors

| Group | Dose | Initial Tumor Volume | Terminal Tumor Volume | RTV  **$\overline{x}$**±SD | T/C |
| --- | --- | --- | --- | --- | --- |
|  | (mg/kg) | (mm^3^) | (mm^3^) |  | （%） |
| HA121-28 | 75 | 225.4±95.0 | 288.9±162.6** | 1.2±0.3** | 7.6 |
| HA121-28 | 50 | 210.2±90.8 | 478.5±307.8** | 2.0±1.1** | 12.8 |
| HA121-28 | 25 | 204.4±72.0 | 1424.3±410.0** | 7.2±1.0** | 45.3 |
| Vandetanib | 75 | 215.5±70.5 | 486.9±221.2** | 2.3±1.0** | 14.3 |
| Vandetanib | 50 | 202.9±74.7 | 690.9±259.8** | 3.6±1.7** | 23.0 |
| Apatinib | 200 | 201.2±83.7 | 793.5±324.4** | 4.2±1.2** | 26.5 |
| Blank group | - | 212.4±82.3 | 3098.7±1236.2 | 15.8±6.0 |  |

RTV: Relative Tumor Volume; T/C: Relative Tumor Growth Rate; *P<0.05, **P<0.01, compared with the blank group

Table S17. Efficacy of HA121-28 on human melanoma A375 tumors

| Group | Dose | Initial Tumor Volume | Terminal Tumor Volume | RTV  **$\overline{x}$**±SD | T/C |
| --- | --- | --- | --- | --- | --- |
|  | (mg/kg) | (mm^3^) | (mm^3^) |  | （%） |
| HA121-28 | 50 | 182.1±99.2 | 879.7±405.8** | 5.7±3.1** | 27.7 |
| HA121-28 | 25 | 164.3±90.0 | 1329.3±1001.3* | 8.7±5.9** | 42.6 |
| HA121-28 | 12.5 | 183.4±113.9 | 1860.7±1044.5 | 11.2±4.7* | 54.6 |
| Vandetanib | 50 | 160.5±97.8 | 1091.3±624.3* | 7.2±1.4** | 35.0 |
| Vandetanib | 25 | 169.7±88.7 | 1719.9±822.4 | 11.4±4.7* | 55.7 |
| Vandetanib | 12.5 | 174.4±105.1 | 2824.1±1001.2 | 22.1±17.2 | 108.1 |
| Blank group | - | 184.3±106.5 | 3380.3±1658.7 | 20.4±6.3 |  |

RTV: Relative Tumor Volume; T/C: Relative Tumor Growth Rate; *P<0.05, **P<0.01, compared with the blank group
