# Supplementary material for: Safety, pharmacokinetics and efficacy of HA121-28 in patients with advanced solid tumors and RET fusion-positive non-small-cell lung cancer: a multicenter, open-label, single-arm phase 1/2 trial
Source: Signal Transduct Target Ther. 2025 Feb 28;10:62. doi: 10.1038/s41392-025-02155-5 (PMC11868595; doi:10.1038/s41392-025-02155-5)
Supplement: Supplementary file 3 — HA121-28 Phase 2 study protocol [file 41392_2025_2155_MOESM3_ESM.pdf]

A Phase 2, Single-arm, Multicenter, Open-label Clinical Study of HA121-28 Tablets for the Treatment of RET Fusion-positive Advanced Non-small Cell Lung Cancer

Protocol Number:	HA122-CSP-004
Version No.:	V 4.0
Version Date:	December 1, 2022
Principal Investigator:	Professor Xu Ruihua
Leading Site:	Sun Yat-sen University Cancer Center
Statistical Institution:	CSPC ZhongQi Pharmaceutical Technology (Shijiazhuang) Co., Ltd.
Sponsor:	CSPC ZhongQi Pharmaceutical Technology (Shijiazhuang) Co., Ltd.
Sponsor Contact:	Xu Wen
Tel:	[redacted]

Confidentiality Statement

This document contains important confidential business information which is the property of CSPC ZhongQi Pharmaceutical Technology (Shijiazhuang) Co., Ltd.. It must not be disclosed unless required by current laws or regulations. This confidentiality requirement must be communicated to all recipients of this document and applies to all future documents that are marked as confidential.

Approval for Clinical Study Protocol -Investigator Signature Page

I have read this clinical study protocol (Protocol ID: HA122-CSP-004, Version V 4.0, Version Date: December 01, 2022), and agree to conduct the clinical study in accordance with all the terms of this protocol, current regulations, and ethical principles of the Helsinki Declaration.

Testing Facility:

Signature of Principal Investigator:

Date: ______ (MM/DD/YYYY)

Approval for Clinical Study Protocol-Sponsor Signature Page

I have read this clinical study protocol (Protocol ID: HA122-CSP-004, Version V 4.0, Version Date: December 01, 2022), and agree to conduct the clinical study in accordance with all the terms of this protocol, current regulations, and ethical principles of the Helsinki Declaration.

Sponsor: CSPC ZhongQi Pharmaceutical Technology (Shijiazhuang) Co., Ltd.

Signature of Sponsor Representative:

Date: ______ (MM/DD/YYYY)

Approval for Clinical Study Protocol-Signature Page of Statistical Institution

I have read this clinical study protocol (Protocol ID: HA122-CSP-004, Version V 4.0, Version Date: December 01, 2022), and agree to conduct the clinical study in accordance with all the terms of this protocol, current regulations, and ethical principles of the Helsinki Declaration.

Statistical Institution: CSPC ZhongQi Pharmaceutical Technology (Shijiazhuang) Co., Ltd.

Signature of Project Director:

Date: ______ (MM/DD/YYYY)

Sponsor (Emergency) Contact

In case of a medical emergency, the project manager of the clinical study team of the sponsor can be contacted. If the project manager cannot be reached, the drug safety emergency contact person listed in the table below can be contacted.

	**Name**	**Tel.**
Project Manager	[redacted]	[redacted]
Drug Safety Emergency Contact	[redacted]	[redacted]

Table of Contents

Approval for Clinical Study Protocol-Investigator Signature Page 2

1. Protocol Synopsis 3

1.1. Summary 3

1.2. Study Schedule 10

2. Introduction 13

2.1. Study Rationale 13

2.2. Background Introduction 13

2.2.1. Preclinical Pharmacodynamic Studies 13

2.2.2. Preclinical Pharmacokinetic Studies 16

2.2.3. Preclinical Toxicology Studies 17

2.2.4. Clinical Studies 18

2.3. Risk/Benefit Assessment 19

2.3.1. Known Potential Risks 19

2.3.2. Known Potential Benefits 19

2.3.3. Assessment of Potential Risks and Benefits 19

3. Study Objectives and Endpoints 20

4. Study Design 20

4.1. Overall Design 20

4.2. Rationale for Dose Selection 21

4.3. End of Study Definition 22

5. Study Population 22

5.1. Inclusion Criteria 22

5.2. Exclusion Criteria 23

5.3. Screening Failure 25

6. Study Intervention 25

6.1. Study Treatment .... **错误!未定义书签。**

6.1.1. Study Treatment Description 25

6.1.2. Dosing Regimen of Study Treatment 26

6.1.3. Dose Modification Principles 26

6.2. Preparation/Handling/Storage/Responsibilities regarding Study Treatment 28

6.2.1. Drug Receipt and Accountability 28

6.2.2. Dosage Form, Appearance, Packaging and Labeling 29

6.2.3. Drug Product Storage and Stability 29

6.3. Methods to Mitigate Bias: Randomization and Blinding 30
6.4. Compliance with Study Intervention 30
6.5. Concomitant Medications and Treatments 30
6.5.1. Prohibited and/or Restricted Medications and Treatments 30
6.5.2. Permitted and/or Cautionary Medications and Treatments 30
7. Discontinuation and Withdrawal Criteria 31
7.1. Discontinuation Criteria 31
7.2. Withdrawal Criteria 32
7.3. Lost to Follow-up 32
8. Study Assessments and Procedures 33
8.1. Tumor Imaging Assessments 33
8.2. Safety and Other Assessments 34
8.3. Adverse Events and Serious Adverse Events 36
8.3.1. Definition 36
8.3.2. Adverse Event Assessment Category 38
8.3.3. Causality 38
8.3.4. Action Taken with Study Treatment 40
8.3.5. Other Specific Treatment for Adverse Events 41
8.3.6. Outcome 41
8.3.7. Collection, Recording and Evaluation of Adverse Events 41
8.3.8. Reporting of Serious Adverse Events 42
8.3.9. Pregnancy 43
8.4. Pharmacokinetic Assessment 44
9. Statistical Considerations 45
9.1. Statistical Assumptions 45
9.2. Sample Size Estimation 45
9.3. Analysis Population 46
9.4. Statistical Analysis 46
9.4.1. General Methods 46
9.4.2. Participant Disposition 46
9.4.3. Demographic and Baseline Analyses 47
9.4.4. Efficacy Analysis 47
9.4.5. Pharmacokinetic Analysis 47
9.4.6. Safety Analysis 48

9.4.7. Analysis of Concomitant And Non-drug Therapies 49
9.4.8. Analysis of Drug Exposure and Compliance 49
9.4.9. Planned Interim Analyses 49
9.4.10. Subgroup Analysis 50
9.4.11. Multiplicity Issues 50
9.4.12. Exploratory Analyses 50
10. Supporting Documentation and Operational Considerations 50
10.1. Regulatory, Ethical, and Study Regulatory Considerations 50
10.1.1. Informed Consent Process 50
10.2. Study Suspension and Discontinuation 51
10.3. Confidentiality and Privacy 51
10.4. Future Use of Data 52
10.5. Quality Assurance and Quality Control 52
10.6. Data Processing and Record Retention 53
10.6.1. Data Collection and Management Responsibilities 53
10.6.2. Retention of Study Records 54
10.7. Protocol Violation 55
10.8. Study Publication and Data Sharing Policy 55
11. References 56
12. Appendix 57
Appendix I: ECOG Performance Score 57
Appendix 2: New York Heart Association (NYHA) Classification in Heart Failure. ..... 错误!未定义书签。
Appendix 3: Excerpt from Response Evaluation Criteria in Solid Tumors (RECIST 1.1) 59

List of Abbreviations

Abbreviation	Definition
ADR	Adverse Drug Reaction
AE	Adverse Event
ALK	anaplastic lymphoma kinase
ALT	Alanine Aminotransferase
ANC	Absolute Neutrophil Count
APTT	Activated Partial Thromboplastin Time
AST	Aspartate Aminotransferase
AUC	Area Under Curve
CI	Confidence Interval
CL	Plasma Clearance
CNS	central nervous system
CR	Complete Response
CRF	Case Report Form
CRO	Contract Research Organization
CT	Computed Tomography
CTCAE	Common Terminology Criteria for Adverse Events
DCR	Disease Control Rate
DMP	Data Management Plan
DNA	Deoxyribonucleic Acid
DOR	Duration of Response
ECG	Electrocardiogram
ECOG	Eastern American Cancer Collaboration
eCRF	Electronic Case Report Form
EDC	Electronic Data Capture System
EOT	End of Treatment
FAS	Full Analysis Set
FDG-PET	Fluorodeoxyglucose-positron emission tomography
GCP	Good Clinical Practice
Hb	Hemoglobin
HBcAb	Hepatitis B Core Antibody
HBsAg	Hepatitis B Surface Antigen
HBV	Hepatitis B Virus
HCG	Human chorionic gonadotropin
HCV	Hepatitis C Virus
HIV	Human Immunodeficiency Virus
HNSTD	Highest non-severely toxic dose
ICF	Informed Consent Form
INR	International Normalized Ratio
IRB	Institutional Review Board
IRC	Independent Review Committee

MRI	Magnetic Resonance Imaging
MTD	Maximal Tolerable Dose
NCI	National Cancer Institute
NSCLC	Non-Small Cell Lung Carcinoma
NYHA	New York Heart Association
ORR	Objective Response Rate
OS	Overall Survival
PD	Progressive Disease
PD-1	Programmed Death-1
PD-L1	Programmed Death Ligand-1
PFS	Progression-Free-Survival
PLT	Platelet count
PR	Partial Response
PT	Prothrombin Time
qd	Once a day
RECIST	Response Evaluation Criteria in Solid Tumor
RNA	Ribonucleic Acid
SAE	Serious Adverse Event
SD	Stable Disease
SOC	System Organ Class
SOPs	Standard Operating Procedure
SS	Safety Analysis Set
SUSAR	Suspected Unexpected Serious Adverse Reaction
t1/2	Half-time
TEAE	Treatment Emergent Adverse Event
ULN	Upper Limit of Normal
WHO	World Health Organization

1. Protocol Synopsis

1.1. Summary

**Protocol Number**	HA122-CSP-004
**Protocol Name**	A Phase 2, Single-arm, Multicenter, Open-label Clinical Study of HA121-28 Tablets for the Treatment of RET Fusion-positive Advanced Non-small Cell Lung Cancer
**Version No./Version Date**	V 4.0/December 1, 2022
**Study Phase**	Phase 2
**Registration Category**	Chemicals Category 1
**Sponsor**	CSPC ZhongQi Pharmaceutical Technology (Shijiazhuang) Co., Ltd.
**Principal Investigator**	Xu Ruihua
**Leading Site**	Sun Yat-sen University Cancer Center
**Study Objectives and Endpoints**	**Primary objective:**<br>To evaluate the efficacy of HA121-28 tablets in patients with RET fusion-positive advanced non-small cell lung cancer (NSCLC).<br>**Secondary objectives:**<br>• To evaluate the safety of HA121-28 tablets in patients with RET fusion-positive advanced NSCLC.<br>• To evaluate the pharmacokinetic (PK) characteristics of HA121-28 tablets in patients with RET fusion-positive advanced NSCLC.<br>**Primary endpoint:**<br>Objective response rate (ORR), evaluated by an independent review committee (IRC) according to Response Evaluation in Solid Tumors Version 1.1 (RECIST 1.1) criteria.<br>**Secondary endpoints:**<br>• ORR evaluated by the investigator based on RECIST 1.1 criteria.<br>• The disease control rate (DCR), progression-free survival (PFS), and duration of response (DOR) were evaluated by the investigator and the IRC according to the RECIST 1.1 criteria.<br>• Overall survival (OS);<br>• Treatment-emergent adverse events (TEAEs) and serious adverse events (SAEs) during the treatment period.<br>• Plasma concentrations of HA121-28.

**Study Design**	This is a single-arm, open-label, multicenter, phase 2 clinical study. The 83 patients with advanced NSCLC who have progressed after at least one line standard of care will be enrolled in the study. The aim of the study is to evaluate the efficacy and safety of HA121-28 tablets in patients with unresectable locally advanced or metastatic RET fusion-positive NSCLC.<br>After obtaining informed consent from the patients or their guardians, patients will be screened within 28 days prior to administering the study treatment.<br>Eligible patients will receive HA121-28 tablets at a recommended starting dose of 450 mg once daily for 21 consecutive days, followed by a 7-day drug-free period, constituting a 28-day treatment cycle. The treatment will be continued until disease progression, intolerable toxicity, death, or any other reason for interruption of the treatment. During the treatment period, investigators can adjust the dose based on tolerability. Refer to Section 6.1.3 for the dose adjustment. Safety assessments will be conducted every cycle on day 28 (±3 days). Tumor assessments will be performed using computed tomography/magnetic resonance imaging (CT/MRI) according to RECIST 1.1 every 8 weeks (±7 days) for the first 40 weeks and then every 12 weeks (±7 days) thereafter until disease progression, initiation of new anti-tumor therapy, death, or patient withdrawal from the study (whichever occurs first).<br>For patients who discontinue treatment and/or withdraw from the study during the study period, an end-of-treatment/withdrawal visit should be conducted within 14 days after the last dose, and a safety follow-up visit should be conducted on day 28 after the last dose. Patients will be given telephone or on-site visits every 12 weeks (±7 days) after completing the last tumor assessment to determine their survival status and subsequent anti-tumor therapy.
**Study Population**	**Inclusion Criteria**<br>1. Voluntarily participate in this study and sign the informed consent form;<br>2. Aged 18 ~ 75 years old (inclusive), male or female;<br>3. Patients with histologically or cytologically confirmed unresectable locally advanced or metastatic NSCLC;<br>4. Centre laboratories with College of American Pathologists (CAP) or Clinical Laboratory Improvement Amendments (CLIA) certification determined RET gene fusion using the "Next-generation" sequencing (NGS) method;<br>5. Progressive disease after at least one line of the standard of care (including disease that progressed during the adjuvant treatment period or within 6 months of the end of adjuvant therapy);<br>6. At least one measurable lesion according to RECIST 1.1 (for lesions previously treated with radiation, the lesion can be marked as a measurable lesion only if

	there is clear disease progression after radiotherapy);<br>7. Eastern Cooperative Oncology Group (ECOG) performance status (PS) score 0-1;<br>8. Adequate organ function, defined as:<br>• Neutrophil count (ANC) ≥ 1.5 × 10^9/L (no G-CSF for WBC-elevating therapy within 2 weeks prior to the laboratory test);<br>• Platelet count (PLT) ≥ 75 × 10^9/L (not receiving platelet transfusion or other drugs to promote platelet production within 2 weeks prior to the laboratory test);<br>• Hemoglobin (Hb) ≥ 90 g/L; (not receiving red blood cell transfusion or erythropoiesis-stimulating drugs within 2 weeks prior to the laboratory test);<br>• Alanine aminotransferase (ALT) and aspartate aminotransferase (AST) ≤ 3 × upper limit of normal (ULN) (≤ 5.0 × ULN for patients with liver metastases);<br>• Serum total bilirubin (TBIL) ≤ 1.5 × ULN;<br>• Serum creatinine ≤ 1.5 × ULN;<br>• Albumin ≥ 30 g/L;<br>9. Male and female patients of childbearing age agree to take effective contraceptive measures during treatment and within 6 months after the last dose of treatment.<br>**Exclusion Criteria**<br>1. Have a documented carcinogenic-driver gene alteration other than RET in NSCLC, including activating EGFR, BRAF, or KRAS mutation, MET exon 14 skipping mutation or high-level amplification, and ALK, ROS1, or NTRK1/2/3 gene fusions;<br>2. Prior treatment with selective RET inhibitors (including investigational RET inhibitors, such as LOXO-292, BLU-667, RXDX-105, vandetanib, cabozantinib, etc.);<br>3. Patients who have previously received any anti-tumor therapy within 4 weeks before the first use of the study treatment (including but not limited to chemotherapy, radiotherapy and targeted therapy, etc.); Patients treated with traditional Chinese medicine or Chinese patent medicine with anti-tumor indications or local palliative radiotherapy for the pain relief of bone metastasis should have a two-week period of detoxification;<br>4. Abnormal coagulation function (INR > 1.5 or APTT > 1.5 × ULN), bleeding tendency (such as an active peptic ulcer), or current treatment with

	thrombolytic or anticoagulant drugs;<br>5. Urine protein ≥ ++ and 24 h urine protein > 1.0 g;<br>6. Major surgical procedures within 4 weeks before the first dose of the study treatment or are expected to undergo major surgery during the study;<br>7. Symptomatic central nervous system (CNS) metastases or imaging evidence for progressive neurological symptoms. Patient requires treatment with corticosteroids for CNS disease, the dose must be stable for two weeks prior to the first dose of the study treatment;<br>8. Presence of poorly controlled pericardial, pleural, or peritoneal effusion;<br>9. Interstitial pneumonia, drug-induced pneumonitis, radiation pneumonitis requiring steroid therapy (except for stable radiation pneumonitis);<br>10. Serious cardiovascular disease, such as greater than New York Heart Association (NYHA) grade 2 heart failure, unstable angina, serious arrhythmia, myocardial infarction or stroke within 6 months prior to the first dose of study treatment, poorly controlled hypertension (defined as systolic blood pressure > 150 mmHg or diastolic blood pressure > 100 mmHg on multiple measurements under drug control);<br>11. Patients who meet any of the following criteria will be excluded:<br>• QT interval (QTcF) value ≥ 470 ms for females and ≥ 450 ms for males; or congenital long QT syndrome, taking drugs known to prolong QT interval, or family history of long QT syndrome;<br>• Resting ECG shows any clinically significant abnormalities in rhythm, conduction, or morphology that require clinical intervention;<br>• Cardiac ejection fraction less than 50%;<br>12. Patients with active hepatitis B virus or hepatitis C virus infection:<br>• HBsAg positive with HBV DNA higher than the upper limit of normal range of the study center;<br>• HCV antibody positive with HCV RNA higher than the upper limit of normal range of the site;<br>13. Human immunodeficiency virus infectors (HIV positive);<br>14. Inability or severe dysphagia;<br>15. Patients who have suffered from or are complicated with any other malignant tumor within 5 years (except radically resected skin basal cell carcinoma, skin squamous cell carcinoma, superficial bladder cancer, local prostate cancer, in situ cervical cancer, or other carcinoma in situ);<br>16. Presence of any severe and/or uncontrolled disease that may affect the drug evaluation in the judgment of the investigator, including but not limited to: life-

	threatening autoimmune system diseases, drug abuse, severe nervous system diseases (such as epilepsy, dementia, etc.); history of severe mental disorders; severe infection, hypothyroidism, etc.;<br>17. Pregnant or lactating women;<br>18. Other conditions that are unsuitable for participation in the study, in the opinion of the investigator.
**Study Treatment**	Dosage Form: Tablet<br>Specification: 25 mg/100 mg/150 mg/200 mg.
**Study Intervention**	The recommended starting dose is 450 mg once daily for 21 consecutive days followed by a 7-day drug-free period, with each 28-day period considered as one cycle. The treatment will be continued until disease progression, intolerable toxicity, death, or any other reason for discontinuation of treatment occurs.<br>Investigators in the study may adjust the dosage based on the patients' tolerance, please refer to Section 6.1.3 for details.
**Sample Size**	This study is a single-arm design, with the primary efficacy endpoint of ORR. From a clinical perspective, an ORR of 30% or higher for the second-line or higher treatment of RET fusion NSCLC is considered clinically significant. Assuming that the ORR of HA121-28 tablets for the second-line or higher treatment of RET fusion NSCLC is 45%, with a one-sided significance level of 0.025 and a power of 80%, the sample size is estimated to be 83 using the single-group target value method. The study will be considered positive (a success) when at least 34 patients show a response, the corresponding ORR is 41%, and the lower limit of the 95% confidence interval can be greater than 30%.

ORR Assumptions	Standard of Control (Lower 95% confidence interval)	Sample Size	Minimum Number of Responses	Response Boundary	95% Confidence Interval (Clopper-Pearson)
45%	30%	83	34	41.0%	(30.28%, 52.31%)

Sample size estimation will be performed using nQuery software (v8).

**Statistical Analysis**	Analysis Set:<br>Full Analysis Set (FAS): All patients who are enrolled and received at least one dose of the study treatment. FAS is used for the demographic, baseline characteristics, and efficacy analyses.<br>Per Protocol Set (PPS): All patients in the FAS who have not committed any major protocol violations. PPS is used for sensitivity analysis.<br>Safety Set (SS): All patients who have received at least one dose of the study treatment.<br>Pharmacokinetic Concentration Set (PKCS): All patients who have received at least

	one dose of the study treatment, and have at least one assessable PK concentration without any significant deviation that significantly affects the drug concentration.<br>General rules:<br>The statistical analysis of this study will be conducted using SAS 9.4 or higher version.<br>For categorical variables, the statistical description will be expressed in terms of the number of cases and percentages. For continuous variables, the statistical description will include the number of cases, mean, standard deviation, median, lower quartile, upper quartile, minimum value, and maximum value. Unless otherwise specified, a two-sided test will be used with a significance level of 0.05, and a 95% confidence interval will be used for parameter estimation.<br>**Efficacy analysis**:<br>Primary endpoint:<br>Analyses based on FAS and PPS. The 95% confidence interval of ORR assessed by IRC will be calculated using the Clopper-Pearson method.<br>Secondary efficacy endpoints:<br>Analyses will be performed based on the FAS. The median time and 95% CI for PFS, DOR, and OS assessed by IRC and investigator will be calculated using the Kaplan-Meier, and corresponding survival curves will be drawn.<br>The 95% confidence interval of ORR assessed by the investigator and DCR assessed by IRC and investigator will be calculated using the Clopper-Pearson method.<br>**Safety analysis:**<br>Analyses will be performed based on SS. Summarize the number and percentage of TEAEs, treatment-related TEAEs, SAEs, drug-related SAE, TEAE leading to discontinuation, and drug-related TEAE leading to discontinuation during the treatment.<br>Summarize the number and percentage of occurrences of TEAEs, treatment-related TEAEs, SAEs, treatment-related SAEs, TEAEs leading to discontinuation, and treatment-related TEAEs leading to discontinuation as per SOC/PT.<br>Summarize the incidence and percentage of TEAEs, treatment-related TEAEs, SAEs, treatment-related SAEs, TEAEs leading to discontinuation, and treatment-related TEAEs leading to discontinuation categorized by SOC/PT and severity.<br>Provide a detailed list of TEAEs/Treatment-related TEAEs categorized by SOC/PT and severity.<br>Laboratory test measures: Provide a cross-tabulation for clinical judgment before and after treatment, and perform descriptive and longitudinal analyses of the measured values and changes from baseline of laboratory test measures before and

	after treatment. Provide a detailed list of examination results.<br>Electrocardiogram: Provide a cross-tabulation of clinical judgments before and after treatment, and perform descriptive and longitudinal analyses of the measured values and changes from baseline of electrocardiogram measures at each time point before and after treatment. Provide a detailed list of the examination results.<br>Physical examination: Provide a cross-tabulation of clinical assessments before and after treatment, and provide a detailed list of examination results.<br>Vital signs: Descriptive and longitudinal analyses will be performed on the measured values and changes from baseline of various measures before and after treatment. A detailed list of examination results before and after treatment will also be provided.<br>**PK analysis:**<br>Analyses will be performed based on PKCS. PK samples will be collected at the designated time points according to the protocol, and the plasma concentration of HA121-28 will be measured. The HA121-28 concentrations will be presented in the table and analyzed by descriptive statistics.<br>If there is sufficient data, the blood HA121-28 concentrations of the patients will be used for population pharmacokinetic (PPK) analysis using a nonlinear mixed-effects model (NONMEM). The analysis report and details can be found in a separate report.<br>The specific statistical analysis contents are detailed in the statistical analysis plan.
**Study Duration**	Approximately 2 years

1.2. Schedule of Assessment

Test Period	Screening		Treatment Period			End of Treatment/Withdrawal Visit (EOT) [3]	Safety Follow-up Period	Follow-up
Visit time	-28~-1	-7~-1	C1D1	C1D15[1]	CnD28[2]	Within 14 days of the last dose	Day 28 after the last dose	Every 12 weeks
Window	N/A	N/A	N/A	± 1 day	± 3 days	N/A	± 3 days	± 7 days
Informed Consent	×							
Demographic data [4]	×							
Height/Weight [5]	×				×	×		
Tumor History and Treatment History [6]	×							
Tumor tissue samples or blood collection [7]	×							
Personal and other past medical history or concomitant diseases [8]	×							
Physical examination	×				×	×	×	
ECOG performance score	×				×	×		
Vital signs		×			×	×	×	
Hematology		×			×	×	×	
Blood chemistry		×			×	×	×	
Urinalysis [9]		×			×	×	×	
Coagulation		×				×		
Serum virology	×							
Pregnancy test [10]		×				×		
12-lead ECG [11]		×		×	×	×	×	
Echocardiography	×					×		

PK blood sampling [12]			×	×	×	×		
Tumor Imaging Assessments [13]	×			**Every 8 weeks ± 7 days from Week 1 to Week 40, and every 12 weeks ± 7 days thereafter, relative to Cycle 1 Day 1.**				
Concomitant Medications/Treatments [14]		×						
Adverse events (AEs) [15]	×							
Survival status and subsequent anti-tumor regimens [16]								×

Note: All examinations scheduled in this study are mandatory requirements for the clinical study. If the investigator has a reasonable clinical need, they may add relevant examination items and frequency during the study period. All patients must sign an informed consent form before any study-related procedures are performed. For reasonable clinical needs, retesting is allowed during the screening period, and the final examination result before enrollment will be used to determine the inclusion/exclusion criteria. For patients who have already signed the informed consent form, re-screening is allowed for reasonable reasons.

[1] The 12-lead electrocardiogram examination on day 15 of cycle 1 conducted at an external hospital will be allowed.
[2] If the study treatment is discontinued for ≥7 days for any reason during the treatment period, the patient will resume the drug upon recovery and proceed directly to the next cycle.
[3] If the treatment has ended/the patient has withdrawn from the study, and the assessments conducted during the end-of-treatment visit overlap with the safety assessments conducted during the previous visits, there is no need to repeat the assessments.
[4] Demographic data: gender, date of birth, age, ethnicity.
[5] Height/weight: Height and weight will be measured during the screening period, and only weight will be measured during subsequent visits.
[6] Tumor history and treatment history: Collect information on pathological diagnosis time, clinical stage, pathological type, presence of brain metastasis, etc. Also collect information on previous tumor treatment history, radiotherapy history, surgical treatment history, and previous treatment outcomes. Additionally, collect information on RET gene fusion.
[7] Collection of tumor tissue samples or blood: This is an optional requirement. Patients may provide previous genetic testing reports for the sponsor's review and confirmation. For those who do not meet the requirements or do not have previous genetic testing reports, sufficient tumor tissue samples (at least 5 slices) and 2ml peripheral blood control samples need to be provided during the screening period. If it is difficult to obtain tumor tissue samples, blood samples (8-10 ml) can also be accepted for RET gene testing. The sample and operational requirements for genetic testing are detailed in the "Central Laboratory Operation Manual".
[8] Personal and medical history: Patient's smoking and quitting history, medication allergies, medical history or coexisting conditions within the past year.
[9] Urinalysis: If the screening period urine protein is ≥++, further quantitative measurement of 24-hour urine protein should be performed. During the treatment period and follow-up period, it may be considered whether to perform further quantitative measurement of 24-hour urine protein

	according to the actual situation of the patients.<br>[10] Pregnancy Testing: Women of childbearing age should undergo pregnancy testing (excluding those who have undergone sterilization, oophorectomy and/or hysterectomy), and both blood HCG pregnancy testing and urine HCG pregnancy testing are acceptable. Women in menopause who have not yet reached postmenopausal status (12 consecutive months of amenorrhea) are considered to be capable of reproduction.<br>[11] Twelve-lead electrocardiogram: During the screening period, two 12-lead electrocardiogram tests should be performed within 7 to 1 day(s) before baseline. The average of the QTcF values from the two tests will be used as the baseline. If any of the test results meet the exclusion criteria (11), the patient will not be enrolled in the study.<br>[12] PK Sample Collection: For patients enrolled after the execution date of the revised protocol (V3.0), blood samples for PK analysis should be collected at any time point on C1D1 4-8 hours after dosing, before (-30min) and 4-8 hours after dosing on C1D15 (±1 day), and at any time point on C1D28 (±3 days), C2D28 (±3 days), C4D28 (±3 days), and C6D28 (±3 days). For patients who are enrolled before this date and have not terminated treatment, blood samples for PK analysis can be collected at any time point during any two visits of C1D15 (±1 day), before (-30min), and 4-8 hours after dosing (if applicable), and CnD28 (±3 days). 3ml of blood should be collected each time, and the time of dosing and actual sampling time should be recorded. If a patient terminates treatment for any reason during the study period, a blood sample can be collected for PK analysis on the day of the EOT visit as appropriate.<br>[13] Tumor imaging evaluation: Tumor imaging evaluation should be conducted every 8 weeks (±7 days) from C1D1 until week 40, and then every 12 weeks (±7 days) thereafter, until disease progression, initiation of new anti-tumor therapy, death, or patient withdrawal from the study (whichever occurs first). Patients judged as CR and PR must undergo efficacy confirmation at least 4 weeks later.<br>[14] Concomitant medication/treatment: Collect all concomitant medication/treatment from patients during the 28 days prior to the first drug administration until the safety follow-up period. For drug treatment, record the generic name, single dose, frequency of administration, reason for treatment, start date, and end date. For non-drug treatment, record the treatment name, start and end dates.<br>[15] AEs: Collection of AEs should be conducted from the signing of the informed consent form to the end of the safety follow-up period. If a serious adverse event (SAE) occurs after the safety follow-up period and the investigator considers that there is a reasonable causal relationship between the SAE and the study drug, it should be reported to the sponsor according to the SAE reporting process.<br>[16] After the final tumor imaging assessment, the patients will be contacted by phone or in-person every 12 weeks (±7 days) to collect information on their survival status and subsequent anti-tumor treatment.

2. Introduction

2.1. Study Rationale

Lung cancer is the most common cancer in China. According to the 2015 cancer incidence and mortality data released by the National Cancer Center, the world age-standardized incidence rate of malignant tumors in the lung is 35.92/100,000, and the world age-standardized mortality rate is 28.02/100,000. Lung cancer can be divided into non-small cell lung cancer (NSCLC) and small cell lung cancer, with NSCLC accounting for about 85%. The frequent translocation of the RET gene and its fusion partners CCDC6, KIF5B, and NCOA4 is seen in 1%-2% of lung adenocarcinomas and is more common in younger patients and never-smokers.

Currently, the only approved targeted therapy for RET fusion gene-positive NSCLC in China is pralsetinib, which has just been approved and has not yet changed clinical practice in China. Therefore, the current clinical practice is mostly according to the treatment of non-squamous NSCLC with a negative driver gene. The effective rate of second-line and above treatments for non-squamous NSCLC in China is less than 20%. There is an unmet clinical need for the development of new molecular targeted drugs for advanced RET fusion NSCLC. HA121-28 is a multi-target tyrosine kinase inhibitor mainly targeting RET and preliminary efficacy has been observed in RET fusion NSCLC in a phase 1 clinical exploratory study. Based on the safety and efficacy data of HA121-28 tablets, the National Medical Products Administration Drug Evaluation Center has communicated and confirmed that a single-arm study should be conducted to further evaluate the efficacy of HA121-28 tablets in second-line and above treatment of RET fusion NSCLC.

2.2. Background

HA121-28 is a multi-target tyrosine kinase inhibitor. At the molecular level, it strongly inhibits the activity of tyrosine kinases such as RET, KDR, EGFR, FGFR1-3, FLT-1, HER-2, LCK, EphA1, and SRC. It has the lowest IC_{50} value for RET.

2.2.1. Preclinical Pharmacodynamic Studies

HA121-28 is a multi-target tyrosine kinase inhibitor. At the molecular level, it has strong

inhibitory effects on the tyrosine kinase activity such as RET, KDR, EGFR, FGFR1-3, FLT-1, HER-2, LCK, EphA1, and SRC, [redacted]

The IC_{50} values of HA121-28 for inhibiting CFPAC-1, A498, A375, 8305C, and A431 cell lines in vitro range from 0.1-1 μM, with values of 207.2±38.33, 858.5±17.88, 715.59±153.65, 623.05±80.26, and 774.87±76.74 nmol/L, respectively. The best inhibitory effect was observed on CFPAC-1 cells. The IC_{50} values for inhibiting ECA-109, HCT116, TT, HT29, SGC-7901, and KYSE-150 cell lines in vitro range from 1-10 μM, with values of 6662.8±54.45, 1419.75±235.82, 3825.9±91.5, 5397.3±813.03, 4364.65±338.07, and 1083.7±220.05 nmol/L, respectively. The IC_{50} values of inhibiting BEL-7402 and MDA-MB-231 cell lines in vitro range from 10-100 μM. [redacted]

In an *in vitro* model of human skin squamous cell carcinoma A431 cells stimulated with 10 ng/mL EGF, both HA121-28 and vandetanib significantly inhibited the activation of EGFR by EGF stimulation and its downstream signaling protein ERK induced. The inhibitory effect was concentration-dependent, and the activity of HA121-28 was higher than that of vandetanib. At all points of time, the expression of EGFR and ERK was not significantly affected. In an *in vitro* model of human HUVEC cells stimulated with 50 ng/mL VEGF165, both HA121-28 and vandetanib significantly inhibited the activation of VEGFR and its downstream signaling protein ERK. The inhibitory effect was concentration-dependent, and the activity of HA121-28 was higher than that of vandetanib. At all points of time, the expression of VEGFR and ERK was not significantly affected. In an *in vitro* model of human thyroid cancer TT cells, both HA121-28 and vandetanib significantly inhibited the activation of RET and its downstream signaling protein ERK. The inhibitory effect was concentration-dependent, and the activity of HA121-28 was higher than that of vandetanib. At all points of time, the expression of RET and ERK was not significantly affected. In an *in vitro* model of human HUVEC cells stimulated

with 50 ng/mL $VEGF_{165}$, both HA121-28 and vandetanib, with IC50 values of 65.44 and 117.21 nM respectively, significantly inhibited the proliferation of HUVEC cells induced by $VEGF_{165}$, and the inhibitory effect was concentration-dependent. HA121-28 was able to inhibit arterial ring angiogenesis in rats and VEGF- and FGF-induced mouse matrigel angiogenesis and its inhibitory activity was equivalent to or better than that of vandetanib, indicating that HA121-28 has anti-angiogenic effects.

In vivo, HA121-28 has anti-tumor activity against human esophageal cancer ECA109 and KYSE150, human thyroid cancer TT, human melanoma A375, and human gastric cancer SGC7901 cell lines. In the KYSE150(human esophageal) and TT(thyroid cancer) models, tumor shrinkage was observed at a dose of 50 mg/kg. HA121-28 has a broad-spectrum anti-tumor effect and can cause partial tumor shrinkage in all the above tumor models in nude mice. Vandetanib also has good efficacy against the above tumor models, but its efficacy is weaker than HA121-28. After oral administration to mice, HA121-28 exhibits linear pharmacokinetic (PK) profiles in both plasma and tumor tissue within the dose range of 12.5-50 mg/kg. The PD experiment results show that there is a clear dose-response relationship between the pharmacodynamic index of tumor-bearing mice and the dose of HA121-28 within the dose range of 12.5-50 mg/kg. The drug concentration of HA121-28 in tumor tissue is higher than that in plasma, which is 4-6 times higher in AUC_{0-72} tumor tissue than in AUC_{0-72} plasma. The elimination of HA121-28 in tumor tissue is significantly slower than in plasma, with an MRT of 17-19 h, which is beneficial for the drug to exert its effect at the tumor area. HA121-28 at doses of 25 and 50 mg/kg has inhibitory effects on the activity of EGFR and its downstream signaling protein ERK. The inhibitory effect of HA121-28 on the EGFR signaling pathway begins to appear 2 hours after a single dose and lasts for 72 h (EGFR) and 24 h (ERK), respectively. Therefore, the level of kinase inhibition in tumor tissue is positively correlated with the tissue drug concentration.

In summary, HA121-28 is a multi-target kinase inhibitor. At the molecular level, it has strong inhibitory effects on the tyrosine kinase activity of RET, KDR, EGFR, FGFR1-3, FLT-1, HER-2, LCK, EphA1, SRC, etc.. Its inhibitory activity on multiple kinases is stronger than that of vandetanib. HA121-28 has good inhibitory effects on various tumor cell lines. It can inhibit the

signaling pathways of EGFR, VEGFR, and RET at the cellular level. HA121-28 can also inhibit angiogenesis. It has significant therapeutic effects on various human tumor xenografts in nude mice. In conclusion, HA121-28 has demonstrated strong *in vivo* and *in vitro* anti-tumor effects, with activity superior to that of vandetanib.

2.2.2. Preclinical Pharmacokinetic Studies

Absorption

HA121-28 was orally administered to rats, dogs, and monkeys, and showed moderate absorption rate and good systemic exposure. C_{max} and $AUC_{0\text{-}96\,h}$ were positively correlated and proportional to the dose. Parameters such as V_d, CL, and $t_{1/2}$ did not significantly change with increasing doses, indicating linear PK characteristics. The oral bioavailability in rats, dogs, and monkeys was 93.8%, 56.0%, and 64.4%, respectively. Tmax ranged from 2.17±0.753 h to 6.75±1.5 h across the different species.

HA121-28 had a high binding rate with plasma proteins in rats, mice, dogs, monkeys, and humans. The order of plasma protein binding rate from high to low was: mice (97.2±0.266%) > rats (92.4±1.28%) ≈ dogs (91.9±0.857%) ≈ monkeys (92.5±0.331%) ≈ humans (90.1±1.05%), there was no concentration dependence.

Distribution

Tissue distribution studies in rats showed that HA121-28 was widely distributed in tissues, and the parent drug could be detected in all tissues studied. The exposure levels ($AUC_{0\text{-}72\,h}$) in various tissues were higher than in plasma, with the highest levels found in the spleen, lungs, and adrenal glands (≈200 times plasma) followed by the liver, kidneys, and ovaries (≈100 times plasma), and then the uterus, testes, thymus, intestine, thyroid, heart, and stomach (≈20~60 times plasma). The exposure levels in the brain, adipose tissue, and muscles were lower (≈3~9 times plasma), and in the bone marrow, it was slightly higher than plasma (≈1.3 times plasma). The level of tumor tissue $AUC_{0\text{-}72}$ of the tumor-bearing mice was 4-6 times higher than that in the plasma $AUC_{0\text{-}72}$.

After intragastric administration to rats, the distribution peak of HA121-28 was reached at 0.5 h post-dose in the liver, stomach, and intestines and at 24 h post-dose in the testes. In most tissues, the content of HA121-28 reached the distribution peak at 4 h and gradually decreased

thereafter. At 72 h post-dose, the content of HA121-28 in most tissues had decreased to 1/10 to 1/70 of the distribution peak, except for the adrenal glands, uterus, and testes, where the content of HA121-28 decreased to 1/8, 1/5, and 1/1.3 of the distribution peak, respectively.

Metabolism

In addition to the parent compound, 7, 7, and 4 metabolites were found in the plasma of rats, dogs, and monkeys, respectively. The main metabolic pathways include N-dealkylation, N-oxidation, O-dealkylation, dehalogenation, monooxygenation, glucuronidation, methylation, and the combination of different metabolic pathways.

[redacted]

Excretion

After administering HA121-28 via gastric lavage, the proportion of excretion through various routes in its original form was found to be feces (27.5%) > bile (3.91%) > urine (2.18%). The proportion of HA121-28 excreted through the intestines with feces was much greater than that excreted through the kidneys.

The total excretion rate of HA121-28 with parent form through urine and feces was 29.7%, and the absolute bioavailability of oral administration in rats was 93.8%, indicating that the clearance of HA121-28 absorbed into the body was mainly through metabolic forms.

2.2.3. Preclinical Toxicology Studies

Safety Pharmacology

Safety pharmacology results showed that a single oral dose of HA121-28 at doses of 30, 100, and 300 mg/kg did not affect respiratory function and neurological behavior in rats. In conscious, unrestrained Beagle dogs, a single oral dose of HA121-28 at doses of 5, 10, and 15 mg/kg, as well as 10 and 15 mg/kg of the marketed drug fentanyl, caused mild prolongation of the Q-T interval and corrected Q-T interval. The degree of corrected Q-T interval prolongation caused by HA121-28 at the same dose was comparable to that of fentanyl.

Single-dose Toxicity

Results of a single-dose toxicity study showed that administering HA121-28 orally via gavage to SD rats at doses of 500, 1000, and 2000 mg/kg resulted in deaths at doses of 1000 and 2000 mg/kg. The maximum tolerated dose (MTD) was 500 mg/kg. The Toxicity of the gastrointestinal, hepatic, and immune systems may have contributed to the deaths. Administering HA121-28 orally to Beagle dogs at doses of 500 and 1000 mg/kg, which came out with an MTD of 1000 mg/kg.

Repeat-dose Toxicity

The results of a 28-day repeated dose toxicity study showed that the main target organs for toxicity in SD rats orally administered HA121-28 for four consecutive weeks were the liver, kidneys, immune system, intestines, female reproductive system, bones, teeth, and skin. The highest no-observed-adverse-effect level (NOAEL) was 15 mg/kg. In Beagle dogs orally administered HA121-28 for four consecutive weeks, the main target organs for toxicity were the immune system, gastrointestinal tract, and adrenal glands. The highest NOAEL was 12 mg/kg.

Genotoxicity

The study of genetic toxicity results indicated that HA121-28 did not exhibit significant genetic toxicity, as evidenced by negative results in the *in vitro* Ames test, CHL test, and *in vivo* mouse micronucleus test.

2.2.4. Clinical Studies

As of August 22, 2022, a total of 5 clinical studies were conducted. For specific details on each study, please refer to the investigator's brochure.

2.3. Risk/Benefit Assessment

2.3.1. Known Potential Risks

Based on phase 1 clinical study of HA121-28 and the safety data available in this study, the main adverse reactions include skin manifestations such as itching, rash, and eczema; gastrointestinal reactions such as nausea, vomiting, diarrhea, and decreased appetite; cardiovascular system reactions such as prolonged QTc interval and hypertension; liver and kidney function reactions such as elevated alanine aminotransferase and serum creatinine. Among them, the most common adverse reactions are diarrhea, rash, and prolonged QTc interval, and the overall safety is good.

2.3.2. Known Potential Benefits

The results of preclinical studies indicate that HA121-28 tablets are a multi-target kinase inhibitor primarily targeting the RET pathway. In phase 1 exploratory clinical study, efficacy was observed in advanced RET fusion NSCLC. As of April 28, 2021, among the advanced RET fusion NSCLC who had undergone at least one efficacy evaluation, 7 of 17 patients showed partial response. HA121-28 tablets may be a strategic treatment option for patients with advanced NSCLC.

2.3.3. Assessment of Potential Risks and Benefits

The preclinical pharmacological mechanism of HA121-28 tablets is clear, and preclinical pharmacological studies have shown that it has significant anti-tumor effects. Corresponding anti-tumor efficacy has also been observed in early clinical studies. Safety results indicate that HA121-28 has good safety and compared with other tyrosine kinase inhibitors, there have no new toxic target organs or adverse reactions been reported.

In terms of risk control, patients with a higher expected safety risk, including but not limited to patients with a history of severe cardiovascular disease or other serious systemic diseases, are excluded from selection, and those with sufficient organ function are included.

Based on the mechanism of action of HA121-28, preclinical studies, and clinical study results obtained, it can be concluded that HA121-28 has good safety and shows promising anti-tumor efficacy in patients with RET fusion NSCLC. Therefore, it is worth further development.

3. Study Objectives and Endpoints

Primary objective:

To evaluate the efficacy of HA121-28 tablets in patients with RET fusion-positive advanced NSCLC.

Secondary objectives:

- To evaluate the safety of HA121-28 tablets in patients with RET fusion-positive advanced NSCLC.
- To evaluate the PK characteristics of HA121-28 tablets in patients with RET fusion-positive advanced NSCLC.

Primary endpoint:

The ORR was evaluated by an IRC according to RECIST 1.1 criteria.

Secondary endpoints:

- ORR evaluated by the investigator according to RECIST 1.1 criteria.
- The disease control rate (DCR), progression-free survival (PFS), and duration of response (DOR) were evaluated by both the investigator and an independent review committee according to the RECIST 1.1 criteria.
- Overall survival (OS);
- Treatment-emergent adverse events (TEAEs) and serious adverse events (SAEs) during the treatment period.
- Plasma concentrations of HA121-28.

4. Study Design

4.1. Overall Design

This is a phase 2, single-arm, open-label, multicenter clinical study.

The study plans to enroll 83 patients with advanced NSCLC who have experienced disease progression after at least the first-line standard of care and evaluate the efficacy and safety of HA121-28 tablets in patients with unresectable locally advanced or metastatic RET fusion-positive NSCLC.

After obtaining informed consent from the patients or their guardians, the screening will begin

within 28 days before the patient administering the study treatment.

Eligible patients in screening will enter the treatment period: patients who meet the inclusion criteria will receive HA121-28 tablets at a recommended starting dose of 450 mg once daily for 21 consecutive days, followed by a 7-day drug-free period, constituting a 28-day treatment cycle. Treatment will continue until disease progression, intolerable toxicity, death, or any other reason for discontinuation of treatment. During the treatment period, investigators may adjust the dose based on the patient's tolerance, as detailed in section 6.1.3. Safety assessments will be conducted every cycle on day 28 (±3 days). Tumor imaging assessments will be performed every 8 weeks (±7 days) from C1D1 based on RECIST 1.1 until week 40, and then every 12 weeks (±7 days) thereafter until disease progression, initiation of new anti-tumor therapy, death, or patient withdrawal from the study (whichever occurs first).

Patients who discontinue treatment and/or withdraw from the study should have a treatment completion/withdrawal visit within 14 days of their last dose. A safety follow-up visit should be conducted 28 days after the last dose. After the final tumor imaging evaluation, researchers should contact patients every 12 weeks (±7 days) by phone or in person to determine their survival status and information on subsequent anti-tumor treatment.

4.2. Rationale for Dose Selection

According to the results of preclinical studies, the anti-tumor efficacy of the drug increases with increasing doses, and the maximum target inhibition can be achieved at the maximum tolerated dose in clinical studies, and achieving the expected clinical anti-tumor efficacy. In the dose escalation of the phase 1 study, a preliminary exploration of the safety and tolerability of doses ranging from 25 mg to 800 mg has been completed. Among the three patients in the 800 mg dose group, two experienced DLT events, and the maximum tolerated dose (MTD) has been determined to be 600 mg. It has been preliminarily demonstrated that the selected dose of 600 mg was tolerable in solid tumor, and anti-tumor effects have been observed in patients at doses of 450 mg and 600 mg.

Based on the current safety and efficacy data, and considering the balance between benefits and risks for the patients, the recommended starting dose of 450 mg was selected for safety and efficacy exploration in the target tumor type in this Phase 2 study, following discussions with

the investigator.

4.3. End of Study Definition

Study completion refers to all patients completing the final visit or completing all the steps listed in the schedule of assessment (SoA), or other reasons that lead to the termination of the study.

5. Study Population

5.1. Inclusion Criteria

(1) Voluntarily participate in this study and sign the informed consent form;

(2) Aged 18 ~ 75 years old (inclusive), male or female;

(3) Patients with histologically or cytologically confirmed unresectable locally advanced or metastatic NSCLC;

(4) RET gene fusion, as demonstrated by the "Next-generation" sequencing(NGS) method in the central laboratory with College of American Pathologists(CAP) or Clinical Laboratory Improvement Amendments(CLIA) certification;

(5) Progressive disease after at least one line of the standard of care (including patients with disease progression during or within 6 months of the end of adjuvant therapy);

(6) At least one measurable lesion according to RECIST 1.1 (for lesions previously treated with radiation, the lesion can be included as a measurable lesion only if there is clear disease progression after radiotherapy);

(7) Eastern Cooperative Oncology Group (ECOG) performance status (PS) score 0-1;

(8) Adequate organ function, laboratory tests meeting the following criteria:

- Neutrophil count (ANC) ≥ 1.5 × 10^9/L (no G-CSF for WBC-elevating therapy within 2 weeks prior to the laboratory test);
- Platelet count (PLT) ≥ 75 × 10^9/L (no platelet transfusion or other drugs to promote platelet production within 2 weeks before the laboratory test);
- Hemoglobin (Hb) ≥ 90 g/L(not receiving red blood cell transfusion or erythropoiesis-stimulating drugs within 2 weeks before the laboratory test);
- Alanine aminotransferase (ALT) and aspartate aminotransferase (AST) ≤ 3 ×

upper limit of normal (ULN) (≤ 5.0 × ULN for patients with liver metastases);

- Serum total bilirubin (TBIL) ≤ 1.5 × ULN;
- Serum creatinine ≤ 1.5 × ULN;
- Albumin ≥ 30 g/L;

(9) Male and female patients of childbearing age must agree to take effective contraceptive measures during treatment and for 6 months after.

5.2. Exclusion Criteria

(1) Have a documented oncogenic driver gene alteration other than RET gene in NSCLC, such as EGFR, BRAF, or KRAS gene activated mutations, MET exon 14 skipping mutations or high-level amplification, and ALK, ROS1, or NTRK1/2/3 gene fusions;

(2) Prior treatment with selective RET inhibitors, including investigational selective RET inhibitors, such as LOXO-292, BLU-667, RXDX-105, vandetanib, cabozantinib, etc.;

(3) Patients who have previously received any anti-tumor therapy (including but not limited to chemotherapy, radiotherapy and targeted therapy, etc.) within 4 weeks before the first use of the study drug; traditional Chinese medicine or Chinese patent medicine with anti-tumor indications within 2 weeks, or local palliative radiotherapy for the relief of bone metastasis pain within 2 weeks;

(4) Patients with abnormal coagulation function (INR > 1.5 or APTT > 1.5 × ULN), bleeding tendency (such as active peptic ulcer), or those receiving thrombolytic or anticoagulant therapy;

(5) Urine routine showed urine protein ≥ + + and 24 h urine protein > 1.0 g, suggesting significant proteinuria. It may be indicative of renal dysfunction and requires further investigation as assessed by the investigator;

(6) Patients who have undergone major surgery within 4 weeks before the first dose or are expected to undergo major surgery during the study;

(7) Patients with central nervous system (CNS) metastases or imaging evidence indicating that the patients currently have progressive neurological symptoms. If the patient requires treatment with corticosteroids for their CNS disease, the dose must be stable within two weeks prior to the first dose;

(8) Presence of poorly controlled pericardial, pleural, or peritoneal effusion;

(9) Interstitial pneumonia requiring steroid therapy, drug-induced pneumonitis, radiation pneumonitis (except for stable radiation pneumonitis);

(10) Significant cardiovascular disease, such as heart failure accessed as New York Heart Association (NYHA) Class 2 or higher, unstable angina, serious arrhythmia, myocardial infarction or stroke within 6 months prior to the first dose, poorly controlled hypertension (defined as systolic blood pressure > 150 mmHg or diastolic blood pressure > 100 mmHg on multiple measurements while on medication);

(11) Patients who meet any of the following criteria will be excluded:

- QT interval (QTcF) value ≥ 470 ms for females and ≥ 450 ms for males; or congenital long QT syndrome, taking drugs known to prolong QT interval, family history of long QT syndrome;
- Resting ECG shows any clinically significant abnormalities in rhythm, conduction, or morphology that require clinical intervention;
- Cardiac ejection fraction less than 50%;

(12) Patients with active hepatitis B virus or hepatitis C virus infection:

- HBsAg positive with HBV DNA higher than the upper limit of the normal range of the study site;
- HCV antibody positive with HCV RNA higher than the upper limit of the normal range of the site;

(13) Human immunodeficiency virus-infected (HIV positive);

(14) Inability or severe dysphagia;

(15) Patients who have suffered from or are complicated with any other malignant tumor within 5 years (except radically resected skin basal cell carcinoma, skin squamous cell carcinoma, superficial bladder cancer, local prostate cancer, in situ cervical cancer or other carcinoma in situ);

(16) Presence of any severe and/or uncontrolled disease that may affect the drug evaluation in the judgment of the investigator, including but not limited to life-threatening autoimmune system diseases; drug abuse; severe nervous system diseases (such as

epilepsy, dementia, etc.); history of severe mental disorders; severe infection, hypothyroidism, etc.;

(17) Pregnant or lactating women;

(18) Other conditions that, in the opinion of the investigator, make participation in the study unsuitable.

5.3. Screening Failure

Patients who have signed the informed consent form but have not started treatment for any reason will be considered screening failures. Patients who have failed screening for valid reasons will be allowed to undergo screening again.

For patients who fail screening, the following information must be collected and the original records must be saved and recorded in the eCRF:

- Reason for Screen Failure
- Informed Consent Form (Record the signing time in the eCRF and save the Informed Consent Form)
- Confirm Inclusion/Exclusion Criteria (Missing allowed)
- Demographic characteristics
- Adverse events (AEs, only if serious AEs occur)
- The previous history of anti-tumor therapy

6. Study Intervention

6.1. Study Treatment

6.1.1. Study Treatment Description

Study treatment: HA121-28 Tablets

Dosage Form: Tablet

Specification: 25mg, 100mg, 150mg, 200mg.

Shelf life: Tentative 24 months for 25mg, 100mg, and 200mg; Tentative 36 months for 150mg.

Molecular formula: $C21H24BrFN4O2 \cdot 2HCl$

Molecular weight: 536.27 (dihydrochloride salt); 463.34 (free base)

6.1.2. Dosing Regimen of Study Treatment

The recommended starting dose is 450 mg. It should be administered once daily for 21 consecutive days, followed by a 7-day drug-free period. Each cycle lasts 28 days and the medication should be taken orally with warm water.

During the treatment period, the investigator may adjust the dosage based on the patient's tolerance.

The time and dose of administration will be recorded in detail in the diary card.

6.1.3. Dose Modification Principles

The dose adjustment principles for related AEs below are recommended. Investigators can also evaluate the risk benefits and current conditions of the subjects based on their own clinical experience and updated research data during the study, and determine whether dose adjustment and management should be performed. For AEs that occur during the study, investigators should provide the best supportive treatment based on their clinical experience. For detailed information on the adjusted dose levels and principles, please refer to Table 6-1 and Table 6-2. The study treatment is allowed to be reduced to 300 mg. If further reduction is necessary, it must be comprehensively evaluated by the investigator and sponsor and approved by the sponsor.

Table 6-1 Dose Modification Levels of Study Treatment

Starting dose	450 mg
Dose reduction	300 mg

Table 6-2 Recommended Dose Modifications for Toxicity

NCI-CTCAE V 5.0 (Supportive Care)	Dose Modification
**Liver**	
AST/ALT (Grade 2)	Continued treatment is recommended with weekly monitoring of liver function until it recovers to grade ≤1 or baseline level.
ALT/AST (Grade 3)	First occurrence: Discontinue medication and recommend monitoring liver function weekly until it recovers to grade ≤1 or baseline level. Then, the investigator should consider resuming the medication at the original dose or reducing it by one level based on the patient's tolerance, and liver function should be monitored weekly for at least 4 weeks. If it occurs again, the investigator will determine whether to reduce the dosage or discontinue the treatment.
ALT/AST (Grade 4)	Treatment discontinuation
ALT/AST>3×ULN (≥ Grade 2) and total bilirubin>2×ULN.	Treatment discontinuation
**Skin adverse reactions (rash, hand-foot syndrome, etc.)**	
Grade 2	Supportive treatment should be continued until the symptoms recover to ≤ grade 1 or baseline level. If the symptoms cannot be relieved after supportive treatment, the medication should be suspended until the symptoms recover to ≤ grade 1 or baseline level. Then, the investigator should consider administering the original dose or reducing the dose level based on the patient's tolerance.
Grade 3	First occurrence: Discontinue the medication until the AE recovers to ≤ Grade 1 or baseline level, and then the investigator will consider resuming the medication at the original dose or reducing the dose level based on the patient's tolerance.<br>Reappearance: The investigator will determine whether to reduce the dosage or discontinue the treatment.
**QTc prolongation**	
QTc>500 msec or an increase in QTc≥60 msec.	After the initial electrocardiogram (ECG) collection, if the average QTc is >500 msec during the same day's retest, the patient must suspend treatment until recovery to ≤1 grade or baseline and reduce the dosage level. If it occurs again, the patient should discontinue treatment. If the increase is ≥60 msec compared to the baseline, the investigator will consider whether to suspend medication or adjust the dosage based on the patient's specific situation.
**Diarrhoea**	

Grade 2	Supportive treatment should be continued until the symptoms recover to ≤ grade 1 or baseline level. If the symptoms cannot be relieved after supportive treatment, the medication should be suspended until the symptoms recover to ≤ grade 1 or baseline level. Then, the investigator should consider administering the original dose or reducing the dose level based on the patient's tolerance.
Grade 3	First occurrence: Discontinue the medication until the AE recovers to ≤ Grade 1 or baseline level, and then the investigator will consider resuming the medication at the original dose or reducing it by one dose level based on the patient's tolerance.<br>Reappearance: The investigator will determine whether to reduce the dosage or discontinue the treatment.
**Hematological toxicity and other treatment-related non-hematological AEs in clinical studies.**	
Grade 3	First occurrence: Discontinue the medication until the AE recovers to ≤ Grade 1 or baseline level, and then the investigator should consider resuming the medication at the original dose or a reduced dose level based on the patient's tolerance.<br>If it occurs again, the investigator will determine whether to reduce the dosage or discontinue the treatment.
Grade 4	Treatment discontinuation
Note: The results of liver function monitoring in another hospital are accepted;	

6.2. Preparation/Handling/Storage/Responsibilities Regarding Study Treatment

6.2.1. Drug Receipt and Accountability

Upon receiving the study drug by mail, the investigator or designated personnel will verify the quantity and condition of the drugs, examine the packaging labels and expiration dates, and document the date and quantity received in the drug receipt and distribution log.

The drug management record for the clinical study will include the date of receipt, quantity, batch and lot number, study ID, study period, patient ID (for distribution of study drug to patients), distribution date and quantity, dispenser, date and quantity of drug returned, and the investigator responsible for drug recovery.

After all patients complete treatment, the study drug (including packaging) will either be returned to the sponsor or destroyed according to their instructions (the sponsor may increase the number of times for retrieval/destruction during the study period). Only study drugs that

have been counted and verified in quantity can be returned. The return of study drugs requires written documentation, which should be archived together with transportation documents. If any unused study drugs are damaged, the investigator will provide a written explanation for each damaged drug and submit it to the sponsor. The damaged study drugs and the number of damaged drugs must be documented in writing, and the damaged drugs must be returned to the sponsor.

6.2.2. Dosage Form, Appearance, Packaging and Labeling

The label of study treatment must contain the following:

(1) Name of the clinical study treatment;
(2) Indications;
(3) Protocol number;
(4) Drug number;
(5) Strength;
(6) Manufacturing batch number;
(7) Mark the instructions "for clinical study use only", "keep out of reach of children", and "return any unused medication and all packaging to the investigator".
(8) Dosage form;
(9) The period of use is indicated as XXXX (year)/XX (month)/XX (day).
(10) Description of usage;
(11) Storage conditions;
(12) The study treatment is provided by the sponsor, CSPC ZhongQi Pharmaceutical Technology (Shijiazhuang) Co., Ltd..

All of the above labels should be included in the general packaging box. If the size of the inner packaging is too small to include all of the above information, at least items (1) to (6) should be labeled.

6.2.3. Drug Product Storage and Stability

This product should be stored at room temperature. The shelf life of the 25 mg, 100 mg, and 200 mg doses is currently set at 24 months, while the shelf life for the 150 mg dose is

temporarily set at 36 months.

6.3. Methods to Mitigate Bias: Randomization and Blinding

Not applicable.

6.4. Compliance with Study Intervention

At each visit of the patients to the clinic, the investigator and/or authorized personnel from the study site will assess the patient's compliance by counting the pills (number of pills dispensed and returned by the patient), checking the patient's diary card, and reviewing the information provided by the patients and/or their caregiver. The usage record of the study treatment, the dosage taken, and the follow-up interval and completion of the study should be recorded and saved in the drug tracking table or equivalent document.

6.5. Concomitant Medications and Treatments

Collect all medication treatment or combined treatment information of the patients from 28 days before the first dose to the safety visit period. Medication treatment should record the generic name of the drug, single dose, frequency of administration, the reason for treatment, the start and end date; Non-medication treatment should record the name of the treatment, and the start and end dates.

6.5.1. Prohibited and/or Restricted Medications and Treatments

During the study, any anti-tumor therapy other than the study treatment is prohibited, including cytotoxic chemotherapy, targeted therapy, biological therapy, radiation therapy, endocrine therapy (with anti-tumor activity), Chinese herbal medicine or traditional Chinese medicine with anti-tumor indications, and other drugs that can affect efficacy evaluation.

6.5.2. Permitted and/or Cautionary Medications and Treatments

During the study, patients are allowed to receive supportive therapy for AEs and other concomitant illnesses based on clinical requirements. Before vaccination, communication and consultation with the investigator and sponsor are required.

(1) During the clinical study, drugs that prolong the QT interval for systemic treatment should be used with caution. If necessary, a 12-lead electrocardiogram monitoring

should be performed based on clinical conditions. Mainly including but not limited to the following categories of drugs:

- Antibiotics (clarithromycin, azithromycin, erythromycin, roxithromycin, metronidazole, moxifloxacin);
- Antiarrhythmic drugs (quinidine, sotalol, amiodarone, procainamide, procainamide).
- Antifungals (fluconazole, ketoconazole);
- Antimalarials (mefloquine, chloroquine);
- Antidepressant medications (amitriptyline, imipramine, clomipramine, lofepramine, doxepin).

(2) If systemic therapy or local analgesia cannot effectively control the bone metastases, palliative small-area radiotherapy is allowed.

7. Discontinuation and Withdrawal Criteria

7.1. Discontinuation Criteria

Patients must stop receiving the study treatment if any of the following conditions occur during the study:

(1) The patient experiences intolerable toxicity;
(2) The investigators believe that continuing to receive the study treatment carries more risks than benefits.
(3) Concomitant diseases that occur during the treatment are not allowed to continue medication in the follow-up.
(4) Radiologically assessed disease progression;
(5) Start a new anti-tumor therapy;
(6) The patient is unwilling to continue the treatment;
(7) Pregnancy of the patient;
(8) Either of the withdrawal criteria is met.

Patients who withdrawal from treatment should continue to follow-up visits according to the study protocol unless the reason for discontinuation is any of the criteria for study withdrawal

listed in section 7.2.

The termination of intervention in a study does not mean the termination of the study. The follow-up procedures specified in the study protocol should be completed accordingly. Investigators should collect safety and efficacy data from patients who have received or completed the intervention (if possible). In addition, there should be a dedicated page in the eCRF to record the date and reason for treatment termination.

7.2. Withdrawal Criteria

The patients have the right to withdraw from the study at any time for any reason, and their interests will not be harmed as a result. Patients will be withdrawn from the study if any of the following situations occur:

(1) Participant withdraws voluntarily;

(2) Death;

(3) Lost to follow-up;

(4) Study discontinuation.

7.3. Lost to Follow-up

If a patient fails to return to the study site for two consecutive planned visits and the study staff is unable to contact them, they will be considered lost to follow-up.

If a patient fails to return to the study site for the scheduled study visit, the following actions must be taken:

- The study site attempts to contact the patients to reschedule missed visits, explain the importance of adhering to the visit schedule, and confirm whether the patients are willing and/or should continue to participate in the study.
- Before a patient is considered lost to follow-up, the investigator or designated personnel will make every effort to re-establish contact with the patient (if possible, attempting to contact the patient or their family members three or more times during different time periods, such as the phone number, landline number, WeChat account, or other instant contact information provided by the patient or their family members. If necessary, reminders can be sent via WeChat or SMS, or a registered letter can be

sent to the patient's latest mailing address or other valid contact information). These attempts to contact the patient should be documented in the patient's medical records or study files.

- If the patient still cannot be contacted, it will be considered as withdrawal from the study due to loss of follow-up.

8. Study Assessments and Procedures

8.1. Tumor Imaging Assessments

Tumor response evaluation will be conducted using RECIST 1.1 criteria and assessed separately by an independent review committee (IRC) and investigators.

Baseline tumor imaging evaluation will be performed within 28 days prior to the first administration of the study treatment. Chest, abdomen, and pelvic contrast-enhanced CT scans should be performed (if the patient is allergic to contrast agents or has contraindications for contrast-enhanced CT, CT plain scan or magnetic resonance imaging (MRI) may be considered), brain contrast-enhanced MRI should be performed (if the patient has contraindications, head CT may be considered), and whole-body bone scans should be performed (if the patient has not developed new bone metastasis symptoms, the bone scan results within 6 months prior to signing the ICF can be accepted; lesions found by bone scan need to be confirmed by CT or MRI). If there are clinical indications, appropriate methods can be used to examine any other known or suspected disease sites.

If the patient has previously undergone relevant imaging evaluation at this hospital and has been confirmed by the investigator and the IRC to meet the study requirements, the imaging data before the patient signs the informed consent form can be used as the baseline without the need for re-examination.

Target Lesion Record: Number, location, description, maximum diameter measurement of each lesion, and minimum diameter measurement of lymph nodes, including the total sum of diameters of all target lesions.

The imaging methods used for follow-up tumor assessment should be consistent with those used during the screening period. If no brain metastases are detected during the screening period

using brain-enhanced MRI (or head CT), the investigator will evaluate whether it is necessary to perform it in the follow-up period. The need for a whole-body bone scan will also be evaluated by the investigator for the follow-up assessment.

Tumor imaging evaluation should be conducted every 8 weeks (±7 days) after the first administration until week 40, then every 12 weeks (±7 days) thereafter until disease progression, initiation of new anti-tumor therapy, death, or patient withdrawal from the study (whichever occurs first). Patients with a first efficacy evaluation of PR or CR must have efficacy confirmed at least 4 weeks later.

8.2. Safety and other assessments

Vital signs

Vital signs include body temperature, respiration, pulse, and blood pressure. It is recommended to rest quietly for at least 3 minutes before measurement and to measure while sitting with the elbow at the same level as the heart. This should be done during the screening period, at the CnD28, EOT visits, and at the visit 28 days after the last medication.

Physical examination

Physical examination includes skin and mucous membranes, lymph nodes, head and neck, chest, abdomen, spine, musculoskeletal system, nervous system, and other areas. It is conducted during the screening period, CnD28, end of treatment visit, and 28 days after the last dose of medication.

12-lead ECG

The investigator should record the results of heart rate, QT, QTcF, QRS, and P-R interval, as well as clinically significant electrocardiogram abnormalities. QTcF is calculated using the following formula: QTcF=$QT/(60/HR)^{0.33}$. Signed original electrocardiograms/copies will be archived at the study site. This will be done during the screening period, C1D15, CnD28, EOT visit, and 28-day follow-up after the last dose.

Echocardiography

The investigator should record the left ventricular ejection fraction and abnormal results of echocardiography. This should be done during the screening period and end-of-treatment visit. If the patient has previously undergone an echocardiogram at the hospital and the examination

time meets the protocol requirements, the patient does not need to undergo the examination again. The results before signing the informed consent form should be used to determine the inclusion/exclusion criteria.

Laboratory tests

Serology will be performed during the screening period, coagulation function and pregnancy tests will be performed during the screening period and at the end of treatment visit (EOT). Hematology, urinalysis, and blood biochemistry will be performed during the screening period, on Day 28 of Cycle 1, at EOT, and 28 days after the last dose of the study drug. If a patient has previously undergone relevant laboratory tests at the study site and the test results meet the protocol requirements, the patient does not need to undergo the tests again and the results prior to signing the informed consent form will be used to determine eligibility. Please refer to Table 8-1 for specific parameters of each test.

Table 8-1 Laboratory Parameters

**Hematology**	Gamma-glutamyl transferase (GGT)
White blood cell count	Creatinine
Red blood cell count	Urea/Urea Nitrogen
Hemoglobin	Uric acid
Platelet count	Blood glucose (fasting)
Absolute Lymphocytes	Triglycerides
Absolute neutrophil count	Total cholesterol
Monocyte count	Potassium
Eosinophil count	Sodium
Basophil count	Chlorine
**Urinalysis**	Calcium
Leukocytes	**Coagulation**
Urine occult blood	Plasma prothrombin time (PT)
Urobilinogen	Activated partial prothrombin time (APTT)
Ketones	International normalized ratio (INR)
Glucose (urine glucose)	**Pregnancy test**
Erythrocyte quantitation	Serum/Urine β-HCG
Leukocyte quantitation	**Serum virology**
Urine protein	Hepatitis B surface antigen (HBsAg)
**24-hour urine protein quantification** (to be tested when screening urine protein is ≥++, and testing during treatment and follow-up period can be considered based on actual situation).	Hepatitis B surface antibody

**Blood chemistry**	Hepatitis B e antigen
Total bilirubin (TBIL)	Hepatitis B e antibody
Direct bilirubin	Hepatitis B core antibody
Alanine aminotransferase (ALT)	Hepatitis C antibody (Anti-HCV)
Aspartate aminotransferase (AST)	Human immunodeficiency virus antibodies (Anti-HIV)
Albumin (ALB)	Hepatitis B virus DNA quantitation (HBV-DNA) *
Alkaline phosphatase (ALP)	Hepatitis C virus RNA quantitation (HCV-RNA) *
Lactate dehydrogenase (LDH)	
* HBV-DNA quantification should be tested when HBsAg is positive, and HCV-RNA quantification should be tested when HCV antibody is positive.	

8.3. Adverse Events and Serious Adverse Events

8.3.1. Definition

8.3.1.1. Adverse Event (AE)

All adverse medical events that occur in patients after receiving the study drug may manifest as symptoms, signs, diseases, or laboratory abnormalities, but may not necessarily be causally related to the study drug.

8.3.1.2. Adverse Drug Reaction (ADR)

Any harmful or unexpected reaction related to the study drug that may occur during a clinical study. There is at least a reasonable possibility of a causal relationship between the study drug and the AE, which cannot be ruled out.

8.3.1.3. Treatment-Emergent Adverse Events (TEAEs)

Events that occur during treatment that are not present before treatment or have worsened compared to before treatment.

8.3.1.4. Serious Adverse Event (SAE)

An SAE refers to one of the following conditions that occur in a patient after receiving the study drug in a clinical study:

(1) Leading to death;

(2) Life-threatening;

The term "life-threatening" in the definition refers to the risk of death for the patient at the time of the event, rather than the possibility of death if the event worsens.

(3) Requires hospitalization or prolongation of hospitalization;

However, the following admissions are not considered SAEs:

- Less than 24 hours in hospital.
- Pre-planned hospitalization (e.g., elective or scheduled surgery arranged prior to the start of the study; hospitalization is part of the study procedure).
- Hospitalization is not related to AEs (such as social hospitalization for short-term care purposes).

(4) Permanent or severe disability or loss of function;

Loss of function refers to a severe impairment of the normal life ability of an individual.

(5) Congenital anomaly or birth defect.

(6) Other medically significant events.

Important medical events may not immediately endanger life, cause death, or require hospitalization, but are generally considered serious if medical intervention is needed to prevent any of the aforementioned outcomes. For example, important treatments in the emergency room, allergic bronchospasm occurring at home, non-hospitalized malnutrition or seizures, drug dependency, or addiction.

8.3.1.5. Suspected Unexpected Serious Adverse Reaction (SUSAR)

Suspected and unexpected serious adverse reactions with the nature and severity of the clinical presentation out of the range of the information available in the investigator's brochure, package insert of marketed drugs, or product characteristics summary.

8.3.1.6. Adverse Events of Special Interest (AESI)

Adverse events of special interest (serious or non-serious) are a type of event that is of scientific and medical concern for the sponsor's drug or study project. Such events usually require further investigation to describe their characteristics and gain understanding. Given the nature of these events, continuous monitoring is required, and a rapid communication mechanism should be established between the investigator and the sponsor.

8.3.2. Adverse Event Assessment Category

According to the following criteria, the investigator should assess and record all of AEs.

8.3.2.1. Seriousness

For each AE, severity must be determined according to the definition of SAE.

8.3.2.2. Severity

The severity of AEs will be recorded using the Common Terminology Criteria for Adverse Events, which is a standard terminology developed by the National Cancer Institute(NCI-CTCAE, version 5.0). If an AE falls outside the scope of this standard, the investigator will be according to Table 8-2 for assessment.

Table 8-2 Severity Criteria

Grading	Severity Description
Grade 1:	Mild; asymptomatic or mild; only observed clinically or diagnostically; no treatment required.
Grade 2:	Moderate; requires minor, localized, or non-invasive treatment; limited instrumental activities of daily living (IADLs) commensurate with age (IADLs refer to activities such as cooking, shopping for clothes, using the telephone, managing finances, etc.).
Grade 3:	Severe or clinically significant but not immediately life-threatening; resulting in hospitalization or prolonged hospital stay; causing disability; limiting self-care activities of daily living (self-care activities of daily living refer to bathing, dressing, eating, grooming, taking medication, etc., and do not include bedridden).
Grade 4:	Life-threatening; urgent treatment indicated.
Grade 5:	Deaths related to AEs.

8.3.3. Causality

8.3.3.1. Causality between Adverse Events and Study Drug

For all AEs, investigators should assess the causal relationship between each event and each study drug separately. The assessment of causality should be performed by an authorized clinical physician, who should not only determine whether there is a causal relationship with the study drug, but also provide as much detail as possible regarding the basis for the determination.

Approach to assessment: Important factors to consider when evaluating the causal relationship between AEs and study drug include:

a. Temporal relationship to dosing

The event should have occurred after the drug administration. The time interval between drug exposure and the occurrence of the event should be evaluated in the clinical context of the event.

b. Reactions after discontinuation (dechallenge) and re-administration (rechallenge) of the medication.

The response of the patients after dechallenge or rechallenge should be evaluated based on the common clinical course of relevant events.

c. Underlying disease, concomitant disease, concurrent disease.

Each event should be evaluated in the context of the current treatment of the disease and the patient's medical history and course of any other underlying conditions.

d. Concomitant Medications or Treatments

The investigator should examine the other drugs or treatments that the patient is receiving when AEs occur, to determine if they may have contributed to the event.

e. Known types of reactions (clinical/preclinical) for such drugs

f. Exposure to physical/psychological stress

Exposure to stress may induce adverse changes in patients and provide a more reasonable explanation for this event.

g. Pharmacology and PK of study treatment

The PK properties of the therapeutic agent (absorption, distribution, metabolism, and excretion), as well as the pharmacodynamics of individual patients, should be taken into account when conducting the investigation.

The relationship between the study drug and AEs is classified as follows: related, probably related, possibly related, possibly unrelated, not related. The specific criteria for determining the relationship are shown in Table 8-3.

Table 8-3 Determination of the Relationship between Adverse Events and Study Drug

	Related	Probably related	Possibly related	Possibly unrelated	Not related

a. Is there a reasonable temporal relationship between the event and the study drug?	+	+	+	+	−
b. Does the event match the known adverse reactions of the drug?	+	+	±	−	−
c. Does the event resolve or disappear after discontinuation or dose reduction?	+	+	±?	±?	−
d. Does the event reappear after re-dosing?	+	?	?	?	−
e. Can the effect of combination therapy, the progression of patients' condition, and the impact of other treatments be used to explain the event?	−	−	±	±	+

Note: + indicates positive, - indicates negative, ± indicates uncertain, ? indicates unclear.

When there is incomplete correspondence with a certain evaluation result or uncertainty about whether a clinical observation is an AE, strict evaluation and reporting should be conducted.

8.3.3.2. Causality to Study Procedures and Operation

Based on the question of whether there is a "reasonable causal relationship" between AEs and the study protocol, the evaluation results should be recorded as "related" or "not related" in the CRF. Generally refers to AEs caused by harm or injury to any clinical procedures (such as tissue biopsy), AEs caused by drug discontinuation, dose reduction or adjustment of treatment regimen required by the study protocol, or AEs caused by other prophylactic medications administered prior to the study drug.

8.3.4. Action Taken with Study Treatment

Record all measures taken to address AEs related to the study drug according to the following categories. Specific measures should be detailed in the CRF.

- Permanently discontinue
- Drug interruption

- Dose reduction
- Dose unchanged
- N/A
- Unknown

8.3.5. Other Specific Treatment for Adverse Events

- No treatment was received
- Drug therapy
- Other treatments

8.3.6. Outcome

The outcome of the AE will be recorded as follows:

- Recovered/resolved
- Recovering/resolving
- Recovered/resolved with sequelae
- Not recovered/not resolved/ongoing
- Fatal
- Unknown

8.3.7. Collection, Recording and Evaluation of Adverse Events

The investigator is responsible for collecting all adverse medical events that occur from the signing of the informed consent form to the end of the safety follow-up period (28 days ± 3 days after the last dose).

After the end of the safety follow-up period, AE will no longer be actively collected. If an SAE occurs and the investigator believes there is a reasonable causal relationship between the SAE and the study drug, the SAE should be reported to the sponsor according to the SAE reporting process.

AEs that have not fully recovered or stabilized by the end of the safety follow-up period (regardless of causality), follow-up must be conducted until recovery (to baseline or complete recovery), clinical stability is achieved, or a reasonable explanation is obtained.

AEs occurring between the informed consent form and first use of the drug may be recorded in

the CRF as medical history/accompanying diseases unless they meet one of the following criteria, in which they should be recorded as AEs: harm caused by any clinical laboratory examination procedures; AEs caused by discontinuation of the study protocol; AEs caused by drugs other than the study drug.

All AEs that occur during the safety follow-up period after the first administration of the drug must be recorded, including information such as the name, the start and end dates (and times) of the AE, severity, seriousness, the investigator's assessment of the relationship with the study drug, measures taken for the study drug in response to the AE, and outcome.

- If a patient dies, the cause or symptom leading to the death should be reported as the AE name. "Death" should be considered as an outcome of the AE. If the cause of death is unknown, "death of unknown cause" should be reported as the AE name.
- Events that clearly correspond to the progression of the tumor disease should not be recorded as AEs. However, if the investigator determines that the event is atypical, accelerated progression, or caused by the study drug, it should be recorded as an AE.
- Any death event caused by any reason during the safety follow-up period should be reported to the sponsor according to the SAE process.

The investigator is responsible for determining the causal relationship between recorded AEs and the study drug, as well as the study procedures and operations, as outlined in section 8.3.3 The sponsor must conduct a separate assessment of the expectedness, severity, and causal relationship between SAE and the study drug. The reference document for the expectedness assessment is the most current version of the Investigator's Brochure (IB).

8.3.8. Reporting of Serious Adverse Events

8.3.8.1. Investigator Responsibilities

Reporting Sponsor

For all SAEs and follow-up information that occur during the observation period described in section 8.3.7, the investigator must report to the sponsor within 24 hours of becoming aware. In the case of reports involving death, the investigator should provide the sponsor with additional information as needed, such as autopsy reports and final medical reports.

Reporting Ethics Committee

The investigator should promptly acknowledge receipt and review of relevant safety information provided by the sponsor regarding the clinical study, and consider whether the treatment of the patients has been adjusted accordingly. If necessary, The investigator should communicate with the patients as early as possible and report any suspected and unexpected serious adverse reactions provided by the sponsor to the ethics committee. In the case of a report involving a death event, the investigator should provide the ethics committee with other necessary information, such as autopsy reports and final medical reports.

8.3.8.2. Responsibilities of the Sponsor

The sponsor should report suspected and unexpected serious adverse reactions to the drug regulatory authority and the health department promptly. The reporting time limit is as follows:

- For fatal or life-threatening SUSARs, they should be reported within 7 days of first knowledge (day 0 being the day the sponsor first becomes aware) and followed up with additional information within the following 8 days.
- Report non-fatal or non-life-threatening SUSARs within 15 days.
- For follow-up reports, report within 15 days of obtaining new information.

The sponsor should promptly report any suspected and unexpected serious adverse reactions to all investigators and testing facilities, as well as the ethics committee involved in the clinical study.

8.3.8.3. Adverse Events of Special Interest

For AESIs, investigators should report to the sponsor following the SAE reporting process within 24 hours of becoming aware of the event.

AESIs in this study are:

- Prolongation of QT interval on electrocardiogram (graded as ≥3 on CTCAE scale).

8.3.9. Pregnancy

The investigator must report all pregnancy events that occur in female patients during the administration of the drug and throughout the safety follow-up period to the sponsor. Pregnancy outcomes should be carefully monitored and any abnormal outcomes in the mother or infant

should be reported.

If a male patient's partner becomes pregnant, information regarding the pregnancy process and outcome should be obtained with the partner's consent as soon as possible.

Once a pregnancy event occurs during the safety follow-up period after administration, the investigator should communicate with the patient in a scientific and rigorous manner based on the drug information, informing her/him of the potential impact and risks of the study treatment on pregnant women and fetuses. If a female patient experiences a pregnancy event, the investigator should immediately suspend the clinical study for that patient and discontinue the study treatment.

Within 24 hours of confirming a pregnancy event in a patient (or patient's partner), the investigator should complete a pregnancy report form and report to the sponsor (institutional review board, if required).

The investigator will complete a pregnancy report form and submit a follow-up report to the sponsor (and the institutional review board if required) within 24 hours of being informed of the pregnancy outcome.

8.4. Pharmacokinetic Assessment

Patients receive HA21-28 treatment after the implementation date of the revised protocol (V3.0) will undergo PK blood collection at any time point 4-8 hours after C1D1, before C1D15 (±1 day) (-30 min), 4-8 hours after dosing (if applicable), or on C1D28 (±3 days), C2D28 (±3 days), C4D28 (±3 days), C6D28 (±3 days) after dosing. For patients enrolled before the execution date mentioned above and have not terminated treatment, PK sampling can be performed at any point during any two visits of C1D15 (±1 day) before (-30 min) and 4-8 hours after dosing, and CnD28 (±3 days). If a patient terminates treatment during the study, a blood sample can be collected for PK analysis on the day of the EOT visit as appropriate. 3ml of blood should be collected each time, and plasma should be separated for HA121-28 concentration determination. Please refer to the relevant operation manual for the processing and storage of blood samples. The actual sampling time will be recorded in the original medical record and eCRF, and used for calculation of relevant parameters.

9. Statistical Considerations

The detailed summary and statistical analysis methods for the data collected in this study will be included in the Statistical Analysis Plan (SAP), which will be finalized after we complete the protocol and CRF but before we lock the database. The SAP will contain detailed information about all the statistical analyses that will be performed based on the main features of the protocol. If there are any changes to the protocol that are deemed to have a significant impact on the SAP by the sponsor or principal investigator, the SAP will need to be revised to maintain consistency with the study protocol.

9.1. Statistical Assumptions

This study is based on the evaluation of objective response rate (ORR) to assess efficacy. The hypothesis is that the efficacy of HA121-28 tablets is superior to the ORR of the historical control group.

H0： $\pi \leq \pi 0$；

H1： $\pi > \pi 0$；

PI 0: Historical control group for the ORR of second-line or above treatment in NSCLC with RET fusion.

A: HA121-28 tablets for the treatment of second-line or above NSCLC with RET fusion, with the ORR as the primary endpoint.

9.2. Sample Size Estimation

This study has a single-arm design, with the main efficacy measure being ORR. From a clinical perspective, an ORR of 30% or higher as the treatment of second-line or higher RET fusion NSCLC is considered clinically significant. Assuming that the ORR of HA121-28 tablets for the treatment as second-line or higher RET fusion NSCLC is 45%, with a one-sided significance level of 0.025 and a power of 80%, the sample size is estimated to be 83 using the single-group target value method. The study is indicated as successful, when at least 34 patients show improvement, corresponding to an ORR of 41%, the lower limit of the 95% confidence interval can be greater than 30%.

Table 9-1 Sample Size Estimation

Drug ORR Assumptions	Control criteria (Lower 95% confidence interval)	Sample Size	Minimum number of responses	Response Boundary	95% confidence interval (Clopper-Pearson)
45%	30%	83	34	41.0%	(30.28%,52.31%)

Sample size estimation will be performed using nQuery 8.

9.3. Analysis of Population

- Full Analysis Set (FAS): Includes all patients who have been enrolled and received at least one dose of the study treatment. FAS is used for demographic, baseline characteristics, and efficacy analysis.
- Per Protocol Set (PPS): Refers to a subset of patients in a clinical study who strictly adhere to the study protocol without any major protocol violations. PPS is used for sensitivity analysis and includes all patients from the full analysis set who meet the criteria for PPS.
- Safety Set (SS): Includes all patients who have received at least one dose of the study treatment.
- Pharmacokinetic Concentration Set (PKCS): Refers to a group of patients who have received at least one dose of the study treatment and have at least one assessable PK concentration without any significant deviation from the dosing regimen.

9.4. Statistical Analysis

9.4.1. General Methods

The statistical analysis of this study will be conducted using SAS 9.4 or higher version.

For the statistical description of categorical variables will be expressed in terms of the number of cases and percentage. For the statistical description of continuous variables includes the number of cases, mean, standard deviation, median, lower quartile, upper quartile, minimum value, and maximum value. Unless otherwise specified, a two-sided test will be used with a significance level of 0.05, and a 95% confidence interval will be used for parameter estimation.

9.4.2. Participant Disposition

- Summarize the information on patient enrollment, completion, withdrawal, protocol violations, and data set allocation.

- Plot a flow chart of patient disposition;
- Make lists to describe detailed information on the patients' enrollment, completion or withdrawal from the study, protocol violations, and data set division.

9.4.3. Demographic and Baseline Analyses

- The FAS set will be used for analysis;
- Statistically describe the baseline characteristics of the patients, including demographics, medical history, and other relevant factors.
- Calculate the sample size, mean, standard deviation, median, lower quartile, upper quartile, minimum value, and maximum value for continuous variables.
- The number and percentage of patients will be calculated with number and grade data.

9.4.4. Efficacy Analysis

- Primary endpoint measures
    - ➢ The analysis will be performed based on FAS and PPS;
    - ➢ Calculate the objective response rate (ORR) assessed by IRC assessment and calculate the 95% confidence interval using the Clopper-Pearson method.
- Secondary efficacy measures
    - ➢ Analysis will be performed based on FAS;
    - ➢ Determine the median time and 95% CI for PFS, DOR, and OS assessed by IRC and investigator using the Kaplan-Meier method. Plot corresponding survival curves;
    - ➢ Calculate the ORR assessed by investigators and the DCR assessed by both the IRC and investigator. Calculate the 95% confidence interval using the Clopper-Pearson method.

9.4.5. Pharmacokinetic Analysis

- Analysis based on PKCS;
- Collect PK samples at the designated time points according to the protocol, and measure the concentration of HA121-28 in plasma. Conduct a tabular and descriptive statistical analysis of the HA121-28 concentration;

- If there is sufficient data, the blood drug concentrations of the patients mentioned above will be analyzed using a nonlinear mixed-effects model (NONMEM) for population pharmacokinetic (PPK) analysis. The analysis report and details can be found in a separate report;
- The specific statistical analysis contents are detailed in the statistical analysis plan.

9.4.6. Safety Analysis

- Analysis based on SS;
- Summarize the number and percentage of Treatment-emergent Adverse event (TEAE)/treatment-related TEAE, SAEs/treatment-related SAEs, TEAE leading to discontinuation/treatment-related TEAE leading to discontinuation during the course of treatment;
- Summarize the number and percentage of TEAEs/treatment-related TEAEs, SAEs/treatment-related SAEs, TEAEs leading to discontinuation/treatment-related TEAEs leading to discontinuation, based on SOC/PT;
- Summarize the incidence and percentage of TEAEs/treatment-related TEAEs, SAEs/treatment-related SAEs, TEAEs leading to discontinuation/treatment-related TEAEs leading to discontinuation by SOC/PT and severity;
- Provide a detailed list of TEAEs/Treatment-related TEAEs categorized by SOC/PT and severity;
- Laboratory indicators: Provide a cross table for clinical judgment before and after medication, and conduct a descriptive and diachronic analysis of the measured values and changes from the baseline of laboratory test measures before and after medication. Provide a detailed list of examination results;
- Electrocardiogram: Provide a cross-tabulation of clinical judgments before and after medication, and perform descriptive and longitudinal analyses of the measured values and changes from baseline of electrocardiogram measures at each time point before and after medication. Provide a detailed list of the examination results;
- Physical examination: Provide a cross-tabulation of clinical assessments before and after medication, and provide a detailed list of examination results;

- Vital signs: Descriptive and longitudinal analyses will be performed on the measured values and changes from the baseline of various measures before and after medication. A detailed list of examination results before and after medication will also be provided.

9.4.7. Analysis of Concomitant and Non-drug Therapies

- Analysis will be performed as per SS;
- Concomitant medications will be summarized by ATC classification;
- Non-drug therapies will be summarized by SOC and PT;
- Details of concomitant and non-drug therapies will be tabulated.

9.4.8. Analysis of Drug Exposure and Compliance

- Perform the analysis as per SS;
- Drug exposure includes the duration of drug exposure, the total amount of drug used, and the average daily dose. The number and proportion of patients, who have dose reduction, temporary discontinuation, and permanent discontinuation of drug administration, will be listed and analyzed in this study;
- The duration of drug exposure will be calculated in days using the following formula:
  - Exposure will be considered missing if the start or end date is missing. Exposure is calculated as the last date of medication use minus the first date of medication use plus one, minus the number of days of missed or paused medication during that period;
- The average daily dose is defined as the total amount of drug used divided by the drug exposure time;
- Medication adherence is defined as the actual amount of medication used divided by the planned amount of medication to be used, multiplied by 100%;
- Calculation of total drug amount: Sum of planned daily drug dosage during the medication period;
- Calculation of actual drug usage: the sum of the actual daily dosage during the medication period.

9.4.9. Planned Interim Analyses

Not applicable.

9.4.10. Subgroup Analysis

Not applicable.

9.4.11. Multiplicity Issues

Not applicable.

9.4.12. Exploratory Analyses

Not applicable.

10. Supporting Documentation and Operational Considerations

10.1. Regulatory, Ethical, and Study Regulatory Considerations

10.1.1. Informed Consent Process

10.1.1.1. Informed Consent Form and Other Documents Provided to Patients

The informed consent form is an important document that provides detailed information about the study intervention, procedures, and risks. It must be reviewed by an ethics committee and written consent must be obtained before starting the study intervention. During the study, other study documents may also be provided to the patients to collect information, including but not limited to patient diaries.

10.1.1.2. Informed Consent Process and Documentation

Informed consent is a process that begins before an individual agrees to participate in a study and continues throughout their participation. The informed consent document must be approved by an Institutional Review Board (IRB) and patients will be asked to read and review the document. The investigator will explain the study to the patient, including the purpose, procedures, potential risks, and the patient's rights, in a way that is understandable to them and answer any questions they may have. Patients should have adequate time to read and ask questions before signing the written informed consent document. Patients should have the opportunity to discuss the study with family or a representative or consider it themselves before agreeing to participate. The informed consent document must be signed by the patient before

any study procedures are performed. Patients must be informed that participation in the study is voluntary and they can withdraw at any time without harm. The investigator will provide the patient with a copy of the informed consent document for their retention. The informed consent process should be conducted before any study procedures are performed and documented in the source file (including the date) along with the signed informed consent document. The investigator must inform the patient that "refusal to participate in this study will not adversely affect the quality of their medical care" to ensure the patient's rights and welfare.

10.2. Study Suspension and Discontinuation

If there are sufficient and reasonable reasons, this study may be temporarily suspended or terminated early. The party responsible for suspending or terminating the study should provide written notice to the investigator, sponsor, and ethics committee, as well as record the reasons for the suspension or termination of the study. If the study is terminated early or temporarily suspended, the principal investigator should promptly notify the patients, ethics committee, and sponsor, and provide the reasons for the termination or temporary suspension of the study. The investigator will contact the patients and notify them of changes to the visit schedule (if applicable).

10.3. Confidentiality and Privacy

The confidentiality and privacy rights of the patients will be strictly protected by the participating investigators, their staff, and the sponsor and its interventions. This confidentiality will extend not only to clinical information related to the patients but also to tests involving biological sample testing and genetic testing. Therefore, the study plan, documents, data, and all other information generated during the study will be kept strictly confidential. Any information or data related to the study may not be disclosed to unauthorized third parties without the written permission of the sponsor.

All study activities will be conducted in a private setting as far as possible.

Clinical research associate (CRA), other authorized representatives of the sponsor, representatives of the Institutional Review Board (IRB), regulatory authorities or the pharmaceutical company providing the study product may inspect all documents and records

that need to be kept by the study patients, including but not limited to medical records (office, clinic, or hospital) and pharmacy records of the study patients. The clinical study site should allow access to these records.

The contact information of the patients will be securely stored at each clinical study center for internal use during the study. At the end of the study, all records will continue to be stored in a secure location for a duration longer than the time required by IRB review, institutional regulations, or sponsor requirements.

10.4. Future Use of Data

The results and data of this study belong to the sponsor. Both the sponsor and the investigator are prohibited from disclosing any information regarding the agreed terms, progress, results, and related data and materials of this study to third parties or publishing them publicly. The investigator is responsible for informing and requiring their staff to comply with the confidentiality obligations of this study. If either party violates or fails to fulfill any provision of the study contract, the other party may terminate the study immediately by written notice.

The sponsor (or its designee) is responsible for preparing and integrating clinical and safety reports. The sponsor will publish the study results following regulations. Investigators may write academic papers on the study results, publicly publish them, or present them at scientific conferences with the consent of the sponsor.

10.5. Quality Assurance and Quality Control

All parties involved in clinical studies, such as sponsors, study sites, and CROs, should take appropriate quality control measures to ensure that the clinical study complies with the Helsinki Declaration, GCP, relevant laws and regulations, and SOPs.

To ensure the quality of the study, the principal investigator and other key personnel will jointly discuss and develop a clinical study plan prior to the formal start of the study. Relevant medical personnel participating in the study will receive appropriate training.

The testing facility must manage the study treatment following standardized procedures. The personnel responsible for the storage of the study treatment must store it in accordance with the storage conditions specified by the sponsor.

According to the principles of Good Clinical Practice (GCP), necessary steps should be taken during the design and implementation phases of a study to ensure that the collected data is accurate, consistent, complete, and reliable. All observed results and abnormal findings in clinical studies should be promptly verified and recorded to ensure data reliability. The various instruments, equipment, reagents, and standards used in the various examination items in clinical studies should have strict quality standards and ensure that they are working under normal conditions. The investigator fills in the information required by the protocol into the eCRF, which is verified by the CRA for completeness and accuracy, and the study staff at the study site are guided to make necessary corrections and supplements.

During or after the study, representatives from the sponsor's clinical quality assurance department can visit the study site at any time to conduct inspections by relevant regulatory guidelines and company policies. During the inspection, all study records, including the original documents, must be reviewed to check and compare CRFs. However, the personal privacy of the patients must be respected. During sponsor or delegate inspections, investigators and related study personnel must be present and available for consultation.

Regulatory agencies can also conduct similar inspection procedures, either as part of the national clinical study quality management compliance program or to review study results in support of registration data. If the regulatory agency has contacted the investigator to notify them of an upcoming inspection, the investigator should immediately notify the sponsor.

The CRA will conduct regular or as-needed clinical monitoring visits to the study site following the Good Clinical Practice to ensure the quality of the clinical study. The investigator should actively cooperate with the CRA during the monitoring visits.

10.6. Data Processing and Record Retention

10.6.1. Data Collection and Management Responsibilities

Before conducting clinical study data management, a Data Management Plan (DMP) is developed by the data management department based on the actual project situation. The DMP is a dynamic document written by data management personnel based on the clinical study protocol, which details and comprehensively specifies and records the data management tasks

of a specific clinical study, including personnel roles, job responsibilities, operating procedures, etc.

Data Collection and Methods

This study uses an Electronic Data Capture System (EDC) for data collection. Data managers/database programmers create accounts and grant different permissions access to the EDC system based on the user's role.

The data in the eCRF should be recorded from source documents and ensure consistency with the source data. All source documents should be kept clear and tidy to ensure accurate identification of the data. The permanent copy of the research visit record will be considered as the source document for recording the data of the enrolled patients. The data entry personnel should timely and accurately enter the data from the source documents, such as research medical records, into the eCRF.

Data Cleaning and Query Resolution

Data cleaning work includes the process of data verification (systematic and manual logic checks), raising questions, answering questions by investigators/research assistants, data updates, and problem-solving.

The data manager and CRA perform regular data cleaning through the EDC system, while the medical monitor conducts regular medical review work through the EDC system. Investigators/investigator assistants provide answers to EDC questions and/or modify erroneous data online. The questioner confirms the answer data and can repeat the inquiry if necessary.

Refer to the data management plan of this study for the data management tasks that are not fully specified in the protocol.

10.6.2. Retention of Study Records

To ensure the evaluation and supervision of the National Medical Products Administration and the sponsor, investigators should keep all research data by GCP, including all patient source data records, such as study medical records or original records, signed informed consent forms, detailed records of drug distribution, etc. The research data should be kept for at least 5 years after the drug is approved for marketing, and the sponsor is responsible for handling it thereafter.

If required by current regulations or agreements with the sponsor, these data should be kept for a longer period. Destruction of any materials is not allowed without the written consent of the sponsor.

All data from this clinical study is owned by the sponsor. Unless required by the National Medical Products Administration, the investigator may not provide any form of data to a third party without written consent from the sponsor.

10.7. Protocol Violation

If there is a protocol violation, the investigator must notify the CRA and review and discuss the implications of the violation. Any violation must be documented, either as a response to a query in the CRF, in the protocol violation report, or a combination of both. The protocol violation report form will be kept by the sponsor. The protocol violation report and supporting documentation must be retained in the investigator's file.

10.8. Study Publication and Data Sharing Policy

The data and results obtained from this study, as well as the intellectual property of all data and results, belong to the sponsor. Investigators must obtain written consent from the sponsor and discuss with the sponsor before publishing any content derived from the study.

The sponsor acknowledges that the investigator has the right to publish the results after the study is completed. However, the investigator must submit the manuscript or abstract to the sponsor before submission, and the sponsor will review the manuscript for accuracy (to avoid inconsistency with materials submitted to regulatory authorities), ensure that confidential or proprietary information is not disclosed, and supplement relevant information as appropriate. The manuscript will be approved promptly and will not be refused for publication unreasonably. If there is a disagreement between the sponsor and the investigator, the proposed content will be discussed to find a mutually satisfactory solution.

For multicenter studies, the first publication must be based on the data from all centers, and the statistical analysis must be conducted according to the protocol by a biostatistician designated or approved by the sponsor instead of the investigator. Investigators participating in multicenter studies can not publish data collected from one or a few centers prior to the first publication of

data from all centers unless formal agreement has been obtained from all other investigators and the sponsor.

The authors of the manuscript are determined through consultation with various parties. If the submission is for summary data, the authors may include members from each participating center in the study as well as staff from the sponsor.

The publication of study results will be detailed in the clinical study protocol.

11. References

[1] Zhang SW, Sun KX, Zheng RS, Zeng HM, Wang SM, Chen R, Wei WQ, He J. Cancer incidence and mortality in China, 2015[J]. JNCC, 2020. DOI: https://doi.org/10.1016/j.jncc.2020.12.001.

[2] NCCN Clinical Practice Guidelines in Non-Small Cell Lung Cancer (2020 Version 8.0) [DB/OL]. http://www.nccn.org.http://www.nccn.org

[3] Zhou Caicun, Wang Jie, Cheng Ying, et al. (2020) Diagnosis and Treatment Guidelines for Non-small Cell Lung Cancer of the Chinese Society of Clinical Oncology (CSCO).

12. Appendix

Appendix I: ECOG Performance Score

Grade	Performance status
0	Fully active, able to carry on all pre-disease performance without restriction
1	Restricted in physically strenuous activity but ambulatory and able to carry out work of a light or sedentary nature, e.g., light house work, office work
2	Ambulatory and capable of all selfcare but unable to carry out any work activities; up and about more than 50% of waking hours
3	Capable of only limited selfcare; confined to bed or chair more than 50% of waking hours
4	Completely disabled; cannot carry on any selfcare; totally confined to bed or chair
5	Dead

Appendix 3: Excerpt from Response Evaluation Criteria in Solid Tumors (RECIST 1.1)

1. Background

1.1. History of RECIST Criteria

Assessment of the change in tumour burden is an important feature of the clinical evaluation of cancer therapeutics. Both tumour shrinkage (objective response) and time to the development of disease progression are important endpoints in cancer clinical trials. The use of tumour regression as the endpoint for phase II trials screening new agents for evidence of anti-tumour effect is supported by years of evidence suggesting that, for many solid tumours, agents which produce tumour shrinkage in a proportion of patients have a reasonable (albeit imperfect) chance of subsequently demonstrating an improvement in overall survival or other time to event measures in randomised phase III studies. At the current time objective response carries with it a body of evidence greater than for any other biomarker supporting its utility as a measure of promising treatment effect in phase II screening trials. Furthermore, at both the phase II and phase III stage of drug development, clinical trials in advanced disease settings are increasingly utilising time to progression (or progression-free survival) as an endpoint upon which efficacy conclusions are drawn, which is also based on anatomical measurement of tumour size. However, both of these tumour endpoints, objective response and time to disease progression, are useful only if based on widely accepted and readily applied standard criteria based on anatomical tumour burden. In 1981 the World Health Organisation (WHO) first published tumour response criteria, mainly for use in trials where tumour response was the primary endpoint. The WHO criteria introduced the concept of an overall assessment of tumour burden by summing the products of bidimensional lesion measurements and determined response to therapy by evaluation of change from baseline while on treatment. However, in the decades that followed their publication, cooperative groups and pharma ceutical companies that used the WHO criteria often ‘modified’ them to accommodate new technologies or to address areas that were unclear in the original document. This led to confusion in interpretation of trial results and in fact, the application of varying response criteria was shown to lead to very different conclusions about the efficacy of the same regimen. In response to these problems, an International Working Party was formed in the mid 1990s to standardise and simplify response

criteria. New criteria, known as RECIST (Response Evaluation Criteria in Solid Tumours), were published in 2000. Key features of the original RECIST include definitions of minimum size of measurable lesions, instructions on how many lesions to follow (up to 10; a maximum five per organ site), and the use of unidimensional, rather than bidimensional, measures for overall evaluation of tumour burden. These criteria have subsequently been widely adopted by academic institutions, cooperative groups, and industry for trials where the primary endpoints are objective response or progression. In addition, regulatory authorities accept RECIST as an appropriate guideline for these assessments.

2. Purpose of this Guidance

This guideline describes a standard approach to solid tumour measurement and definitions for objective assessment of change in tumour size for use in adult and paediatric cancer clinical trials. It is expected these criteria will be useful in all trials where objective response is the primary study endpoint, as well as in trials where assessment of stable disease, tumour progression or time to progression analyses are under taken, since all of these outcome measures are based on an assessment of anatomical tumour burden and its change on study. There are no assumptions in this paper about the proportion of patients meeting the criteria for any of these endpoints which will signal that an agent or treatment regimen is active: those definitions are dependent on type of cancer in which a trial is being undertaken and the specific agent(s) under study. Protocols must include appropriate statistical sections that define the efficacy parameters upon which the trial sample size and decision criteria are based. In addition to providing definitions and criteria for assessment of tumour response, this guideline also makes recommendations regarding standard reporting of the results of trials that utilise tumour response as an endpoint.

While these guidelines may be applied in malignant brain tumour studies, there are also separate criteria published for response assessment in that setting. This guideline is not intended for use for studies of malignant lymphoma since international guidelines for response assessment in lymphoma are published separately.

Finally, many oncologists, in their daily clinical practice, follow their patients' malignant disease by means of repeated imaging studies and make decisions about continued therapy on

the basis of both objective and symptomatic criteria. It is not intended that these RECIST guidelines play a role in that decision-making, except if determined appropriate by the treating oncologist.

3. Measurability of tumour at baseline

3.1. Definitions

At baseline, the tumor lesions/lymph nodes will be classified as measurable or non-measurable as follows:

3.1.1. Measurable

Tumour lesions: Must be accurately measured in at least one dimension (the longest diameter in the plane of measurement is to be recorded) with a minimum size of:

- 10 mm by CT scan (CT scan slice thickness no greater than 5 mm; see Appendix II on imaging guidance)..
- 10 mm caliper measurement by clinical exam (lesions that cannot be accurately measured with calipers should be recorded as non-measurable).
- 20 mm by chest X-ray.

Malignant lymph nodes: To be considered pathologically enlarged and measurable, a lymph node must be ⩾15 mm in the short axis when assessed by CT scan (CT scan slice thickness recommended to be no greater than 5 mm). At baseline and in follow-up, only the short axis will be measured and followed.

3.1.2. Non-measurable

All other lesions, including small lesions (longest diameter <10 mm or pathological lymph nodes with ⩾10 to <15 mm short axis) as well as truly non-measurable lesions. Lesions considered truly non-measurable include: leptomeningeal disease, ascites, pleural or pericardial effusion, inflammatory breast disease, lymphangitic involvement of skin or lung, abdominal masses/abdominal organomegaly identified by a physical exam that is not measurable by reproducible imaging techniques.

3.1.3. Special considerations regarding lesion measurability

Bone lesions, cystic lesions, and lesions previously treated with local therapy require particular comments:

Bone lesions:.

- Bone scan, PET scan or plain films are not considered adequate imaging techniques to measure bone lesions. However, these techniques can be used to confirm the presence or disappearance of bone lesions.
- Lytic bone lesions or mixed lytic-blastic lesions, with identifiable soft tissue components, that can be evaluated by cross-sectional imaging techniques such as CT or MRI can be considered as measurable lesions if the soft tissue component meets the definition of measurability described above.
- Blastic bone lesions are non-measurable.

Cystic lesions:

- Lesions that meet the criteria for radiographically defined simple cysts should not be considered as malignant lesions (neither measurable nor non-measurable) since they are, by definition, simple cysts.
- 'Cystic lesions' thought to represent cystic metastases can be considered as measurable lesions, if they meet the definition of measurability described above. However, if noncystic lesions are present in the same patient, these are preferred for selection as target lesions.

Lesions with prior local treatment:

- Tumour lesions situated in a previously irradiated area, or in an area subjected to other loco-regional therapy, are usually not considered measurable unless there has been demonstrated progression in the lesion. Study protocols should detail the conditions under which such lesions would be considered measurable.

3.2. Specifications by methods of measurements

3.2.1. Measurement of Lesions

All measurements should be recorded in metric notation, using calipers if clinically assessed. All baseline evaluations should be performed as close as possible to the treatment start and never more than 4 weeks before the beginning of the treatment.

3.2.2. Method of assessment

The same method of assessment and the same technique should be used to characterise each

identified and reported lesion at baseline and during follow-up. Imaging-based evaluation should always be done rather than clinical examination unless the lesion(s) being followed cannot be imaged but are assessable by clinical exam.

Clinical lesions: Clinical lesions will only be considered measurable when they are superficial and ≥10 mm in diameter as assessed using calipers (e.g. skin nodules). For the case of skin lesions, documentation by colour photography including a ruler to estimate the size of the lesion is suggested. As noted above, when lesions can be evaluated by both clinical exam and imaging, imaging evaluation should be undertaken since it is more objective and may also be reviewed at the end of the study.

Chest X-ray: Chest CT is preferred over chest X-ray, particularly when progression is an important endpoint, since CT is more sensitive than X-ray, particularly in identifying new lesions. However, lesions on chest X-ray may be considered measurable if they are clearly defined and surrounded by aerated lungs.

CT, MRI: CT is the best currently available and reproducible method to measure lesions selected for response assessment. This guideline has defined the measurability of lesions on CT scan based on the assumption that CT slice thickness is 5 mm or less. As is described in Appendix II, when CT scans have slice thickness greater than 5 mm, the minimum size for a measurable lesion should be twice the slice thickness. MRI is also acceptable in certain situations (e.g. for body scans).

Ultrasound: Ultrasound is not useful in assessment of lesion size and should not be used as a method of measurement. Ultrasound examinations cannot be reproduced in their entirety for independent review at a later date and because they are operator-dependent, it cannot be guaranteed that the same technique and measurements will be taken from one assessment to the next. If new lesions are identified by ultrasound in the course of the study, confirmation by CT or MRI is advised. If there is concern about radiation exposure at CT, MRI may be used instead of CT in selected instances.

Endoscopy, laparoscopy: The utilisation of these techniques for objective tumour evaluation is not advised. However, they can be useful to confirm complete pathological response when biopsies are obtained or to determine relapse in trials where recurrence following complete

response or surgical resection is an endpoint.

Tumour markers: Tumour markers alone cannot be used to assess objective tumour response. If markers are initially above the upper normal limit, however, they must normalise for a patient to be considered in complete response. Because tumour markers are disease specific, instructions for their measurement should be incorporated into protocols on a disease specific basis. Specific guidelines for both CA-125 response (in recurrent ovarian cancer) and PSA response (in recurrent prostate cancer), have been published. In addition, the Gynecologic Cancer Intergroup has developed CA125 progression criteria which are to be integrated with objective tumour assessment for use in first-line trials in ovarian cancer.

Cytology, histology: These techniques can be used to differentiate between PR and CR in rare cases if required by protocol (for example, residual lesions in tumour types such as germ cell tumours, where known residual benign tumours can remain). When effusions are known to be a potential adverse effect of treatment (e.g. with certain taxane compounds or angiogenesis inhibitors), the cytological confirmation of the neoplastic origin of any effusion that appears or worsens during treatment can be considered if the measurable tumour has met criteria for response or stable disease in order to differentiate between response (or stable disease) and progressive disease.

4. Tumor Response Evaluation

4.1. Assessment of overall tumour burden and measurable disease

To assess objective response or future progression, it is necessary to estimate the overall tumour burden at baseline and use this as a comparator for subsequent measurements. Only patients with measurable disease at baseline should be included in protocols where objective tumour response is the primary endpoint. Measurable disease is defined by the presence of at least one measurable lesion. In studies where the primary endpoint is tumour progression (either time to progression or proportion with progression at a fixed date), the protocol must specify if entry is restricted to those with measurable disease or whether patients having non-measurable disease only are also eligible.

4.2. Baseline documentation of ‘target’ and ‘non-target’ lesions

When more than one measurable lesion is present at baseline all lesions up to a maximum of

five lesions total (and a maximum of two lesions per organ) representative of all involved organs should be identified as target lesions and will be recorded and measured at baseline (this means in instances where patients have only one or two organ sites involved a maximum of two and four lesions respectively will be recorded). For evidence to support the selection of only five target lesions, see analyses on a large prospective database in the article by Bogaerts et al. Target lesions should be selected on the basis of their size (lesions with the longest diameter), be representative of all involved organs, but in addition should be those that lend themselves to reproducible repeated measurements. It may be the case that, on occasion, the largest lesion does not lend itself to reproducible measurement, in which circumstance, the next largest lesion which can be measured reproducibly should be selected.

Lymph nodes merit special mention since they are normal anatomical structures that may be visible by imaging even if not involved by tumour. As noted in Section 3, pathological nodes that are defined as measurable and may be identified as target lesions must meet the criterion of a short axis of ⩾15 mm by CT scan. Only the short axis of these nodes will contribute to the baseline sum. The short axis of the node is the diameter normally used by radiologists to judge if a node is involved in solid tumour. Nodal size is normally reported as two dimensions in the plane in which the image is obtained (for CT scan, this is almost always the axial plane; for MRI the plane of acquisition may be axial, sagittal or coronal). The smaller of these measures is the short axis. For example, an abdominal node that is reported as being 20 mm× 30 mm has a short axis of 20 mm and qualifies as a malignant, measurable node. In this example, 20 mm should be recorded as the node measurement. All other pathological nodes (those with short axis ≥10 mm but <15 mm) should be considered non-target lesions. Nodes that have a short axis <10 mm are considered non-pathological and should not be recorded or followed.

A sum of the diameters (longest for non-nodal lesions, short axis for nodal lesions) for all target lesions will be calculated and reported as the baseline sum diameters. If lymph nodes are to be included in the sum, then as noted above, only the short axis is added into the sum. The baseline sum diameters will be used as reference to further characterise any objective tumour regression in the measurable dimension of the disease.

All other lesions (or sites of disease) including pathological lymph nodes should be identified

as non-target lesions and should also be recorded at baseline. Measurements are not required and these lesions should be followed as 'present', 'absent', or in rare cases 'unequivocal progression' (more details to follow). In addition, it is possible to record multiple nontarget lesions involving the same organ as a single item on the case record form (e.g. 'multiple enlarged pelvic lymph nodes' or 'multiple liver metastases').

4.3. Response criteria

This section provides the definitions of the criteria used to determine objective tumour response for target lesions.

4.3.1. Evaluation of target lesions

Complete Response (CR): Disappearance of all target lesions. Any pathological lymph nodes (whether target or non-target) must have reduction in short axis to <10 mm.

Partial Response (PR): At least a 30% decrease in the sum of diameters of target lesions, taking as reference the baseline sum diameters.

Progressive Disease (PD): At least a 20% increase in the sum of diameters of target lesions, taking as reference the smallest sum on study (this includes the baseline sum if that is the smallest on study). In addition to the relative increase of 20%, the sum must also demonstrate an absolute increase of at least 5 mm. (Note: the appearance of one or more new lesions is also considered progression).

Stable Disease (SD): Neither sufficient shrinkage to qualify for PR nor sufficient increase to qualify for PD, taking as reference the smallest sum diameters while on study .

4.3.2. Special notes on the assessment of target lesions

Lymph nodes. Lymph nodes identified as target lesions should always have the actual short axis measurement recorded (measured in the same anatomical plane as the baseline examination), even if the nodes regress to below 10 mm on study. This means that when lymph nodes are included as target lesions, the 'sum' of lesions may not be zero even if complete response criteria are met, since a normal lymph node is defined as having a short axis of <10 mm. Case report forms or other data collection methods may, therefore, be designed to have target nodal lesions recorded in a separate section where, in order to qualify for CR, each node must achieve a short axis <10 mm. For PR, SD and PD, the actual short axis measurement of the nodes is to

be included in the sum of target lesions.

Target lesions that become 'too small to measure'. While on study, all lesions (nodal and non-nodal) recorded at baseline should have their actual measurements recorded at each subsequent evaluation, even when very small (e.g. 2 mm). However, sometimes lesions or lymph nodes which are recorded as target lesions at baseline become so faint on CT scan that the radiologist may not feel comfortable assigning an exact measure and may report them as being 'too small to measure'. When this occurs it is important that a value be recorded on the case report form. If it is the opinion of the radiologist that the lesion has likely disappeared, the measurement should be recorded as 0 mm. If the lesion is believed to be present and is faintly seen but too small to measure, a default value of 5 mm should be assigned (Note: It is less likely that this rule will be used for lymph nodes since they usually have a definable size when normal and are frequently surrounded by fat such as in the retroperitoneum; however, if a lymph node is believed to be present and is faintly seen but too small to measure, a default value of 5 mm should be assigned in this circumstance as well). This default value is derived from the 5 mm CT slice thickness (but should not be changed with varying CT slice thickness). The measurement of these lesions is potentially non-reproducible; therefore providing this default value will prevent false responses or progressions based upon measurement error. To reiterate, however, if the radiologist is able to provide an actual measure, that should be recorded, even if it is below 5 mm.

Lesions that split or coalesce on treatment. When non-nodal lesions 'fragment', the longest diameters of the fragmented portions should be added together to calculate the target lesion sum; similarly, as lesions coalesce, a plane between them may be maintained that would aid in obtaining maximal diameter measurements of each individual lesion. If the lesions have truly coalesced such that they are no longer separable, the vector of the longest diameter in this instance should be the maximal longest diameter for the 'coalesced lesion'.

4.3.3. Evaluation of non-target lesions

This section provides the definitions of the criteria used to determine the tumour response for the group of non-target lesions. While some non-target lesions may actually be measurable, they need not be measured and instead should be assessed only qualitatively at the time points

specified in the protocol.

Complete Response (CR): Disappearance of all non-target lesions and normalisation of tumour marker level. All lymph nodes must be non-pathological in size (<10 mm short axis).

Non-CR/Non-PD: Persistence of one or more non-target lesion(s) and/or maintenance of tumour marker level above the normal limits.

Progressive Disease (PD): Unequivocal progression of existing non-target lesions. (Note: the appearance of one or more new lesions is also considered progression).

4.3.4. Special notes on assessment of progression of nontarget disease

The concept of progression of non-target disease requires additional explanation as follows: When the patient also has measurable disease. In this setting, to achieve 'unequivocal progression' on the basis of the non-target disease, there must be an overall level of substantial worsening in non-target disease such that, even in presence of SD or PR in target disease, the overall tumour burden has increased sufficiently to merit discontinuation of therapy. A modest 'increase' in the size of one or more non-target lesions is usually not sufficient to quality for unequivocal progression status. The designation of overall progression solely on the basis of change in non-target disease in the face of SD or PR of target disease will therefore be extremely rare.

When the patient has only non-measurable disease.This circumstance arises in some phase III trials when it is not a criterion of study entry to have measurable disease. The same general concepts apply here as noted above. However, in this instance, there is no measurable disease assessment to factor into the interpretation of an increase in non-measurable disease burden. Because worsening in non-target disease cannot be easily quantified (by definition: if all lesions are truly non-measurable) a useful test that can be applied when assessing patients for unequivocal progression is to consider if the increase in overall disease burden based on the change in non-measurable disease is comparable in magnitude to the increase that would be required to declare PD for measurable disease: i.e. an increase in tumour burden representing an additional 73% increase in 'volume' (which is equivalent to a 20% increase diameter in a measurable lesion). Examples include an increase in a pleural effusion from 'trace' to 'large', an increase in lymphangitic disease from localised to widespread, or may be described in

protocols as 'sufficient to require a change in therapy'. If 'unequivocal progression' is seen, the patient should be considered to have had overall PD at that point. While it would be ideal to have objective criteria to apply to non-measurable disease, the very nature of that disease makes it impossible to do so, therefore the increase must be substantial.

4.3.5. New Lesions

The appearance of new malignant lesions denotes disease progression; therefore, some comments on detection of new lesions are important. There are no specific criteria for the identification of new radiographic lesions; however, the finding of a new lesion should be unequivocal: i.e. not attributable to differences in scanning technique, change in imaging modality or findings thought to represent something other than tumour (for example, some 'new' bone lesions may be simply healing or flare of pre-existing lesions). This is particularly important when the patient's baseline lesions show partial or complete response. For example, necrosis of a liver lesion may be reported on a CT scan report as a 'new' cystic lesion, which it is not.

A lesion identified on a follow-up study in an anatomical location that was not scanned at baseline is considered a new lesion and will indicate disease progression. An example of this is the patient who has the visceral disease at baseline and while on study has a CT or MRI brain ordered which reveals metastases. The patient's brain metastases are considered to be evidence of PD even if he/she did not have brain imaging at baseline.

If a new lesion is equivocal, for example, because of its small size, continued therapy and follow-up evaluation will clarify if it represents a truly new disease. If repeat scans confirm there is definitely a new lesion, then progression should be declared using the date of the initial scan. While FDG-PET response assessments need additional study, it is sometimes reasonable to incorporate the use of FDG-PET scanning to complement CT scanning in assessment of progression (particularly possible 'new' disease). New lesions on the basis of FDG-PET imaging can be identified according to the following algorithm:

a. Negative FDG-PET at baseline, with a positive FDG-PET at follow-up, is a sign of PD based on a new lesion.

b. No FDG-PET at baseline and a positive FDG-PET at follow-up: If the positive FDG-PET at

follow-up corresponds to a new site of disease confirmed by CT, this is PD. If the positive FDG-PET at follow-up is not confirmed as a new site of disease on CT, additional follow-up CT scans are needed to determine if there is true progression occurring at that site (if so, the date of PD will be the date of the initial abnormal FDG-PET scan). If the positive FDG-PET at follow-up corresponds to a pre-existing site of disease on CT that is not progressing on the basis of the anatomic images, this is not PD.

4.4. Evaluation of best overall response

The best overall response is the best response recorded from the start of the study treatment until the end of treatment taking into account any requirement for confirmation. On occasion a response may not be documented until after the end of therapy so protocols should be clear if post-treatment assessments are to be considered in determination of best overall response. Protocols must specify how any new therapy introduced before progression will affect best response designation. The patient's best overall response assignment will depend on the findings of both target and non-target disease and will also take into consideration the appearance of new lesions. Furthermore, depending on the nature of the study and the protocol requirements, it may also require confirmatory measurement (see Section 4.6). Specifically, in non-randomised trials where response is the primary endpoint, confirmation of PR or CR is needed to deem either one the 'best overall response'. This is described further below.

4.4.1. Time point response

It is assumed that at each protocol-specified time point, a response assessment occurs. Table 1 on the next page provides a summary of the overall response status calculation at each time point for patients who have measurable disease at baseline.

When patients have non-measurable (therefore non-target) disease only, Table 2 is to be used.

4.4.2. Missing assessments and inevaluable designation

When no imaging/measurement is done at all at a particular time point, the patient is not evaluable (NE) at that time point. If only a subset of lesion measurements are made at an assessment, usually the case is also considered NE at that time point, unless a convincing argument can be made that the contribution of the individual missing lesion(s) would not change the assigned time point response. This would be most likely to happen in the case of PD. For

example, if a patient had a baseline sum of 50 mm with three measured lesions and at follow-up only two lesions were assessed, but those gave a sum of 80 mm, the patient will have achieved PD status, regardless of the contribution of the missing lesion.

4.4.3. Best overall response: all time points

The best overall response is determined once all the data for the patient is known. Best response determination in trials where confirmation of complete or partial response IS NOT required: Best response in these trials is defined as the best response across all time points (for example, a patient who has SD at first assessment, PR at second assessment, and PD on last assessment has a best overall response of PR). When SD is believed to be best response, it must also meet the protocol specified minimum time from baseline. If the minimum time is not met when SD is otherwise the best time point response, the patient's best response depends on the subsequent assessments. For example, a patient who has SD at first assessment, PD at second and does not meet minimum duration for SD, will have a best response of PD. The same patient lost to follow-up after the first SD assessment would be considered inevaluable. Best response determination in trials where confirmation of complete or partial response IS required: Complete or partial responses may be claimed only if the criteria for each are me at a subsequent time point as specified in the protocol (generally 4 weeks later). In this circumstance, the best overall response can be interpreted as in Table 3.

4.4.4. Special notes on response assessment

When nodal disease is included in the sum of target lesions and the nodes decrease to 'normal' size (<10 mm), they may still have a measurement reported on scans. This measurement should be recorded even though the nodes are normal in order not to overstate progression should it be based on increase in size of the nodes. As noted earlier, this means that patients with CR may not have a total sum of 'zero' on the case report form (CRF).

In trials where confirmation of response is required, repeated 'NE' time point assessments may complicate best response determination. The analysis plan for the trial must address how missing data/assessments will be addressed in determination of response and progression. For example, in most trials it is reasonable to consider a patient with time point responses of PR-NE-PR as a confirmed response.

Patients with a global deterioration of health status requiring discontinuation of treatment without objective evidence of disease progression at that time should be reported as 'symptomatic deterioration'. Every effort should be made to document objective progression even after discontinuation of treatment. Symptomatic deterioration is not a descriptor of an objective response: it is a reason for stopping study therapy. The objective response status of such patients is to be determined by evaluation of target and non-target disease as shown in Tables 1–3.

Conditions that define 'early progression, early death and inevaluability' are study specific and should be clearly described in each protocol (depending on treatment duration, treatment periodicity).

In some circumstances it may be difficult to distinguish residual disease from normal tissue. When the evaluation of complete response depends upon this determination, it is recommended that the residual lesion be investigated (fine needle aspirate/biopsy) before assigning a status of complete response. FDG-PET may be used to upgrade a response to a CR in a manner similar to a biopsy in cases where a residual radiographic abnormality is thought to represent fibrosis or scarring. The use of FDG-PET in this circumstance should be prospectively described in the protocol and supported by disease-specific medical literature for the indication. However, it must be acknowledged that both approaches may lead to false positive CR due to limitations of FDG-PET and biopsy resolution/sensitivity.

For equivocal findings of progression (e.g. very small and uncertain new lesions; cystic changes or necrosis in existing lesions), treatment may continue until the next scheduled assessment. If at the next scheduled assessment, progression is confirmed, the date of progression should be the earlier date when progression was suspected.

Table 1: Time point response: patients with target (+/– non-target) disease.

Target Lesions	Non-Target Lesions	New Lesions	Overall response
CR	CR	No	CR
CR	Non-CR/Non-PD	No	PR
CR	Not evaluable	No	PR
PR	Non-progression or	No	PR

	incomplete assessment		
SD	Non-progression or incomplete assessment	No	SD
Not fully evaluated	Non-progression	No	NE
PD	Any situation	Yes or No	PD
Any situation	PD	Yes or No	PD
Any situation	Any situation	Yes	PD
CR = complete response	PR = partial response	SD = stable disease	PD = progressive disease<br>NE = inevaluable

In SD cases, at least one follow-up measurement after enrollment must meet the SD criteria, and the follow-up interval should be at least 6-8 weeks after enrollment.

Table 2 Time point response: patients with non-target disease only.

Non-Target Lesions	New Lesions	Overall response
CR	No	CR
Non-CR or non-PD	No	Non-CR or Non-PD
Not all evaluated	No	NE
Unequivocal PD	Yes or No	PD
Any	Yes	PD

Note: CR = complete response, PD = progressive disease, and NE = inevaluable. a 'Non-CR/non-PD' is preferred over 'stable disease' for non-target disease since SD is increasingly used as endpoint for assessment of efficacy in some trials so to assign this category when no lesions can be measured is not advised.

Table 3 – Best overall response when confirmation of CR and PR required

Overall response first time point	Overall response subsequent time points	Best overall response
CR	CR	CR
CR	PR	SD, PD or PR[a]
CR	SD	SD provided minimum criteria for SD duration met, otherwise, PD.
CR	PD	SD provided minimum criteria for SD duration met, otherwise, PD.
CR	NE	SD provided minimum criteria for SD duration met,

		otherwise, NE.
PR	CR	PR
PR	PR	PR
PR	SD	SD
PR	PD	SD provided minimum criteria for SD duration met, otherwise, PD.
PR	NE	SD provided minimum criteria for SD duration met, otherwise, PD.
NE	NE	NE

Note: CR = complete response, PR = partial response, SD = stable disease, PD = progressive disease, and NE = inevaluable. a If a CR is truly met at first time point, then any disease seen at a subsequent time point, even disease meeting PR criteria relative to baseline, makes the disease PD at that point (since disease must have reappeared after CR). Best response would depend on whether minimum duration for SD was met. However, sometimes 'CR' may be claimed when subsequent scans suggest small lesions were likely still present and in fact the patient had PR, not CR at the first time point. Under these circumstances, the original CR should be changed to PR and the best response is PR.

4.5. Frequency of tumour re-evaluation

Frequency of tumour re-evaluation while on treatment should be protocol specific and adapted to the type and schedule of treatment. However, in the context of phase II studies where the beneficial effect of therapy is not known, follow-up every 6–8 weeks (timed to coincide with the end of a cycle) is reasonable. Smaller or greater time intervals than these could be justified in specific regimens or circumstances. The protocol should specify which organ sites are to be evaluated at baseline (usually those most likely to be involved with metastatic disease for the tumour type under study) and how often evaluations are repeated. Normally, all target and non-target sites are evaluated at each assessment. In selected circumstances certain non-target organs may be evaluated less frequently. For example, bone scans may need to be repeated only when complete response is identified in the target disease or when progression in bone is suspected.

After the end of the treatment, the need for repetitive tumour evaluations depends on whether the trial has as a goal the response rate or the time to an event (progression/death). If 'time to an event' (e.g. time to progression, disease-free survival, progression-free survival) is the main

endpoint of the study, then a routine scheduled re-evaluation of protocol-specified sites of disease is warranted. In randomised comparative trials in particular, the scheduled assessments should be performed as identified on a calendar schedule (for example: every 6–8 weeks on treatment or every 3–4 months after treatment) and should not be affected by delays in therapy, drug holidays or any other events that might lead to an imbalance in a treatment arm in the timing of disease assessment.

4.6. Confirmatory measurement/duration of response

4.6.1. Confirmation

In non-randomised trials where response is the primary endpoint, confirmation of PR and CR is required to ensure responses identified are not the result of measurement error. This will also permit appropriate interpretation of results in the context of historical data where response has traditionally required confirmation in such trials. However, in all other circumstances, i.e. in randomised trials (phase II or III) or studies where stable disease or progression are the primary endpoints, confirmation of response is not required since it will not add value to the interpretation of trial results. However, elimination of the requirement for response confirmation may increase the importance of central review to protect against bias, in particular in studies which are not blinded.

In the case of SD, measurements must have met the SD criteria at least once after study entry at a minimum interval (in general, not less than 6–8 weeks) that is defined in the study protocol.

4.6.2. Duration of overall response

The duration of overall response is measured from the time measurement criteria are first met for CR/PR (whichever is first recorded) until the first date that recurrent or progressive disease is objectively documented (taking as reference for progressive disease the smallest measurements recorded on study).

The duration of overall complete response is measured from the time measurement criteria are first met for CR until the first date that recurrent disease is objectively documented.

4.6.3. Duration of stable disease

Stable disease is measured from the start of the treatment (in randomised trials, from date of randomisation) until the criteria for progression are met, taking as reference the smallest sum

on study (if the baseline sum is the smallest, this is the reference for calculation of PD).

The clinical relevance of the duration of stable disease varies in different studies and diseases. If the proportion of patients achieving stable disease for a minimum period of time is an endpoint of importance in a particular trial, the protocol should specify the minimal time interval required between two measurements for determination of stable disease.

Note: The duration of response and stable disease as well as the progression-free survival are influenced by the frequency of follow-up after baseline evaluation. It is not in the scope of this guideline to define a standard follow-up frequency. The frequency should take into account many parameters including disease types and stages, treatment periodicity and standard practice. However, these limitations of the precision of the measured endpoint should be taken into account if comparisons between trials are to be made.

4.7. Progression-free survival/proportion progression-free

4.7.1. Phase II trials

This guideline is focused primarily on the use of objective response endpoints for phase II trials. In some circumstances, ‘response rate’ may not be the optimal method to assess the potential anticancer activity of new agents/regimens. In such cases ‘progression-free survival’ (PFS) or the ‘proportion progression-free’ at landmark time points, might be considered appropriate alternatives to provide an initial signal of biologic effect of new agents. It is clear, however, that in an uncontrolled trial, these measures are subject to criticism since an apparently promising observation may be related to biological factors such as patient selection and not the impact of the intervention. Thus, phase II screening trials utilising these endpoints are best designed with a randomised control. Exceptions may exist where the behaviour patterns of certain cancers are so consistent (and usually consistently poor), that a non-randomised trial is justifiable. However, in these cases it will be essential to document with care the basis for estimating the expected PFS or proportion progression-free in the absence of a treatment effect.
